# Supplementary material for: Transcriptional Regulation of Plant Biomass Degradation and Carbohydrate Utilization Genes in the Extreme Thermophile Caldicellulosiruptor bescii
Source: mSystems. 2021 Jun 1;6(3):e01345-20. doi: 10.1128/mSystems.01345-20 (PMC8579813; doi:10.1128/mSystems.01345-20)
Supplement: FIG S1 [file msystems.01345-20-sf001.pdf]

Figure S1

31899.10.peg.2835 (Athe_2267) *axgR-axgG2* AraR-binding site **(repressor)**

31899.10.peg.2835	Caldicellulosiruptor bescii strain DSMZ 6725	1121259.3.peg.1214	Caldicellulosiruptor acetigenus DSM 7040
632348.3.peg.408	Caldicellulosiruptor kronotskyensis 2002	1214564.3.peg.2167	Caldicellulosiruptor sp. F32
632292.3.peg.531	Caldicellulosiruptor hydrothermalis 108	1387557.3.peg.2675	Caldicellulosiruptor sp. Wai35.B1
632518.3.peg.2079	Caldicellulosiruptor owensensis OL	351627.8.peg.279	Caldicellulosiruptor saccharolyticus DSM 8903

```
31899.10.peg.2835  ttattttgaacgatgttatcaaaagtgttcagattgttgacaggcttgataaaatgtaatacactgaaGaaaagaaa-a
632348.3.peg.408   ttattttgaacgatgttatcaaaagtgttcagattgttgacaggcttgataaaatgtaatacactgaagaaaagaaa-a
632292.3.peg.531   ttttcttgaacaatgttatcaaaaatgttcagcttgttgacaggcctgataaaatgtaatacactgaagaaaagaaa-a
1121259.3.peg.1214 tttttttgaacgatgttatcaaaagtgttcagcttgttgacaggcctgataaaatgtaatacactgaagaaaagaaa-a
632518.3.peg.2079  tttttttgattaatgttatcaaaagtgttcagtttattgacaggtctgatgaaatgtaataaactgaaaaaaagaaa-a
1214564.3.peg.2167 tttttttgaacgttgttatcaaaagtgttcaccatgttgacagacttgataaaatataatacactgatggaaagaagga
351627.8.peg.279   tttttttgaacgttgttatcaaaagtgttcaccatgttgacagacttgatagaatataatacactgatggaaagaagga
1387557.3.peg.2675 tttttttgaacgttgttatcaaaagtgttcattatgttgacatacttgataaaatataatacactgatggaaagaaaga
                   ** * ****    *********** ******   * ******    ****  *** ***** *****   ******  *

31899.10.peg.2835  cgtattaaacttgttcacatacaactttttcggtgatataattttcatataacttgcaaaagtggtgaatattg-ATGCTTGT
632348.3.peg.408   cgtattaaacttgttcacatacaactttttcggtgatataattttcatataaattgcaaaagtggtgaatattg-ATGCTTGT
632292.3.peg.531   tatatcaaacttgttcacatacaacttttttggtggtataatagcataagaacttgcaaaagtggtgaatattg-ATGCTTGT
1121259.3.peg.1214 tatatcaaacttgttcacatacaacttttttagtggtataatagcataagagtttgcaaaagtggtgaatattg-ATGCTTGT
632518.3.peg.2079  tatatcaaacttgttcacatacaactttttaagtggtataatatcataagagtttgcaaaagtggtgaatattg-ATGCTTGT
1214564.3.peg.2167 tatataaaagttgtacgtgtacaacttttttcaaaaccaaaaatgatacaa--tcgtaaaagtggtgaatattaaATGCTTGT
351627.8.peg.279   tatataaaagttgtatgtgtacaacttttttcaaagccaaaaatgatacaa--tcgtaaaagtggtgaatattaaATGCTTGT
1387557.3.peg.2675 tttataaaagttgtatgtgtacaacttttttcaaagccaaaaatgatacaa--ttgtaaaagtggtgaatattaaATGCTTGT
                     *** *** ****     ***********         **         *  * * ****************  ********
```

31899.10.peg.2833 (Athe_2266) *axgGHF* AxgR-binding site **(potential repressor)**

31899.10.peg.2833	Caldicellulosiruptor bescii strain DSMZ 6725	632518.3.peg.2076	Caldicellulosiruptor owensensis OL
632348.3.peg.409	Caldicellulosiruptor kronotskyensis 2002	1387557.3.peg.2674	Caldicellulosiruptor sp. Wai35.B1
632292.3.peg.533	Caldicellulosiruptor hydrothermalis 108	1214564.3.peg.2165	Caldicellulosiruptor sp. F32
1121259.3.peg.1216	Caldicellulosiruptor acetigenus DSM 7040	351627.8.peg.280	Caldicellulosiruptor saccharolyticus DSM 8903

```
31899.10.peg.2833  gttcaaaataataaaaattttttgattaaaaaagaaggattttgattttggtgtagaatatatataaaTagaaaagcaaa
632348.3.peg.409   gttcaaaataataaaaattttttgattaaaaaagaaggattttgattttggtgtagaatatatataaatagaaaagcaaa
632292.3.peg.533   gttcaagaaaataaaatttttttgattaaaaaagaaggattttgattttggtgtagaatatatataaatagaaaagcgaa
1121259.3.peg.1216 gttcaaaaaaataaaaattttttgattaaaaaagaaggattttgattttggtgtagaatatatataaatagaaaagcaaa
632518.3.peg.2076  aatcaaaaaaataaaaaattttgattaaaaaagaaggattttgattttgatgtagaatatatataaatagagaaacgaa
1214564.3.peg.2165 gttcaaaaaaataaaaaattttgattaaaaaagaaggattttgatttgaatgtagaatatatataattacaagacacaa
351627.8.peg.280   gttcaaaaaaataaaaaattttgattaaaaaagaaggattttgatttgaatgtagaatatatataattacaagacacaa
1387557.3.peg.2674 gttcaaaaaaataaaaaattttgattaaaaaagaaggattttgatttgaatgtagaatatatataattacaagacacaa
                     **** * *******  ******************************   *************** ** *  *   **

31899.10.peg.2833  tgataaatcag-agtaaatagatttaccaaaactacaataat-ttttaacaaagcgaggtgagatttgcATGAAAAGCTCCACTGATA
632348.3.peg.409   tgataaatcag-agtaaatagatttaacaaaactacaataat-ttttaacaaagcgaggtgagatttgcATGAAAAGCTGCACTGATA
632292.3.peg.533   agatggataca-aataaatagttttaccaaaactactataat-ctttaacaaagcgaggtgagatttgcATGAAAAGCTCCACTGATA
1121259.3.peg.1216 agatagatgca-agtaaatagttttaccaagactactataat-ttttaacaaagcgaggtgagatttgcATGAAAAGCTCCACAGATG
632518.3.peg.2076  aaatgaatgag-gatagatagttttaccaggaatgctataat-ttttaacaaagcgaggtgagatttgcATGAAAAGCTCCACTGATA
1214564.3.peg.2165 aattaaatcaacaatgcacctgattaaccaaaagtttgtaatgctaaaacaaagcgaggtga-acctgtGTGAAGGATTCTGCATCTG
351627.8.peg.280   aattaaatcaaccatgcacctgattaaccaaaagtttgtaatgctaaaacaaagcggggtga-aatagcGTGAAGGATTCTGCATCTG
1387557.3.peg.2674 aattaaatcaaccatgcacatgattaaccaaaagtttgtaatgctaaaacaaagcgaggtga-acctgtGTGAAGGATTCTGCATCTG
                     *  **      *  *     *** *    *      ****  *  ********* ***** *   *  ****     *   *   *
```

31899.10.peg.2793 (Athe_2229) ***araB-araR-araD*** AraR-binding site **(potential repressor)**

31899.10.peg.2793	Caldicellulosiruptor bescii strain DSMZ 6725	1387557.3.peg.2638	Caldicellulosiruptor sp. Wai35.B1
1121259.3.peg.1252	Caldicellulosiruptor acetigenus DSM 7040	1214564.3.peg.364	Caldicellulosiruptor sp. F32
1222016.3.peg.2413	Caldicellulosiruptor changbaiensis strain CBS-Z	608506.3.peg.2142	Caldicellulosiruptor obsidiansis OB47
351627.8.peg.827	Caldicellulosiruptor saccharolyticus DSM 8903	632518.3.peg.2034	Caldicellulosiruptor owensensis OL
632348.3.peg.445	Caldicellulosiruptor kronotskyensis 2002	632292.3.peg.586	Caldicellulosiruptor hydrothermalis 108

```
31899.10.peg.2793  aaatagctatatgtg---ttgaaaaactaatgcaaaatgaagaatttgttttaaaacctcttcccaagtttctggaaaacaacaaacttcttttaggaac
632348.3.peg.445   aaatagctatatgtg---ttgaaaaactaatgcaaaatgaagaatttgttttaaaacctcttcccaagtttctggaaaacaacaaacttcttttaggaac
1121259.3.peg.1252 agatagcagtatgtg---tagataaacttatgcaaaatgaggaatttgttttaaaaccacttccaaaagcactggaaaaaaacaagctccttttggggac
632518.3.peg.2034  agattgctttatgtg---ttgagaaacttatgcaaaatgaggagtttgttttaaaacctcttccaaaggcacttgaaaataacaaacttcttcttggaac
608506.3.peg.2142  agattgctttatgtg---ttgaaaaactaatgcaaaatgaagaatttgttttaaaacccccttccaaaagtgctggaaaacaataaacttcttcttggaac
1387557.3.peg.2638 agatagtactatgttt-tcatattttcccatataaaaaagataattacttataaaaa----ttaaaaagc--------aaagtaaacttcttttagccac
632292.3.peg.586   agatagtactgtgttctcatattttcccatatacaatagaaaattacttacaaaaaa---ttaaaaagc--------aaaataaacttcttttaggcac
1222016.3.peg.2413 agattaaatctttctc----ggagtattaacactttctatataaaattatatcactcttttttaaaaaagt-------gaccattcgttttgtttttttcgc
351627.8.peg.827   agattaaatctttctc----ggaatattaacactttctatataaaattatatcactcttttttaaaaagt-------gaccattcgttttgtttttttcgc
                   * **       *                 *            *      *   *       *   **           *        *   * *     *

31899.10.peg.2793  aacaattgattatgaaggcaatgtgatacttgtattgaatccagaatattttaggctctaaaagagaaagagcattaaattgtccgcgtacaactttttt
632348.3.peg.445   aacaattgattatgaaggcaatgtgatacttgtattgaatccagaatattttaggctctaaaagagaaggagcattaaattgtccgcgtacaactttttt
1121259.3.peg.1252 aactattgactatgaaggaaatgttatacttgtgttaaatcccgaatattttaggctctaaaagagaagga-tgtgaaattgttcacgtacaactttttt
632518.3.peg.2034  aactatagactatgaaggaaatgttatacttgtcttgaatcctgaatatttcaggctttgaaagagaagga-tgtgaaattgttcacgtacaactttttt
608506.3.peg.2142  aactatagactatgaaggaaatgttatacttgtgttgaatcctgaatatttcaggctttgaaagagaagga-tgtgaaattgttcacgtacaactttttt
1387557.3.peg.2638 ttcgattgactacgaaggaaaggtaattttggtattgaatccagcgcattttagaaactaaaagagaagga-tgtgaaattgttcacgtacaactttttt
632292.3.peg.586   ttcgactgactacgatggaaaggtaattttggtattgaatccagggtattttataatctaaaagagaagga-tgtgaaattgttcacgtacaactttttt
1222016.3.peg.2413 t-cttttaattctt---ccatctttttcccataaatcaactttgtgtaaaa-atggtttgagtggaatacatacttaaattgtatatatacaactttttt
351627.8.peg.827   t-ctttcagttctt---ctatcttttttcctataaaacaactttgtgtaaaa-ataatttgagtggaatgtatgcttaaattgtatatatacaactttttt
                     *       *        *   *  *          **     *   *    *     * *  *  *   *   * *******     ************

31899.10.peg.2793  tgctccttctcttttgttctttttgcatatatgatactatattttta-a-gaaacatgtatgcacaagcaaa-ggggaaaaggggggattttttATGGCAAA
632348.3.peg.445   tgctccttctcttttgttctttttgcatatatgatactatattttta-aagaaacatgtatgcacaagcaaa-ggggaaaaggggggattttttATGGCAAA
1121259.3.peg.1252 cactccttctcttttgttctttttgcatatatgctactatattttta-aagaaacatgtatgcacaagcaaa-ggagaaaaggggggattttttATGGCAAA
632518.3.peg.2034  cactccttctcttttgttctttttgcatatatgttactatattttttagaaaaaacatgtatgcacaggcaaa-ggagaaaaggggggattttttATGCCAAA
608506.3.peg.2142  cactccttctcttttgttctttttgcatatatgctactatattttta-aagaaacatgtatgcacaagcaaa-ggggaaaaggggggattttttATGGCAAA
1387557.3.peg.2638 cactccttctcttttgttctttttgtatatatgttactatattctta-aagaaacatgtatgcacaagcaaa-ggagagaaggggggattttttATGGCAAA
632292.3.peg.586   cactccttctcttttgttctttttgcatatatgttactatattttta-aagaaacatgtatgcacaagcaaaaggggaaaaggggggattttttATGGCAAA
1222016.3.peg.2413 tatcctttctcttttgttctttttgcatatatgctactatattttta-aagaaacatgtatgcacaagcaaaaggggaaaaggggggattttttATGGCAAA
351627.8.peg.827   tatcctctctcttttgttctttttgcatatatgctactatattttta-aagaaacatgtatgcacaagcaaaaggggaaaaggggggattttttATGGCAAA
                       *  ****************** ******* ********* *** *  *************** ***** ** ** **************** ****
```

31899.10.peg.1161 (Athe_0707) *araI* AraR-binding site (potential activator)

31899.10.peg.1161	Caldicellulosiruptor bescii strain DSMZ 6725	1387557.3.peg.975	Caldicellulosiruptor sp. Wai35.B1
632292.3.peg.2052	Caldicellulosiruptor hydrothermalis 108	632518.3.peg.625	Caldicellulosiruptor owensensis OL
632348.3.peg.2026	Caldicellulosiruptor kronotskyensis 2002	1222016.3.peg.2247	Caldicellulosiruptor changbaiensis strain CBS-Z
1121259.3.peg.920	Caldicellulosiruptor acetigenus DSM 7040	1387569.3.peg.802	Thermoanaerobacter cellulolyticus NA10
608506.3.peg.727	Caldicellulosiruptor obsidiansis OB47	351627.8.peg.995	Caldicellulosiruptor saccharolyticus DSM 8903

```
31899.10.peg.1161  aaagttttattttaaaagtg----gattttgaaaagtttgcttcccaacttgtatgtgaacaatttt-ttgattttaactggaatataatggaattatt
632292.3.peg.2052  aaagttttattttaaaagtg----gattttgaaaagtttgcttcccaacttgtatgcgaacaatttt-ttaattcaaaatggaatataatggaattatt
632348.3.peg.2026  aaagttttattttaaaagtg----gattttgaaaagtttgctttccaacttgtatgcgaacaatttt-ttaattcaaaatggaatattatggaattatt
1121259.3.peg.920  aaagttttattttaaaagtg----gattttgaaaagtttgtttcccaacttgtatgtgaacaatttt-ttgattttaaatggaatataatggaattatt
1387557.3.peg.975  aaagttcttattttaaaagta----gattttgaaaagtttacttctcaacttgtatgtgaacaatttt-ttaattttaaatggaatataatggtattatt
1222016.3.peg.2247 aaagtttctattttaaaagta----gattg-aaaagatttttcccgcacttgtacgtgaacaattttcttacaatgatatggcattttgtgggaataat
351627.8.peg.995   aaagtttctattttaaaagta----gattg-aaaagatttttcccgcacttgtacgtgaacaattttcttacaatgatatggtattttgtgggaataat
1387569.3.peg.802  aaagttttattttaaaagta----gattg-aaaagatttttacctcacttgtacatgaacaactttcttaaaatgatatggcattttatggcaataat
608506.3.peg.727   caaattcttacaaacacattataaaaagctacaaagtttc-acacttaacttgtatgtgaacaattttttaattataactggaatataatggtattaat
632518.3.peg.625   aaaattcttacaaatatattataaaaagctacaaagttttatacttaacttgtatgtgaacaattttttaattataaatggaatatgatggaattaat
                    ** **  **      * * *      *     ***  **         *******   ****** *** **       *  *** ** *  *** * ** *

31899.10.peg.1161  ttattgtatgcacaagttgaaaggtatataattaaattAtaaaaacatttccgaatttataagaacacttgggagggaatat-ttaagATGATTCAAA
632292.3.peg.2052  ttattgtatgcacaagttgaaaggtatataatttaattataaaaccatttccgatttaaaatgaacatttgggagggaatat-ttaagATGATTCAAA
632348.3.peg.2026  ttattgtatgcacaagttgaaaggtatataatttaattat------------gatttaaaatgaacatttgggagggaacat-ttaagATGATTCAAA
1121259.3.peg.920  ttattgtatgcacaagttgaaaggtatataatttaattataaaaccatttctgatttaaaatgaacatttgggagggaatat-taaaaATGCTGCAAA
1387557.3.peg.975  ttattgtatgcacaagtttaaagatatataatttaattataaaatcatttctgatttaaaatgaacaattgggagggaatat-caaaaATGCTGCAAA
1222016.3.peg.2247 ttattgtatgcacaagcccaaagttatataatt--attataacaaaacataaaaacttaatgaaatgttaggagggattttt-tcaatATGCTACAAA
351627.8.peg.995   ttattgtatgcacaagcccaaagttatataatt--attataacaaaacataaaaacttaatgaaatgttaggagggattttt-tcaatATGCTACAAA
1387569.3.peg.802  ttattgtacgtacaagcacaaagctatataatt--attataacaaagcgtaaaaacttaatgaaatgttaggagggattatt-tcaaaATGTTACAAA
608506.3.peg.727   ttattgtatgcacaagttgaaaagtatataatttgattataaggacatttccgaatttacaagaacatttgggagggaatat-ttgaaATGATTCAGA
632518.3.peg.625   ttattgtatgcacaagttgaaaagtatataatttaattataaaaacatttctgaatttacaagaacattggggagggaatatcttaagATGATTCAAA
                   ******** * *****   ***  *********  *****              *  * *    **   * **  **      *    * *** * ** *
```

31899.10.peg.1583 (Athe_1102) *arbA* AraR-binding site ; ArbA-binding site (potential repressor)

31899.10.peg.1583	Caldicellulosiruptor bescii strain DSMZ 6725	632348.3.peg.1681	Caldicellulosiruptor kronotskyensis 2002
632292.3.peg.1727	Caldicellulosiruptor hydrothermalis 108	632518.3.peg.991	Caldicellulosiruptor owensensis OL
351627.8.peg.1715	Caldicellulosiruptor saccharolyticus DSM 8903	1121259.3.peg.1106	Caldicellulosiruptor acetigenus DSM 7040
1222016.3.peg.1854	Caldicellulosiruptor changbaiensis strain CBS-Z	632335.8.peg.1211	Caldicellulosiruptor kristjanssonii 177R1B
608506.3.peg.1531	Caldicellulosiruptor obsidiansis OB47	632516.3.peg.1446	Caldicellulosiruptor lactoaceticus 6A

```
31899.10.peg.1583  gatagcattcataaaaatcaagtttgt---tattaaaacttttaataa------aaa-cttgtccacatacaagctaactcagtttaagttcataa
632335.8.peg.1211  gatagcattcaaaaaaatcaagtttgt---tattaaaacttttaataa------aaa-cttgtccacatacaagctaactcagtttaagttcataa
632516.3.peg.1446  gatagcattcaaaaaaatcaagtttgt---tattaaaacttttaataa------aaa-cttgtccacagacaagctaactcagtttaagttcataa
632348.3.peg.1681  gacagcattcaaaaaagtcaagttctt---tattaaaacttttaataa------aaa-cttgtatacatacaagttaactcagtttaaattcgtaa
608506.3.peg.1531  cttcttaaaatttttaataattcttgtacatatacaagttaaatatag----ctaa-ttaattttaaatcttttaaaaatcaacatatttt-acac
632518.3.peg.991   tttttttaatttttttataaatcttgtacacgtacaagttaa-tatag----ccaaatttattttaaa-catttaaaaatcaatttatttttacac
632292.3.peg.1727  ttttttaa------ttttttattta-----ttatctaccttcattt------ta------gcttttttaaaaaacatt---taaaattttatat
351627.8.peg.1715  tattttaa------ttttttatttacatattaacacatttttgctt------tattcaaagtttttattttagtttattgaataaaattttagat
1222016.3.peg.1854 tatttcaaaaaaaattatagaacttgtatatgtacaagtaactaaatagcaaatatatttagtttttaaaaatttatttgaataaaactgaaaat
1121259.3.peg.1106 tatttcaaaaaaaattatagaacttgtacatgtacaagtaactaaatagcaaatatatttagtttttaaaaatttatttgaataaaactgaaaat
1214564.3.peg.1113 agccatatttgagataatcaagtttac---tgttaaaacttttataa------aaaacttgtatatatacaagttt-ctcagctttgatttacaa
1387557.3.peg.1339 atctttgatt--agttgtgacttctaca---atgcaggtgtattctaa-----taaa-aaatttttcctacaaact-------cttgacactatca
                                    *                                     *

31899.10.peg.1583  gcttttgctcatttcttgcaaattattttaaaaccagctaaaataaaaatGttcaaatc---tataaaaaa-gggtgg------aataaaaatcATGAAACAAA
632335.8.peg.1211  gcttttgctcatttcttgcaaattattttaaaaccagctaaaataaaaatgttcaaacc---tataaaaaaagggtgg------aataaaaatcATGAAACAAA
632516.3.peg.1446  gcttttgctcatttcttgcaaattattttaaaaccagctaaaataaaaatgttcaaatc---tataaaaaa-gggtgg------aataaaaatcATGAAACAAA
632348.3.peg.1681  gtttttacttgtttcttgcaaattattttaaaaccagctaaaataaaaatgtttaaatc---tataaaaaa-gggtgg------aataaaaatcATGAAACAAA
608506.3.peg.1531  atcactacttccttcttgcaaagtattttaaaaccagctaaaataaaaatgttcaaatc---tacaaaaaa--ggtgg------attaaaagtcATGAAACAAA
632518.3.peg.991   attactgcttccttcttgcaaattattttaaaaccagctaaaataaaaatgttcaaaac---tacaaaaaa--ggtgg------attaaaaatcATGAAACAAA
632292.3.peg.1727  ---t-gtactttaagttgcttttgttttaaaatgatgtaaaataaaaaaggtttctc----cctaaataaattcaag---ggtggtaagacatATGAAACAAA
351627.8.peg.1715  aatt-ataaatacacttgctttttcttttaaaaaagtataaaataaaagagtcttatttaaacataaaaaaataaaaattaggtggtaagatcaATGAAACAAA
1222016.3.peg.1854 acttcatattctatcttgcaaattattttaaaaccagctaaaataaaaatgtttaaatc---tataaaaaa-gggtgg------aataaaaatcATGAAACAAA
1121259.3.peg.1106 acttcatattctatcttgcaaattattttaaaaccagctaaaataaaaatgttcaaatc---tataaaaaaagggtgg------aataaaaatcATGAAACAAA
1214564.3.peg.1113 gcctt-gcttgtttcttgcaaattattttaaaaccagctaaaataaaaatgtttaaatc---tataaaaaa-gggtgg------aataaaa-tcATGAAACAAA
1387557.3.peg.1339 aattttacttgtttcttgcagattattttaaaaccagctaaaataaaaatgtttaatac--ttattaaaaa-gggtgg------aatgcagaaaATGAAACAAA
                                  ****    * ********     **********  * *             ** **               *       **********
```

31899.10.peg.1584 **(Athe_1103)** ***abfA*** AraR-binding site **(potential activator);** ArbA-binding sites **(potential repressor)**

31899.10.peg.1584	Caldicellulosiruptor bescii strain DSMZ 6725	632335.8.peg.1212	Caldicellulosiruptor kristjanssonii 177R1B
632516.3.peg.1445	Caldicellulosiruptor lactoaceticus 6A	1214564.3.peg.1111	Caldicellulosiruptor sp. F32
632348.3.peg.1680	Caldicellulosiruptor kronotskyensis 2002		

```
                 arbA<-                                      ArbA-binding site-3
31899.10.peg.1584   CATgattttattccaccctttttt-atagatttgaaCatttttattttagctggttttaaaataatttgcaagaaatgagcaaaagcttatgaa
632516.3.peg.1445   CATgattttattccaccctttttt-atagatttgaacatttttattttagctggttttaaaataatttgcaagaaatgagcaaaagcttatgaa
632335.8.peg.1212   CATgattttattccacccttttttttataggtttgaacatttttattttagctggttttaaaataatttgcaagaaatgagcaaaagcttatgaa
632348.3.peg.1680   CATgattttattccaccctttttt-atagatttaaacatttttattttagctggttttaaaataatttgcaagaaacaagtaaaaacttacgaa
1214564.3.peg.1111  CATgatttt-attccaccctttttt-atagatttaaacatttttattttagctggttttaaaataatttgcaagaaacaagcaag-gcttgtaaa
                    ********* ************** **** *** ******************************************  ** **   ***   **

                                      AraR-binding site
31899.10.peg.1584   cttaaactgagttagcttgtatgtggacaagttttt-attaaaagttttaataacaaacttgatttttatgaatgctatctatataatttaac
632516.3.peg.1445   cttaaactgagttagcttgtctgtggacaagttttt-attaaaagttttaataacaaacttgattttttttgaatgctatctatataatttaac
632335.8.peg.1212   cttaaactgagttagcttgtatgtggacaagttttt-attaaaagttttaataacaaacttgattttttttgaatgctatctatataatttaac
632348.3.peg.1680   tttaaactgagttaacttgtatgtatacaagttttt-attaaaagttttaataaagaacttgacttttttgaatgctgtctatataatctaac
1214564.3.peg.1111  tcaaagctgaga-aacttgtatatatacaagttttttataaaaagttttaacagtaaacttgattatctcaaatatggcttgtataatttaat
                       ** *****  * ***** * *  ********** ** *********** *   ******* * *    ***      * ****** ***
                         ArbA-binding site-2                      ArbA-binding site-1
31899.10.peg.1584   taaaaatattttaattaagaataAactaaaatacgtgtattgagaatttgcttaaaaaggtcttaaaataatttacaaaaggaggaaatatATGAA
632516.3.peg.1445   taaaaatattttaattaagaataaataaaaatatgtgtattgagaatttgcttaaaaaggtcttaaaataatttacaaaaggaggaaatatATGAA
632335.8.peg.1212   taaaaatattttaattaagaataaataaaaatacgtgtattgagaatttgcttaaaaaggtcttaaaataatttacaaaaggaggaaatatATGAA
632348.3.peg.1680   taaa-atattttaattaagattaaataaaaatgtgtatatagaaaatttgcctaaaaaagttttaaaaataat-atcaaaggaggaaatatATGAA
1214564.3.peg.1111  taaa-atattttaattggaattaaactaaa-tacatactttgaaagtatactcaaatgatattagaaaataa----gaaaggaggaaatacATGAA
                    **** ***********   * ****  *** *   *   * ** * * * *  ***      *  ***  *      ************* *****
```

31899.10.peg.2956 (Athe_2376) axeUVW (unknown AxeRS-binding site)

31899.10.peg.2956 Caldicellulosiruptor bescii strain DSMZ 6725
632518.3.peg.2252 Caldicellulosiruptor owensensis OL
632292.3.peg.151 Caldicellulosiruptor hydrothermalis 108
632348.3.peg.165 Caldicellulosiruptor kronotskyensis 2002

```
31899.10.peg.2956 taatcata-ttttactacaattatgaggagggtgaccagtcttttttgattctatgtaaagCttgataacgctcatcttg
632518.3.peg.2252 aagaagtaattttatgatacctatt-gtaaagtaaagggcctttttt-aaatatat-------ttgaccatgtt-attata
632348.3.peg.165  aagtagtaattttatgatacctatt-gtaaagttaagtaccttttttaaatatat-------ttgaccatgtt-attata
632292.3.peg.151  taatcata-ttttactacaattatgaggagggtgaccagtcttttttgattctatgtaaagcttgataacgctcatcttg
                   *    ** *****  * *  ***  * *  ** *     ******  * * ***       ****  * * * **  *

31899.10.peg.2956 agcgtttttgcaaaaggctattaaaacaaaacaaacagtggagggaggaagtcaaaGTGATAAAAAGTAAAA
632518.3.peg.2252 aaatcgcacaatcaaatctattaaaacaaaacaaacattgaagggaggaagtcaaaTTGATTAAAAGTAAAA
632348.3.peg.165  aaattgcacgactaaatctataaaaacaaaacaaaaagtgaagggaggaagttaaaTTGGTAAAAAATAAAA
632292.3.peg.151  agcgtttttgcaaaaggctattaaaacaaaacaaacagtggagggaggaagtcaaaTTGATAAAAAGCAAAA
                  *            **  **** ************* * ** *********** *** ** * ****  ****
```

31899.10.peg.2953 (Athe_2373)*axeRS-abnA*

31899.10.peg.2953 Caldicellulosiruptor bescii strain DSMZ 6725
632518.3.peg.2248 Caldicellulosiruptor owensensis OL
632292.3.peg.155 Caldicellulosiruptor hydrothermalis 108
632348.3.peg.168 Caldicellulosiruptor kronotskyensis 2002

```
31899.10.peg.2953 TAAagtaagatattggtttttgtgaagattatttgaaagatgatataatttaaattAcaatgggtttagtgcaaaaaagggggcagaccctat-ATGT
632292.3.peg.155  TAAagtaagatattggtttttgtgaaggatatttgaatagtgttataattttaattgcaaggtgttttaaa-ataaaagggggcagaccctat-ATGT
632348.3.peg.168  TAAagtaagatattggtttttgtgaaggatatttgaatagtgttataattttaattgaaaggtgttttaaa-ataaaagggggcagaccctgt-ATGT
632518.3.peg.2248 TAAagtaagatattggtttttgtgaaagatatttgaaagatgatataattgaaattataaggggtttagagcagaaaagggggcaaaccctattATGT
                  **************************   ********   ** *******  ****  ** * ****     * *********** ***** * ****
```

31899.10.peg.878 (Athe_0458) bglA

31899.10.peg.878	Caldicellulosiruptor bescii strain DSMZ 6725	351627.8.peg.1224	Caldicellulosiruptor saccharolyticus DSM 8903
632348.3.peg.2291	Caldicellulosiruptor kronotskyensis 2002	632292.3.peg.2316	Caldicellulosiruptor hydrothermalis 108
632516.3.peg.1326	Caldicellulosiruptor lactoaceticus 6A	1222016.3.peg.518	Caldicellulosiruptor changbaiensis strain CBS-Z
632335.8.peg.2389	Caldicellulosiruptor kristjanssonii 177R1B	1214564.3.peg.2420	Caldicellulosiruptor sp. F32
1121259.3.peg.2180	Caldicellulosiruptor acetigenus DSM 7040	608506.3.peg.463	Caldicellulosiruptor obsidiansis OB47
1387557.3.peg.665	Caldicellulosiruptor sp. Wai35.B1	1387569.3.peg.610	Thermoanaerobacter cellulolyticus NA10
632518.3.peg.327	Caldicellulosiruptor owensensis OL	1387555.3.peg.2523	Caldicellulosiruptor sp. Rt8.B8

```
31899.10.peg.878   tggttaaaaacatctaaaaattttgctatattcacttaaaatttttatttgcaaactcaaattttatatggcaatataatttGatatg-
632348.3.peg.2291  tggttaaaaacatctaaaaattttgctatattcacttaaaatttttatttgcaaactcaaattttatatggcaatatattttgatatg-
632292.3.peg.2316  tggtgaaaaacatctaaaaattttgctatattcacttaaaatttttatttgcaaactcaaattttatatggcaatatattttgatatg-
1222016.3.peg.518  tggtgaaaaacatctaaaaattttgctatattcacttaaaatttttatttgcaaactcaaattttatatggcaatatattttgatatg-
1214564.3.peg.2420 tggtgaaaaacatctaaaaattttgctatattcacttaaaatttttatttgcaaactcaaattttatatggcaatatattttgatatg-
1387557.3.peg.665  tagtaaaaaacatctaaaaattttgtaatattcatttaaaatttttatttgcgcattctaattttatatggcagtatattttgatatg-
1387555.3.peg.2523 tagtaaaaaacgtataaaaattttgaaatattcatttaaaatttttatttgcacactccaattttatatggcaatatattttgatatg-
608506.3.peg.463   ttataaaaaacaattaaaaatttcgctataatcatttaaaatttttatttgcaaactcaaatcttatgtggcaatataatttaatatac
351627.8.peg.1224  tggtaaaaaacctctaaaaattctgcaatattcacttaaaatttttatttgcaaactcaaattttatatggcaatatattttgttatg-
1387569.3.peg.610  tggtaaaaaacatctaaaatttttgctatatttatttaaaatttttatttgcaaagtcaaattttatatggcaatatattttgatatg-
632518.3.peg.327   tggtgaaaaacatctaaaaattttgctatattcacttaaaatttttatttgcaaactcaaattttatatggcaatattttttgatatg-
1121259.3.peg.2180 tggtgaaaaacatctaaaaattttgctatatttatttaaaatttttatttgcaaactcaaattttatatggcaatatattttgatatg-
632335.8.peg.2389  tggtgaaaaacatctaaaaatttactatattcacttaaaatttttatttgcaaactcaaattttatatggcaatatattttgatatg-
632516.3.peg.1326  tggtgaaaaacatctaaaaattttgctatatttatttaaaatttttatttgcaaactcaaattttatatggcaatatattttgatatg-
                   *  * ******   ***** **     *** * * *****************  * ** *** ********** ***  ***  ***

31899.10.peg.878   agtgtaattgatttcaaaagaaacaaaagctttaagtc----aaggaggtc-acaa---cATGAGTTTACCAAAAGGATTTCTGTGGGGT
632348.3.peg.2291  agtgtaattgatttcacaactaaaaatttctattactc----aaggaggtttacaaa--cATGAGTTTACCAAAAGGATTTCTGTGGGGT
632292.3.peg.2316  agtgtaattgatttcaaaagaaacaaaagctttatgtc----aaggaggtc-acaa---cATGAGTTTTCCAAAAGGATTTCTGTGGGGT
1222016.3.peg.518  agtgtaattgatttcaaaagaaacaaaagctttaagtc----aaggaggtc-acaa---cATGAGTTTTCCAAAAGGATTTCTGTGGGGT
1214564.3.peg.2420 agtgtaattgatttcaaaagaaacaaaagctttaagtc----aaggaggtc-acaa---cATGAGTTTTCCAAAAGGATTTCTGTGGGGT
1387557.3.peg.665  agtgtaattgatttcataacaaa-aagcttttttgctt----aaggaggtctacaa--atATGAGCTTTCCAAAAGGATTTTTGTGGGGT
1387555.3.peg.2523 agtgtaattgatttcacaacaaa-aag-ttttttgcta----agggaggttttcaa--atATGAGCTTTCCAAAAGGATTTTTGTGGGGT
608506.3.peg.463   aatgtaattgatttcaaaataaa-atgacactaacttt----aaggaggtctattacaaaATGAGTTTTCCAAAAGGATTTCTGTGGGGT
351627.8.peg.1224  agtgtaattgatttcatgacaaaaaaagctttattatccttaaaggaggtttatgg--acATGAGTTTCCCAAAAGGATTTTTGTGGGGT
1387569.3.peg.610  agtgtaattgatttcacgacaaaaaagttttattactt----aaggaggtttacaa--aaATGAGTTTCCCAAAAGGATTTCTGTGGGGT
632518.3.peg.327   agtgtaattgatttcaaaagaaacaaaagctttaagtc----aaggaggtc-acaa---cATGAGTTTTCCAAAAGGATTTTTGTGGGGT
1121259.3.peg.2180 agtgtaattgatttcacaactaaaaagctttattactc----aaggaggtttacaa--acATGAGTTTTCCAAAAGGATTTTTGTGGGGT
632335.8.peg.2389  agtgtaattgatttcacaactaaaaagctttattactc----aaggaggtttacaa--acATGAGTTTTCCAAAAGGATTTTTGTGGGGT
632516.3.peg.1326  agtgtaattgatttcacaactaaaaagctttattactc----aaggaggtttacaa--acATGAGTTTTCCAAAAGGATTTTTGTGGGGT
                   * *************   *  ** **                * ******          ***** ** ************ ********
```

31899.10.peg.880 (Athe_0460) cbp-cbp2 new direct repeat motif for unknown TF (motif 3) (potential activator)

31899.10.peg.880	Caldicellulosiruptor bescii strain DSMZ 6725	1387557.3.peg.667	Caldicellulosiruptor sp. Wai35.B1
632348.3.peg.2289	Caldicellulosiruptor kronotskyensis 2002	1387555.3.peg.2521	Caldicellulosiruptor sp. Rt8.B8
632335.8.peg.2387	Caldicellulosiruptor kristjanssonii 177R1B	1387569.3.peg.612	Thermoanaerobacter cellulolyticus NA10
632516.3.peg.1329	Caldicellulosiruptor lactoaceticus 6A	632292.3.peg.2313	Caldicellulosiruptor hydrothermalis 108
1121259.3.peg.2183	Caldicellulosiruptor acetigenus DSM 7040	1222016.3.peg.521	Caldicellulosiruptor changbaiensis strain CBS-Z
608506.3.peg.465	Caldicellulosiruptor obsidiansis OB47	351627.8.peg.1227	Caldicellulosiruptor saccharolyticus DSM 8903
632518.3.peg.329	Caldicellulosiruptor owensensis OL	1214564.3.peg.2422	Caldicellulosiruptor sp. F32

```
31899.10.peg.880   tagtgcaaaataaaagcaaaataattaaagttggcagtttggcaaaaaaggtataattaagat-Aggcatattatcttattga
608506.3.peg.465   taatgcaaaataaaagcaaaataattaaagtagccaattttatagagaaggtataattaagattaggtttataatagcaaaaa
632518.3.peg.329   taatgcaaaataaaagcaaaataattaaagtagctaattttatagagaaggtataattaagattaggtctataatagcaaaaa
632348.3.peg.2289  tagtgcaaaataaaagcaaaataattaaagttggcagtttggcaaaaaaggtataattaagat-aggcatattatcttattga
632335.8.peg.2387  tagtgcaaaataaaagcaaaataattaaagttggcagtttggcaaaaaaggtataattaagat-aggcatactgtctgattaa
632516.3.peg.1329  tagtgcaaaataaaagcaaaataattaaagttggcagtttggcaaaaaaggtataattaagat-aggcatactgtctgattaa
1121259.3.peg.2183 tagtgcaaaataaaagcaaaataattaaagttggcagtttggcaaaaaaggtataattaagat-aggcatgctgtctgattaa
1387557.3.peg.667  tagtgcaaaattaaagcaaaataattaaagttggcagttcggtaaaaaaggtataattaaggt-aggcttgcggctttataaa
632292.3.peg.2313  tagtgcaaaataaaggcaaaataattaaagtaggcagtttggcaaaaaagatataattaagat-aagttcattggctaacaaa
1222016.3.peg.521  tagtgcaaaataaaggcaaaataattaaagtaggcagtttggcaaaaaagatataattaagat-aagttcattggctaacaaa
351627.8.peg.1227  tagtacaaaattaaggcaaaataattaaaa-gattgattatggtcaaaaggtataattaagatgataggcaaaagtg-aaaat
1214564.3.peg.2422 tagtacaaaattaaggcaaaataattaaaa-gattgattatggtcaaaaggtataattaagatgataggcaaaagtg-aaaat
1387569.3.peg.612  tagtacaaaattaaggcaaaataattaaaa-gattgattatggtcaaaaggtataattaagatgataggcaaaagtg-aaaat
1387555.3.peg.2521 tagtgcaaaattagggcaaaataattaaaa-gattgattgtagacaaaaggtataattttat-ataggcaaaaatgcaaaac
                   ** * ****** *  **************        **      * *** *******    * *

31899.10.peg.880   -atatgtttcg---agaaaggggttt--tcaaaaATGAAGTTTGGCTATTTTGACGATGCTAAAAGA
608506.3.peg.465   -atatgtgctgggtagaaaggagtttt-tgaaaaATGAAGTTTGGATACTTCGATGATGCTAAAAGA
632518.3.peg.329   --tatgtggtgggtagaaaggagtctt-tgtaaaATGAAGTTTGGATATTTCGATGATTCTAAAAGA
632348.3.peg.2289  -atatgtttcg---agaaaggggttt--tcaaaaATGAAGTTTGGCTATTTTGACGATGCTAAAAGA
632335.8.peg.2387  -atatgttttg---agaaaggggttt--tcaaaaATGAAGTTTGGCTATTTTGACGATGCTAAAAGA
632516.3.peg.1329  -atatgttctg---agaaaggggttt--tcaaaaATGAAGTTTGGCTATTTTGACGATGCTAAAAGA
1121259.3.peg.2183 -atatgttctg---agaaaggggttt--tcaaaaATGAAGTTTGGCTATTTTGACGATGCCAAAAGA
1387557.3.peg.667  -tattgttctg---tgaaaggggttt--t-aagaATGAAGTTTGGTTACTTCGATGATGGCAAGAGA
632292.3.peg.2313  gactttctttt---agaaaggagtttaataaaaaATGAAGTTTGGATATTTTGACGATGCCAAAAGA
1222016.3.peg.521  gactttctttt---agaaaggagtttaataaaaaATGAAGTTTGGATATTTTGACGATGCCAAAAGA
351627.8.peg.1227  cataatttgcaaaa-gaagggagactg-ataactTTGAAATTTGGTTATTTTGATGACAATAAAAGA
1214564.3.peg.2422 cataatttgcaaaa-gaagggagactg-ataactTTGAAATTTGGTTATTTTGATGACAATAAAAGA
1387569.3.peg.612  cataatttgcaaaa-gaagggagactg-ataactTTGAAATTTGGTTATTTTGATGACAATAAAAGA
1387555.3.peg.2521 cacagtatgcaaaaagaagggagattg-agagctTTGAAATTTGGCTATTTTGATGACCAGAAAAGA
                                  *** ****  *         **** ***** ** ** ** **    ** ***
```

31899.10.peg.541 (Athe_0146) *fruRKAB-hpr-ptsI* FruR-binding site (repressor)

31899.10.peg.541	Caldicellulosiruptor bescii strain DSMZ 6725
632348.3.peg.2553	Caldicellulosiruptor kronotskyensis 2002
1387557.3.peg.413	Caldicellulosiruptor sp. Wai35.B1
632292.3.peg.2599	Caldicellulosiruptor hydrothermalis 108
632516.3.peg.2570	Caldicellulosiruptor lactoaceticus 6A
1121259.3.peg.556	Caldicellulosiruptor acetigenus DSM 7040
632335.8.peg.171	Caldicellulosiruptor kristjanssonii 177R1B
632518.3.peg.93	Caldicellulosiruptor owensensis OL
608506.3.peg.157	Caldicellulosiruptor obsidiansis OB47
1214564.3.peg.1796	Caldicellulosiruptor sp. F32
1222016.3.peg.2788	Caldicellulosiruptor changbaiensis strain CBS-Z
351627.8.peg.2634	Caldicellulosiruptor saccharolyticus DSM 8903
1387555.3.peg.481	Caldicellulosiruptor sp. Rt8.B8

```
31899.10.peg.541   tgcaaatcaaacaaatttagacaaga--aggcatatattttttgtaaaaggtgcttgacagggtagctttcatgttctataatgaa
632348.3.peg.2553  tgcaaatcaaacaaatttagacaaga--aggcatatattttttgtaaaaggtgcttgacagggcagctttcatgttctataatgaa
632292.3.peg.2599  tgcaaatcaaacaaatttagacaaga--aggcatatattttttgtaaaaggtgcttgacagggcagctttcatgttctataatgaa
1387557.3.peg.413  tgcaaatcaaacaaatttgaaccaaa--aggaatatattttttgtaaaaggtgcttgacagggtagctttcatattctataatgaa
1121259.3.peg.556  tgcaaatcaaacaaatttagacaaga--aggcatatattttttgtaaaaggtgcttgacagggcagctttcatgttctataatgaa
632335.8.peg.171   tgcaaatcaaacaaatttgaacaaga--aggcatatattttttgtaaaaggtgcttgacagggcagctttcatgttctataatgaa
632516.3.peg.2570  tgcaaatcaaacaaatttagacaaga--aggcatatattttttgtaaaaggtgcttgacagggcagctttcatattctataatgga
632518.3.peg.93    tgcaaatcaaacaaatttgaacaaga--aggtatatattttttgtaaaaggtgcttgacagggcagcattcatgttctataatgaa
608506.3.peg.157   tgcaaatcaaacaaatttaaacaaga--aggcatatattttttgtaaaaggtgcttgacagggtagttttcatgttttataatgaa
1214564.3.peg.1796 ttcaaattttacgtttctctatgtagtctgccaaaaatttttgtttaaaatagcttgacaagaaaactttcatatcctataataaa
351627.8.peg.2634  ttcaaattttacgtttctctatgtagtctgccaaaaatttttgtttaaaatagcttgacaagaaaactttcatatcctataataaa
1222016.3.peg.2788 tttgagtttcaaactg----acaaagtcaag-aaaaatttttgtttaaaatagcttgacaagaaaactttcatatcctataataaa
1387555.3.peg.481  tttaaatataacaaactttgattttatttaa-aaacatacattattgaaata--ttgacaattttaaaaaaatgtcatataataaa
                   *   * *   *         *           * * **   *  *  **     ******           ** *  ******  *

31899.10.peg.541   attAatcataaatgagcataaatgattaaattcattcatta-aaatgagcataaacgaaag---gtgaataaaATGTTTGCAGAGGAA
632348.3.peg.2553  attaatcataaatgagcataaatgattaaattcattcatca-aaatgagcataaacgaaag---gtgaataaaATGTTTGCAGAGGAA
632292.3.peg.2599  attaatcataaatgagcataaatgattaaattcattcacca-aaatgagcacaaacgaaag---gtgaataaaATGTTTGCAGAGGAA
1387557.3.peg.413  attaatcataaatgagcataaatgattaaattcattcacca-aaatgagcataaacgaaag---gtgaacaaaATGTTTGCAGAGGAA
1121259.3.peg.556  attaatcataaatgagcataaatgattaaattcattcatcg-aaatgagcacaaacgaaag---gtgaataaaATGTTTGCAGAGGAA
632335.8.peg.171   attaatcataaatgagcataaatgattaaattcattcatcg-aaatgagcacaaacgaaag---gtgaataaaATGTTTGCAGAGGAA
632516.3.peg.2570  attaatcataaatgagcataaatgattaaattcattcatcg-aaatgagcacaaacgaaag---gtgaataaaATGTTTGCAGAGGAA
632518.3.peg.93    attaatcataaatgagcataaataattaaattcattcatca-aaatgagcacaaacgaaag---gtgaataaaATGTTTGCAGAGGAA
608506.3.peg.157   attaatcgcaaatgagcataaatgattaaattcattcatca-aaatgagcacaaacgaaag---gtgaataaaATGTTTGCAGAGGAA
1214564.3.peg.1796 atcaatcacaaatgagcatattaagtcacaaataatcataaaaaatgagcataattgagaa-aaggtgatactATGTTTGCAGAAGAG
351627.8.peg.2634  atcaatcacaaatgagcatattaagtcacaaataatcataaaaaatgagcataattgagaa-aaggtgatactATGTTTGCAGAAGAG
1222016.3.peg.2788 atcaatcacaaatgagcatattaagtcacaaataatcataaaaaatgagcataattgagaa-aaggtgatactATGTTTGCAGAAGAG
1387555.3.peg.481  atcaatcataaatgagcatataaaatcataaacgagca-gataagtgagcataaatgagagtaaggtgatgggATGTTTGCAGAGGAG
                   ** ****  ***********     * * *      **    ** ****** **  ** *    *   *    *********** **
```

31899.10.peg.2673 (Athe_2118) scrK

31899.10.peg.2673 Caldicellulosiruptor bescii strain DSMZ 6725
632516.3.peg.2459 Caldicellulosiruptor lactoaceticus 6A
608506.3.peg.2030 Caldicellulosiruptor obsidiansis OB47
1387555.3.peg.940 Caldicellulosiruptor sp. Rt8.B8
1387555.3.peg.2205 Caldicellulosiruptor sp. Rt8.B8
1222016.3.peg.795 Caldicellulosiruptor changbaiensis strain CBS-Z
351627.8.peg.1460 Caldicellulosiruptor saccharolyticus DSM 8903
632518.3.peg.1929 Caldicellulosiruptor owensensis OL
632335.8.peg.520 Caldicellulosiruptor kristjanssonii 177R1B
1121259.3.peg.1951 Caldicellulosiruptor acetigenus DSM 7040
632348.3.peg.545 Caldicellulosiruptor kronotskyensis 2002
1214564.3.peg.122 Caldicellulosiruptor sp. F32
1387557.3.peg.2531 Caldicellulosiruptor sp. Wai35.B1
632292.3.peg.690 Caldicellulosiruptor hydrothermalis 108

```
31899.10.peg.2673  -gagtatattaaaaggtttctcacaaggtttatttatccctctatatcttattcacttgtgaatattaaatgttctataata
1222016.3.peg.795  agagtatattaaaaggcttctcacaaggtttatttatcc-tctatattttcttcacttatgaacactaaatgttctataata
351627.8.peg.1460  agagtatattaaaaggcttctcacaaggtttatttatcc-tctatattttcttcacttatgaacactaaatgttctataata
1387555.3.peg.940  -gagtatattaaaaggtttctcacaaggtttatttatccctctatatcttattcacttatgaacactaaatgttctataata
1214564.3.peg.122  -gagtatattaaaaggcttctcacaaggtttatttatccctctatatcttcttcacttctgaatattaaatgttctataata
1387555.3.peg.2205 -gagtatattaaaaggtttctcacaaggtttatttatccctctatatcttattcacttgtgaatattaaatgttctataata
632516.3.peg.2459  -gagtatattaaaaggtttctcacaaggtttatttatccctctatatcttattcacttgtgaatattaaatgttctataata
608506.3.peg.2030  -gagtatattaaaaggtttctcacaaggtttatttatccctctatatcttcttcacttctgaatattaaatgttctataata
632518.3.peg.1929  tcaaaagaccaaagtacaggttaaagggtt---ttatct---tat-tcttcttcacttctgaatattaaatgttctataata
632348.3.peg.545   tcaaaagaccaaagtacatgtcaaagagtt---ttatct-------ttttcttcacttctgaatattaaatgttctataata
1121259.3.peg.1951 tctaaagagtagatgatatattata---tt---ttaccc---tat-tcttcttcacttctgaatattaaatgttctataata
632292.3.peg.690   tctaaa-agtagatgatatat-ata---tt---ttaccc---tat-tcttcttcacttctgaatattaaatgttctataata
632335.8.peg.520   tctaaagagtagatgatatattata---tt---ttaccc---tat-tcttcttcacttctgaatatcaaatgttctataata
1387557.3.peg.2531 catatttaataagttatat-taata---tt---ctacct---tat-t-ttcttcactttcgagaagcaaatgttttataata
                          *  *         * * *   **    ** *        * ** *******  **  *  ******* *******

31899.10.peg.2673  atgttaT----actcaaagaaaaggggctattggtttTTGAAAGTTGTCTGCTACGG
1222016.3.peg.795  atgttat-----actcaaagaaaaggggctactagtttTTGAAAGTTGTCTGCTACGG
351627.8.peg.1460  atgttat-----actcaaagaaaaggggctactagtttTTGAAAGTTGTCTGCTACGG
1387555.3.peg.940  atgttat-----actcaaagaaaaggggctactagtttTTGAAAGTTGTCTGCTACGG
1214564.3.peg.122  atgttat-----actcaaagaaaaggggctactagtttTTGAAAGTTGTCTGCTACGG
1387555.3.peg.2205 atgttat-----actcaaagaaaaggggctactagtttTTGAAAGTTGTCTGCTACGG
632516.3.peg.2459  atgttat-----actcaaagaaaaggggctattggtttTTGAAAGTTGTCTGCTACGG
608506.3.peg.2030  atgttat-----actcaaagaaaaggggctattggtttTTGAAAGTTGTCTGCTACGG
632518.3.peg.1929  atgttat-acttaa---gaataaaggggctattggtttTTGAAAGTTGTCTGCTACGG
632348.3.peg.545   ttgttat-acttaataagaataaaggggctactaatttTTGAAAGTTGTCTGCTACGG
1121259.3.peg.1951 atgttat-----acccgaa-aaaaggaggttattggtctTTGAAAGTTGTCTGCTATGG
632292.3.peg.690   atgttat-----actcgaa-aaaaggaggttgttggtttTTGAAGGTTGTTTGCTACGG
632335.8.peg.520   atgttat-----actcaaagaaaaggggctactagtttTTGAAAGTTGTCTGCTACGG
1387557.3.peg.2531 atgttatcatttaattaacataagggaggaatctacaatTTGAAAATTGTCTGCTTTGG
                    ******     *       **  ** **   *    ******  **** ****  **
```

31899.10.peg.968 (Athe_0537) mtlD

31899.10.peg.968	Caldicellulosiruptor bescii strain DSMZ 6725	632516.3.peg.2347	Caldicellulosiruptor lactoaceticus 6A
632348.3.peg.2210	Caldicellulosiruptor kronotskyensis 2002	1387557.3.peg.782	Caldicellulosiruptor sp. Wai35.B1
632292.3.peg.2218	Caldicellulosiruptor hydrothermalis 108	1222016.3.peg.2362	Caldicellulosiruptor changbaiensis strain CBS-Z
632518.3.peg.439	Caldicellulosiruptor owensensis OL	1214564.3.peg.1586	Caldicellulosiruptor sp. F32
608506.3.peg.541	Caldicellulosiruptor obsidiansis OB47	351627.8.peg.871	Caldicellulosiruptor saccharolyticus DSM 8903
632335.8.peg.605	Caldicellulosiruptor kristjanssonii 177R1B	1387569.3.peg.684	Thermoanaerobacter cellulolyticus NA10
1121259.3.peg.1648	Caldicellulosiruptor acetigenus DSM 7040	1387555.3.peg.2424	Caldicellulosiruptor sp. Rt8.B8

```
31899.10.peg.968   tgaagtagctttcaagttagtgataaactttata-acAgaaaatcaaaaa-cttcatcaaagagggtttaaaaaagacaATGAAAGCAGC
632335.8.peg.605   TGAagtgacctgcaaattagtgataaactttata-gtagaaaactaaaaa-cttcatcaaagagggtttaaaagaaaaaATGAAGGCAGC
1121259.3.peg.1648 tgaagtgacctgcaaattagtgataaactttata-gtagaaaactaaaaa-cttcatcaaagagggtttaaaagaaaaaATGAAGGCAGC
632348.3.peg.2210  tgaagtagctttcaagttagtgataaactttata-atagaaaatcaaaaa-cttcatcaaagagggtttaaaaaagacaATGAAAGCAGC
632292.3.peg.2218  tgaagtagctttcaagttagtgataaactttata-atagaaaatcaaaaa-cttcatcaaagagggtttaaaaaagacaATGAAAGCAGC
632518.3.peg.439   tgaagtgattttcaagtaagtagtaaactttata-atagaaaatcaaaag-ctttatcaaagagggt----aaaaaacaATGAAAGCAGC
608506.3.peg.541   tgaagtgattttcaagtaagtagtaaactttatatatagaaaatcaaaaa-ctttatcaaagagggtttaaaaaagacaATGAAAGCAGC
1387557.3.peg.782  tgaagtagctataaggttagtgataaactttata-ataaaaaatcaaaaaacttcagcagagagggtttaaaaaagacaATGAAAGCAGC
1214564.3.peg.1586 tgaaaaatcttgcttggtagtggtaaactgaata-ttagatga----aattttct-ttagagaaggattagaataaa-aGTGAGAGCGGC
351627.8.peg.871   tgaaaaatcttgcttggtagtggtaaactgaata-ttagatga----aattttct-ttagagaaggattagaataaa-aGTGAGAGCGGC
1222016.3.peg.2362 tgaaaaatcttgcttggtagtggtaaactgaata-ttagatga----aattttct-ttagagaaggattagaataaa-aGTGAGAGCGGC
1387569.3.peg.684  tgaaaaattagagtaaaaagtgttaaactctaaa-atagaaaa----agttctttactcaaaaaagaagggtaaaaa-aGTGAAGGCAGC
1387555.3.peg.2424 tgaaaaattggggtaaaaagtgttaaactcaaag-atagaaaa----tat-ccctggttgaaaaagaagggtgaaaa-aGTGAAGGCAGC
632516.3.peg.2347  tgaagtgacctgcaaattagtgataaactttata-gtagaaaactaaaaa-cttcatcaaagagggtttaaaagaaaaaATGAAGGCAGC
                   ****              ***  ******        ***  *                 * *  *        *   * ***  ** **
```

31899.10.peg.1156 (Athe_0702-0706) Athe_0702-0705-rhaA RhaR2-binding site **(potential repressor)**

31899.10.peg.1156	Caldicellulosiruptor bescii strain DSMZ 6725	1121259.3.peg.915	Caldicellulosiruptor acetigenus DSM 7040
632292.3.peg.2057	Caldicellulosiruptor hydrothermalis 108	1387557.3.peg.970	Caldicellulosiruptor sp. Wai35.B1
632348.3.peg.2031	Caldicellulosiruptor kronotskyensis 2002	608506.3.peg.722	Caldicellulosiruptor obsidiansis OB47
632518.3.peg.620	Caldicellulosiruptor owensensis OL	351627.8.peg.990	Caldicellulosiruptor saccharolyticus DSM 8903

```
31899.10.peg.1156 atcaaaataacattgacaaaaaaacaaacaaaatgatactatta-aattggagatgaaaaaagttaacatgatagatgaaaaa
521460.8.peg.751  atcaaaataacattgacaaaaaaacaaacaaaatgatactatta-aattggagatgaaaaaagttaacatgatagatgaaaaa
632348.3.peg.2031 atcaaaataatattgacaaaaaaacaaacaaaatgatactatta-aattggagatgaaaaaagttaacatgatagatgaaaaa
632518.3.peg.620  atcaaaacaacattgacaagaaa-caaacaaaatgatactatta-aattggagatgaaaaaagttaacatgatagatgaaaaa
608506.3.peg.722  atcaaaacaacattgacaagaaa-caaacaaaatgatactatta-aattggagatgaaaaaagttaacatgatagatgaaaaa
1121259.3.peg.915 atcaaaataacattgacagaaaa-caaacaaaatgatactattg-aattgaagatgaaaaaagttaacatgatggatgaaaaa
632292.3.peg.2057 atcaaaataacattgacaaaaaa-caaacaaaatgatactatta-aattggagatgaaaaaagttaacatgatagatgaaaaa
1387557.3.peg.970 gtcaaaataacattgacaaaaaa-caaacaaaatgatactatta-aattggaaatgaacaaaattaacataataaatgaaaaa
351627.8.peg.990  acacaaataatattgacataaaa-caaacaaaatgatattattttgaataaagatgaaataatttttcatca-taatgaaaaa
                      *** ** *******  *** ************** ****   * *  * *****  ** **  *** **  ********

31899.10.peg.1156 atatatcaaacatattgaaataaataaacagctaaaatggaggtttttgagATGGCAACACCTGT
521460.8.peg.751  atatatcaaacatattgaaataaataaacagctaaaatggaggtttttgagATGGCAACACCTGT
632348.3.peg.2031 atatatcaaacatattgaaataaataaacagctaaaatggaggtcatt-gatATGGCAACACCTGT
632518.3.peg.620  gtatatcaaatctattgaaacaaataaacaggtcaaatggaggtcatt-aatATGGCAACACCTGT
608506.3.peg.722  atatatcaaatctattgaaacaaataaacaggtcaaatggaggtcatt-aatATGGCAACACCTGT
1121259.3.peg.915 atatatcaaatctattgaagcaaataaacaggtcaaatggaggtcatt-aatATGGCAATGCCTGT
632292.3.peg.2057 atatatcaaacatattgaaacaaacaaacagctcaaatggaggttttt-gatATGGCGGTGCCTGT
1387557.3.peg.970 acatatcaaatcttttgaaataaataaacagataaaatggaggttttt-gatATGGCGGTACCTGT
351627.8.peg.990  atatttcaaataatgtgttaccaactaacaattttc-cggagggtttc-aaaATGGCAACACCTGT
                    ** *****     **     **  ****  *     *****   *   * *****   *****
```

31899.10.peg.1301 (Athe_0841) rhaB-rhaR RhaR-binding site (potential repressor)

31899.10.peg.1301	Caldicellulosiruptor bescii strain DSMZ 6725	1387557.3.peg.1113	Caldicellulosiruptor sp. Wai35.B1
632348.3.peg.1910	Caldicellulosiruptor kronotskyensis 2002	1222016.3.peg.2133	Caldicellulosiruptor changbaiensis strain CBS-Z
632292.3.peg.1934	Caldicellulosiruptor hydrothermalis 108	351627.8.peg.1118	Caldicellulosiruptor saccharolyticus DSM 8903
632518.3.peg.751	Caldicellulosiruptor owensensis OL	1214564.3.peg.2099	Caldicellulosiruptor sp. F32
608506.3.peg.852	Caldicellulosiruptor obsidiansis OB47		

```
31899.10.peg.1301  aaaatagcaaacaaaactTAAatgtgatataataaaaacaaacacaaaataacc-caaacagaataaggaggacaaaaATGAAAAGTA
632348.3.peg.1910  aaaatagcaaacaaaacttaaatgtgatataataaaaacaaacacaaaataact-caaacagaataaggagga-aaaaATGAAAAGTA
632292.3.peg.1934  aaaatagcaaacaagactcaaatatgatataataaaaacaaacataaaataact-aaaacagaataaggaggacaaaaATGAAGAGTA
632518.3.peg.751   aaaatagcaaacaaaattcaaatatgatataataaaaacagacacaaaataacc-aaaacagaataaggaggacaaaaATGAAAAGTA
608506.3.peg.852   aaaatagcaaacaaaacttaaatgtgatataataaaaacaaacacaaaataacc-aaaacaaaataaggaggacaaaaATGAAAAGTA
1387557.3.peg.1113 aaaatagcaaacaaaactcaaatgtgatataataaaaacaaacacaaaataacc-aaaacagaataaggaggacaaaaATGAAGAGTA
1222016.3.peg.2133 aagatagcaaacaaaaattaaatatgatataataaaaacaaacataaaagaagtaaaaacaaatgg-ggagga-aaaaATGAAAACAA
1214564.3.peg.2099 aagatagcaaacaaaaattaaatatgatataataaaaacaaacataaaagaagtaaaaacaaatgg-ggagga-aaaaATGAAAACAA
351627.8.peg.1118  aagatagcaaacaaaaattaaatatgatataataaaaacaaacataaaaaaagtaaaaacaaatg--ggagga-aaaaATGAAAACAA
                   ** *********** * * **** **************** *** **** **    ***** *    ****** ********* *  *
```

31899.10.peg.2630 (Athe_2081) rhaYZX

31899.10.peg.2630	Caldicellulosiruptor bescii strain DSMZ 6725	1387557.3.peg.2499	Caldicellulosiruptor sp. Wai35.B1
632348.3.peg.589	Caldicellulosiruptor kronotskyensis 2002	1222016.3.peg.834	Caldicellulosiruptor changbaiensis strain CBS-Z

```
31899.10.peg.2630  aatacagaagtaaatttaaatccataaaacagcaaataaaacagat--tcaatttaaa-caaatataaaataaaagcagaaagtaaggaatatta
1387557.3.peg.2499 tagtaatggtaaatacaagatttaaaagatattttataaatttggtagtcgaatcaaa--aaatgtaaattgagaa-agacaaaat--attttct
1222016.3.peg.834  taattaaagtaaatacaaactcaataagtcaaatttcaataaaaattaaaaaattcga--agatatgaatagagaa-agataaaacacattttct
632348.3.peg.589   aatacagaataaaatttaaatccataaaacagcaaataaaacagat--tcaatttaaacaaatagaaaataaaagcagaaagtaaggaatatta
                    *   *     **    *  *  * **   *      **      *     * *   *  * **   **   * *  *** *  *   * * *

31899.10.peg.2630  tcttttatccttgcaaCaagtgaaggagggtataaatgctcaatagagcatttaattttaaaaaaaaaggaaggaggaaattt-tATGAAAAGAATA
1387557.3.peg.2499 tcttttgga-gtgttatg-gcaaaaatgctcttaaaagagcaattgtaaatattactacaaaaaacataaagggagggaagttcaGTGAAAAAGTTT
1222016.3.peg.834  tcttttaaa-ttgtaatg-gtaaaagtgctctttaaagagcaattgtaaatattaatacaaaaaatataaaggagggaagttcaGTGAAAAAGTTT
632348.3.peg.589   tcttttatccttgcaacaagtgaaggagggtataaatgctcaatagagcatttaattttaaaaaa--aggaaggaggaaatttatATGAAAAGAATA
                   ******     **  *   *  **   *    * ** *  **** *   ** * * *  ******     ******* ** **   ******   *
```

31899.10.peg.1149 (Athe_0695) fucA-rhaD1 RhaR-binding site (repressor)

31899.10.peg.1149	Caldicellulosiruptor bescii strain DSMZ 6725
632348.3.peg.2038	Caldicellulosiruptor kronotskyensis 2002
632518.3.peg.613	Caldicellulosiruptor owensensis OL
632292.3.peg.2065	Caldicellulosiruptor hydrothermalis 108
1121259.3.pg.907	Caldicellulosiruptor acetigenus DSM 7040
608506.3.peg.713	Caldicellulosiruptor obsidiansis OB47
1387557.3.peg.963	Caldicellulosiruptor sp. Wai35.B1

```
31899.10.peg.1149 ttttaaataaatcatagtgacaaaatacctgagttatattataataattaaTaaaagacatcaaaatcaaatacaaaaacacataaatacaaaactattt
632348.3.peg.2038 ttttaaataaatcatagtgacaaaatacctgagttatattataataattaataaaagacatcaaaatcaaatacaaaaacacataaatacaaaactattt
632292.3.peg.2065 ttttaaataaatcatagtgacaaaatatctgagttgtattataataattaataaaagacatcaaaatcaaatataaaaacacataaaaacaaaactattt
1121259.3.peg.907 ttttaaataaacgatagtgacaaaatatctgagttgtattataataattaataaaagacatcaaaatcaaatataaaaacacataaaaacaaaactaatt
1387557.3.peg.963 ttttaaataaattatagtgacaaaatatctgagttatattataataattaataaaaggcatcaaaatcaaatacaaaatcacataaaaacaaaactattt
608506.3.peg.713  ttttagataaatgatagtgacaaaatatctgagttgtattataataattaataaaagacatcaaaatcaaatataaaaacacataaaagcaaaactattt
632518.3.peg.613  ttttagataaatgatagtgacaaaatatctgagttgtattataataattaataaaagacatcaaaatcaaatacaaaaacacataaatacaaaactattt
                  ***** *****  ************** ******* ********************* *************** **** ********  ******** **

31899.10.peg.1149 aactttagctttaaaaa-tcaagcataattaaacataaaatcaaaa-ttgcacaaatatcggttgtagaaaggagcataaattATGAAATTTGAACTACT
632348.3.peg.2038 aactttagctttaaaaa-tcaagcataattaaacataaaatcaaaaattgcacaaatatcggttgtagaaaggagcataaattATGAAATTTGAACTACT
632292.3.peg.2065 aacttcagcttcaaaaa-tcaagcacaattaaacataaaaacaaaa-ttgcacaaatatcagttgtggagaggagcgtaaattATGAATTTTGAACTACT
1121259.3.peg.907 aacttcagcttcaaaaa-tcaggcacaattaaacataaaagcaaaa-ttgcacaaatatcagttgaggaaaggagcataaattATGAATTTTGAACTACT
1387557.3.peg.963 aacttcagcttcaaaaa-tcaagcacaattaaacataaaagcaaaa-ttgcacgaatattaattgtgggaaggggcataaattATGAATTTTGAACTACT
608506.3.peg.713  aactttgacttaaaaaa-tcaagcacgagtaaacataaatacaaaa-ctgcacaaatattagttgtagagaggggcacaaattATGAAATTTGAACTACT
632518.3.peg.613  aactttagctttaaaaaatcaagcacaagtaaacataaatgcaaaa-ttgcacaaatatcagttgtagagaggggcataaattATGAAATTTGAACTACT
                  *****   *** ***** *** ***  * **********  *****  ***** *****   ***  *  *** **  ********** ***********
```

31899.10.peg.2619 (Athe_2073) fucI-alfA FucR-binding sites (repressor)

31899.10.peg.2619	Caldicellulosiruptor bescii strain DSMZ 6725	351627.8.peg.1488	Caldicellulosiruptor saccharolyticus DSM 8903
608506.3.peg.1973	Caldicellulosiruptor obsidiansis OB47	1214564.3.peg.994	Caldicellulosiruptor sp. F32
632518.3.peg.1864	Caldicellulosiruptor owensensis OL	1387569.3.peg.1853	Thermoanaerobacter cellulolyticus NA10
1222016.3.peg.846	Caldicellulosiruptor changbaiensis strain CBS-Z		

```
31899.10.peg.2619  tctgattgacaagaaatgagccaaagaatataata----aaatAaaagtcaaacttttaatttatcgtat--aatgtaaaacgtttagcatatagtga
1222016.3.peg.846  tgttattgacaaggaatgtatcaaagaatataata----aaatcaaaagtaaagttttcattttgtcatgg--tttgtgaaacgtttagcatatggtga
1387569.3.peg.1853 tgttattgacaaggaatgtatcaaagaatataata----aaatcaaaaataaaattttcattttgtcatgg--tttgtgaaacgtttagcatatggtga
351627.8.peg.1488  tgttattgacaaggaatgtatcaaagaatataata----aaatcaaaaatagaattttattttgtcgtgc--gatgtgaaacgtttagcatatggtga
1214564.3.peg.994  tgttattgacaaggaatgtatcaaagaatataata----aaatcaaaaatagaattttattttgtcatgc--aatgtgaaacgtttagcatatggtga
608506.3.peg.1973  tcttttattcttttctttagcttgtattccagtatcctacacaagaggttaaaaattgagtccgctgtataattagtaaaataatcatcaaaaggtgt
632518.3.peg.1864  ggataggatttagttctacaccaaaatggaaaatatctcatgccactccttgtagtttttgatttttttgaataggagactacatttttttaattatat
                                   *    *         * **    *    *           **          *       *  *     *      *   *

31899.10.peg.2619  gagaatttggtgataaattacctttccacttttatagtaaatagaaaaatatgatacacgattgtgactaactgacaattatataaaagtttata-gc
1222016.3.peg.846  gagagttttagtgataa-ttgactctctatttttatagtaaatagaaaaatgcgatatacaattaggactaacggacaattatataaaagcttggg-gc
1387569.3.peg.1853 gagagttttagtgataa-ttaactttctatttttatagtaaatagaaaattgcgatatacaattaggactaacggacaattatataaaagcttggt-gc
351627.8.peg.1488  gagagttttaatgatga-ttgactctctatttttagagtaaatagaaaaatgtgatatacaattgtgactaaaggacaattatgtaaaagtttata-gc
1214564.3.peg.994  gagagttttaatgatga-ttgactctctatttttagagtaaatagaaaaatgtgatatacaattgtgactaaaggacaattatgtaaaagtttata-gc
608506.3.peg.1973  tcacgttt-gtcaagaaaatgag-----atttttgagactactaaaaaatattgctattaaacaagtactaa------aatatatattgttacaag-ga
632518.3.peg.1864  tcatgttaggatggaggcagcctcaattctttttatgagtttcttctcactattttcgcacatttttttcgctgtggtaaatatctctacacctattcga
                        **                       ****              *      *     *      *         * *** *           *

31899.10.peg.2619  -aaaaaattaggcaaataattgacaagttttaaaaaaagagtgtatac----------------taaaaacagaataaatgatattata-attatttt
1222016.3.peg.846  -aaaaaattagttaaata--ttgacaagttgtaaaatag--catatatac---------------ttaaagacagaatgaatagcgttata-attatttt
1387569.3.peg.1853 -aaaaaattagttaaata--ttgacaagttgtaaaatag--catatatacaatagcatatatactttaaagacagaatgaatagcgttata-attatttt
351627.8.peg.1488  gaaaaaattaggcaaatatattgacaagttgtgaaaaag--agtgtatac----------------tagaaacagaataaatggcgttata-attatttt
1214564.3.peg.994  gaaaaaattaggcaaatatattgacaagttgtgaaaaag--agtgtatac----------------tagaaacagaataaatggcgttata-attatttt
608506.3.peg.1973  tagaaattcgattcactatattgacaaattgtgaaaaat--agtatatac----------------taagttcaaaaatagtgttgatatatattttttt
632518.3.peg.1864  gggaaattcaattaactatattgacaaattgtgaaaaat--agtatatat----------------taaccttaaaaatagtgtaaatgtatact-tttt
                      *** *      * **  ******* ** * *** *   * ****                 **     * **  * *     * ** * * ****

31899.10.peg.2619  tgcaaaacaaaagctaaacgtttcacaatctttcattccatttcaccaatgtagttttgc-atctcaaaaattgaaagaaaggagaaatagcaaaaATG
1222016.3.peg.846  tctaaaacaaaagctaaacgtttcacatactttcattccatttcaccaatgtagttttgc-atctcaaaaactgaaagaaaggagaaatagcaaaaATG
1387569.3.peg.1853 tctaaaacaaaagctaaacgtttcatatactttccttccattttgccaatatagttttgg-atctcaaaaactgaaagaaaggagaaatagcaaaaATG
351627.8.peg.1488  tgtaaaacaaaagctaaacgtttcacatactttcattccatttcaccaatgtagttttgc-atctcaaaaactgaaagaaaggagaaatagtaaaaATG
1214564.3.peg.994  tgtaaaacaaaagctaaacgtttcacatactttcattccatttcaccaatgtagttttgc-atctcaaaaactgaaagaaaggagaaatagcaaaaATG
608506.3.peg.1973  tgtaaaataaaagctaaacgtttcataatattatttctcacttatatatagtagtttttttatcttcagaattgcagaaaaggagtaaaattaaaaATG
632518.3.peg.1864  tgcaaaacaaaaactaaacgtttcataatattatttcttacttgtatatagtagtctttt-atcttcagaattacaaaaaaggagtaatattaaaaATG
                   *  **** **** ************ *   **   *   * **    *   **** **   ****  * ** *  *  ******* ** *  *******
```

31899.10.peg.2621 (Athe_2074) fucR

31899.10.peg.2621	Caldicellulosiruptor bescii strain DSMZ 6725
351627.8.peg.1487	Caldicellulosiruptor saccharolyticus DSM 8903
1214564.3.peg.993	Caldicellulosiruptor sp. F32
1222016.3.peg.844	Caldicellulosiruptor changbaiensis strain CBS-Z
1387569.3.peg.1859	Thermoanaerobacter cellulolyticus NA10
632292.3.peg.734	Caldicellulosiruptor hydrothermalis 108
1121259.3.peg.2454	Caldicellulosiruptor acetigenus DSM 7040
632348.3.peg.595	Caldicellulosiruptor kronotskyensis 2002
608506.3.peg.1986	Caldicellulosiruptor obsidiansis OB47
632518.3.peg.1881	Caldicellulosiruptor owensensis OL
1387557.3.peg.2480	Caldicellulosiruptor sp. Wai35.B1

```
31899.10.peg.2621  gctttctaaaattcttgatttaagagagtttaggatgataaa-ataaActaataa--aaggttattataca----aaagaggGTG
632292.3.peg.734   acatgctaaaactcttgattgaaaaggctttaaggtgataaa-ataaactaaaaataaaaattactgtaca----aaagaggGTG
1222016.3.peg.844  gcttcccaaaattcttgatttaagagagcttagggtggtaaa-ataaactaataa-aaaattcattgtaca----aaagaggGTG
351627.8.peg.1487  atgttgtaaaattgttgatttaaaaaactttatagtgataaa-ataa--tagcaa----aatagttaccca----aaagaggGTG
1214564.3.peg.993  atgttgtaaaattgttgatttaaaaaactttatagtgataaa-ataa--tagcaa----aatagttaccca----aaagaggGTG
1387569.3.peg.1859 atgttgtaaaattgttgatttaaaaaactttatagtgataaa-ataa--tagcaa----aatagttaccca----aaagaagGTG
1121259.3.peg.2454 tcattattgaa---aagagtaaagacaagatggtaaaataaacaataaagaatatggccgaaaattcagta---cgagagggGTG
1387557.3.peg.2480 acggctttaagtataagtttaatgtcaattgaaaagaacaaagagaaaatgataaaataaatatcaaaataaaacaaagaagGTG
608506.3.peg.1986  taattattgaa---gtgaataaatacaaagtgataaaataaacaataaaggagctttagaaaagttcaaac---tgagaggaGTG
632518.3.peg.1881  tgattattgaa---gtgaataaagacaaagtgataaaataaacaataaaggagttttagaaaagttcaaac---tgagaggaGTG
632348.3.peg.595   ttattattgaa---aagaataaagacaagatggtaaaataaacaataaagaa-tattgccaaaattcagta---cgagagg-GTG
                            *      *  * *                 *** *  *                             *     ***
```

31899.10.peg.2969 (Athe_2387) *kdgR-kdgAK-kduID-rhiN-pgl* KdgR-binding site (repressor)

31899.10.peg.2969	Caldicellulosiruptor bescii strain DSMZ 6725	1222016.3.peg.371	Caldicellulosiruptor changbaiensis strain CBS-Z
632348.3.peg.152	Caldicellulosiruptor kronotskyensis 2002	1387557.3.peg.2819	Caldicellulosiruptor sp. Wai35.B1
632292.3.peg.140	Caldicellulosiruptor hydrothermalis 108	632518.3.peg.2273	Caldicellulosiruptor owensensis OL
1214564.3.peg.1491	Caldicellulosiruptor sp. F32	1121259.3.peg.1423	Caldicellulosiruptor acetigenus DSM 7040
608506.3.peg.2316	Caldicellulosiruptor obsidiansis OB47	632335.8.peg.2146	Caldicellulosiruptor kristjanssonii 177R1B
632516.3.peg.1526	Caldicellulosiruptor lactoaceticus 6A	351627.8.peg.408	Caldicellulosiruptor saccharolyticus DSM 8903

```
31899.10.peg.2969  tttggtga-atatttccctgcgggaaggaggttattcccgcagggggatatttaaataagtaaaaaatcgattattttaaaa-gg
632348.3.peg.152   tttggtga-atatttccctgcgggaaggaggttattcccgcagggggatatttaaataagtaaaaaatcgattattttaaaaagg
632292.3.peg.140   tttagaaa-atgtttccctgcgggaaaagacttgt-cccgcagggag-catttaaattag--aaaaattgattattttaaaa-ac
632516.3.peg.1526  tttggtaa-gtatttccctgcgggaaga-ggcttgtcccgcagggag-cattt--agaattaccaaaatgtttattttaaaa-at
1121259.3.peg.1423 tttggtaa-gtatttccctgcgggaaga-ggcttgtcccgcagggag-cattt--agaattaccaaaatgtttattttaaaa-at
632335.8.peg.2146  tttggtaa-gtatttccctgcgggaaga-ggcttgtcccgcagggaa-cattt--agaattaccaaaattttattttaaaa-at
608506.3.peg.2316  tttggtaa-atatttccctgcgggaagt-gacttgtcccgcagggag-cattt--aaattagcaaaattgattattttaaaa-ag
632518.3.peg.2273  tttggtaa-atatttccctgcgggaagt-gacttgtcccgcagggag-cattt--aaattagcaaaattgattattttaaaa-ag
1214564.3.peg.1491 tttggtaa-atatttccctgcgggaaggtggttattcccgcagggag-cattt--aaattagcgaaattgataattttaaag-cc
351627.8.peg.408   ttttgtaa-atatttccctgcgggaaggtggttattcccgcagggag-cattt--aaattagcgaaattgataattttaaag-cc
1222016.3.peg.371  cttagaaatatgtttccctgcgggaaga-gacttgtcccgcagggag-catatgagagttgtcaaaattgtttattttaaaa-at
1387557.3.peg.2819 tttggtaa-gtatttccctgcgggaaga-gacttttcccgcagggag-tatttggaagctcttaaaattgtttattttaaaa-at
                    ** *  *  * **************      *   *********    ** *           ***    * ********
```

```
31899.10.peg.2969  cagaaaataatgctataatattttT-atgaataatacgcaacagcgtttcacaatacgaaacaggagagtagaatATGGCGCAAAA
632348.3.peg.152   cagaaaataatgctataatattttt-atgaataatacgcaacagcatttcacaatacgaaacaggagagtagaatATGGCGCAAAA
632292.3.peg.140   cagaaaagaatgttataatattttt-gtgaataatacgcaacaccgtttcacaatacgaaacaggagagtataagATGGCGCAAAA
632516.3.peg.1526  cataaaacaatgctatagtatttt--gtgaacaatacgaaacagcgtttcacaatacgaaacaggagaggctgatATGGCGCAAAA
1121259.3.peg.1423 cataaaacaatgctatagtatttt--gtgaacaatacgaaacagcgtttcataatacgaaacaggagaggctgatATGGCGCAAAA
632335.8.peg.2146  cataaaagaatgttataatatttt--gcgaacaatacgaaacagcgtttcacaatacgaaacaggagaggctgatATGGCGCAAAA
608506.3.peg.2316  cagaagagagtgctataatattttt-gtgaacaatacgaaacagcgtttcataatatgaaacaggagagactgatATGTCGCAAAA
632518.3.peg.2273  cagcaaagaatgctataatattttt-gtgagcaatacgaaacagcgtttcacaatatgaaacaggagagactgatATGTCGCAAAA
1214564.3.peg.1491 cagaaaataatgctataatattttt-atgaacaatacgaaacagtatttcacaatacgaaacaggagagactgatATGTCACAAAA
351627.8.peg.408   cagaaaggaatgctataatattttt-atgaacaatacgaaacagtatttcacaatacgaaacaggagagactgatATGTCACAAAA
1222016.3.peg.371  cataaaataatgctataatgtttt-gtgaacaatacgaaacagcatttcacaatacgaaacaggggagactgatATGTCACAAAA
1387557.3.peg.2819 cataaaataatgctataattttttttgtaaacaatacgaaacagtatttcacaatacgaaacaggggagactgatATGTCACAAAA
                   **  *   * ** **** * ****     *  ****** ****   ***** **** ******** ***    * *** * *****
```

31899.10.peg.1896 (Athe_1402) **uxaC** **new inverted repeat motif for unknown TF** **(motif 4) (potential repressor)**

31899.10.peg.1896 Caldicellulosiruptor bescii strain DSMZ 6725
632335.8.peg.1510 Caldicellulosiruptor kristjanssonii 177R1B
1121259.3.peg.230 Caldicellulosiruptor acetigenus DSM 7040
1387555.3.peg.1427 Caldicellulosiruptor sp. Rt8.B8
632348.3.peg.1384 Caldicellulosiruptor kronotskyensis 2002
632292.3.peg.1416 Caldicellulosiruptor hydrothermalis 108
351627.8.peg.2129 Caldicellulosiruptor saccharolyticus DSM 8903
608506.3.peg.1248 Caldicellulosiruptor obsidiansis OB47
632516.3.peg.132 Caldicellulosiruptor lactoaceticus 6A
632518.3.peg.1266 Caldicellulosiruptor owensensis OL

```
31899.10.peg.1896  TAActccacaaaaatccaaatataagattttgaattcaaaacagaataaagagagagggaaagtg
632335.8.peg.1510  TAActccacaaaagtccaaagataagtttttggattcacaatggaataaagagagagggaaagtg
1121259.3.peg.230  TAActccacaaaagtccaaagataagtttttggattcacaatggaataaagggagagggaaagtg
1387555.3.peg.1427 TAActccacaaaagtccaaaggaaagtttttgaatttgcaatagaataaagaaagagggaaagtg
632348.3.peg.1384  TAActccacaaaagtccaaatataagattttgaattcaaaacagaataaagagagagggaaagtc
632292.3.peg.1416  TAAatccacaaaagtccaaagataag-ctttggattcacaatggaattaagatagagggaaagtg
351627.8.peg.2129  TAActccacaaaagtccaaagaaaagtttttgaattcataatggaataaagaaagagggaaagtg
608506.3.peg.1248  TAActccacaaaagtccaaagataagtttttggattcacaatggaataaagagagagggaaagtg
632516.3.peg.132   TAActccacaaaagtccaaagataagtttttggattcacaatggaataaagagagagggaaagtg
632518.3.peg.1266  TAActccacaaaagtccaaagataagtttt-ggactcacaatggaacaaagagagagggaaagtg
                   *** ********* ******   ***  ** * * *   **  ***  ***  ***********

31899.10.peg.1896  ttgaactttccctcttttcattttataatatattCaaacagattttgttgtgaattttatttgaatcttttataaattgtgttgaaataagtttcaaggaggtttggaaagATGA
632335.8.peg.1510  ttgaactttccctcttttcattttataatatattcaaacagattttgttgtgaattttgtttgaatcttttagaaattgtgttgaaacaagtttcaaggaggtttggaaagATGA
1121259.3.peg.230  ttgaactttccctcttttcattttataatatattcaaacagattttgttgtgaattttgtttgaatcttttagaaattatgttgaaacaagtttcaaggaggtttggaaaaATGA
1387555.3.peg.1427 ttgaactttccctcttttcattttataatatattcaaacaggatatgttaagaattttgtt-gaatcttttggaaattatgttgaaacaagtttcaaggaggtttggaaagATGA
632348.3.peg.1384  ttgaactttccctcttttcattttataatatattcaaacagattttgttgtgaattttgtttgaatcttttagtaattgtgttgaaataagtttcaaggaggtttggaaagATGA
632292.3.peg.1416  ttgaactttccctcttttcattttataatatattcaaacagattctgttgtgaattttgtttgaatcttttagaaattgtgttgaaacaagtttcaaggaggtttggaaagATGA
351627.8.peg.2129  ttgaactttccctcttttcattttataatatattcaaacagattttgttgtgaattttgtttgaatcttttagaaattgtgttgaaacaagtttcaaggaggtttggaaagATGA
608506.3.peg.1248  ttgaactttccctcttttcattttataatatattcaaatagattttactgtgaattttgtttgaatcttttagaaatcttgttgaagtaaattttaaggaggtttggaaagATGA
632516.3.peg.132   ttgaactttccctcttttcattttataatatattcaaacagattttgttgtgaattttgtttgaatcttttagaaattatgttgaaacaagtttcaaggaggtttggaaaaATGA
632518.3.peg.1266  ttgaactttccctcttttaattttataatatattcaaacagattttgctgtgaattttgtttgaatctttttgaaattatgttgaaacaagttacaaggaggtttggaaagATGA
                   ****************** ******************* **  * *  *  ******* ** *********   ***  ******** ** **  ********************
```

31899.10.peg.1314 (Athe_0854) *aguX-uxuBA-bgaL* XynR-binding site (potential repressor)

31899.10.peg.1314 Caldicellulosiruptor bescii strain DSMZ 6725
608506.3.peg.865 Caldicellulosiruptor obsidiansis OB47
632292.3.peg.1920 Caldicellulosiruptor hydrothermalis 108
632516.3.peg.2060 Caldicellulosiruptor lactoaceticus 6A
1121259.3.peg.1821 Caldicellulosiruptor acetigenus DSM 7040
632335.8.peg.2015 Caldicellulosiruptor kristjanssonii 177R1B
1387557.3.peg.1127 Caldicellulosiruptor sp. Wai35.B1
632518.3.peg.768 Caldicellulosiruptor owensensis OL
632348.3.peg.1897 Caldicellulosiruptor kronotskyensis 2002

```
31899.10.peg.1314  AGAAATTCTGTTTCTGTCAACGAGGAGTAAtgtggtttttatta-tttagaaatcgttttctaaatagctgcggaaaggtgtgattaa-ttATGATTTT
608506.3.peg.865   AGAAATTCTGTTTCTGTCAACGAGGAGTAAtgtagtttttatta-tttagaaatcgttttctaaa-acctgcggaagggtgtggttaa-ttATGATTTT
632516.3.peg.2060  AGAAATTCTGTTTCTGTCAATGAAGATTAAttttctatggtatt-ttaagaaatcgttttctaaaaatctgcgggaggctgggatttg-ctATGATTTT
1121259.3.peg.1821 AGAAATTCTGTTTCTGTCAATGAAGATTAAttttctatggtatt-ttaagaaatcgttttctaaaaatctgcgggaggctgggatttg-ctATGATTTT
632335.8.peg.2015  AGAAATTCTGTTTCTGTCAATGAAGATTAAttttctatagtatt-ttaagaaatcgttttctaaaaatctgcaggaggctgggatttg-ctATGATTTT
632292.3.peg.1920  AGGAATTCTGTATCTGTCAATGAAGACTAAttttctatgttatt-tttagaaatcgttttctaaaaatctgcaggaggttgtgatttg-ctATGATTTT
1387557.3.peg.1127 AGGAATTCTATATCTATCAATGAAGATTAAttttctatgttatt-tttagaaatcgttttctaaaaatctgcaggaggttgtgatttg-ctATGATTTT
632348.3.peg.1897  AGGAATTCTGTATCTGTCAATGAAAGTTAAttttc-agaaacta-aaaagaaatcgttttctaaaa-taagcaggggaagtgatttg-ttATGATTTT
632518.3.peg.768   AGAAATTCAGTTTCTGCAAACGATGGGTAGtattatttttattactttagaaatcgttttctaaaa-tttaaagaaaggtgtgattaaattATGATTTT
                   ** *****  * ***   ** **    ** * *         *     *****************        *   *  * * **    *********
```

31899.10.peg.472 (Athe_0088) *Athe_0088-xynA2* XynR-binding site (weak site score, not conserved) (potential repressor)

31899.10.peg.472 Caldicellulosiruptor bescii strain DSMZ 6725
632348.3.peg.84 Caldicellulosiruptor kronotskyensis 2002
632518.3.peg.44 Caldicellulosiruptor owensensis OL
1387555.3.peg.462 Caldicellulosiruptor sp. Rt8.B8

```
31899.10.peg.472  aaaaaa-gtttaattttttaggtatttttttaagattttagggtttacattttctagttgttaatttataataaacTaaccttttgataaagtgat
632348.3.peg.84   aaaaaaagtttaattttttaggtatttttttaagattttagggtttacattttctaattgtgaatttataataaactcaccttttgataaagtgat
632518.3.peg.44   aaaaaa-gtataatttttagggtaattttt-aaaattttagggtttacattttataattgtgacactataataaaatcaccatctgaaagaagaaa
1387555.3.peg.462 aaagaa-gtatgatttataggggatttttt-aaaattttagggtttacagaagttaattgtgaatttataataaaatcactaacaacaaagaagag
                  *** ** ** * **** *  ** * ***** ** ***************     ** **** *   ********* * **        *     *

31899.10.peg.472  taaataaaaagattttaataaaccgttgtgtaatatgcattaagatatgaagtaaataaaataacggtaaaaaataaacaaaattttgtaaca
632348.3.peg.84   taaataaaaagattttaataaaccgttgtgtaatatgcattaagataggaagtaaataaaataacggtaaaaaataaacaaaattttgtaaca
632518.3.peg.44   aaaataataagattttaccaaaagattgtggagtatgcagtaaac-----agaaaatgagaaggtagttaatga-taaatgataccggtttctaat
1387555.3.peg.462 taaatag-gatatttgtccaattaatta--gaagatgtatttaatattatgattaaaaatttagagattgtta--tacatgacttggactgtgatt
                   *****   * ****    **    **    *  *** * * *          **  *        *       ** *  *       *   *

31899.10.peg.472  ttatttaagtgaagggaaggtgaaaggaggtagcaatGTGACAGACTTATATATAACCATTTTATTTGTC
632348.3.peg.84   ttatttaagtgaagggaaggtgaaaggaggtagcaatGTGACAGACTTGTATATAACCATTTTATTTGTC
632518.3.peg.44   ttgttga--tgaaaataaagaggaaggggggta-tagcGTGCAAAATATATTCATTGTTGCTTTATTGGTT
1387555.3.peg.462 gtattt---------tgacttgaaaggaggaggaaaaGTGAATAACTCTTTATTTGTAAACCTTCTAATT
                   * **            *   * **** **    *  ***    *    *   *       *  *  *
```

31899.10.peg.1309 (Athe_0849) *bxgLFG* BxgRS-binding site (potential activator)

31899.10.peg.1309	Caldicellulosiruptor bescii strain DSMZ 6725	632516.3.peg.2055	Caldicellulosiruptor lactoaceticus 6A
632348.3.peg.1902	Caldicellulosiruptor kronotskyensis 2002	1387557.3.peg.1122	Caldicellulosiruptor sp. Wai35.B1
1121259.3.peg.1826	Caldicellulosiruptor acetigenus DSM 7040	608506.3.peg.860	Caldicellulosiruptor obsidiansis OB47
632292.3.peg.1925	Caldicellulosiruptor hydrothermalis 108	1222016.3.peg.924	Caldicellulosiruptor changbaiensis strain CBS-Z
632335.8.peg.2020	Caldicellulosiruptor kristjanssonii 177R1B		

```
31899.10.peg.1309  AAAATATAGacttttgacatatcaattttatgggggaaatatgttagattttaagggttgttttttatctaaatactatgtaaaatgaacAgtagagaatga
632348.3.peg.1902  AAAATATAGacttttgacatatcaattttatgggggaaatatgttagattttaagggttgttttttatctaaatactatgtaaaatgaacagtagaggatga
1121259.3.peg.1826 AAAATATAGtttttttgacatatcaattttatgggggaaatatgttagattttaagggttgttttttatctaaatactatgtaaaatgaacagtagaggatga
632516.3.peg.2055  AAAATATAGtttttttgacatatcaattttatgggggaaatatgttagatcttaagggttgttttttatctaaatactatgtaaaatgaacagtagaggatga
632335.8.peg.2020  AAAATATAGtttttttgacatatcaattttatgggggaaatatgttagattttaagggttgttttttatctaaatactacgtaaaatgaacagtagaggatga
632292.3.peg.1925  AGAATATAGtttttttgacatatcaattttatgggggaaatatgttagattttgagggttgttttttatctaaatactatgtaaaatgaacagtagaggatga
1387557.3.peg.1122 AAAATATAGtttttttgacatatcaattttatggggtaaatatgttagattttaagggttgttttttatctaaatactatgtaaaatgaacagtagaggatga
608506.3.peg.860   AAAATATAGtttttttgacatatcaattttatgggggaaatatgttagattttaagggttgttttttacctaaatactatgtaaaatgaacagtagaggatga
1222016.3.peg.924  AGATTATAAacttct-atatatcaattttatggggtaaatatgttagattttaagggttgttttttaaacaaatactatgtaaaatgaacaatagaggatgt
351627.8.peg.2911  AGATTGTAAacttct-atatatcaattttatggggtaaatatgttagattttaagggttgttttttaaataaatactatgtaaaatgaacaatagaggatgt
                         **      *    **  ** * *   *     ** ***** ** ** *************   ******** ************ ***** ***

31899.10.peg.1309  taattgttcatccttgataaaaattcaaagagaggaggcgtt-tcaatTTGAAATTTTTAA
632348.3.peg.1902  taattgttcatccttgataaaaattcaaagagaggaggcgtt-tcaatTTGAAATTTTTAA
1121259.3.peg.1826 taattgttcatcctaaataaaaattcaaggaggggaggcgtg-taaatTTGAAATTTATAA
632516.3.peg.2055  taattgttcatcctaaataaaaattcaaggagaggaggcgtg-taaatTTGAAATTTATAA
632335.8.peg.2020  taattgttcatcctaaataaaaattcaaggagaggaggcgtg-taaatTTGAAATTTATAA
632292.3.peg.1925  taattgttcatcctaaataaaaattcaaggagaggaggcgtg-taaatTTGAAATTTATAA
1387557.3.peg.1122 taattgttcatcctaaataaaaattcaaggagaggaggcgtg-caaatTTGAAATTTGTAA
608506.3.peg.860   taattgttcatcctaaataaaagttcaaggagaggaggcgt--caaaaTTGAAATTTATAA
1222016.3.peg.924  aaaaaata-atcct---cagaaaattaaaagaaggaggcattcttaaaGTGAGTTTTAAAA
351627.8.peg.2911  taaaaata-atcct---cagata-ttaatagaaggaggtattcttaaaGTGAGCTTTAAAA
                    **   *  *****    * *   * **     *****  *    **  ***  ***  **
```

31899.10.peg.1307 (Athe_0847) *bxgL1-X* Predicted BxgRS-binding site (potential activator)

31899.10.peg.1307	Caldicellulosiruptor bescii strain DSMZ 6725	608506.3.peg.858	Caldicellulosiruptor obsidiansis OB47
632348.3.peg.1904	Caldicellulosiruptor kronotskyensis 2002	351627.8.peg.2913	Caldicellulosiruptor saccharolyticus DSM 8903
1121259.3.peg.1828	Caldicellulosiruptor acetigenus DSM 7040	1222016.3.peg.922	Caldicellulosiruptor changbaiensis strain CBS-Z
632335.8.peg.2022	Caldicellulosiruptor kristjanssonii 177R1B	1387555.3.peg.2040	Caldicellulosiruptor sp. Rt8.B8

```
31899.10.peg.1307  ggctccaaaaaa-gtcaattttcaggggaaaaatataaaattttcagggttacatttctacaatttatagttataatttattttAg
632348.3.peg.1904  ggctccaaaaaa-gtcaattttcaggggaaaaatataaaattttcagggttacatttctacaatttatagttataatttatttcag
1121259.3.peg.1828 ggatttaaaaaaagtcaatttgcggggaaaaatataaaattttcagggttacatttctacaatttatagttataatttatttcag
632335.8.peg.2022  ggatttaaaaaaagtcaatttgcggggaaaaatataaaattttcagggttacatttctacaatttatagttataatttatttcag
608506.3.peg.858   ggattaaaaaaa-gtcaatttgtggggaaaaatataaaattttcagggttacacttttataatttatagctataatttatttcag
351627.8.peg.2913  agtttaaaaacaagtcaagtttatggggtaaaattattaaatttttagggtcacaagttttaattttatagttataatttacatgta
1222016.3.peg.922  agtttaaaaacaagtcaagtttatggggtaaaattattaaatttttagggtcacaagttttaattttatagttataatttacatgta
1387555.3.peg.2040 aatctgcaaa-aagtcaaatttatagggtaaaattataaatttttgggggtaaaaaaattataattcattgttataatttaaccata
                          *** * ***** ***   ***  *** *** ** *****  **** * *    *  * ** ** * *********

31899.10.peg.1307  attcacaa---ttttaaaagtctttttgcccgtagaaacgtcaaaata----tacagtaaggagggtttgagATGTCAAAAA
632348.3.peg.1904  attcacaa---tttttaaagtctttttgctcgtagaaatgtcaaaata----tacagtgaggagggtttgagATGTCAAAAA
1121259.3.peg.1828 attcacaa---tttttaaagtctttttgcccttggaaatgtcaaaata----tacagtgaggagggtttggtATGTCAAAAA
632335.8.peg.2022  attcacaa---tttttaaagtctttttgcccttggaaatgtcaaaata----tacagtgaggagggtttggtATGTCAAAAA
608506.3.peg.858   attcacaa---tttttatagtctttttgctcgtgaaaatgtcaaaata----tacagtgaggagggtttggtATGTCAAAAA
351627.8.peg.2913  atgattagaggattttcttttagtgctggtgtggtatgtatcaaaaaaa-tctataaaggggaggagtttgaATGACAAAAA
1222016.3.peg.922  atgattagaggattttcttttagtgctggtgtggtatgtataaaaaaaaatctagaaaggggaggagtttgaATGTCAAAAA
1387555.3.peg.2040 gcattcaa---attttatctcctgttaaaacatttcttatcaaaattacacaaagagggaggagaggtt--ATGTCCAAAA
                         *     ***     *  *  *             * ****       *       *  *   *   *** * ****
```

31899.10.peg.1408 (Athe_0938) *galEKT* potential terminator downstream of Athe_0937

31899.10.peg.1408	Caldicellulosiruptor bescii strain DSMZ 6725	632518.3.peg.813	Caldicellulosiruptor owensensis OL
632348.3.peg.1829	Caldicellulosiruptor kronotskyensis 2002	1387557.3.peg.1205	Caldicellulosiruptor sp. Wai35.B1
632292.3.peg.1861	Caldicellulosiruptor hydrothermalis 108	1222016.3.peg.1901	Caldicellulosiruptor changbaiensis strain CBS-Z
632516.3.peg.2227	Caldicellulosiruptor lactoaceticus 6A	1387569.3.peg.2489	Thermoanaerobacter cellulolyticus NA10
1121259.3.peg.960	Caldicellulosiruptor acetigenus DSM 7040	1387555.3.peg.1947	Caldicellulosiruptor sp. Rt8.B8
632335.8.peg.1053	Caldicellulosiruptor kristjanssonii 177R1B	1214564.3.peg.1210	Caldicellulosiruptor sp. F32
608506.3.peg.1666	Caldicellulosiruptor obsidiansis OB47	351627.8.peg.1668	Caldicellulosiruptor saccharolyticus DSM 8903

```
31899.10.peg.1408  TAAaaaagaagtgcattcaaattgcacttctttttttttagaatgaaaaatAggaagaaagga-----gaaaaaattttattttgat-gtg-gggga-gaaaaaATG
632348.3.peg.1829  TAAaaaagaagtgcattcaaattgcacttctttttttttagaatgaaaaataggaagaaagga-----gaaaaagttttattttgattgtg-gggga-gaaaaaATG
632292.3.peg.1861  TAGaaaagaagtgcattcaaattgcacttctttttttttagaatgaaaattaggaagaaagga-----gaaaaaattttattt-gat-gtg-gggga-gaaaaaATG
1121259.3.peg.960  TAAaaaagaagtgcattcaaattgcacttctttttttttagaatgaagattaggaagaaagga-----gaaaaa-ttttgttttgat-gtgaggga-gaaaaaATG
632335.8.peg.1053  TAAaaaagaagtgcattcaaattgcacttcttttttttt-agaatgaagattaggaagaaagga-----gaaaaa-ttttgttttgat-gtgaggga-gaaaaaATG
632516.3.peg.2227  TAAaaaagaagtgcattcaaattgcacttctttttttttagaatgaagattaggaagaaagga-----gaaaaa-ttttgttttgat-gtgaggga-gaaaaaATG
1387557.3.peg.1205 TAAaaaagaagtgcatttaatttgcacttctttttttttagaatgaaaattaggaagaaagga-----gaaaaaattttattttgat-gtg-gggga-gaaaaaATG
608506.3.peg.1666  TAGaaaagaagtgcattcaaattgcacttctttttttttagaatgaaaattaggaagaaagga-----gaaaaaattttattt-gat-gtg-gggga-gaaaaaATG
632518.3.peg.813   TAGaaaagaagtgcattcaaattgcacttcttttttttt-agaatgaaaattaggaagaaagga-----gaagaaattttattt-gat-gtg-gggga-gaaaaaATG
1222016.3.peg.1901 TAAaaaagaagtgcatttaaattgcacttctttttttttagaatgaaaatagaagaaa--agc----taaaaaaataatttaaaaac-atgggggag--aaaagATG
1214564.3.peg.1210 TAAaaaagaagtgcatttaaattgcacttctttttttttagaatgaaaatagaagaaa--agc----taaaaaa-taatttaaaaac-atgggggag--aaaagATG
351627.8.peg.1668  TAAaaaagaagtgcatttaaattgcacttctttttttttagaatgaaaattaggaaaa--agc----taaaaaa-taatttaaaaac-atgggggag--aaaaaATG
1387569.3.peg.2489 TAAaaaagaagtgcatttaaattgcacttcttttttatcagaatgaaaattaggaaaa--agc----taaaaaa-taatttaaaaac-atgggggag--aaaaaATG
1387555.3.peg.1947 TAAaaaagaagtgcattcaaattgcacttctttttttttagaatgaaattaggaaaaacaaacccaataaaaaacaattatacagat-gtgaggggatagaaagATG
                   ***************** ** *************** * ********        * *           ** **    * *    *   ** ***     *** ***
```

31899.10.peg.888 **(Athe_0468)** ***agaL2***

31899.10.peg.888	Caldicellulosiruptor bescii strain DSMZ 6725	632516.3.peg.2292	Caldicellulosiruptor lactoaceticus 6A
1214564.3.peg.310	Caldicellulosiruptor sp. F32	1387557.3.peg.689	Caldicellulosiruptor sp. Wai35.B1
1387569.3.peg.625	Thermoanaerobacter cellulolyticus NA10	632292.3.peg.2286	Caldicellulosiruptor hydrothermalis 108
351627.8.peg.1257	Caldicellulosiruptor saccharolyticus DSM 8903	1222016.3.peg.551	Caldicellulosiruptor changbaiensis strain CBS-Z
632348.3.peg.2280	Caldicellulosiruptor kronotskyensis 2002	1387555.3.peg.2501	Caldicellulosiruptor sp. Rt8.B8
1121259.3.peg.2201	Caldicellulosiruptor acetigenus DSM 7040	632518.3.peg.376	Caldicellulosiruptor owensensis OL
632335.8.peg.1996	Caldicellulosiruptor kristjanssonii 177R1B	608506.3.peg.480	Caldicellulosiruptor obsidiansis OB47

```
31899.10.peg.888   agtgaatatagcaaaacatctctctt-gatatattataaattttAaaagaaccagctt----------aaaaagaaggaggcttaaaagATGCCAA
1214564.3.peg.310  agtgaatatagcaaaacatttttcattaatatattataaattttgaaaatacaagctt-----------aaaaaaaggaggccaaaaagATGCCAA
351627.8.peg.1257  agtgaatatagcaaaacattttccattaatatattataaattttgaaagtacaaactt----------gaaaaaaaggaggcccaaaatATGTCAA
632348.3.peg.2280  agtgaatatagcaaaacatctcacat-gatatattataaattttaaaagaaccagctt----------aaaaagaaggaggtttaaaagATGCCAA
632335.8.peg.1996  agggaatatagcaaaacacctttcat-gatatattataaatcttaaaagcctaaaatt----------gaaaatgaggaggacttaaaaATGCCAA
632516.3.peg.2292  agggaatatagcaaaacacctttcat-gatatattataaatcttaaaagcctaaaatt----------gaaaatgaggaggacttaaaaATGCCAA
632518.3.peg.376   aaggagtgtagataaaaatctttgga-aatatatgataaaattcgaaaattca--------------aaggcaaaaggaggttttaaaaATGCCAA
608506.3.peg.480   aatgaatgtagataaaaatctttgaa-aatatatgataaaattcggaaattcataggttga-----aaaggcaagaggaggttttgaaaATGCCAA
1387555.3.peg.2501 agaaaatatggataacagaatttctc-agtatattataatcttaaactacaaaaaata----------aacgaaagggtgaaatgaaaaATGCCAA
1121259.3.peg.2201 attaggtatggtgaaaatattttaccag-tctattattaa--tt-ttgaaacctaaattt--------taaaaaggaggatttccagtaATGCCAA
1387557.3.peg.689  aataggtatagtgaaaatactttactcg-tctattattaa--tt-gtgaatcctaaattt--------taaaagggaggatttccagt-ATGCCAA
632292.3.peg.2286  aattgctatagaaaaagaaagtaccttgatatattattaaagtt-gcgggataaaaatttataaagaacataaagaaggggcaacaaaATGCCAA
1222016.3.peg.551  aattgctatagaaaaagaaagtaccttgatatattattaaagtt-gcgggataaaaatttataaagaacataaagaaggggcaacaaaATGCCAA
1387569.3.peg.625  agaaaatatgg-attattagctttttaaatgtattattgatcttagtgataaataaata------------gaaagaggaatgagcaaaATGCCAA
                   *     * * *                  * *** **     *                             *    *           *** ***
```

31899.10.peg.1585 **(Athe_1104)** ***agaL1***

31899.10.peg.1585	Caldicellulosiruptor bescii strain DSMZ 6725	1222016.3.peg.1847	Caldicellulosiruptor changbaiensis strain CBS-Z
632348.3.peg.1679	Caldicellulosiruptor kronotskyensis 2002	351627.8.peg.1721	Caldicellulosiruptor saccharolyticus DSM 8903
632335.8.peg.1213	Caldicellulosiruptor kristjanssonii 177R1B	632518.3.peg.1002	Caldicellulosiruptor owensensis OL
632292.3.peg.1715	Caldicellulosiruptor hydrothermalis 108	1387557.3.peg.1344	Caldicellulosiruptor sp. Wai35.B1
1121259.3.peg.1115	Caldicellulosiruptor acetigenus DSM 7040	608506.3.peg.1529	Caldicellulosiruptor obsidiansis OB47

```
31899.10.peg.1585  tct-gatagaagtttcaaaattataagaatttaaatacagcggccgaaaggttaaaataaaagAtatattc----atggaaggagatgagagtATGCTC
632348.3.peg.1679  tct-gatagaagtttcaaaattataagaatttaaatacagcggccgaaaggttaaaataaaagatatattc----atggaaggagatgagggtATGCTC
632292.3.peg.1715  tct-aatagaagtttcaaaattattaga-tttaaatacagcggccgaaaggttaaaataaaagatatattc----atggaaggagatgagagtATGCTC
632335.8.peg.1213  ttttaagagaaatttcaaagttattagatttaaactaccgccactgagaagttaaaataaaaaatgtatttca-gagaaaaggagatgagagtATGCTC
351627.8.peg.1721  ttcttacagaaatttcaaaattattaga-ttcaaatacagtagccgaaaagttaaaataaagaataaattcattaatgaaaggagatgaaggtATGCTC
1387557.3.peg.1344 t-ctgatagaaatttcaaaattattaga-tttaaatacagtagctaaaaacttaaaataaaagataaattcatta-tggaaggagatgagagtATGCTC
1222016.3.peg.1847 ttctgatagaactttcaaaattataagaatttaaatacagcggccgaaaggttaaaataaaagatatattcat----ggaaggagatgagagtATGCTC
632518.3.peg.1002  t-ctaatagaaatttcaaaattattaga-ttcaactacagtagccgaaaagttaaaataaagaataaattcattaatggaaggagatgagggtATGCTC
608506.3.peg.1529  t-ctaatataagtttcaaaattat-aag-tttaaatacagcagccaaaaggttaaaataaaatatatattcat--atggaaggagatgagggtATGCTC
1121259.3.peg.1115 t--------------ctaaattattaga-tttaaatacagcggccgaaaggttaaaataaaagatatattcat--atggaaggagatgagggtATGCTC
                   *              * ** **** *   ** ** *** *   *  * *  **********  **  ***         **********  ********
```

31899.10.peg.2950 (Athe_2370) *ebgM*

31899.10.peg.2950	Caldicellulosiruptor bescii strain DSMZ 6725	608506.3.peg.2302	Caldicellulosiruptor obsidiansis OB47
632348.3.peg.171	Caldicellulosiruptor kronotskyensis 2002	632516.3.peg.911	Caldicellulosiruptor lactoaceticus 6A
351627.8.peg.419	Caldicellulosiruptor saccharolyticus DSM 8903	1121259.3.peg.1415	Caldicellulosiruptor acetigenus DSM 7040
1387557.3.peg.2810	Caldicellulosiruptor sp. Wai35.B1	632518.3.peg.2245	Caldicellulosiruptor owensensis OL
1222016.3.peg.381	Caldicellulosiruptor changbaiensis strain CBS-Z	632292.3.peg.158	Caldicellulosiruptor hydrothermalis 108

```
31899.10.peg.2950  tttgatattgtaaagtaa-ttgtaatataaagtaaaataaatagtGtaaaaacaaagccttgttacaatttctaaaa----gaggtgatactctGTGCTTGA
632348.3.peg.171   tttgatattgtaaagtaa-ttgcaatataaagtaaaataaatagtgtaaaaacaaagccttgttacaatttctaaaa----gaggtgatactctGTGCTTGA
632516.3.peg.911   tttgatattgtatgttca-tctgctaataaattaaaatgaataatgtata-----acc---atagttactttaaaaa-tgtgaggtgaaatt-cATGCTTGA
1121259.3.peg.1415 tttgatattgtatgttca-tctgctaataaattaaaatgaataatgtatataaaaacc---atagttattttaaaaa-tgtgaggtgaaatt-cATGCTTGA
632518.3.peg.2245  tttgatattgtatgtcta-cttgttgataaagtaaaatgaataatgtacataaaaagcg--atagttattttagaaagtgtgaggtgaaatt-cATGCTTGA
351627.8.peg.419   tttgatattgtatgtcta-cttgttgataaagtaaaatgaataatgtacacaaaaacc---acagtaattttaaaacatgtgaggtggatttatATGCTTGA
1222016.3.peg.381  tttgatattgtatgtcta-cattttgataaagtaaaatgaataatgtacacaaaaacc---acagtaattttaaaacatgtgaggtggatttatATGCTTGA
1387557.3.peg.2810 tttgatattgtatgtcta-cttgttgataaagtaaaatgaataatgtacacaaaaacc---acagtaattttaaaacgtgtgaggtgaatttacATGCTTGA
608506.3.peg.2302  tttgatattgtatgtgca-cctgctaataaattaaaatgaataatgtatataaaaacg---atagttattttaaaaa-tgtgaggtgaaatt-tATGCTTGA
632292.3.peg.158   tattgtcttttctgttaatccctttattcaacaagctatata-tattaacggtaagtttgctggtttttcaaaggtctctcacATGATGCA-TGAGTTTGA
                   *    * ** * * *  *        **  *  **  *  *** * *  *     *             *            *             * ****
```

31899.10.peg.754 (Athe_0343) *iolR*

31899.10.peg.754	Caldicellulosiruptor bescii strain DSMZ 6725	632516.3.peg.1409	Caldicellulosiruptor lactoaceticus 6A
632348.3.peg.2393	Caldicellulosiruptor kronotskyensis 2002	1387557.3.peg.571	Caldicellulosiruptor sp. Wai35.B1
632292.3.peg.2400	Caldicellulosiruptor hydrothermalis 108	608506.3.peg.322	Caldicellulosiruptor obsidiansis OB47
351627.8.peg.1314	Caldicellulosiruptor saccharolyticus DSM 8903	632518.3.peg.244	Caldicellulosiruptor owensensis OL
1214564.3.peg.2267	Caldicellulosiruptor sp. F32	1222016.3.peg.636	Caldicellulosiruptor changbaiensis strain CBS-Z
1121259.3.peg.382	Caldicellulosiruptor acetigenus DSM 7040	1387555.3.peg.774	Caldicellulosiruptor sp. Rt8.B8
632335.8.peg.355	Caldicellulosiruptor kristjanssonii 177R1B	1387569.3.peg.2047	Thermoanaerobacter cellulolyticus NA10

```
31899.10.peg.754   caaaataattacaaatgttgcaagaatattgccaaagatataaaataaaaaa-cAgaagtaaaagtatctgggggtgtaaaATGC
632348.3.peg.2393  caaaataattacaaatgttgcaagagtattgccaaagatataaaataaaaaa-cagaagtaaaagtatctgggggtgtaaaATGC
632292.3.peg.2400  caaaataattacaaatgttgcaaggacattgccaaaggtatagaataaaaaa-cagaagtaaaatcatctgggggtgtaaaATGC
608506.3.peg.322   caaagtaattacaaaggttgcaaggatttttcaaaggtataaaataaaaaa-caaaagtaaaagtatctggggtgtgaaATGC
351627.8.peg.1314  caaaataattacaaatgttgaaaggattttgtcaaaggtataaaataaaaaa-cagaagtaaaagtatctgggggtgtaaaATGC
1214564.3.peg.2267 caaaataattacaaatgttgaaaggattttgtcaaaggtataaaataaaaaa-cagaagtaaaagtatctgggggtgtaaaATGC
1387557.3.peg.571  caaaataattacaaatgttgaaaggattttgccaaaggtataaaataaaaaaacagaagtaaaagtatctgggggtgtaaaATGC
632335.8.peg.355   caaaataattacaaaggttgcaaggattttgccaaaggtataaaataaaaag-cagaagtaaaagtatctgggggtgtaaaATGC
632516.3.peg.1409  caaaataattacaaaggttgcaaggattttgccaaaggtataaaataaaaag-cagaagtaaaagtatctgggggtgtaaaATGC
1121259.3.peg.382  caaaataattacaaaggttgcaaggattttgccaaaggtataaaataaaaag-cagaagtaaaagtatctgggggtgtaaaATGC
632518.3.peg.244   cgagataaatacaaaggttgcaaggattttggcaaaggtataaaataaaaaacataaagtaaaaatatatgggggtgtaaaATGC
1222016.3.peg.636  -agaataa-caga--tattgaatagatggtttgaaaggtataaaataga-ga-cagtaatataaatttttgggggtgtaaaATGC
1387569.3.peg.2047 -agaatag-caatattattgaatagagagtggaaaaggtatagaatagaaga-caggaatataaattttttgggggtgtaaaATGC
1387555.3.peg.774  -aaagcaa-tatt--tgttgaacataaggtcagaagagtataaaatagataa-aa--agtataattttttcgggggtgtaaaATGC
                         *   *      *** *       *   **   **** **** *        * ** **   *  ******** ******
```

31899.10.peg.755 (Athe_0344) *iolGI* IolR-binding sites (repressor)

31899.10.peg.755 Caldicellulosiruptor bescii strain DSMZ 6725
632348.3.peg.2392 Caldicellulosiruptor kronotskyensis 2002
1121259.3.peg.381 Caldicellulosiruptor acetigenus DSM 7040
632335.8.peg.356 Caldicellulosiruptor kristjanssonii 177R1B
632516.3.peg.1410 Caldicellulosiruptor lactoaceticus 6A
608506.3.peg.323 Caldicellulosiruptor obsidiansis OB47
632292.3.peg.2399 Caldicellulosiruptor hydrothermalis 108
351627.8.peg.1315 Caldicellulosiruptor saccharolyticus DSM 8903
1387557.3.peg.572 Caldicellulosiruptor sp. Wai35.B1
632518.3.peg.245 Caldicellulosiruptor owensensis OL
1222016.3.peg.637 Caldicellulosiruptor changbaiensis strain CBS-Z

```
31899.10.peg.755  tttgaaaaacaaaaaataatactttctcatgaaataaaaacaagggagtctgcataaga------aggttatttttt-attttaaa
632348.3.peg.2392 tttgaaaaacaaaaaataatacttcctcatgagctcaagacaagagagtctgcctaag--------ggttattttttt-attttaaa
632292.3.peg.2399 tttgaaaaacaaaaaataatacttcctcatgagctcaagacaagagagtctgcctgag--------ggttattttttt-attcttaa
351627.8.peg.1315 tttaaaaaacaaaaaattatacttcctcatgaactcaagacaagagaatctgcttgaa--------gtctattttttt-attttaaa
1387557.3.peg.572 tttaaaaaacaaaaaattatacttcctcatgagctcaagacaagagaatctgcttgaa--------gtctatttttttattttaaa
632335.8.peg.356  tttaaaaaacaaaagattatacttcctcatgagctcaaaacacgagagtctgcttgat--------gtttatttttttattttaaa
632516.3.peg.1410 tttaaaaaacaaaagattatacttcctcatgagctcaaaacacgagagtctgcttgat--------gtttattttttt-attttaaa
1121259.3.peg.381 tttaaaaaacaaaagattatacttcctcatgagctcaaaacacgagagtctgcttgat--------gtttatttttttattttaaa
608506.3.peg.323  ttaaaacagcaaagaattatacttcctcatgagctcaaaacaagagagtctgcctgat--------gtttatttttt--attttaaa
632518.3.peg.245  tatgtaaaacagagaattattctgccacacgaactgaaaactagagagtcggcatgatttaataaaaattaatttttttgtttt-aa
1222016.3.peg.637 ttttcaaaagaaaagtttattcttccccatgagataaaaacaagagaatcggcatgaa----------gcacttttttgtttttaaa
                  *    * *  * *   * ** **  * ** **  * ** **  * ** ** ** * *             * ****   ** * **

31899.10.peg.755  accattgtaattgattacactatgagttataatatcataTgtaatcaattacac-aattaaggaggcagggaa-aaTTGAAAAA
632348.3.peg.2392 accattgtaattgattacactatgagttataatatcatatgtaatcaattacac-aattaaggaggcagggaa-aaTTGAAAAA
632292.3.peg.2399 accattgtaattgattacactatgagttataatatcttatgtaatcaattacac-tattgaggaggcagggaa-aaTTGAAAAA
351627.8.peg.1315 accgttgtaattgattacactatgagttataatatcttatgtaatcaattacac-tattaaggaggcagggaa-aaTTGAAAAA
1387557.3.peg.572 accattgtaattgattacaccatgagttataatatttatgtaatcaattacaccaatagaggggaagacaa-aaTTGAAAAA
632335.8.peg.356  accattgtaattgattacacaatgagatataatatttatgtaatcaattacaccaataaaggggaaggcta-aaTTGAAAAA
632516.3.peg.1410 accattgtaattgattacacaatgagatataatatttatgtaatcaattacaccaataaaggggaaggcta-aaTTGAAAAA
1121259.3.peg.381 accattgtaattgattacacaatgagatataatatttatgtaatcaattacaccaataaaggggaaggcta-aaTTGAAAAA
608506.3.peg.323  accattgtaattgattacacaatagtatataatgatatatgtaatcatttacactaacgaagggagaagacca-aaTTGACAAA
632518.3.peg.245  actattgtaattgattacagtatgttttataatgatgtatgtaatcaattacacaaaaaaaggggaggaattacaaTTGACTAA
1222016.3.peg.637 ctcattgtaattgattacatttgtaatataattat-tgtgtaatcatttacacaagcaggggaggaatgctg-agATGTTAAA
                      ***************   *    ******    * ******** ******       **           *  **   **
```

31899.10.peg.2905 (Athe_2330) *hemXYZ* Potential HemR-binding site (potential activator)

31899.10.peg.2905	Caldicellulosiruptor bescii strain DSMZ 6725	632335.8.peg.2556	Caldicellulosiruptor kristjanssonii 177R1B
632348.3.peg.238	Caldicellulosiruptor kronotskyensis 2002	608506.3.peg.2256	Caldicellulosiruptor obsidiansis OB47
632518.3.peg.2204	Caldicellulosiruptor owensensis OL	1121259.3.peg.1358	Caldicellulosiruptor acetigenus DSM 7040
632292.3.peg.217	Caldicellulosiruptor hydrothermalis 108	632516.3.peg.956	Caldicellulosiruptor lactoaceticus 6A

```
31899.10.peg.2905  gatgagtacatttttaagaagcttaatattgtcctttaaaccttaacattttcctctttcaaaaagcctcacctccccctttataataaaagctGtaaag
632348.3.peg.238   gatgagtacatttttaagaagcttaatattgtcctttaaaccttaacattttcctctttcaaaaagcctcacctccccctttataataaaagctgtaaag
632335.8.peg.2556  catgagtacatttttaagaagcttaatattgtccttttcaatcttaacattttcctctttaaaaaacttctatcccccctttataataaaagctgtaaag
632516.3.peg.956   catgagtacatttttaagaagcttaatattgtccttttcaatcttaacattttcctctttaaaaaacttctatcccccctttataataaaagctgtaaag
1121259.3.peg.1358 catgagtacatttttaagatgcttaatattatccttttcaatcttaacattttcctctttctaaaagctttatctccccttttataataaaagctgtaaag
632292.3.peg.217   catgagtacatttttaagaagcttaatattgtccttttcaatcttaatattttcctcttccaaaaagctttggctcccctttataataaaagctgtaaag
632518.3.peg.2204  catgagtatatttttaagaagcttaatattgtccttttcaatcttaatattttcctctttcaaaaagctatagtccccctctataattaaaactgtaaag
608506.3.peg.2256  agcaagtacatttttaagaagtttaacattgtccttttcaatcttaatattttcctctttcaaaaagctatagtccccctctataattaaaactgtaaag
                       **** ********** * **** *** ****** ** ***** ***********   ****         ***** ****** *** ********

31899.10.peg.2905  cccgctgacaagttgctaaaaacaataaacttttgtcgcgggtaatttggaaaaaatcaataaaaactttc-aaaacgaggaggtttaaagATGGATTGG
632348.3.peg.238   cccgctgacaagttgctgaaaacaataaacttttgtcgcgggtaatttggaaaaaatcaataaaaactttt-aaaacaaggaggtttaaagATGGATTGG
632335.8.peg.2556  cccgccgacaagttgctgaaaataataaagctttgtcgcgggtaatttggaaaaaatcaataaaaactttt-aaagcaaggaggtttcaaaATGGACTGG
632516.3.peg.956   cccgccgacaagttgctgaaaataataaagctttgtcgcgggtaatttggaaaaaatcaataaaaattttt-aaagcaaggaggtttcaaaATGGACTGG
1121259.3.peg.1358 cccgctgacaagttgctgaaaataataaagctttgtcgcgggtaatttggaaaaaatcaataaaaactttt-aaagcaaggaggtttcaaaATGGACTGG
632292.3.peg.217   cccgccgacaagttgctgaaaacaataagcgcttgctgcgggtaatttggaaaaattcaataaaaactttt-aaaacaaggaggtttcaaaATGGACTGG
632518.3.peg.2204  cccgtcgacaagatgttgaaaacaacaagagcttgccgcgggaattttggaaaaattcaataaaaaattttaaaatgaggaggttgtaaaATGAACTGG
608506.3.peg.2256  cccgtcgacaagatgttgaaaacaacaagagcttgccacgggaattttggaaaaattcaataaaaaatttt-aaaatgaggaggttgtaaaATGAACTGG
                   ****  ****** ** * **** ** **     ***   **** * ********** ********** ***  ***   ********  ** *** * ***
```

31899.10.peg.2904 (Athe_2329) *hemR*

31899.10.peg.2904	Caldicellulosiruptor bescii strain DSMZ 6725	632516.3.peg.957	Caldicellulosiruptor lactoaceticus 6A
632348.3.peg.239	Caldicellulosiruptor kronotskyensis 2002	632292.3.peg.220	Caldicellulosiruptor hydrothermalis 108
1121259.3.peg.1356	Caldicellulosiruptor acetigenus DSM 7040	1387557.3.peg.2760	Caldicellulosiruptor sp. Wai35.B1
632335.8.peg.2554	Caldicellulosiruptor kristjanssonii 177R1B		

```
31899.10.peg.2904  ggacaatattaagcttcttaaaaatgtactcatcatttatattttctttgcaatttgtttcattgctttcaatattgcttgtattataattgaaattGc
632335.8.peg.2554  ggacaatattaagcttcttaaaaatgtactcatgatttatattttctttgcaatttgtttcattgctttctatattgcttgtattataattgaaattgc
632516.3.peg.957   ggacaatattaagcttcttaaaaatgtactcatgatttacattttctttgcaatttgtttcattgctttctgtattgcttgtattataattgaaattgc
1121259.3.peg.1356 ggataatattaagcatcttaaaaatgtactcatgatttatattttctttgtaatttgtttcattgctttctgtattgcttgtattataattgaaattgc
632292.3.peg.220   ggacaatattaagcttcttaaaaatgtactcatgatttatattttctttgcaatttgtttcattgctttctatattgcttgtattataattgaaattgc
632348.3.peg.239   ggacaatattaagcttcttaaaaatgtactcatcatttatattttctttgtaatttgtttcattgctttcaatattgcttgtattataattgaaattgc
                   *** ********** ***************** ***** ********** *******************  **************************
```

```
31899.10.peg.2904  ccaaaaatgagggtttaa--atatatgatatttgaggaggatttttttaagtATGTTCAAGAAAATATTGTGGCGATTTATCTTTTCATATCTTGTTATT
632335.8.peg.2554  ctaaaaagaagagttaca-aaattcatttttttgaggaggatttttttaagtATGTTTAAGAAAATCTTGTGGCGGTTTGTCTTTTCATATCTTGTCATT
632516.3.peg.957   ccaaaaaagaaggactta-cgaatttatttttttgaggaggatttttttaaggATGTTTAAGAAAATCTTGTGGCGGTTTGTCTTTTCATATCTTGTCATT
1121259.3.peg.1356 ccaaaaagaaaggctaca-gaatttatttttttgaggaggatttctttaaggATGTTTAAGAAAATCTTGTGGCGGTTTGTCTTTTCATATCTTGTTATT
632292.3.peg.220   ccaaaaaagaagggcttacgaatttatttttttgaggaggatttttttaaggATGTTTAAGAAAATCTTGTGGCGGTTTGTCTTTTCATATCTTGTCATT
632348.3.peg.239   ccaaaaatgagggtttaa--atatatgatatttgaggaggatttttttaagtATGTTCAAGAAAATATTGTGGCGATTTGTCTTTTCATATCTTGTTATT
                   * *****   *   *     *      *     * ************** ****** ***** ******** ******** *** *************** ***
```

31899.10.peg.2908 (Athe_2333) *hemE*

31899.10.peg.2908	Caldicellulosiruptor bescii strain DSMZ 6725	1387557.3.peg.2765	Caldicellulosiruptor sp. Wai35.B1
632348.3.peg.235	Caldicellulosiruptor kronotskyensis 2002	632516.3.peg.952	Caldicellulosiruptor lactoaceticus 6A
632292.3.peg.213	Caldicellulosiruptor hydrothermalis 108	608506.3.peg.2260	Caldicellulosiruptor obsidiansis OB47
1121259.3.peg.1362	Caldicellulosiruptor acetigenus DSM 7040	632518.3.peg.2208	Caldicellulosiruptor owensensis OL
632335.8.peg.2560	Caldicellulosiruptor kristjanssonii 177R1B		

```
31899.10.peg.2908  CATGATTGGGGCTATTAAAGAGTAAtatttgtggtataataaaatAaacttaaacaaaagaaaagcggggaaaatacaatATGAACAAAGA
608506.3.peg.2260  CATGATTGGGGCTATCAAAGAGTAAtaatttgtgatataattaaataaacttgaacaaaagagaagtggtgtaaaatcaacATGAAAAAAGA
632518.3.peg.2208  CATGATTGGGGCTATCAAAGAGTAAtaatttgtgatataattaaataaacttaaacaaaagagaagtggtgtaaaatcaacATGAAAAAAGA
1121259.3.peg.1362 CATGATTGGGGCTATTAAAGAGTAAtaatttgtggtataattaaataaacttaaacaaaagaaaagtggtgcaaaatcaatATGAAAAAAGA
632516.3.peg.952   CATGATTGGGGCTATTAAAGAGTAAtaatttgtggtataattaaataaacttaaacaaaagaaaagtggtgcaaaatcaatATGAAAAAAGA
632335.8.peg.2560  TATGATTGGGGCTATTAAAGAGTAGtaatttgtgatataataaaataaatctaaacaaaagaaaagtggtgcaaaatcaatATGAAAAAAGA
632292.3.peg.213   CATGATTGGGGCTATTAAAGAGTAAtaatttgtggtataattaaataaacttaaacaaaagaaaagtggtgcaaaatcaatATGAAAAAAGA
1387557.3.peg.2765 CATGATTGGGGCTATTAAAGAATAAtagtttgtggtataattaaataaacttaaacaaaagaaaagtggggcaaaatcaatATGAAAAAAGA
632348.3.peg.235   CATGATTGGAGCTATTAAAGAGTAAtatttgtggtataataaaataaacttaaacaaaagaaaagcggggaaaatacaatATGAACAAAGA
                    ******** ***** ***** ** ** ****** ****** *******   * ********* *** ** * ***   *** ***** *****
```

31899.10.peg.2910 (Athe_2335) *hemGD* Predicted UxaR-binding site (potential repressor)
weak XynR-binding site (not conserved in C. bescii) (repressor)

31899.10.peg.2910	Caldicellulosiruptor bescii strain DSMZ 6725	1214564.3.peg.888	Caldicellulosiruptor sp. F32
632348.3.peg.229	Caldicellulosiruptor kronotskyensis 2002	632518.3.peg.2210	Caldicellulosiruptor owensensis OL
632292.3.peg.211	Caldicellulosiruptor hydrothermalis 108	608506.3.peg.2263	Caldicellulosiruptor obsidiansis OB47
1121259.3.peg.1364	Caldicellulosiruptor acetigenus DSM 7040	632516.3.peg.950	Caldicellulosiruptor lactoaceticus 6A
1222016.3.peg.2597	Caldicellulosiruptor changbaiensis strain CBS-Z	351627.8.peg.690	Caldicellulosiruptor saccharolyticus DSM 8903
632335.8.peg.2562	Caldicellulosiruptor kristjanssonii 177R1B		

```
31899.10.peg.2910  ctggcataattttgccagcttatttttttttggttttattgttgaaaatgaaaatcaataggtgtaaaatatatttaaaaacacataaaaacaaaaaa-tc
1222016.3.peg.2597 ccggca-aattttgctggcttatttttttt--gcttt---gttgaaaattgaaactaataggtgtaaaatatatttaaaaacacacaaaagcaaaaaa-tt
1214564.3.peg.888  ccggca-a-ttttgccggtttatttttttt--gcttt---gttgaaaattgaaactaataggtgtaaaatatatttaaaaacacacaaaagcaaaaaa-tt
632348.3.peg.229   ctggcgtaattatgccagcttatttttttttggttttattgttgaaaataaaaattaatagttgtaaaatatatttaaaaacacataaaaacaaaaa--tc
632335.8.peg.2562  ctggcataattttgccagctaattttttt-ggttttattgttgaaaataaaaaacaataggtgtaaaatatatttaaaaacacataaaaacaaagaa-at
632516.3.peg.950   ctggcataattttgccagcttatttttt--ggttttattgttgaaaatgaaaaacaataggtgtaaaatatatttaaaaacacataaaaacaaaaaa-tt
1121259.3.peg.1364 ctggcataattttgccagctaattttttt-ggttttattgttgaaaatgaaaaacaataggtgtaaaatatatttaaaaacacataaaaacaaagaa-at
632292.3.peg.211   ctggcataattttgccagcttattttttt-ggttttattgttgaaaatgaaaatcaataggtgtaaaatatatttaaaaacacataaaaacaaaaaa-at
632518.3.peg.2210  ctggcataattatgccagcttatttttat-ggttttgttattgaaaataagagatggtagatataaaatatatttaaaaacacacaaaaacaaaaaattt
608506.3.peg.2263  ctggcataattttgccagcttatttttat-ggttttgttgttgaaaataagagatgatagatataaaatatatttaaaaacacacaaaaacaaaaaattt
351627.8.peg.690   tctctacacctattcgag---atttttttttggttttattgttgaaaatgaaaatcaataagtgtaaaatatatttaaaaacacataaaaacaaaaaa-tt
                          *  * * *   *   ******    * ***    ********   *     **  * ********************* **** **** *

31899.10.peg.2910  tttcttaatcacaattttttatagttttttaaa-gttaacttttttgttatggaattatgtcgaatggaatttgaacaaggggtttgatgagataATGTAT
1222016.3.peg.2597 tttcttaatcccaatttttta-accgttttcatcttggcattttagttgggatttgtgcaaaatggaatttaaacaagggggcgcagt-agaagATGTAT
1214564.3.peg.888  tttcttaatcccaatttttta-accgttttcattttgacattttagttgggatttgtgcaaaatggaatttaaacaagggggcgcaaa-agaaaGTGTAT
632348.3.peg.229   tttcttaatcacaattttttatagttttttaaagttaacttttttgttatggaattatgtcgaatggaatttgaacaaggggtttgatgagataATGTAT
632335.8.peg.2562  tttcttaatcccaattttataaccgttttcaaat-tgactttttactttggatttgtgcaaagtggaatttgaacaaggggtatggtgagaagATGTAC
632516.3.peg.950   tttcttaatcccaattttataaccgttttcaaat-tgactttttactttggatttgtgcaaagtggaatttgaacaaggggtatggtgagaagATGTAC
1121259.3.peg.1364 tttcttaatcccaattttataaccgttttcaaat-tgactttttactttggatttgtgtaaaatggaatttgaacaaggaggtatggtgagaagATGTAT
632292.3.peg.211   tttcttaatcccaatttttttaaccgttttcaaat-tgactttttactttggatttgtgtcgaatggaatttgaacaaggggtatggtgagaagATGTAT
632518.3.peg.2210  tttcttaatccctatttctaaatcgttttcaaat-tgactttttgctttggatttgtacaaaatagaatttgaacaaggaggtatggtgagaggATGTAT
608506.3.peg.2263  tttcttaatccctatttctaaatcgttttcaaat-tgactttttactttggatttgtacaaaatggaatttgaacaaggaggtatggtgggaggATGTAT
351627.8.peg.690   tttcttaatcccaattttttaaccgttttcatct-tgacattttagttggaatttgtgcaaaatggaatttgaataaggaggcgcaaa-agaaaGTGTAT
                   ********** * ****   * *   **** *  **  * ****  *  * * ** *  * * ****** ** **** **        **   *
```

31899.10.peg.812 (Athe_0397) ***pgmB-kojP*** **KojR -binding site (repressor)**

31899.10.peg.812	Caldicellulosiruptor bescii strain DSMZ 6725	632335.8.peg.387	Caldicellulosiruptor kristjanssonii 177R1B
632348.3.peg.2355	Caldicellulosiruptor kronotskyensis 2002	608506.3.peg.399	Caldicellulosiruptor obsidiansis OB47
1121259.3.peg.2529	Caldicellulosiruptor acetigenus DSM 7040	632292.3.peg.2362	Caldicellulosiruptor hydrothermalis 108
632516.3.peg.2194	Caldicellulosiruptor lactoaceticus 6A	351627.8.peg.509	Caldicellulosiruptor saccharolyticus DSM 8903
1387557.3.peg.616	Caldicellulosiruptor sp. Wai35.B1	1222016.3.peg.460	Caldicellulosiruptor changbaiensis strain CBS-Z

```
31899.10.peg.812   aaaaa-cgaaaataatcgaaaacgatattgaaaacaattcgaaaaacttttataataatcttAtacaacaatcacagcaaaggggtttact-tttcATGG
1121259.3.peg.2529 aaaaatcgaaaataatcgaaaacgatattgaaaacaattcgaaaaccatttataataatcttatacgacaatctcagcaaaggggtttgct-tttcATGG
632516.3.peg.2194  aaaaatcgaaaataatcgaaaacgatattgaaaacaattcgaaaaccatttataataatcttatacgacaatctcagcaaaggggtttgct-tttcATGG
632335.8.peg.387   aaaaatcgaaaataatcgaaaacgatattgaaaacaattcgaaaaccatttataataatcttatacgacaatctcagcaaaggggtttgct-tttcATGG
1387557.3.peg.616  gaaaaacgaaaataaacgaaaacgatattgaaaacaattcgaaaactatttataatagtcttataaaacaatatcagcaaaggggtttgct-tttcATGG
632348.3.peg.2355  aaatgtcgaaaataatcgaaaacgatattgaaaacaattcgaaaaacttttataataatcttatacaacaatcacagcaaaggggtttact-tttcATGG
608506.3.peg.399   ggaaatcgaaaataaacgaaaacgatattgaaaacaattcgaaaaccatttataatagtcttataccacaatttcagcaaaggggtttgct-tttcATGG
632292.3.peg.2362  aaaaatcgaaaataatcgaaaacgatattgaaaacaattcgaaaaacttttataataatcttatatcacaatctcagcaaaggggtttgct-tttcATGG
351627.8.peg.509   attcttcgaaaataaccgaaaacgatattgaaaacaattcgaaaaaattttataatagttgtataaaactattttagtaaaggggcctgctattttATGG
1222016.3.peg.460  cctcttcgaaaataatcgaaaacaatattgaaaacaattcgaaaaaaatttataataactttaggagacaactttagtaaaggggcttgctactttATGG
                         ********* ******* *********************   *********    **    ** *    ** *******  * **  ** ****
```

31899.10.peg.817 (Athe_0402) ***kojR***

31899.10.peg.817	Caldicellulosiruptor bescii strain DSMZ 6725	632292.3.peg.2357	Caldicellulosiruptor hydrothermalis 108
632335.8.peg.393	Caldicellulosiruptor kristjanssonii 177R1B	608506.3.peg.405	Caldicellulosiruptor obsidiansis OB47
632516.3.peg.2199	Caldicellulosiruptor lactoaceticus 6A	1222016.3.peg.465	Caldicellulosiruptor changbaiensis strain CBS-Z
632348.3.peg.2349	Caldicellulosiruptor kronotskyensis 2002	1387569.3.peg.2742	Thermoanaerobacter cellulolyticus NA10
1387557.3.peg.621	Caldicellulosiruptor sp. Wai35.B1	351627.8.peg.514	Caldicellulosiruptor saccharolyticus DSM 8903
1121259.3.peg.2534	Caldicellulosiruptor acetigenus DSM 7040		

```
31899.10.peg.817   agtaataaaaatgttgaaacagatggcaaagaaagtgtataattattttCaaggttttagtgaaacatttcaaaaataagagggagacacga-aaaATG
632516.3.peg.2199  aataacaaaagtattgaaacagaagccaaagaaagtgtataattaatttcaaggttttagtgaaacatttcaaaaataagagggagaaacgagaaaATG
1121259.3.peg.2534 aataacaaaagtattgaaacagaaggcaaagaaagtgtataattaatttcaaggttttagtgaaacatttcaaaaataagagggagaaacgagaaaATG
632348.3.peg.2349  agtaataaaaatgttgaaacagaagacaaagaaagtgtataattaatttcaaggttttagtgaaatatttcaaaaaca-gaaggtgaaaagaaagaATG
632292.3.peg.2357  agtaataaaaatgttgaaacagaaggcaaagaaagtgtataattaatttcaaggttttagtgaaacatttcaaaaaacagagggagaaaagaaagaATG
632335.8.peg.393   agtaataaaaatgttgaaatagaaggcaaagaaagtgtataattaatttcaaggttttagtgaaacatttcaaaaaacagagggagaaacgtgagaATG
1387557.3.peg.621  agtaataaaaatgttgaaacagaaggcaaagaaagtgtataattattttcaaggttttagtgaaacatttcaaaaaacagagggagaaacgtgagaATG
608506.3.peg.405   agtaataaaaatgttgaaaaataaggcaaagaaagtgtataattaatttcaaggttttagtaaaacatttcaaaaaacagagggagaaacggaaaaATG
1222016.3.peg.465  agtaacaaagatattgaatttatcaagaagaacagtgtataatttattttaaggttttagtaaaacatttcaaaaa-cagagggagaaaa---gtaATG
351627.8.peg.514   agtaacaaagatattgaatttatcgagaagaacagtgtataatttattttaaagttttagtaaaacatttcaaaaa-cagagggagaaaa---ataATG
1387569.3.peg.2742 agtaattatgatattgaatttaccaaggaagagagtgtataattaatttcaaggttttagcaaaatatttcaaaaa-cagagggagaaaat--ataATG
                   * ***  *   * *****          *  * ***********  *** ** *******  *** **********   ** ** ** *      ****
```

31899.10.peg.814 (Athe_0399) *kojEFG* KojR -binding sites (repressor)

31899.10.peg.814	Caldicellulosiruptor bescii strain DSMZ 6725	632335.8.peg.389	Caldicellulosiruptor kristjanssonii 177R1B
632348.3.peg.2353	Caldicellulosiruptor kronotskyensis 2002	632292.3.peg.2360	Caldicellulosiruptor hydrothermalis 108
1121259.3.peg.2531	Caldicellulosiruptor acetigenus DSM 7040	608506.3.peg.401	Caldicellulosiruptor obsidiansis OB47
632516.3.peg.2196	Caldicellulosiruptor lactoaceticus 6A	351627.8.peg.511	Caldicellulosiruptor saccharolyticus DSM 8903

```
31899.10.peg.814   cagggagtttgattttacgaaaacatttcgaaaaaagtttgaaagcttgaaatatataagacagcatt-acttatacttaataaacaaaatctaa
632348.3.peg.2353  cagctcctttgattttacgaaaacatttcgaaaaaagtttgaaagcttgaaatatataagacagcatt-acttatacttaataaacaaaatctaa
1121259.3.peg.2531 cagggagtttaatttcacgaaaagatttcgaaaaaagtttgaaagcttggaccatatgaaacagcatt-gcttatgcttaataaacaaaattcga
632516.3.peg.2196  cagggagtttaatttcacgaaaagatttcgaaaaaagtttgaaagcttggaccatatgaaacagcatt-gcttatgcttaatatacaaaattcga
632335.8.peg.389   cagggagtttaatttcacgaaaagatttcgaaaaaagtttgaaagcttggaccatatgaaacagcatt-gcttatgcttaataaacaaaattcga
608506.3.peg.401   cagggagtttagtttcacgaaaagatttcgaaaaaagtttggaagtttggagaatatgaaacagcatt-gattatacctaataaacaaaatttaa
632292.3.peg.2360  cagcgagttttattttacgaaaagatttcgaaaaa-gtttgatagtaaggagaacatgaagcatcctttacttatgcttaataaacaaaattcaa
351627.8.peg.511   caggggatttgtttttcgaaaaaagtttcgatgaaggtt-aaagctgtcttggaactgtagcaaaata-gtttataatatataaacaaaaaacaa
                   ***    ***  ***    ****  ******  ** ***              *  *    **   *    ****     *** ******    *

31899.10.peg.814   aaattaattaaaaaacctatagacaaaattactaacttgttgtataataaaatcAtcgaaaacatttccgaaa-tatttaattcaa---aaagg
632348.3.peg.2353  aaattaattaaaaaacctgtagacaaaattactaacttgttgtataataaaatcatcgaaaacatttccgaaa-tatttaattcaa---aaagg
1121259.3.peg.2531 aaatcaaataaaaaacctatagacaaaattactaacttgttgtataataaaattatcgaaaacatttccgaaa-tatttaattcaa---aaggg
632516.3.peg.2196  aaatcaaataaaaaacctatagacaaaattactaacttgttgtataataaaattatcgaaaacatttccgaaa-tatttaattcaa---aaggg
632335.8.peg.389   aaatcaaataaaaaacctatagacaaaattactaacttgttgtataataaaattatcgaaaacatttccgaaa-tatttaattcaa---aaagg
608506.3.peg.401   aaattaaattaaaaacctatagacaagattactaacttgttgtataataaaatcatcgaaaacatttccgaaa-tatttaattcaa---aaagg
632292.3.peg.2360  aaattaattaaaaaacctatagacaaaattactaacttgttgtataataaaattatcgaaaacatttccgaaa-tatttaattcaa---aaagg
351627.8.peg.511   aaattttttaagataagtatggacaaaaataataacttgttgtataataaaattgtcgaaaacatttccgaaaatatttaagtaatctcaaagg
                   ****    * * * *  * * ***** * ** *********************  ****************** ******* * *    ** **

31899.10.peg.814   aggtttg-tttgATGAAAAAATTTCTAACAATTTTATTAACGTTAATTTTCCTACT
632348.3.peg.2353  aggtttg-ttttATGAAAAAATTTCTAACAATTTTATTAACGTTAATTTTCCTACT
1121259.3.peg.2531 aggtttg-ttttATGAAAAAATTTCTAACAGTTTTATTAACGCTAATTTTCCTACT
632516.3.peg.2196  aggtttg-ttttATGAAAAAATTTCTAACAATTTTATTAACGCTAATTTTCCTACT
632335.8.peg.389   aggtttg-ttttATGAAAAAATTTCTAACAATTTTATTAACGCTAATTTTCCTACT
608506.3.peg.401   aggtttg-ttttATGAAAAAATTTCTAACAATTTTATTAACACTAATTTTTCTACT
632292.3.peg.2360  aggtttg-ttttATGAAAAAATTTCTAACAATTTTATTAACGCTAATTTTCCTACT
351627.8.peg.511   aggtttgattctATGAAAAGGTTGTTAGCAGGTTTACTTATTTTAGTATTTGTTAT
                   ******* **  *******  **  ** **  **** * *   ** * **   *  *
```

31899.10.peg.814 (Athe_0403) *treP* KojR -binding site (potential activator)

31899.10.peg.818 Caldicellulosiruptor bescii strain DSMZ 6725
632348.3.peg.2348 Caldicellulosiruptor kronotskyensis 2002
608506.3.peg.406 Caldicellulosiruptor obsidiansis OB47
632292.3.peg.2356 Caldicellulosiruptor hydrothermalis 108
632335.8.peg.394 Caldicellulosiruptor kristjanssonii 177R1B
1121259.3.peg.2535 Caldicellulosiruptor acetigenus DSM 7040
1387557.3.peg.622 Caldicellulosiruptor sp. Wai35.B1
1222016.3.peg.466 Caldicellulosiruptor changbaiensis strain CBS-Z
1387569.3.peg.882 Thermoanaerobacter cellulolyticus NA10

```
31899.10.peg.818   cttacaagcaagaatattttttctgt----gctttatttgaacataa--ttttaccgaaaatatttcggtaacattcccaaaacatatttaaaaag
632348.3.peg.2348  ctcacaagcaagaatattttttctgt----gctttatttgaacataa--ttttaccgaaaatatttcggtaacattcccaaaacatatttaaaaag
1387569.3.peg.882  ctcacaagcaagaatattttttctgt----gctttatttgaatgtaa--ttttaccgaaaatatttcggtagcattcccaaaacatatttaaaaag
632335.8.peg.394   ctcacaaacaagaatattctctctgt----gtttatttgaatctaa--ttttatcgaaaatatttcggtaacatttccaaaacatatttaaaaag
1121259.3.peg.2535 ctcacaaacaagaatattctatctgt----gctttatttgaatctaa--ctttatcgaaaatatttcggtaacattcccaaaacatatttaaaaag
608506.3.peg.406   ctcacaaacaagaatattctctctgt----aatttatttgaacataa--tgttaccgaaaatatttcggtaacattcccaaaacatatttaaaaag
1222016.3.peg.466  ctcacaaacaagaatattctctctgt----gctttatttgaacccaa--ttttatcgaaaatatttcggtaacattcccaaaacatatttaaaaag
1387557.3.peg.622  gtaaaactcatttcttttttacccatcaaattctctttaaacataaattt-tatcgaaaatattccggtaacattcccaaaacatatttaaaaag
632292.3.peg.2356  tttgacttcttttatttttttacccatcaaatttacaattaaacacaaaattataccgaaaatatttcggtaacattcccaaaacatatttaaaaag
                          *      *     * ** *      *       **          ** **************** **** *******************

31899.10.peg.818   taacttccagccattgaactgtaaattatattaaggtatgattacGtaaaaaagattttgctgcgaaaggagaatacagcTTGAG
632348.3.peg.2348  taacttccagccattgaactgtaaattatattaaggtatgattacataaaaaagattttgctgcgaaaggagaatacagcTTGAG
1387569.3.peg.882  taacttccagctatttaactgtaaattataataaggtatgattacataaaaaagattttgctgcgaaaggagaatccagcTTGAG
632335.8.peg.394   taacttccagccattgaactgtaaattataataaggtatgattacataaaaaagattttgctgcgaaaggagaatacagcTTGAG
1121259.3.peg.2535 taacttccagccattgaactgtaaattataataaggtatgattacataaaaaagattttgctgcgaaaggagaatacagcTTGAG
608506.3.peg.406   taacttccagccattgaactgtaaattataataaggtatgattacgtaaaaaagattttgcagcgaaaggagaatacagtTTGAG
1222016.3.peg.466  taacttccagccattgaactgtaaattatattaaggtatgattacataaaaaagattttgctgcgaaaggagaattcagcTTGAG
1387557.3.peg.622  taacttccagccattgaactgtaaattataataaggtatgattacataaaaaagattttttctgcgaaaggagaatacatcTTGAG
632292.3.peg.2356  taacttccagccattgaactgtaaattataataaggtatgattacgtaaaaaagattttgctgcgaaaggagaatacagcTTGAG
                   ***** *** * *** **** * ******* ************** ************* * ************* **  *****
```

31899.10.peg.126 (Athe_2579) *nplT* MalR -binding site (potential repressor)

31899.10.peg.126	Caldicellulosiruptor bescii strain DSMZ 6725	632516.3.peg.554	Caldicellulosiruptor lactoaceticus 6A
1214564.3.peg.74	Caldicellulosiruptor sp. F32	1222016.3.peg.447	Caldicellulosiruptor changbaiensis strain CBS-Z
351627.8.peg.495	Caldicellulosiruptor saccharolyticus DSM 8903	632518.3.peg.2312	Caldicellulosiruptor owensensis OL
632292.3.peg.292	Caldicellulosiruptor hydrothermalis 108	632348.3.peg.301	Caldicellulosiruptor kronotskyensis 2002
632335.8.peg.148	Caldicellulosiruptor kristjanssonii 177R1B	608506.3.peg.2361	Caldicellulosiruptor obsidiansis OB47
1121259.3.peg.1294	Caldicellulosiruptor acetigenus DSM 7040	1387569.3.peg.2726	Thermoanaerobacter cellulolyticus NA10

```
31899.10.peg.126   ctttataagtgc-ctacaaaacaaat-ttatgcaaacatttgcattatagatagaaaagtgataaaa
632348.3.peg.301   ctttataagtgc-ctacaaaacaaat-ttatgcaaacatttgcattatagatagaaaagtgataaaa
1214564.3.peg.74   ctttataagtac-ctacaaaacaaat-ttatgcaaacatttgcattatagatagaaaagtgataaaa
351627.8.peg.495   ctttataagtac-ctacaaaacaaat-ttatgcaaacatttgcattatagatagaaaagtgataaaa
632518.3.peg.2312  ctttatgagtac-ctacaaaacaaat-ttgtgcaaacatttgcattatagatagaaaagtgataaaa
632335.8.peg.148   gcttgaaagtgcacttcaaaaaaa-ccttgtgcaaacatttgcattatagatagaaaagtgataaaa
632516.3.peg.554   gcttgaaagtgcacttcaaaaaaaaccttgtgcaaacatttgcattatagatagaaaagtgataaaa
1121259.3.peg.1294 gcttgaaagtgcacttcaaaaaaa-ccttgtgcaaacatttgcattatagatagaaaagtgataaaa
632292.3.peg.292   gcttgaaagtgt-cctcaaaaaaaatcttgtgcaaacatttgcattatagatagaaaagtgataaaa
1222016.3.peg.447  gcttgaaagtgt-ccttaaaaaaa-tcttgtgcaaacatttgcattatagatagaaaagtgataaaa
608506.3.peg.2361  gcttgaaaatgttatttataaaaa-tcttgtgcaaacatttgcattacagatagaaaagtgataaaa
1387569.3.peg.2726 cctataaagttctacttttaa-agcttttgtgcaaacatttgcattattcattgaaaagtggtaaaa
                     *    * *         ** *    ** *****************   ** ******** *****
```

```
31899.10.peg.126   taaatttcAatgttgtatttcaaaaaa--tatttccgaaggagagtgcaa--aatTTGCAGGTTATC
632348.3.peg.301   taaatttcaatgttgtatttcaaaaaa--tatttcagaaggagagtgcaa--aatTTGCAGGTTATT
1214564.3.peg.74   taaatttcaatgttgtatttcaaaaaaaatatttcagaaggagagtgcaa--agtTTGCAGGTTATT
351627.8.peg.495   taaatttcaatgttgtatttcaaaaaaaatatttcagaaggagagtgcaa--agtTTGCAGGTTATT
632518.3.peg.2312  taaatttcaatgttgtatttcaaaaaa--tatttcaaaaggagagtgtaagaagcTTGCAAGTTATT
632335.8.peg.148   taaatttcaatgttatatttcaaaaaaa-tatttcagaaggagagtgcac--agtTTGCAAGTCATT
632516.3.peg.554   taaatttcaatgttatatttcaaaaaaa-tatttcagaaggagagtgcac--agtTTGCAAGTCATT
1121259.3.peg.1294 taaatttcaatgttatatttcaaaaaaa-tatttcagaaggagagtgcac--agtTTGCAAGTCATT
632292.3.peg.292   taaatttcaatgttgtatttcaaaaaa--tatttcagaaggagagtgcac--agtTTGCAGGTTATT
1222016.3.peg.447  taaatttcaatgttgtatttcaaaaaa--tatttcaaaaggagagtgtaagaagcTTGCAGGTTATT
608506.3.peg.2361  taaatttcaatattgtatttcaaaaaa--tatttcaaaaggagagtgcac--ggtTTGCAGGTTATT
1387569.3.peg.2726 taaaactcaatcatggtatttaaaagat---tttataaaggagtgtgcagaaactTTGGAAATTATT
                   ****  *****  *    ** **** *    ***   ****** *** *      *** *  * **
```

31899.10.peg.125 (Athe_2578) *malFG-glgP-malR* MalR-binding site (repressor)

31899.10.peg.125	Caldicellulosiruptor bescii strain DSMZ 6725	1121259.3.peg.1293	Caldicellulosiruptor acetigenus DSM 7040
632348.3.peg.302	Caldicellulosiruptor kronotskyensis 2002	1387557.3.peg.17	Caldicellulosiruptor sp. Wai35.B1
632292.3.peg.293	Caldicellulosiruptor hydrothermalis 108	632335.8.peg.149	Caldicellulosiruptor kristjanssonii 177R1B
351627.8.peg.496	Caldicellulosiruptor saccharolyticus DSM 8903	632518.3.peg.2311	Caldicellulosiruptor owensensis OL
632516.3.peg.555	Caldicellulosiruptor lactoaceticus 6A	1222016.3.peg.448	Caldicellulosiruptor changbaiensis strain CBS-Z

```
31899.10.peg.125   ctaaaaaa--tgttgacacgcaagaaagaggattgtataatattctCaagagcaaacgattgcacattct--atttcaaaggaggaaaaaa-attATG
351627.8.peg.496   ctaaaaaaa-tgttgacacgcaagcaagaggattgtataatattttcaagagcaaacgattgcacacact--attttaaaggaggaaaaaagattATG
1387557.3.peg.17   gcaaaaaat-tattgacaaacgaaaaatacaattgtataatattttcaagagcaaacgattgcacatttt--attttaaaggaggaaaaaagattATG
632348.3.peg.302   ataaaaatt-tgttgacaggaataaaagatgattgtataatattttcaagagcaaacgattgcacatttt--attttaaaggaggaaaaag-attATG
632292.3.peg.293   ctaaaaaaa-tgttgacacgcaagaaagataattatataatattttcaagagcaaacgattgcacattct--atttcaaaggaggaaaaag-attATG
632516.3.peg.555   agaaaaagt-tattgacgtgaaagaaaaatgattgtataatatcctcaagagcaaacgattgcacattttt-attttagaaggaggaaaaagattATG
1121259.3.peg.1293 agaaaaagt-tattgacgtgaaagaaaaatgattgtataatatcctcaagagcaaacgattgcacattttt-attttagaaggaggaaaaagattATG
632335.8.peg.149   agaaaaagt-tattgacgtgaaagaaaaatgattgtataatattctcaagagcaaacgattgcacattttt-atttcagaaggaggaaaaagattATG
1222016.3.peg.448  actaaaaaa-tattgacacgcaagtaagtcaattgtataatatttttaagagcaaacgattgcacattttt-atttcagaaggaggaaaaagattATG
632518.3.peg.2311  ttaaaaaaa-tattgacacgcaaggaagaggattgtataatattcttgagagcaaacgattgcacatttttattttaaaggaggaaaaag-attATG
608506.3.peg.2360  ttaaaaaaagtattgacatgcacaaaagacaattatataatattctcaagagcaaacgattgcacactttt-attttaaaggaggaaaaag-attATG
                      ****   * *****         **    *** ********   *  ******************    *  **** * * *  * ****   ******
```

31899.10.peg.121 (Athe_2574) *malE* MalR-binding site (repressor)

31899.10.peg.121	Caldicellulosiruptor bescii strain DSMZ 6725	632335.8.peg.153	Caldicellulosiruptor kristjanssonii 177R1B
632292.3.peg.297	Caldicellulosiruptor hydrothermalis 108	632518.3.peg.2307	Caldicellulosiruptor owensensis OL
1121259.3.peg.1289	Caldicellulosiruptor acetigenus DSM 7040	608506.3.peg.2356	Caldicellulosiruptor obsidiansis OB47
1222016.3.peg.452	Caldicellulosiruptor changbaiensis strain CBS-Z	632348.3.peg.306	Caldicellulosiruptor kronotskyensis 2002
632516.3.peg.559	Caldicellulosiruptor lactoaceticus 6A	351627.8.peg.500	Caldicellulosiruptor saccharolyticus DSM 8903

```
31899.10.peg.121   tctctctcttgacttttttagagagaaatatataatgttattAtcaaacgtttgcatatt----aaataaacttaagggaggttgttttgattATG
632348.3.peg.306   tctctctcttgacttttttagagagaaatatataatgttattatcaaacgtttgcatatc----aaataaatttaagggaggttgttttgattATG
632518.3.peg.2307  tctttctcttgacttttttagaaagaaatatataatgttattatcaaacgtttgcacatt----aaataaacttaagggaggttgttttgattATG
608506.3.peg.2356  tctctctcttgactttttt-agagagaaatatataatgttattatcaaacgtttgcacattt---aaataaacttaagggaggttgttttgattATG
632516.3.peg.559   tctctctcttgacttttttagagagaaatatataatgttattatcaaacgtttgcatgtt----aaataaacttaagggaggttgtgttgattATG
632335.8.peg.153   tctctctcttgacttttttagagagaaatatataatgttattatcaaacgtttgcatgtt----aaataaacttaagggaggttgtgttgattATG
1121259.3.peg.1289 tctctctcttgacttttttagagagaaatatataatgttattatcaaacgtttgcacatt----aattaaagttaagggaggttgttttgattATG
632292.3.peg.297   tctctctcttgacttttttagagagaaatgtataatgttattatcaaacgtttgcatgta----aaataaacttaagggaggttgttttgattATG
1222016.3.peg.452  tctttctcttgacttttttagagagaaatgtataatgttattatcaaacgtttgcactct----aaataagcttaagggaggttgttttgattATG
351627.8.peg.500   -ctttctcttgacttttttagagagaaatgtataatgttattatcaaacgtttgcatattgaataaacaaatttaagggaggttgttttgattATG
                    ** *************** *** ****** **************************        **  **  ************** *********
```

31899.10.peg.538 (Athe_0143) *amyA* MalR-binding site (potential repressor)

31899.10.peg.538 Caldicellulosiruptor bescii strain DSMZ 6725
632292.3.peg.2603 Caldicellulosiruptor hydrothermalis 108
632348.3.peg.2556 Caldicellulosiruptor kronotskyensis 2002
1387569.3.peg.66 Thermoanaerobacter cellulolyticus NA10
632335.8.peg.167 Caldicellulosiruptor kristjanssonii 177R1B
1121259.3.peg.559 Caldicellulosiruptor acetigenus DSM 7040
1387557.3.peg.412 Caldicellulosiruptor sp. Wai35.B1
608506.3.peg.154 Caldicellulosiruptor obsidiansis OB47
632518.3.peg.90 Caldicellulosiruptor owensensis OL

```
31899.10.peg.538  agaaaa-gacactacaaaatttgagttttgtatgctaaaataat-----atagattgcaaacgtttgctcaaaat-aagctat-
632292.3.peg.2603 agaaaaagacactacaaaatttgagttttgtatgctaaaataatttcatatagattgcaaacgtttgctcaaaat-aagctat-
1387569.3.peg.66  agaaaaagacactacaaaatttgagttttgtatgctaaaataatttcatatagattgcaaacgtttgcccaaaat-aagctat-
632348.3.peg.2556 agaaaaagccactacaaaatttgagttttgtatggtaaaataatttcatacaaattgcaaacgtttgcttataattaagctata
632335.8.peg.167  agaaaaaaccactacaaattttgaattttgtatgctaaaataatttcgtatagattgcaaacgtttgctcaaaat-aagttga-
1121259.3.peg.559 agaaaaaaccactacaaattttgaattttgtatgctaaaataatttcatatagattgcaaacgtttgcttaaaat-aagttga-
1387557.3.peg.412 agaaaaagccactacaaattttgaggtttgtatgctaaaataatttcatacagactgcaaacgtttgcccaaagt-aaattga-
608506.3.peg.154  agaaaaagccactacaaattttaaattttgtatgctaaaataatttcatataaattgcaaacgtttgctcaaaat-aagctat-
632518.3.peg.90   ---aaaagccactacaaattttaaattttgtatgataaaataatcatgtacatattgcaaacgtttgctttaagt-aagcttt-
                     ***   ********* *** *  ******** *********     * * * *************    * * **  *

31899.10.peg.538  -gggtataaattaattA---gaacaaattgaaaaaggggagaaataagaaagATGTCAGCTTTAAAAAAGCTT
632292.3.peg.2603 -gggtataaattaatta---gaacaaattgaaaaaggggagaaataagaaagATGTCAGCTTTAAAAAAGCTT
1387569.3.peg.66  -gggtataaattaatta---gaacaaattgaaaaaggggagaaacaagaaagATGTCAGTTTTAGAAAAACTT
632348.3.peg.2556 tgggtataaattaaatgtaggaaaaattcaaaaaaggggagaaataagaaagATGTCAGCTTTAAAGAAGCTT
632335.8.peg.167  -gggtataaattta-tt-agaagcaaaatgaaaaaggggagaaataagaaaaATGTCAGTTTTAAAAAAGCTT
1121259.3.peg.559 -gggtataaattta-tc-agaagcaaaatgaaaaaggggagaaataagaaaaATGTCAGCTTTAAAAAAGCTT
1387557.3.peg.412 -gggtataaatgtaatt-agaagcaaaatgaaaaaggggagaaataaaaaagATGTCAGCATTAAAAAAGCTT
608506.3.peg.154  -gggtataaattaa-tt-agaagcaaactgaaaaaggggagaaataagaaagATGTCAGCATTAAAAGAGCTT
632518.3.peg.90   -gggtataaattaatta---gaaacaactgaaaaaggggagaaacagggaagATGTCAACATTAAGCAGGCTT
                   **********  * *     *   *    ************** *   ** ******   ***      ***
```

31899.10.peg.627 (Athe_0228) *cga* MalR-binding site (repressor)

31899.10.peg.627	Caldicellulosiruptor bescii strain DSMZ 6725
632348.3.peg.2474	Caldicellulosiruptor kronotskyensis 2002
632292.3.peg.2487	Caldicellulosiruptor hydrothermalis 108
1222016.3.peg.168	Caldicellulosiruptor changbaiensis strain CBS-Z
1214564.3.peg.1943	Caldicellulosiruptor sp. F32
632516.3.peg.1656	Caldicellulosiruptor lactoaceticus 6A
1121259.3.peg.447	Caldicellulosiruptor acetigenus DSM 7040
608506.3.peg.245	Caldicellulosiruptor obsidiansis OB47
632335.8.peg.273	Caldicellulosiruptor kristjanssonii 177R1B
1387569.3.peg.162	Thermoanaerobacter cellulolyticus NA10
632518.3.peg.181	Caldicellulosiruptor owensensis OL

```
31899.10.peg.627   g--aaa--attaaaattagtaaaattgctgaatgcacaactagtcagatattt-gatataataaaatTactgcaaatgtttgcgtaaaaa
1222016.3.peg.168  g--aaa--attaaaattagtaaaattgctgaatgcacaacaaatcagatattt-gatataatatgattactgcaaatgtttgcgcaaaaa
632335.8.peg.273   g--aaa--attaaaattagtaaaattgctgaatgcacaacaagtcagatattt-gatataatatgattactgcaaatgtttgcgcaaaaa
632348.3.peg.2474  g--agagaataaaaattagtaaaattgctgaatgcacaaatgttgtaatatct-gatataataaaatcactgcaaatgtttgcgcaacaa
608506.3.peg.245   agggaaaaataaaaattggtaaaattgttgaatgca-aagccgagtaatattttgatataataaaactgttgcaaatgtttgcacaacaa
632518.3.peg.181   a--gaaaaaataaaattggtaaaattgctgaatccacaaaccacataaaattt-gctataatgaaattgccgcaaaggtttgcatagcaa
1214564.3.peg.1943 g--gcaaaaataaaattagtaaaattgctgaatgcacaacaagtcagatattt-gttataatatgattactgcaaatgtttgcgcaacaa
1387569.3.peg.162  g--gcaaaaataaaattagtaaaattgctgaatgcacaacaagtcagatattt-gatataatatggttactgcaaatgtttgcgcaacaa
632516.3.peg.1656  ag-gcaaaaataaaattagtaaaattgctgaatgcacaacaagtcagatattt-gatataatatgattactgcaaatgtttgtgcaaaaa
1121259.3.peg.447  ag-gcaaaaataaaattagtaaaattgctgaatgcacaacaagtcagatattt-gatataatatgattactgcaaatgtttgcgcaaaaa
632292.3.peg.2487  g--aaaaacaagacaaggcttcagtttgaaaaaatgcagctcatatagtataataactttaattcttcttagcaaacgtttgagttaaaa
                        *      * *    *  * **    **     *           **       * *          ***** *****      **
```

```
31899.10.peg.627   tttaaatat-aagaaagagggatcat-ATGAGAAAGCCACATATTATAGAAGCTATAATT
1222016.3.peg.168  tttaaatat-aagaaagagggatcataTTGAGAAAGCCGCACGTGATAGAAGCTATAATT
632335.8.peg.273   tttaaatat-aagaaagagggatcataTTGAGAAAGCCGCACGTGATAGAAGCTATAATT
632348.3.peg.2474  tttgtatat-aggaaagagggatcat-ATGAGAAAGCCGCACGTAGTAGAAGCTATAATT
608506.3.peg.245   tttgaataagaagaaagagggattataTTGAAGAAAGCACATGTTATAGAAGCCATAATT
632518.3.peg.181   atacggtat-aagaaagagggattataTTGAGAAAACCGCACGTGATAGAAGCTATTATA
1214564.3.peg.1943 tttgtatat-ggggaagagggatcat-ATGAGAAAGCCGCACGTAGTAGAAGCTATAATT
1387569.3.peg.162  tttgtatat-ggaaaagagggatcat-ATGAGAAAGCCGCACGTAGTAGAAGCTATAATT
632516.3.peg.1656  tttaaatat-aagaaagagggatcataTTGAGAAAGCCGCACGTGATAGAAGCTATAATT
1121259.3.peg.447  tttaaatat-aagaaagagggatcataTTGAGAAAGCCGCACGTAGTAGAAGCTATAATT
632292.3.peg.2487  ttaaaacat--ggaaagagggatcat-ATGAGAAAGCCGCACGTGATAGAAGCTATAATA
                    *     *      ********* **  ***  **  * **  *  ******* ** **
```

31899.10.peg.627 (Athe_0609) *pulA-amyX* MalR-binding site (repressor)

31899.10.peg.1045	Caldicellulosiruptor bescii strain DSMZ 6725	632335.8.peg.2159	Caldicellulosiruptor kristjanssonii 177R1B
632348.3.peg.2129	Caldicellulosiruptor kronotskyensis 2002	632516.3.peg.1534	Caldicellulosiruptor lactoaceticus 6A
1387557.3.peg.870	Caldicellulosiruptor sp. Wai35.B1	632518.3.peg.535	Caldicellulosiruptor owensensis OL
351627.8.peg.794	Caldicellulosiruptor saccharolyticus DSM 8903	1222016.3.peg.2457	Caldicellulosiruptor changbaiensis strain CBS-Z
1121259.3.peg.823	Caldicellulosiruptor acetigenus DSM 7040	608506.3.peg.624	Caldicellulosiruptor obsidiansis OB47
632292.3.peg.2140	Caldicellulosiruptor hydrothermalis 108	1387569.3.peg.2093	Thermoanaerobacter cellulolyticus NA10

```
31899.10.peg.1045  ttcaaattgcagcatattgacaattttaataaaaagatatataatcattaCaaagttaaagcaaacctttgcattcacaaaagaggtttttggattttc
632335.8.peg.2159  ttcaaattgcagcatattgacaattttaattaaaagatatataatcattacaaagtaaaggcaaacctttgcattaacaaattgta-tttttgattctt
632516.3.peg.1534  ttcaaattgcagcatattgacaattttaattaaaagatatataatcattacaaagtaaaggcaaacctttgcattaacaaattgta-tttttgattctt
632348.3.peg.2129  ttcaaattgcagcatattgacaatgttaatgaaaagatatataatcattacaaagtaaaagcaaacctttgcattaataaattgta-tttttgattttt
1121259.3.peg.823  ttcaaattgcagcatattgacaattttaataaaaagatatataatcattacaaagttaaagcaaacatttgcattgataaaaggtg-ttttgaacaatc
1387557.3.peg.870  ttcaaattgcagcatattgacaattttaataaaaagatatataatcattacaaagttaaggcaaacatttgcattcacaaaagagacttttgaattttt
632292.3.peg.2140  ttcaaattgcagcatattgacaattttaataaaaagatatataatcattacaaagttaaggcaaacctttgcattcataaaagaca-ttttagagtttc
632518.3.peg.535   ttcaaattgcagcatattgacaattttaataaaaagatatataatcattacaaagttaaggcaaacctttgcattcacaaaagacg-ttttggagtttc
608506.3.peg.624   ttcaaattgcagcatattgacaattttaataaaaagatatataatcattacaaagtttaagcaaacctttgcattcacaaaagaca-ttttggagtttc
351627.8.peg.794   tgcaatctgcagcatattgacaattttcatcaaaattatataatcaaaacaaa-gccaagcaaacatttgcataaacttttaacctcttcaaa-tttt
1387569.3.peg.2093 tacaatctgcagcatattgacaattttcatcaaaattatataatcaaaacaaa-gccaagcaaacatttgcataaacttttagcctcttaaaaatttt
1222016.3.peg.2457 ttcaatatgcagcatattgacaattttaattaaaaattatataatcaaaacaaaagctaagcaaacatttgcataatctctaagactaattaaaattca
                   * ***  ***************** ** ** ****  **********  *****    * ****** *******               *   *

31899.10.peg.1045  gaaattattg-------tttaggtgggttgttt-----caaaaaaaaa-ataattaa-----------attt-att---gggaggta-agagagATG
632335.8.peg.2159  aaatttatta-------tttatttgcgtttgtt-----ttaaaaat----cttattaa-----------attt-gct---gggaggta-agaaagATG
632516.3.peg.1534  aaatttatta-------tttatttgcgtttgtt-----ttaaaaat----cttattaa-----------attt-gct---gggaggta-agaaagATG
632348.3.peg.2129  aaatttatta-------tttatttgtgattgtt-----ttaaaa-t----cttatcaa-----------attt-gct---gggaggta-agaaagATG
1121259.3.peg.823  aagtatatta-------tttgtt---gaattgt-----ttaaaatc----cttattaa-----------atttagct---gggaggta-agaaagATG
1387557.3.peg.870  aaagctattg-------gataggtgaattgttt-----taaaagac-----taaccat-----------attt-att---gggaggta-agaaagATG
632292.3.peg.2140  gggtttatta-------ttt-gttgcgttgttt-----caaaaaaa----ctaattaa-----------atta-act---aggaggta-agaaagATG
632518.3.peg.535   gggtttatta-------ttt-gtcgcgttgttt-----caaaaaa-----ctaattaa-----------atta-act---aggaggta-agaaagATG
608506.3.peg.624   gggtttatta-------ttt-gttgcgttgttt-----caaaaa------ctgattaa-----------atta-gct---aggaggta-agaaagATG
351627.8.peg.794   ttaattgtcaattttaatttatgtataaagctt--gatttaaacttagctttccttatttc-----ttaatttaatttaaaggaggtttatactgATG
1387569.3.peg.2093 ttcattgtcaattttaacttatgtgtaaagcct--aatttaatcttagctttcattgttt------ttaatccaatttaaaggaggtttatgttaATG
1222016.3.peg.2457 gttattattggttcagtttgacttgaaggtttttatcttagtttttacctttgtttttttatcaattaatacagtttgaaggaggtctatatttATG
                        * *                        *       *          *                 **     *    ******  *    ***
```

31899.10.peg.2766 (Athe_2204) ***malR1***

31899.10.peg.2766	Caldicellulosiruptor bescii strain DSMZ 6725	1387557.3.peg.2617	Caldicellulosiruptor sp. Wai35.B1
632348.3.peg.465	Caldicellulosiruptor kronotskyensis 2002	608506.3.peg.2119	Caldicellulosiruptor obsidiansis OB47
1121259.3.peg.1274	Caldicellulosiruptor acetigenus DSM 7040	632518.3.peg.2011	Caldicellulosiruptor owensensis OL
632516.3.peg.2187	Caldicellulosiruptor lactoaceticus 6A	1387569.3.peg.1967	Thermoanaerobacter cellulolyticus NA10
632335.8.peg.427	Caldicellulosiruptor kristjanssonii 177R1B	351627.8.peg.1370	Caldicellulosiruptor saccharolyticus DSM 8903
632292.3.peg.609	Caldicellulosiruptor hydrothermalis 108	1222016.3.peg.700	Caldicellulosiruptor changbaiensis strain CBS-Z

```
31899.10.peg.2766  aagcttttcagtataa-------tttgcaattttcagaatatttggtattattgaaattAaactttaagagaataaaggtaagaaag--agttgggatg
632348.3.peg.465   aagcttttcagtataa-------tttgcaattttcagaatatttggtattattgaaattaaactttaagagaataaaggtaagaaag--agttgggatg
1121259.3.peg.1274 aagcttttcagtataa-------tttgcaattttctgaatatttggtactattgaaattaagctttaagaagagaaatgcaaaaaaa-gagttgggatg
632335.8.peg.427   aagcttttcagtataa-------tttgcaattttctgaatatttggtactattgaaattaagctttaagaagagaaatgcaaaaaaa-gagttgggatg
632516.3.peg.2187  aagcttttcagtataa-------tttgcaattttctgaatatttggtactattgaaattaagctttaagaagagaaatgcaaaaaaa-gagttgggatg
632292.3.peg.609   aaacttttcagtataa-------tttgcaattttctgaatatttggtattattgaaattaagctttaagaaaagaaatgcaaaaaaaaagagttgggatg
1387557.3.peg.2617 aaacttttcagtctaa-------tttgcaattttctgaatatttggtattattgaaattaagctttaagaagaaaaatgcaaaaaag--agttgggatg
608506.3.peg.2119  aaacttttcaggataa-------tttgcaattttccaaatatctggtattattaaaattaatctttaagaaaagaaatgcaaaaaaa---gttgggatg
632518.3.peg.2011  aaacttttcaggataa-------tttgcaattttctgaatatttggtattattaaaattaatctttaagaaaagaaatgcaaaaaaa---gttgggatg
351627.8.peg.1370  tcgatattcagcatcaaaagatatttgaaatatttcagaaatttagtattattgatattaagttc----agaa-caaaacaagaagg---aataggatg
1222016.3.peg.700  tcgatattcagcttcaaaagatatttgaaatatttcagaaatttagtattattgatattaagttc----agaa-caagacaagaagg---aataggatg
1387569.3.peg.1967 gtgatttt--gtataagacaaggtttgaaatatctcaaaaatttagtattattgatactaagctt----aagatcaagataaaaaag---aataggatg
                       * **  *  * *       **** *** * **  ** ** * *** **** * * *** *     *  *  *                * *****
```

```
31899.10.peg.2766  agattaagATGGTAACTATTAAAGACATAGCAAGGGAAGCAGGTGTTTCA
632348.3.peg.465   agattaagATGGTAACTATTAAAGACATAGCAAGAGAAGCAGGTGTTTCA
1121259.3.peg.1274 agagtaagATGGTAACAATTAAAGACATAGCAAGGGAAGCAGGTGTTTCA
632335.8.peg.427   agagtaagATGGTAACAATTAAAGACATAGCAAGGGAAGCAGGTGTTTCA
632516.3.peg.2187  agagtaagATGGTAACAATTAAAGACATAGCAAGGGAAGCAGGTGTTTCA
632292.3.peg.609   agagtaagATGGTAACAATTAAAGACATAGCAAGGGAAGCAGGTGTTTCA
1387557.3.peg.2617 agagcaaaATGGTAACTATTAAAGACATAGCAAGGGAAGCAGGTGTTTCA
608506.3.peg.2119  agagtaagATGGTAACAATTAAAGACATAGCGAGAGAAGCAGGTGTTTCA
632518.3.peg.2011  agagtaagATGGTAACAATTAAAGACATAGCGAGAGAAGCAGGTGTTTCA
351627.8.peg.1370  agaatgaaATGGTAACCATAAAGGATATAGCGAGAGAAGCAGGTGTGTCA
1222016.3.peg.700  agaatgaaATGGTAACCATAAAGGATATAGCGAGAGAAGCAGGTGTGTCA
1387569.3.peg.1967 ataatgggATGGTAACCATAAAGGATATAGCAAGAGAAGCAGGGGTGTCA
                   * *     ******** ** ** ** ***** ** ******** ** ***
```

31899.10.peg.868 (Athe_0448) ***pulA2***

31899.10.peg.868	Caldicellulosiruptor bescii strain DSMZ 6725	1387557.3.peg.659	Caldicellulosiruptor sp. Wai35.B1
632348.3.peg.2303	Caldicellulosiruptor kronotskyensis 2002	632292.3.peg.2323	Caldicellulosiruptor hydrothermalis 108
1121259.3.peg.2162	Caldicellulosiruptor acetigenus DSM 7040	632518.3.peg.313	Caldicellulosiruptor owensensis OL
632516.3.peg.906	Caldicellulosiruptor lactoaceticus 6A	608506.3.peg.448	Caldicellulosiruptor obsidiansis OB47
632335.8.peg.2395	Caldicellulosiruptor kristjanssonii 177R1B	1222016.3.peg.2484	Caldicellulosiruptor changbaiensis strain CBS-Z

```
31899.10.peg.868   at--tttcttctcatat-atatacccagaaaaaatatactaaaaacatcttttacatatatattcttaagctacaatttttt-
632348.3.peg.2303  at--tttttctcatattatatacccagaaaaaatagactaaaaacatcttttacatatgtattcttaagctacaattttttt
632516.3.peg.906   at-tttcttctcatat-ttatacccagaaaaaagggtctaaaaacatcttttgcatatgtattctcaaggtgcaa-ttttttt
632335.8.peg.2395  at-tttcttctcatat-ttatacccagaaaaaagggtctaaaaacatcttttgcatatgtattctcaaggtgcaa-ttttttt
1121259.3.peg.2162 at-tttcttctcatat-ttatacccagaaaaaagggtctaaaaacatcttttgcatatgtattctcaaggtgcaa-ttttttt
632292.3.peg.2323  at-ttt-cttctcatat-ttatacccagaaaaaagagactaaaaacatcttttgcatatgtattcttaagctgcaa-tttttc
632518.3.peg.313   at-ttt-cttctcatat-ttatacccagaaaaaagagactaaaaacatcttttgcatatgtattcttaagctgaaa-tttt--c
1387557.3.peg.659  agatttcttctaatat-ttatacccggaaaaaaggggcaaaaaacatcttttgtatatgtgttcttaaactgcaa-ttttttt
608506.3.peg.448   at-tttcttcttttat-ttataccccaataaaagggaataaaaacattttataatttataattctcagtcaacaaattttttt
1222016.3.peg.2484 gaattttaaagggg----gactattcaaaaggcaaagtgaatagtcccctttt-----cgtttttccaa--taaaacttttagg
351627.8.peg.775   gaatgttgaaaggg----gactattcaaaaggcaaagtgaatagtcccctttt-----cgttttttcaa--taaaacttttagg
1387569.3.peg.2117 agatattgaaaaagcaaggaaaaatataaaagcaaagagattgg-catagttt-----cgtttttccaa--taaaactttttggg
                        *                *     *    *          *    * *         **   *      ** ****
```

```
31899.10.peg.868   actttaaaaaag-ggtataaatgatAtataatttcaaaa-tattgcatatcctattctttagaaaaagataggtggttttaATGTTA
632348.3.peg.2303  actttaaaaaag-ggtataaatgatatataatttcagaa-tattgcatatcctattctttagaaaaagataggtggttttaATGTTA
632516.3.peg.906   attttcaaaaag-ggtataaatgatatataatttctgca-tattgcatatcctattctttagaaaaagacaggtggttttaATGATA
632335.8.peg.2395  attttcaaaaag-ggtataaatgatatataatttctgca-tattgcatatcctattctttagaaaaagacaggtggttttaATGATA
1121259.3.peg.2162 attttcaaaaag-ggtataaatgatatataatttctgca-tattgcatatcctattctttagaaaaagacaggtggttttaATGATA
632292.3.peg.2323  actttcaaaaag-ggtataaatgatatataatttctgca-tattgcatatcctattctttagaaaaagacgggtggttttaATGATA
632518.3.peg.313   attttcaaaaag-ggtataaatgatatataatttctgca-tattgcatatcctattatttagaaaaagacgggtggttttaATGATA
1387557.3.peg.659  attttcaaaacg-ggtataaatgatatataatttctgca-tattgcatatcctattctttagaaaaagacaggtggttttaATGATA
608506.3.peg.448   atttcaaaaaag-ggtataaatgatatataatttcttaagtattgcatatcttattatttagaaaaagacaggtggttttaATGATA
1222016.3.peg.2484 gtattaaaaaatcaataaagatgaattagaataatcttattgt--catattctaaatcttaagaatgatagggtgagtttgATGATA
351627.8.peg.775   gtattaaaaaatcaataaagatgaattagaataatcttattgt--catattctaaatcataagaatgatagggtgagtttgATGATA
1387569.3.peg.2117 gtattaaaaaatcaataaagatgaattagaataatcttattgt--catattctaaatcttaagaatgatagggtgagtttgATGATA
                      *  ****     ** * ****  ** ***      * * *  *****  **     **  **      ****  *** *** **
```

31899.10.peg.561 (Athe_0165) *malL*

31899.10.peg.561	Caldicellulosiruptor bescii strain DSMZ 6725
632348.3.peg.2536	Caldicellulosiruptor kronotskyensis 2002
632292.3.peg.2579	Caldicellulosiruptor hydrothermalis 108
1387569.3.peg.47	Thermoanaerobacter cellulolyticus NA10
1222016.3.peg.2759	Caldicellulosiruptor changbaiensis strain CBS-Z
351627.8.peg.2620	Caldicellulosiruptor saccharolyticus DSM 8903
1214564.3.peg.1813	Caldicellulosiruptor sp. F32
608506.3.peg.176	Caldicellulosiruptor obsidiansis OB47
632335.8.peg.182	Caldicellulosiruptor kristjanssonii 177R1B
1121259.3.peg.542	Caldicellulosiruptor acetigenus DSM 7040
632518.3.peg.114	Caldicellulosiruptor owensensis OL

```
31899.10.peg.561   catgagataaaaaaataaattctttttcctcaaaagcatg--ttttagtatattttgtactgataatgttttgtatagcaagtaataca
632292.3.peg.2579  cttgcttaattttttgataaatttttttcctcaaaagaata--gtttagtatcttttgtcttgaaaatgttttgtatagcaagtaatacc
1387569.3.peg.47   cttgcttaattttttgataaatttttttcctcaaaagaata--gtttagtattttttgtctcgaaaatgttttgtatagcaagtaatgtc
632518.3.peg.114   cttgtttaattttttgataaatttttttcctcaaaagaata--gtttagtatcttttgtcttgaaaatgttttgtatagcaagtaatgtc
632348.3.peg.2536  catgagataaaaaaataaattctttttcctcaaaagcatg--ttttagtatattttgtactgataatgttttgtatagcaagtaataca
632335.8.peg.182   catgagataaaaaaataaattctttttcctcaaaagcatg--ttttagtatattttgtactgataatgttttgtatagcaagtaataca
1121259.3.peg.542  catgagataaaaaaataaattctttttcctcaaaagaata--gtttagtatcttttgtcctgataatgttttgtatagcaagtaatgcc
608506.3.peg.176   catgagataaaaaaataaattatttttcctcaaaagaata--gtttagtatcttttgtcctgataatgttttgtatagcaagtaattcc
1222016.3.peg.2759 cacgaactaaagaaataagctattaatagccaattgttcaacgtttagtaacttttcctctgaaaatgttttatattctaaacaacgac
351627.8.peg.2620  catgaattgaagaaataaactattaatagccaattgttcaacatttagtaacttttcctctgaaaatgttttatattctaaacaacgac
1214564.3.peg.1813 catgaattgaagaaataagctattaatagccaatagtacaacgtttagtaactttttctctgaaaatgttttatattctaaacaacgac
                   *  *             *  * **  *   ***  *       *******  ****     ** ********* ***  **  **

31899.10.peg.561   tagtgcttttgttaaatttatgttataatatct-ccCagagtacattat---tgtaggaaaggagcagagATGGAC-T-TGCACAAAAA
632292.3.peg.2579  tactgtttttgtaaaatttatgttatactat-gatataccacattttat---tg----aaaggagcagggatggac-T-TGCACAAAAA
1387569.3.peg.47   tactgtttttgtaaaatttatgttatactattgatataccacactttat---tg----aaaggagcaggaatttacaT-TGCTAAAAAA
632518.3.peg.114   tactgtttttgtaaaatttatgttatactattgatataccacattttat---tg----aaaggagcagggatggac-T-TGCACAAAAA
632348.3.peg.2536  tagtgcttttgttaaatttatgttataatatct-cacagagtacattat---tgtaggaaaggagcagagATGGAC-T-TGCACAAAAA
632335.8.peg.182   tagtgcttttgttaaatttatgttataatatct-cccagagtacattat---tgtaggaaaggagcagggatggac-T-TGCACAAAAA
1121259.3.peg.542  tactgtttttgtaaaatttatgttatactatttatatgctatactttat---tg----aaaggagaagtgattgac-T-TGCACAAAAA
608506.3.peg.176   tactgtttttgtaaaatttatgttatactatttatatgctatattttat---tg----aaaggagaagtgATGGAC-T-TGCACAAAAA
1222016.3.peg.2759 ttctgtttttgtaaataataaggtataatttaaataagagaattcaacagatgctaagaaaggagaaaagcaaacgtATGCATAAAAA
351627.8.peg.2620  tt-tgtttttgtaaataataaggtataatttaaataagagaattcaacagatgcaaagaaaggagaaaagcaaacgtATGCATAAAAA
1214564.3.peg.1813 ttctgtttttgtaaattataaggtataatttagatatgagaattccataggtgctgagaaaggagaagt-caaacgtATGCATAAAAA
                   *  ** ****** **   ** * **** * *           *    *    **     ** *   *            ***  *****
```

31899.10.peg.2884 (Athe_2311) *malR2*

31899.10.peg.2884	Caldicellulosiruptor bescii strain DSMZ 6725	632518.3.peg.2152	Caldicellulosiruptor owensensis OL
632348.3.peg.346	Caldicellulosiruptor kronotskyensis 2002	632292.3.peg.486	Caldicellulosiruptor hydrothermalis 108
632335.8.peg.2526	Caldicellulosiruptor kristjanssonii 177R1B	1214564.3.peg.2455	Caldicellulosiruptor sp. F32
1121259.3.peg.1170	Caldicellulosiruptor acetigenus DSM 7040	351627.8.peg.2690	Caldicellulosiruptor saccharolyticus DSM 8903
632516.3.peg.2440	Caldicellulosiruptor lactoaceticus 6A	608506.3.peg.2228	Caldicellulosiruptor obsidiansis OB47

```
31899.10.peg.2884  -ctcttttttgttgtct-ttgttcaacaaaatttcgcacattttatacaaaatatgtattgatttcgtcaa-agtt
1214564.3.peg.2455 -ggtttttttattatat-ttgttcaacaaattttagcttattttatacaaaatatgtattgatttcgctaa-agta
351627.8.peg.2690  -ggtttttttattatat-ttgttcaacaaattttagcttattttatacaaaatatgtattgatttcgctaa-agta
1121259.3.peg.1170 -t---tttttgttatcc-ttgttcaacaaaatttcgcacattttatacaaaatatgtattgatttcgtcca-agct
632292.3.peg.486   -tctttttttgttatcc-ttgttcaacaaaatttcgcacattttatacaaaatatgtattgatttcgtcca-aatt
632335.8.peg.2526  aacgctttttgttatct-ttgttcaacaaaatttcgcacattttatacaaaatatgtattgatttcgttaa-aatt
632516.3.peg.2440  aacgctttttgttatct-ttgttcaacaaaatttcgcacattttatacaaaatatgtattgatttcgttaa-aatt
632518.3.peg.2152  agggctttttgttgtct-ttgttcaacaaaatttcgcacattttatacaaaatatgtattgatttcgtcaa-agtt
632348.3.peg.346   acagctttttgttatca-ttgttcaacaaaatttagcacatttcatacaaaatatgtattgattttgtcca-gatt
608506.3.peg.2228  aactacttttacaacaagttaataaaaggtattgagacagaggcacgtgaaaaaggatataatataattcttggtg
                         ****        **  * **     **  *        *    *** * *   * ** *

31899.10.peg.2884  aactgctatatttttatttAaa-gccaaaatcgattacgtaccaatataaatca--aagaagaaaaggATGAGGAAGATG-AAGTA
1214564.3.peg.2455 acctgatatattttaaaataaa-tacgaaaacgattac----------tgataaaaagaagggctgggatga--aatATG-AAGTA
351627.8.peg.2690  acctgatatattttaaaataaa-tacgaaaacgattac----------tgataaaaagaagggctgggatga--aatATG-AAGTA
1121259.3.peg.1170 aactgctatatttttatttata-gccgaaatcgattacgtataaatccaaatca--aaga-gaaaaggATGGGAAAGATG-AAGTA
632292.3.peg.486   agctgctatatttttatttata-gccgaaatcgattacgtacaaatccaaatca--aaga-gaaaaggatgaggaagATG-AAGTA
632335.8.peg.2526  agctgctatatttttatttaaa-tccgaaatcgattacgctataatgtaaattattgaggagaaaaggATGAGGAAGATG-AAGTA
632516.3.peg.2440  agctgctatatttttatttaaa-tccgaaatcgattacgctataatgtaaattattgaggagaaaaggATGAGGAAGATG-AAGTA
632518.3.peg.2152  aactgctatatttttatttaaa-gccgaaatcgattacgtatcaatatagatca--aagaagaaaagaATGAGGAAGATG-AAGTA
632348.3.peg.346   agctgctatattcatgtctata-ggcgaaatcgattac-taaaaaacaaaatta---tatggaagaggatgagaaagATG-AAGTA
608506.3.peg.2228  atttttcaaattctcaagaaattgaagaagaatattacagaatgatgaagggcagttagcagatggaatcTTGATTGTTGGAAGTA
                   *  *   * ***       *       **    *****               *       *                ** *****
```

31899.10.peg.2884 **(Athe_2310)** ***malEFG2*** **MalR2-binding site** **(repressor)**

31899.10.peg.2883 Caldicellulosiruptor bescii strain DSMZ 6725
632348.3.peg.347 Caldicellulosiruptor kronotskyensis 2002
608506.3.peg.2227 Caldicellulosiruptor obsidiansis OB47
1121259.3.peg.1171 Caldicellulosiruptor acetigenus DSM 7040
632335.8.peg.2525 Caldicellulosiruptor kristjanssonii 177R1B
632518.3.peg.2151 Caldicellulosiruptor owensensis OL
632516.3.peg.2441 Caldicellulosiruptor lactoaceticus 6A
632292.3.peg.487 Caldicellulosiruptor hydrothermalis 108
1214564.3.peg.2456 Caldicellulosiruptor sp. F32
351627.8.peg.2689 Caldicellulosiruptor saccharolyticus DSM 8903
1387557.3.peg.2742 Caldicellulosiruptor sp. Wai35.B1
1222016.3.peg.2847 Caldicellulosiruptor changbaiensis strain CBS-Z

```
31899.10.peg.2883  aaaaacctattgacaaattgaacaagatataatatattttattTagcgtaaacgattacggtg--------------------gaagacaaaaata----
632348.3.peg.347   aaaaacctattgacaaattgaacaagatataatatattttatttagcgtaaacgattacgatg--------------------gaaggcaaaaata----
632335.8.peg.2525  aaaaacctattgacaaattgaacaagatataatatattttatttagcgtaaacgattacgata--------------------gacaacaaaaata----
632516.3.peg.2441  aaaaacctattgataaattgaacaagatataatatattttatttagcgtaaacgattacgata--------------------gacaacaaaaata----
1121259.3.peg.1171 aaaaacctattgacaaattgaacaagatataatatattttatttagcgtaaacgattacgata--------------------gacaacaaaaata----
1387557.3.peg.2742 aaaaacctattgacaaattgaacaagatataatatattctatttagcgtaaacgattacgata--------------------gacaataaaaata----
632292.3.peg.487   aaaaacctattgacaaattgaacaagatataatatattttatttagcgtaaacgattacgata--------------------ggcatcaaaaata----
608506.3.peg.2227  aaaaacctattgacaaattgaacaagatataatatattttatttagcgtaaacgattacgtca--------------------atcattaaaaata----
632518.3.peg.2151  aaaaacctattgacaaattgaacaagatataatatattttatttagcgtaaacgattacgtta--------------------gttattaaaaata----
1214564.3.peg.2456 aaaaacatattgacaagttaaacaaaacgtagtatattttatttagcgtaaacgattactaagatttcatagaaaaacaactaaaaactaaaaatattac
1222016.3.peg.2847 aaaaacatattgacaagttaaacaaaacgtagtatattttatttagcgtaaacgattactaagatttcatagaaaaacaactaaaaactaaaaatattac
351627.8.peg.2689  aaaaacatattgacaagttaaacaaaacgtagtatattttatttagcgtaaacgattactaagatttcatggaaaaacaactaaaaattaaaaatattac
                   ****** ****** ** ** ***** *  ** ****** ********************                              *******

31899.10.peg.2883  ----attgcctg--------------------------------gcctacacaaa---act-----------------atttagaatcagaccaggcata
632348.3.peg.347   ----attgcctg--------------------------------gtctgcacaaa---act-----------------atttagaatcagaccaggcata
632335.8.peg.2525  ----attgcctg--------------------------------gtctaacaaaa---aat-----------------ctttaggactagaccaggcaca
632516.3.peg.2441  ----attgcctg--------------------------------gtctaacaaaa---aat-----------------ctttaggactagaccaggcaca
1121259.3.peg.1171 ----actgcctg--------------------------------gtctaacaaaa---aat-----------------ctttaagaccagaccaggcaca
1387557.3.peg.2742 ----gctgcctg--------------------------------gtctaaaaaaa---gta-----------------ttttgagctcagaccaggcaca
632292.3.peg.487   ----attgcctg--------------------------------gtctaacaaaa---agt-----------------atttagaaccagaccaggcata
608506.3.peg.2227  ----attgcctg--------------------------------gtc----aaaa---aat-----------------gttttgaattagaccaggcacg
632518.3.peg.2151  ----attgcctg--------------------------------gtc----aaaa---aat-----------------tttttaaatcagaccaggcgta
1214564.3.peg.2456 ataaattgccaacgcaaagacataaaatcttaggagagtatcccacccgacaaaacaaaatgctcggctctttttccacttcaaaaagagccgagccata
1222016.3.peg.2847 ataaattgccaacgcaaagacataaaatcttaggagagtatcccacccgacaaaacaaaatgctcggctctttttccacttcaaaaagagccgagcccca
351627.8.peg.2689  ataaattaccaacacaaag-tgtaaagtgtttgaa---tctcccacccgacaaaacaaaatgctcggctctttttccacttcaaaaagagccgagcccca
                    * **                          *     ***                        **       ** * ** *

31899.10.peg.2883  aaagtaggtatatcccatc-------atatgggataatatacaactcccaatt-tatattatatcataaaa-ctcattaaaaggaggtatttttgtaATG
632348.3.peg.347   aaagtaggtatatcccatc-------atatgggataatatacaactcccaatt-tatattatatcataaaa-ctcattaaaaggaggtatttttgtaATG
632335.8.peg.2525  aaaattggtatatcccata-------atatgggataatatacaactccccattctatattataccataaaa-cttactaaaaggaggtattttta-aATG
632516.3.peg.2441  aaaattggtatatcccata-------atatgggataatatacaactccccattctatattataccataaaa-cttactaaaaggaggtattttta-aATG
1121259.3.peg.1171 aaaattggtatatcccata-------atatgggataatatacaactccccattctatattataccataaaa-cttactaaaaggaggtattttta-aATG
1387557.3.peg.2742 gaaattggtatatcccata-------atctgggataatatacaactccccactctatattataccataaa--cttactaaaaggaggtattttta-aATG
632292.3.peg.487   aaagttggtatatcccata-------atatgggataatatacaactccccattctatattataccataaa--cttactaaaaggaggtattttta-aATG
608506.3.peg.2227  aaaatcgatatatcccata-------ttatgggataatatacaactcccaatt-tatattatatcataaa--tttactaaaaggaggtattttta-aATG
632518.3.peg.2151  aaaatcgatatatcccata-------ttatgggataatatataacccccaatt-tatattatatcataaa--tt-actaaaaggaggtattttta-aATG
1214564.3.peg.2456 aaaattggtatgtcccacactgttgtgtgtgggatgatataaaac-ccctatttaaaattatatcacaaaaacttacaaaaaggaggttgtatttttATG
1222016.3.peg.2847 aaaataggtatgtcccacactgctgtgtgtgggatgatataaaac-ccctatttaaaattatatcacaaaaacttacaaaaaggaggttgtatttttATG
351627.8.peg.2689  aaaataggtatgtcccacactgctgtgtgtgggatgatataaaac-ccctatttaaaattatatcacaaaaacttacaaaaaggaggttgtatttttATG
                    ** * * *** *****          * ****** ***** *** *** * *  * ****** ** ***   * *  **********  * **   ***
```

31899.10.peg.94 **(Athe_2557)** ***mosERP*** **MosR-binding site** **(repressor)**

31899.10.peg.94	Caldicellulosiruptor bescii strain DSMZ 6725	608506.3.peg.2349	Caldicellulosiruptor obsidiansis OB47
632335.8.peg.2375	Caldicellulosiruptor kristjanssonii 177R1B	351627.8.peg.339	Caldicellulosiruptor saccharolyticus DSM 8903
632516.3.peg.1339	Caldicellulosiruptor lactoaceticus 6A	1214564.3.peg.1383	Caldicellulosiruptor sp. F32
1121259.3.peg.1148	Caldicellulosiruptor acetigenus DSM 7040	632292.3.peg.306	Caldicellulosiruptor hydrothermalis 108

```
31899.10.peg.94    ataaataaaagtttt-caagggcttatccagctacagc-tttggatgagcccttttttaaaaaaaacaattgacaga
632335.8.peg.2375  gtaaataagacttttacaagggcttgtccggctgcagcttttggataagcccttttttaaaaaa---ccgttgagagg
632516.3.peg.1339  gtaaataagacttttacaagggcttgtccggctgcagcttttggataagcccttttttaaaaaa---ccgttgacagg
1121259.3.peg.1148 gtaaataagacttttacaagggcttgtccggctgcagcttttggataagcccttttttaaaaaa---ccgttgacagg
608506.3.peg.2349  ataaataagcctttttaaagggcttgtccggctttagccttaggataagctctttttaaaaaa---ccgttgacagg
351627.8.peg.339   gcaaataagaattca-caggggcttgtccaattgaagc-ataggataagcctttttttaaaaaa--caattgacagg
1214564.3.peg.1383 gcaaataagaattca-caggggcttgtccaatttcagc-tttggataagcccttttttaaaaaa--caattgacaga
632292.3.peg.306   ataaataagactttttcaagggcttgtccggctacagc-ttaggataagcccttttttaaaaaa---caattgacaga
                     ******   **    * ****** ***   *  ***  * **** ***  ***** *****   *  **** **

31899.10.peg.94    atataaaaaaacaattaatatagaaAtataaagtgaacgattaagtaacctatttactaaacttttaagtgagctggt-aaaaaATGGATATTA
632335.8.peg.2375  atataaaaaaacaattaatatagaaatataaagtgaacgattaagtaacccatttgcaaaacttttgagaaggtaggt-agaaaATGGATATTA
632516.3.peg.1339  atataaaaaaacaattaatatagaaatataaagtgaacgattaagtaacccatttgcaaaacttttgagaaggtaggt-agaaaATGGATATTA
1121259.3.peg.1148 atataaaaaaacaattaatatagaaatataaagtgaacgattaagtaacccatttgcaaaacttttgagaaggtaggt-agaaaATGGATATTA
608506.3.peg.2349  atataaaaaaacaattaatatagaaatataaagtgaacgattaagtaacccatttgtaaaacttttgagaaggtaggt-agaaaATGGATATTA
351627.8.peg.339   atataaaaaaacaattaatatagaaatataaagtgaacgattaagtaacctatttactaaatttttaagtgagctggtgaaaaaATGGATATTA
1214564.3.peg.1383 atataaaaaaacaattaatatagaaatataaagtgaacgattaagtaacccatttactaaacttttaagtgagctggtgaaaaaATGGATATTA
632292.3.peg.306   atataaaaaaacaattaatatagaaatataaagtgaacgattaagtaacctatttactaaacttttaagtgagctggt-gaaaaATGGATATTA
                   ************************************************** ****   *** **** **   *  ***   *************
```

31899.10.peg.91 **(Athe_2554)** ***mosABC*** MosR-binding site **(potential repressor)**
potential MosSQ-binding site **(tandem repeat)** **(potential repressor)**

31899.10.peg.91	Caldicellulosiruptor bescii strain DSMZ 6725	351627.8.peg.343	Caldicellulosiruptor saccharolyticus DSM 8903
632335.8.peg.2372	Caldicellulosiruptor kristjanssonii 177R1B	1214564.3.peg.1386	Caldicellulosiruptor sp. F32
632516.3.peg.1339	Caldicellulosiruptor lactoaceticus 6A	632292.3.peg.306	Caldicellulosiruptor hydrothermalis
1121259.3.peg.1151	Caldicellulosiruptor acetigenus DSM 7040	632348.3.peg.316	Caldicellulosiruptor kronotskyensis 2002
608506.3.peg.2346	Caldicellulosiruptor obsidiansis OB47		

```
31899.10.peg.91    ctgctggcctttttgattttgtgatgttttcc-aaacttgaaattggtttatttactggtaaagtaaaa-tctaaaatctacaGtataaaatattaaatctctcgg
1121259.3.peg.1151 ctgctggcctttttgattttgttatattttcc-aaacttaaaattggtttatttactggtaaagtaaaa-tctaaaatctgcagtataaaatcttaaatctctcgg
632516.3.peg.1339  ctgctggcctttttgattttgttatattttcc-aaacttaaaattggtttatttactggtaaagtaaaa-tctaaaatctgcagtataaaatcttaaatctctcgg
632335.8.peg.2372  ccgctgtcctttttgattttgttatatttccc-aaactcaaaattggtttatttactggtaaagtaaaa-tctaaaatctacagtataaaatcttaaatctctcgg
632292.3.peg.306   ctgctggcctttttgattttgtgatgttttcc-aaacttgaaattggtttatttactggtaaagtaaaa-tctaaaatctgcggtataaaatattaagtttctcgg
632348.3.peg.316   ctgctggtctttttgattttgtgatattttc--aaacttgaaattagtttatttactggtaaagtaaaa-tctaaaatctgcggtataaaatattaagtttctcgg
351627.8.peg.343   ctgctggcctttttgattttgtaatgttactc-aa--taaaaattggttaatttactggtaaagtaaaa-tctaaaatctgcagtataaaatattaaatttctcgg
1214564.3.peg.1386 ctgctggcctttttgattttgtgatgttttcc-aaacttgaaattggtttatttactggtaaagtaaaa-tctaaaatctgcggtataaaatattaagtttctcgg
608506.3.peg.2346  ttgctggccttttttaatttttattatatttttttaaacttacaatcagtttatttactggtaaagtaaaaatataaaatctacagtataaaatattaaatctctcgg
                     ****  ****** ***** * ** **     **  *   ***  *** ******************* * ******** * ********* **** * ******
```

```
31899.10.peg.91    gttgttaaatatgcaccacaaaatttataatgaatatGtagttgttttgcagtttttctatgcaaaaattacattctagaaaggaggattgattATGTTTAAA
1121259.3.peg.1151 gttgttaaatatgcaccacaaatttataatgaatatgtagttgttttgcagtttttctatacaaaaattacattctaaaaaggaggattgagtATGTTTAAA
632516.3.peg.1343  gttgttaaatatgcaccacaaatttataatgaatatgtagttgttttgcagtttttctatacaaaaattacattctaaaaaggaggattgagtATGTTTAAA
632335.8.peg.2372  gttgttaaatatgcaccacaaatttataatgaatatgtagttgttttgcagtttttctatacaaaaattacattctaaaaaggaggattgagtATGTTTAAA
632292.3.peg.309   gttgttaaatatgcaccacaaatttataatgaacatgtagttgttttgcagtttttctatacaaaaattacattctaaaaaggaggattgattATGTTTAAA
632348.3.peg.316   gttgttaaatatgcaccataaatttataatgaacatgtagttgttttgcagtttttctatacaaaaattacattctagaaaggaggattgattATGTTTAAA
351627.8.peg.343   gttgttaattatgcaccacaaatttataataaacatgtagttgttttgcagtttttctatacaaaaattacattctaaaaaggaggattgattATGTTTAAA
1214564.3.peg.1386 gttgttaaatatgcaccacaaatttataatgaacatgtagttgttttgcagtttttctatacaaaaattacattctaaaaaggaggattgattATGTTTAAA
608506.3.peg.2346  gttgttaaatatgcaccacaaatttataatgaatatgtagttgttttgcagtttttctatacaaaaattacattctgaaaaggaggatggtttATGTTTAAA
                   ******** ********* *** ******** ** ************************** ***************  ********** *  **********
```

31899.10.peg.88 **(Athe_2551)** ***mosSQ***

31899.10.peg.88	Caldicellulosiruptor bescii strain DSMZ 6725	1121259.3.peg.1154	Caldicellulosiruptor acetigenus DSM 7040
632348.3.peg.319	Caldicellulosiruptor kronotskyensis 2002	632516.3.peg.1346	Caldicellulosiruptor lactoaceticus 6A
1222016.3.peg.344	Caldicellulosiruptor changbaiensis strain CBS-Z	632292.3.peg.312	Caldicellulosiruptor hydrothermalis 108
608506.3.peg.2343	Caldicellulosiruptor obsidiansis OB47	351627.8.peg.346	Caldicellulosiruptor saccharolyticus DSM 8903
632335.8.peg.2369	Caldicellulosiruptor kristjanssonii 177R1B		

```
31899.10.peg.88    TTTATACATGGTCTTACAATTGGTAGTATAAAAGAGTAAagaagaatatgttataattaaatCaatattcttcttagtttaaaaaagATGAAAAGATTCATG
1121259.3.peg.1154 TTCATACACGGTCTTACAATTGGTAGTATAAAAGAGTGAagaagagtgtgttataattaaatcaatatccttttagcttaaaagaagatggaaagattcATG
632516.3.peg.1346  TTCATACACGGTCTTACAATTGGTAGTATAAAAGAGTGAagaagagtgtgttataattaaatcaatatccttttagcttaaaagaagatggaaagattcATG
632335.8.peg.2369  TTCATACACGGTCTTACAATTGGTAGTATAAAAGAGTGAagaagagtgtgttataattaaatcaatatcctcttagcttaaaagaagatggaaagattcATG
608506.3.peg.2343  TTCATACACGGTCTTACAATTGGTAGTATAAAAGAGTGAagaagagtgtgttataattaaatcaatatcctcttagcataaaaagaagaggaaagatttATG
1222016.3.peg.344  TTCATACACGGTCTTACAATTGGTAGTATAAAAGAGTGAagaagagtgtgttataattaaatcaatattctcttagcttaaaagaagatggaaagattcATG
632292.3.peg.312   TTCATACACGGTCTTACAATTGGTAGTATAAAAGAGTGAagaagagtgtgttataattaaatcaatattctcttagcttaaaagaagatggaaagattcATG
351627.8.peg.346   TTTATTCATGGTCTTACAATTGGTAGTATAAAAGAGTAAcaagaagtatggtataattaaatcaatatccacat-gcctgaaagaaactgggaagactcATG
632348.3.peg.319   TTTATACATGGTCTTACAATTGGTAGTATAAAAGAGTAAagaagaatatgttataattaaatcaatattcttcttagcttaaaaaagatgaaaagattcATG
                   ** ** ** **************************** *  *  * * ** ***************** *   *    * ***  *             ***
```

31899.10.peg.1130 (Athe_0679) *mosP2*

31899.10.peg.1130	Caldicellulosiruptor bescii strain DSMZ 6725
632348.3.peg.2055	Caldicellulosiruptor kronotskyensis 2002
632335.8.peg.1641	Caldicellulosiruptor kristjanssonii 177R1B
632292.3.peg.2081	Caldicellulosiruptor hydrothermalis 108
632516.3.peg.2164	Caldicellulosiruptor lactoaceticus 6A
608506.3.peg.693	Caldicellulosiruptor obsidiansis OB47
1121259.3.peg.890	Caldicellulosiruptor acetigenus DSM 7040
632518.3.peg.598	Caldicellulosiruptor owensensis OL
1214564.3.peg.1100	Caldicellulosiruptor sp. F32
351627.8.peg.966	Caldicellulosiruptor saccharolyticus DSM 8903
1222016.3.peg.2274	Caldicellulosiruptor changbaiensis strain CBS-Z

```
31899.10.peg.1130  tcacccccgctaaattattgattttctt--ctttattgacaggtttc-aaagatattttacataatttttTgtaattattca
632292.3.peg.2081  tcacccccgttaaattattgattttctt--ttttcttgagagatttttaaaaatatttta-ataattttttgtaattattca
632516.3.peg.2164  tcacccccgctaaattattgattttctt--ttttcttgacagatttttaaaaatatttta-ataattttttgtaattattca
632335.8.peg.1641  tcacccccgctaaattattgattttctt--ttttcttgacagatttttaaaaatatttta-ataattttttgtaattattca
1121259.3.peg.890  tcacccc-gctaaatttttgtttttctt--tgttcttgacagatttttaaaaatatttta-ataattttttgtaattattca
1214564.3.peg.1100 tcacccc-gctaaatttttgtttttctt--tgtccttgacagttttttaaaaatatttta-ataattttttgtaatgattca
351627.8.peg.966   tcacccc-gctaaatttttgtttttctt--tgtccttgacagttttttaaaaatatttta-ataattttttgtaatgattca
1222016.3.peg.2274 tcacctcttt---tgtgaaaattggtt--atccgcttgacagttttttaaaaatatttta-ataattttttgtaatgattca
632348.3.peg.2055  tcacctcttt---tgtgaaaattggtt--atcctattgacaggtttc-aaagatattttacataattttttgtaattattca
608506.3.peg.693   tcacctcttt---ctcaaaaacctgtttgactcccttgacagatttccaaagatattttacataattttttgtaattattca
632518.3.peg.598   tcacctcttttttcttaaaaacctgtttgactcccttgacagatttccaaagatattttacataattttttgtaattattca
                   ***** *                   *        **** ** ***  *** ******** **************** *****

31899.10.peg.1130  ataccaaatgtggtatatttcaatgtatagacaattt-atttgtggggggattaaaacaATGAAAAAGGTCTT
632516.3.peg.2164  acagcaaatgtggtatatttcaatgtatagacaattt-atttgtggggggattaaaataATGAAAAAGGTCTT
632335.8.peg.1641  acagcaaatgtggtatatttcaatgtatagataattt-atttgtggggggattttattA-TGAAAAAGGTCTT
1121259.3.peg.890  acagcaaatgtggtatatttcaatgtatagataattt-atttgtggggggattttattA-TGAAAAAGGTCTT
1214564.3.peg.1100 acagaaaatgtggtatatttcattgtatagataaatc-att-gtggggggatttta-cA-TGAAGAAGGTCTT
351627.8.peg.966   acagaaaatgtggtatatttcattgtatagataaatc-att-gtggggggatttta-cA-TGAAGAAGGTCTT
1222016.3.peg.2274 acagaaaatgtggtatatttcattgtatagataaatc-att-gtggggggatttta-cA-TGAAGAAGGTCTT
632348.3.peg.2055  ataccaaatgtggtatatttcaatgtatagacaattt-atttgtggggggattaaaacaATGAAAAAGGTCTT
608506.3.peg.693   atatcaaatgtggtatatttcaatgtataaataattttatttgtggggggattttaatA-TGAAAAAGGTCTT
632518.3.peg.598   atatcaaatgtggtatatttcaatgtatagataattt-atttgtgaggggattttaatA-TGAAAAAGGTCTT
                   * *  ***************** ****** * ** *  *** *** *******  *    **** ********
```

31899.10.peg.2262 (Athe_1752) *manB*

31899.10.peg.2262	Caldicellulosiruptor bescii strain DSMZ 6725	632518.3.peg.1589	Caldicellulosiruptor owensensis OL
1387569.3.peg.1550	Thermoanaerobacter cellulolyticus NA10	1222016.3.peg.1016	Caldicellulosiruptor changbaiensis strain CBS-Z
632348.3.peg.1021	Caldicellulosiruptor kronotskyensis 2002	608506.3.peg.910	Caldicellulosiruptor obsidiansis OB47
632292.3.peg.1073	Caldicellulosiruptor hydrothermalis 108	351627.8.peg.2479	Caldicellulosiruptor saccharolyticus DSM 8903
1121259.3.peg.611	Caldicellulosiruptor acetigenus DSM 7040	1214564.3.peg.424	Caldicellulosiruptor sp. F32
632335.8.peg.1905	Caldicellulosiruptor kristjanssonii 177R1B	1387555.3.peg.1144	Caldicellulosiruptor sp. Rt8.B8
632516.3.peg.1607	Caldicellulosiruptor lactoaceticus 6A	1387557.3.peg.2027	Caldicellulosiruptor sp. Wai35.B1

```
31899.10.peg.2262  ttctttataaaagt---ttagaaattcctttcaaatatttgaaaaaattctgaaactcttgtatactat-atagtGgtt-----
1387569.3.peg.1550 ctcttt-ttaaaatatttc-gaaaataccttctgctatttgaaaatttttgaaactcttgtatactat-atagtggtcaagca
1387555.3.peg.1144 ctcttt-taaaaatgtttttgaaaatatataccgctatttgaaaaatttttgaaactcttgtatactat-atagtggtcaagca
351627.8.peg.2479  ctctttatttgaataattc-aaaaatatcttgtcttgtttgaaaaattcttgaaacttttgtatactat-ataattgtcaggat
1214564.3.peg.424  ctctttatttgaataattc-aaaaatatcttgtcttgtttgaaaaattcttgaaacttttgtatactat-ataattgtcaggat
1222016.3.peg.1016 ctcttt-tttgaataattc-aaaaatatcttgtcttgtttgaaaaattcttgaaacttttgtatactat-ataattgtcaggat
632348.3.peg.1021  ttctttataaaagt---ttagaaattcctttcaaatatttgaaaaaattctgaaactcttgtatactat-atagtggtt-----
632292.3.peg.1073  ttctttataaaagt---ttagaaattcctttcaaatatttgaaaaaattctgaaactcttgtatactat-atagtggtt-----
1121259.3.peg.611  ttctttataaaaat---ttagaaatttcattcaaatatttgaaaaaatcctgaaactcttgtatactat-atagtggtt-----
1387557.3.peg.2027 ttctttataagaat---ttagaaatttctttcaaatatttgaaaaaatcctgaaactcttgtatactattatagtggtt-----
608506.3.peg.910   ttctttataaaaat---ttagaaattactctcaaatatttgaaaaaattctgaaactcttgtatactat-atagtggtt-----
632335.8.peg.1905  ttctataagcgggtgg-tagt-----tgactcaaatatttgaaaaaatcctgaaactcttgtatactat-atagtggtt-----
632516.3.peg.1607  ttctataagcgggtgg-tagt-----tgactcaaatatttgaaaaaatcctgaaactcttgtatactat-atagtggtt-----
632518.3.peg.1589  ccctacaaactcttga-cactacattcttttcaaatatttgaaaaaattctgaaactcttgtatactat-atagtggtc-----
                     **         *                     * ********  *  ******* *********** *** * **

31899.10.peg.2262  -------------------tcaagatttattatagcacaatcattat-ttctttgcagg-aggtgaaggat--aATGATAAA
1387569.3.peg.1550 ttcacagcaagaccacctttcaagattcattatagcatattttcaattcacattgcagg-aggtgaaaaaa--gATGATAAA
1387555.3.peg.1144 ctctcagcaagaccacttttcaagattcattatagcacattttcaattcagat-gcagg-aggtgaaaaaaaagATGATAAA
351627.8.peg.2479  tt----ataagactatttct-aagattcattatagcacattttatgcactgtttgcagg-aggtgaaacaa--gATGATAAA
1214564.3.peg.424  tt----ataagactatttct-aagattcattatagcacattttatgcactgtttgcagg-aggtgaaacaa--gATGATAAA
1222016.3.peg.1016 tt----ataagactatttct-aagattcattatagcacattttatgcactgtttgcagg-aggtgaaacaa--gATGATAAA
632348.3.peg.1021  -------------------tcaagatttattatagcacaatcattat-ttctttgcagg-aggtgaaggat--cATGATAAA
632292.3.peg.1073  -------------------tcaagatttattatagcacaatcattat-ttctttgcagg-aggtgaagaat--cATGATAAA
1121259.3.peg.611  -------------------tcaagatttattatagcacaatctgcttattctttgcagg-aggtgaagaat--cATGATAAA
1387557.3.peg.2027 -------------------tcaagatttattatagcacaatctttat-ttctttgcagg-aggtgaagaat--cATGATAAA
608506.3.peg.910   -------------------ccaagatttattatagcacaattattac-ttctctgcagg-aggtgaaaagta-tATGATAAA
632335.8.peg.1905  -------------------tcaagatttattatagcacaatctgcttattctttgcagg-aggtgaagaat--cATGATAAA
632516.3.peg.1607  -------------------tcaagatttattatagcacaatctgcttattctttgcagg-aggtgaagaat--cATGATAAA
632518.3.peg.1589  -------------------ctaaaatttattatagcataaccttctgagtctttgcagggaggtgaaaagt--tATGATAAA
                                          ** *** ********* *               ***** *******       ********
```

31899.10.peg.631 (Athe_0232) *GT-manT-mnnA*

31899.10.peg.631	Caldicellulosiruptor bescii strain DSMZ 6725	1121259.3.peg.446	Caldicellulosiruptor acetigenus DSM 7040
632348.3.peg.2468	Caldicellulosiruptor kronotskyensis 2002	351627.8.peg.162	Caldicellulosiruptor saccharolyticus DSM 8903
632292.3.peg.2486	Caldicellulosiruptor hydrothermalis 108	1387569.3.peg.136	Thermoanaerobacter cellulolyticus NA10
632518.3.peg.187	Caldicellulosiruptor owensensis OL	1214564.3.peg.1938	Caldicellulosiruptor sp. F32
608506.3.peg.252	Caldicellulosiruptor obsidiansis OB47	1387555.3.peg.257	Caldicellulosiruptor sp. Rt8.B8
632516.3.peg.1657	Caldicellulosiruptor lactoaceticus 6A	1387557.3.peg.497	Caldicellulosiruptor sp. Wai35.B1
632335.8.peg.274	Caldicellulosiruptor kristjanssonii 177R1B		

```
31899.10.peg.631   gcaaacttaaagtattataaattggagaagataat-attagaaaaagaa-attaaaaatatagaaaaatttttgttaatact
632348.3.peg.2468  gcaaacttaaagtattataaattggagaagataat-attagaaaaagaa-attaaaaatatagaaaaatttttgttaatact
1387569.3.peg.136  gcaaacttaaagtattataaattggagaagataat-attagaaaaagaa-attaaaaatatagaaaaatttttgttaatact
351627.8.peg.162   acaaacttaaagtattagaagttggagaagataat-tttagataaagaa-actaaaattatagaaacatttctgttaaaact
1214564.3.peg.1938 acaaacttaaagtattagaagttggagaagataat-tttagataaagaa-actaaaattatagaaacatttctgttaaaact
632518.3.peg.187   acaaacttaaagtattagaagttggataaaataat-attagccaaaaagattaaaattttagaaaaatttttgttacaaac
608506.3.peg.252   ataaacttaaagtattagcatttggagaaaataat-attagacaaagga-attaaaattacagaagcatttttgttataact
1387555.3.peg.257  ttattttt---tatgtcattttggagaagataaa-tttgtacaaaaaa-tttaaaattatcattaaaatttgttacaacc
1387557.3.peg.497  ttattttt---tatgtcattttggagaagataat-tttatacaaaaaa-aatgaagttattataataatttgttacaaca
632516.3.peg.1657  ttatattt---catctcaacttggaaagtataatatttagacaaatttttaattcaatctcaaaattttgttataact
632335.8.peg.274   ctatattt---catctcaacttggaaagtataatatttagacaaatttttaattcaatctcaaaattttgttataact
1121259.3.peg.446  ctatattt---catctcaacttggaaagtataatatttagacaaatttttaattcaatctcaaaattttgttataact
632292.3.peg.2486  ctatattt---catctcaacttggaaagtataatatttagacaaatttttaattcaatttcaaaattttgttataact
                     *   **     **      ***** *  ****   **    ***                     * ** *****  *
```

```
31899.10.peg.631   atacacaaagtggtataaattatatAaaccct-cttaaa--aaattgccaaaagaaaggagatgaatggTTGCGGATACT
632348.3.peg.2468  atacacaaagtggtataaattatataaaccct-cttaaa--aaattgccaaaagaaaggagatgaatggTTGCGGATACT
1387569.3.peg.136  atacacaaagtggtataaattatataaaccct-cttaaa--aaattgccaaaagaaaggagatgaatggTTGCAGATACT
351627.8.peg.162   ttatgcaaagtggtataaaatatataaaccct-tttaaagaaaattgccaatagaaaggagatgaatggTTGAAAATACT
1214564.3.peg.1938 ttatgcaaagtggtataaaatatataaaccct-tttaaagaaaattgccaatagaaaggagatgaatggTTGAAAATACT
632518.3.peg.187   ttgcacaaagtggtataaattatataaaccta-aagaaat-aaactgtca---gaaaggggatgaatggTTGCAAATACT
608506.3.peg.252   ttgcacaaagtggtataaattatataaaacca-aagaaat-aaactgtca---gaaaggggatgaatgaTTGCGAATACT
1387555.3.peg.257  ttgtataaagtggtataaattatataaaccccatttagaaaaattttcttcaagaaaggggatggatagTTGCGGATACT
1387557.3.peg.497  ttgtataaagtggtataaattatataaaccctatttagaaaaaatttcttaaagaaaggggatggatagTTGCAAATACT
632516.3.peg.1657  ttgcacaaagtggtataaattatataaacccg--aaaagccaagctgtca---gaaaggagatgaatggTTGCGGATACT
632335.8.peg.274   ttgcacaaagtggtataaattatataaacccg--aaaagccaagctgtca---gaaaggagatgaatggTTGCGGATACT
1121259.3.peg.446  ttgcacaaagtggtataaattatataaacccg--aaaagccaaactgtca---gaaaggagatgaatggTTGCGAATACT
632292.3.peg.2486  ttgcacaaagtggtataaattatataaaccct--aaaagtaaaactgtca---gaaaggagatgaatggTTGCGGATACT
                    *    ************* ******** *      *    **  *       ****** **** **  ***   *****
```

31899.10.peg.625 (Athe_0227) *mnnB*

31899.10.peg.625	Caldicellulosiruptor bescii strain DSMZ 6725	632292.3.peg.2501	Caldicellulosiruptor hydrothermalis 108
632348.3.peg.2475	Caldicellulosiruptor kronotskyensis 2002	1222016.3.peg.156	Caldicellulosiruptor changbaiensis strain CBS-Z
632335.8.peg.264	Caldicellulosiruptor kristjanssonii 177R1B	608506.3.peg.244	Caldicellulosiruptor obsidiansis OB47
1121259.3.peg.448	Caldicellulosiruptor acetigenus DSM 7040	632518.3.peg.179	Caldicellulosiruptor owensensis OL
632516.3.peg.1655	Caldicellulosiruptor lactoaceticus 6A	351627.8.peg.153	Caldicellulosiruptor saccharolyticus DSM 8903

```
31899.10.peg.625  aaagggtgatatgaagattttcacccctttatttttttcttaaaaaatgagataaagactgaaaataaagaaagaaaaacttgctgtgagaagtattataat
608506.3.peg.244  aaagggtgatttggatattttcacccttttatttttt-cttaaaaaatgagataaagattaaaaataaagaaagaaaaacttgctgtgagaagtattataat
632518.3.peg.179  aaagggtgatttggatattttcacccttttattt-tttctcaaaaaatgagacaaagactaaaaataaaggaagaaaaatttgctgcgcaaggtattataat
632348.3.peg.2475 aaagggtgatttggatattttcacccttttattt-tttcttaaaaaatgagataaagactaaaaataaagaaagagaaacttgctgtgagaagtattataat
632516.3.peg.1655 aaagggtga-----agattttcacccttttattt-tttcttaaaaaatgagataaagactaaaaataaagaaagagaaacttgctgtgagaagtattataat
632335.8.peg.264  aaagggtgat----aatctttcacccttatttac-tttcttaaaaaatgagataaagactaaaaataaagaaagagaaacttgctgtgagaagtattataat
1121259.3.peg.448 aaagggtgat----aatctttcacccttatttg--tttttaaaaaatgagataaagactaaaaataaagaaagaaaaacttgctgtgaggaatattataat
632292.3.peg.2501 aaagggtgatatgaagattttcacccttttatttttttcttaaaaaatgagataaagactaaaaataaagaaagagaaacttgtggtgagaaatattataat
1222016.3.peg.156 aaaaggtaacaagaagatttctccttattata--tttctaaaattatgagataaagagtaaaaatacaggcagataaagcaaatgtgtaaagtattataat
351627.8.peg.153  aaaaggtaatataaagatttctcctcaat-tatatttctaaaattatgagataaagagtaaaaatacaggcagataaagcaaatgtgtaaagtattataat
                  *** *** *      *   **** **     * *    **  * ***   ****** ***** * ****** **   *** ***        * *      *********

31899.10.peg.625  a-aattct-Aagagacttt-------acaaacacaggagggcagaaact---GTGCGAATAAATCTTGACGGAAAATGGAAGTTCAG
608506.3.peg.244  a-aattct-aagagacttt-------acaaacacaggagggcagaaact---GTGAGAATAAATCTTGACGGGAAATGGAAGTTCAG
632518.3.peg.179  t-aatttt-aagagacttt-------acaaacacaggagggcagaaact---GTGAGAATAAATCTTGACGGGAAATGGAAATTCAA
632348.3.peg.2475 a-aattctgaaaagactatc-----aaaaaaaacaggagggtaaaagct---GTGCGAATAAGTCTTGACGGAAAATGGAAGTTCAG
632516.3.peg.1655 a-aattct-aagagactat-------acaaacacaggagggcaaaagct---GTGAGAATAAGTCTTGACGGAAAGTGGAAGTTTAA
632335.8.peg.264  a-aattct-aagagactat-------acaaacacaggagggcaaaagct---GTGAGAATAAGTCTTGACGGAAAGTGGAAGTTTAA
1121259.3.peg.448 a-aattctgaaaagactatc------aaaaaaacaggaggagaaaagct---GTGAGAATAAATCTTGACGGGAAATGGAAGTTCAG
632292.3.peg.2501 a-aattctgaaaagactatc------aaaaaaacaggagggcaaagact---GTGAAAATAAGTCTTGACGGAAAGTGGAAGTTCAG
1222016.3.peg.156 ataatcttagacaaaacatgttgctgaaaaggaaaaacaggggggagtttacaTTGAAGATAAGCCTTGATGGAAAATGGAAATTTAG
351627.8.peg.153  ataatcttagacaaaacatgttgctgaaaaagaaaaacaggggggagtttacaTTGAAGATAAGCCTTGATGGGAAATGGAAATTTAG
                  * ***  *    *                 **                       **   ****   ***** ** ** ***** ** *
```

31899.10.peg.967 (Athe_0536) *manP*

31899.10.peg.967	Caldicellulosiruptor bescii strain DSMZ 6725	632518.3.peg.438	Caldicellulosiruptor owensensis OL
632348.3.peg.2211	Caldicellulosiruptor kronotskyensis 2002	608506.3.peg.540	Caldicellulosiruptor obsidiansis OB47
632292.3.peg.2219	Caldicellulosiruptor hydrothermalis 108	351627.8.peg.870	Caldicellulosiruptor saccharolyticus DSM 8903
1387557.3.peg.781	Caldicellulosiruptor sp. Wai35.B1	1222016.3.peg.2363	Caldicellulosiruptor changbaiensis strain CBS-Z
1121259.3.peg.1647	Caldicellulosiruptor acetigenus DSM 7040	1387569.3.peg.683	Thermoanaerobacter cellulolyticus NA10
632335.8.peg.604	Caldicellulosiruptor kristjanssonii 177R1B		

```
31899.10.peg.967   aacatatcattttcaaaatttaaaacaagaaaagttcaggttatttttttcaattgaaattttcaaaacttgaattaaaattatgtg
632348.3.peg.2211  aacatatcattttcaaaatttaaaacaagaaaagttcaggttatttttttcaattgaaattttcaaaacttgaattaaaattatgtg
1121259.3.peg.1647 aacatatcattttccaaatttaaggaaagaaaattacaggttatttttttcaattgaaattttcatcggttgaattaaaattatgtg
632335.8.peg.604   aacatatcattttccaaatttaaggaaagaaaattacaggttatttttttcaattgaaattttcatcggttgaattaaaattatgtg
1387557.3.peg.781  aacatatcattttccaaatttaaggaaagaaaattgcaggttatttttttcaattgagattttcaaaagttaaattaaaattatatg
608506.3.peg.540   aacatatcattttcgaaattttaagaaa-agaattgcaggttatttttttcaattgaaattttcaaaagttaaattaaaattatgtt
351627.8.peg.870   gacatatcaattttcaaaaagaaacgcaaaaaggatgcaggatatttttttcaattgcaattttaaaagttaaattaaaattatgtg
1222016.3.peg.2363 gacatatcaattttcaaaaggaacgcaaaaaggatgcaggatatttttttcaattgcaattttaaaagttcaattaaaattatgtg
632518.3.peg.438   aacatatca-ttttcaaaatttaagaaaagaatt-gcaggttatttttttcaattgaaattttcaaaagttaaattaaaattatgtt
1387569.3.peg.683  gacaaatcaatttccaaaaaaaagttcattgaaag-acaggatttttttcaattgcaattttcaagagttcaattaaaattatgtg
632292.3.peg.2219  aacatatcattttctaaaatttaaggaaagaaaagtgcaggttatttttttcaattgaaattttcaaaagttaaattaaaattatgtg
                    *** **** ***   ****         *          **** * ************  ***** *     ** ************ *

31899.10.peg.967   ttGa-aatttcgaaataaaattatttga-taaaaaaacaagagggggcgcaagatacttttATGTTTAAACT
632348.3.peg.2211  ttga-aatttcgaaataaaattatgtga-taaaaaaacaagagggggcgcaagatacttttATGTTTAAACT
1121259.3.peg.1647 ttga-agtttcgaaagaaaattatgtgagcaaaaaaacaaaagggggcgcaagatacttttATGTTTAAACT
632335.8.peg.604   ttga-agtttcgaaagaaaattatgtgagcaaaaaaacaaaagggggcgcaagatacttttATGTTTAAACT
1387557.3.peg.781  ttga-agtttcataagaaaatcatgtgg-ccaaaaaacaaaagggggcgcaagatacttttATGTTTAAACT
608506.3.peg.540   ttga-agtttcaaaagagaatcattatagtgaaaa-acaaa-gggggcgcaagatacttttATGTTTAAACT
351627.8.peg.870   ttga-aacttcaaaagaagaattgatagcagaaaaaacaaa-gggggcgcaagatacttttATGTTCAAACT
1222016.3.peg.2363 atga-aacttcaaaagaagagctgacagcagaaaaaacaaaagggggcgcaagatacttttATGTTCAAACT
632518.3.peg.438   ttga-agtttcaaaagaaaattattata---gtgaaaaacaagggggcgcaagatacttttATGTTCAAACT
1387569.3.peg.683  aggataaatccaaaagaaaaat-tgatt-g--aaagacaaaagggggcgcaagacacttttATGTTCAAACT
632292.3.peg.2219  ttga-agtttcgaaagaaaatttatgtg-agcaaaaacaaaagggggcgcaagatacttttATGTTTAAACT
                     ** *    *  ** ** *              * ****  *** **         ********* *****
```

31899.10.peg.490 (Athe_0104<>Athe_0105) gxgRS-gxgX<>*gxgABC* potential GxgRS-binding site (tandem repeat) (potential activator)

31899.10.peg.490	Caldicellulosiruptor bescii strain DSMZ 6725	608506.3.peg.104	Caldicellulosiruptor obsidiansis OB47
632348.3.peg.133	Caldicellulosiruptor kronotskyensis 2002	1387557.3.peg.362	Caldicellulosiruptor sp. Wai35.B1
632292.3.peg.113	Caldicellulosiruptor hydrothermalis 108	1121259.3.peg.2109	Caldicellulosiruptor acetigenus DSM 7040
632518.3.peg.58	Caldicellulosiruptor owensensis OL	351627.8.peg.2703	Caldicellulosiruptor saccharolyticus DSM 8903
632335.8.peg.2651	Caldicellulosiruptor kristjanssonii 177R1B		

```
31899.10.peg.490   TCAATCTAAAGCTCAAATTTTGCATTCTTTTTCGCATctttg-ttttt-cctccctcAacaaccattatagcatttttg-acacacagc
632348.3.peg.133   TCAATCTAAAGCTCAAATTTTGCATTCTTTTTCGCATctttgcttttt-cctccctcaacaaccattatagcatttttg-acacacagc
632335.8.peg.2651  TGATTCGAAAACTGAATTTTTGCAGTTTTGTCCTCATctttattgctgcctcccttacgtctcattataacattttgt-ttgcaactt
1121259.3.peg.2109 TGATTCGAAAACTGAATTTTTGCAGTTTTGTCCTCATctttattgctgcctcccttacgtctcattataacattttgt-ttgcaactt
1387557.3.peg.362  TAATCTGAAAACTGAATTTTTGCAGTTTAGTCCTCATctttattgcttcctccctcagtcttcattataacattttgt-ttgcaactt
351627.8.peg.2703  TAATCTGAAAGCTGAATTTTTGCAGTTTAGTCCTCATctttattgcttcctccctcagtcttcattataacattttgt-ttgcaactt
632292.3.peg.113   TGATTCTAAAACTCAAGTTTTGCAATCTTTTTTTCATctttgatttttgcctccctcaaaaaccattatagcattttc--atataaagc
632518.3.peg.58    TTATTCTAAAACTCAGGTTTTGCAATCTTTTTTTCATctttgatttttgcctccctcaaaaaccattataacatttac--atatagctt
608506.3.peg.104   TTATTCTAAAACTCAGGTTTTGCAATCTTTTTTTCATctttgatttttgcctccctcaaaaatcattataacatttac--atatagctt
                   * *     ** ** *  *******   *      *** **   **    ******* *     ******* *****        *

31899.10.peg.490   accctatagtaattttttgatttcaaaaaagtgctattcgttttgtaatgaaaatttaataattccaccaacacgtcaaaaaataatagtg
632348.3.peg.133   accctatagtaattttttgatttcaaaaaagtgctattcgtcttgtaatgaaaatttaataattccaccaacacgtcaaaaaataatagtg
632335.8.peg.2651  acc-catagtaattttttgatttcaaaaaagtactattcactttataagacatatttaataattccactaacaagacaaaaaataatagtg
1121259.3.peg.2109 acc-catagtaattttttgatttcaaaaaattactattcactttataagacatatttaataattccactaacaagacaaaaaataatagtg
1387557.3.peg.362  acc-tatagtacttttttgatttcaaaaaagtactattcactttataagacatttttaataattccactaacaagacaaaaaataatagtg
351627.8.peg.2703  acc-tatagtacttttttgatttcaaaaaagtactattcactttataagacatttttaataattccactaataagacaaaaaataatagtg
632292.3.peg.113   accctatagtaattttttgatttcaaaaaagtgctattcattttgcaaagtaaatttaataattccaccaacacgtcaaaaaataatagtg
632518.3.peg.58    gccctatagtaattttttgagttcaaaaaagtgctattcattttgtaaaggaaatttaataattccaccaacatgtcaaaaaataatagtg
608506.3.peg.104   gccctatagtaattttttgagttcaaaaaagtgctattcattttgtaaggaaagtttaataattccaccaatatgtcaaaaaataatagtg
                    **  ****** ******** ********* * ******   **  **   *  ************** ** * * ***************

31899.10.peg.490   tatttgaaaatgtttattgttaaaatgatattGcaaatcaaacaaaaaaatttgataaggaggtttaattgtaATGAAAAA
632348.3.peg.133   tatttgaaaatgtttattgttaaaatgatattgcaaatcaaacaaaaaaatttgataaggaggtttaattgtaATGAAAAA
632335.8.peg.2651  tcaatgtaaatgtttattgttaaaatgatattgcaaaacaaacaaaaaaattgattaaggaggtcaggttgtaATGAAAAA
1121259.3.peg.2109 tcaatgtaaatgtttattgttaaaatgatattgcaaaacaaacaaaaaaattgattaaggaggtcaggttgtaATGAAAAA
1387557.3.peg.362  tcaatgtaaatgtttgttgataaaatgatattgcaaaacaaacaaaaaaatcgattgaggaggtcaggttataATGAAAAA
351627.8.peg.2703  ctaatttaaatgtttgttgttaaaatgatattgcaaatcaaacaaaaaaattgattgaggaggtcatgttataATGAAAAA
632292.3.peg.113   tatttgaaaatgtttattgttaaaatgatattgcaaatcaaacaaaaaaatttaagagggaggttgaattgtaATGAAAAA
632518.3.peg.58    ttttcgataatatttgttgttaaaatgatattgcaaatcaaacaaaaaaatttaagaaggaggtcaaattgtaATGAAAAA
608506.3.peg.104   tttttgataatatttgttgttaaaatgatattgcaaatcaaacaaaaaaatctaagagggaggtcaaattgtaATGAAAAA
                           *** *** *** ***************** *************       *** **    ** * ********
```

1222016.3.peg.2866 Caldicellulosiruptor changbaiensis strain CBS-Z
1387569.3.peg.374 Thermoanaerobacter cellulolyticus NA10
632292.3.peg.123 Caldicellulosiruptor hydrothermalis 108
608506.3.peg.113 Caldicellulosiruptor obsidiansis OB47
1121259.3.peg.2118 Caldicellulosiruptor acetigenus DSM 7040

```
632292.3.peg.123   AATTTTTTGTTGAGGACATttttttacacactcctttaaaaactattttagcatctgaagttatcaagttaaactccaatatacag
1121259.3.peg.2118 AATTTTTTGTTGAGGACATttttttacacactcctttaaaa-ctattttaacatctgaagttatcaagttaaactccaatatacag
608506.3.peg.113   AATTTTTTGTTGAGGTCATttttttacacactcctttaaaaattattttaacatctgaattcagcaagttaaactctaatatacag
1222016.3.peg.2866 ACTTTTTTGCAAAGGTCATttttttacactctcctttacaaactattttatcatctatgttttaagaattaaacaacaataaatag
1387569.3.peg.374  ACTTTTTTGCAAAGGTCATttttttacactctcctttacaaactattttatcatctatgttttaagaattaaacaacaataaatag
                   * *******   *** ************* ******** **  ******* *****    *     * ******   **** * **

632292.3.peg.123   caaaaaattgaccacaaaaaattgcagtaaattttttatttcatttgttcaaaagcttaaaaag-ttgacaaaaaagtttagtgattt
1121259.3.peg.2118 caaaaaattgaccacaaaaaattgcagtaaattttttatttcatttgttcaaaaacttaaaaac-ttgacaaaaaagtttagtgattt
608506.3.peg.113   caaaaaattgaccacaaaaaattgcagtaagtttttcatttagtttgttcataaaactgaaaag-ttgacaaataaatttagtgattt
1222016.3.peg.2866 taaaaaattgagcacaaaaaattgcagtacagcttagaaattatttgtacatatgaataaaaaa-tgaacaaaaaagcacagtgaaat
1387569.3.peg.374  taaaaaaatgagcacaaaaaattgcagtacagcttagaaattatttgtacatatgaataaaaaaatgaacaaaaaaacacagtgaaat
                    ****** *** *****************    **  *  *  ***** ** *    * ****  *  ***** **    *****  *

632292.3.peg.123   gctattaattttgatttaaatttgatagtgtaatcaaa-atttata-taaaaactaaataacatgaggaggttttggtaaATGTT
1121259.3.peg.2118 gctattaattttgatttaaatttgatagtgtaatcaaa-atttata-taaaaactaaataacatgaggaggttttggtaaATGTT
608506.3.peg.113   gctattaattttgatttaaatttgatagtgtaatcaaa-ttttata-gaaaaactaaatagtataaggaggttttggtaaATGTT
1222016.3.peg.2866 attatagattttagtttaaatttattggtgaaatcaaaatctcataatcaaaattaaatattatagggaggttttggtaaATGTT
1387569.3.peg.374  attatagattttagtttaaatttattggtgaaattaaa-tctcataatcaaaattaaatattatagggaggttttggtaaGTGTT
                     ***  *****  *********  * *** *** ***   * ***   **** ******  **  ************** ****
```

31899.10.peg.1037 (Athe_0603) *xylA* XylR-binding site (repressor)

31899.10.peg.1037	Caldicellulosiruptor bescii strain DSMZ 6725
632348.3.peg.2140	Caldicellulosiruptor kronotskyensis 2002
632292.3.peg.2151	Caldicellulosiruptor hydrothermalis 108
632335.8.peg.2163	Caldicellulosiruptor kristjanssonii 177R1B
1121259.3.peg.814	Caldicellulosiruptor acetigenus DSM 7040
632516.3.peg.1539	Caldicellulosiruptor lactoaceticus 6A
1387557.3.peg.860	Caldicellulosiruptor sp. Wai35.B1
632518.3.peg.520	Caldicellulosiruptor owensensis OL
608506.3.peg.613	Caldicellulosiruptor obsidiansis OB47

```
31899.10.peg.1037 tttgcaaaataatctttgtcttgaaagaaggattttttgaaaaaatgaagaatatatattatAgata--ttagtttgtttaataaacaaactaagtaca
632348.3.peg.2140 tta--aaaacattctttgttttgaaagaaggattttttgaaaaagtggagaatatatattataataaacttagtttgtttaataaacaaactaagtatg
632292.3.peg.2151 tttgcaaaataatctttgttttgaaagaaggattttttgaaaaaatgaagaatatatattataatgatgttagtttgtttaataaacaaactaagtacg
1387557.3.peg.860 ttg-caaaataatctttgttgtgaaagaaggattttttgaaaaaatgaagaatatatattatgatgatattagtttgtttaataaacaaactaagtata
608506.3.peg.613  ttg-caaaacattctttgtttt-aaagaaggattttttgaaaaaatgaagaatatataatataataatattagtttgtttaataaacaaactaagtatg
632335.8.peg.2163 ttggaaaaaatttttttgctttaaaagaaggattttttgaaaaaatgaagaatatatattataatgatgttagtttgtttaataaacaaactaattgta
1121259.3.peg.814 ttggaaaaaatttttttgctttaaaagaaggattttttgaaaaaatgaagaatatatattataatgatgttagtttgtttaataaacaaactaattgta
632516.3.peg.1539 ttggaaaaaatttttttgctttgaaagaaggattttttgaaaaaatgaagaatatatattataatgatgttagtttgtttaataaacaaactaattgta
632518.3.peg.520  tggcaaaaaatttttttgctttaaaagaaggattttttgaaaggatgaagaatatatatt-----ataatgttagtttgtttaataaacaaactaattgta
                  *    ****   * ****   * *******************   ** *********         *  ************************* *

31899.10.peg.1037 cgtactggcatgtttaaaaaaataaggttta-gttaaaaaactg-atttattatagaaggagagtgagtt-ataaaATGAAGTACTTCAAAGACATT
632348.3.peg.2140 tgta-tggcatgttttaaaaataagattta-gtcaaaaaatt--atatattatataaggagtgtgaatttataaaATGAAGTACTTCAAAGATATT
632292.3.peg.2151 cgtactggcatgtttaaaaaataaggttta-gttaaaaaaatt-atttattatagaaggagagtgaatttataaaATGAAGTACTTCAAAGATATT
1387557.3.peg.860 cgtactggcatgttctaaaattaaggttta-gtttaaaaattacttttattatagaaggagagtgagttt-taaaATGAAGTACTTCAAAGACATT
608506.3.peg.613  tttacaaacatttttaaaaaagtgggtttacgttaagaaatt--atttattatagaaggagagtgagtt-ataaaATGAAGTACTTCAAAGACATT
632335.8.peg.2163 tacaagaattttgtttaaaacttagatgtg-cttaaaaaattt--ttcattatagaaggagagtgagtt-ataaaATGAAATACTTCAAAGACATT
1121259.3.peg.814 tacaagaattttgtttaaaacttagatgtg-cttaaaaaattt--ttcattatagaaggagagtgagtt-ataaaATGAAATACTTCAAAGACATT
632516.3.peg.1539 tacaagaattttgtttaaaacttagatgtg-cttaaaaaattt--ttcattatagaaggagagtgagtt-ataaaATGAAATACTTTAAAGACATT
632518.3.peg.520  tgtaaggactttgtttaaaacttagatgtg-cttaaaaa-ttt--tttattatagaaggagagtgagtt-ataaaATGAAGTACTTCAAAGACATT
                     *      *  *  ****    * * *   *  * **  *   *  ****** ****** **** **  ********* ***** ***** ***
```

31899.10.peg.1053 **(Athe_0614)** ***xynUVW-xylR-xynA*** **XylR-binding site** **(repressor)**

31899.10.peg.1053	Caldicellulosiruptor bescii strain DSMZ 6725	632292.3.peg.2137	Caldicellulosiruptor hydrothermalis 108
632348.3.peg.2121	Caldicellulosiruptor kronotskyensis 2002	632335.8.peg.638	Caldicellulosiruptor kristjanssonii 177R1B
1387557.3.peg.876	Caldicellulosiruptor sp. Wai35.B1	632516.3.peg.2373	Caldicellulosiruptor lactoaceticus 6A
608506.3.peg.630	Caldicellulosiruptor obsidiansis OB47	1121259.3.peg.826	Caldicellulosiruptor acetigenus DSM 7040
632518.3.peg.542	Caldicellulosiruptor owensensis OL	1222016.3.peg.2454	Caldicellulosiruptor changbaiensis strain CBS-Z
351627.8.peg.797	Caldicellulosiruptor saccharolyticus DSM 8903	1387569.3.peg.2083	Thermoanaerobacter cellulolyticus NA10
1387555.3.peg.737	Caldicellulosiruptor sp. Rt8.B8	1214564.3.peg.112	Caldicellulosiruptor sp. F32

```
31899.10.peg.1053  acaaaagattaaaaaatcgaagaaggagttttaaaatttgcagcgaaata-tataaatAgtttgtttaagaaataaaccaa-aaggagtgatt-taatGTGAA
608506.3.peg.630   ctaaaacattaaaaaatcaaagaaggattttaaaaattatagcgaaata-tataaatagtttgtttaataaataaactaa-aaggagtgatg-ttgtGTGAA
632518.3.peg.542   ataaaacattaaaaaatcaaagaaggattttaaaaattatagcgaaata-tataattagtttgtttaataaataaacaa--aaggagtgatg-ttgtGTGAA
632348.3.peg.2121  acaaaagattaaaaaatcgaagaaggagttttaaaatttgcagcgaaata-tataaatagtttgtttaataaataaaccaa-aaggagtggtg-taatGTGAA
632292.3.peg.2137  acaatagattaaaaaatcaaagaaggaatttaaaatttgcagcgaaata-tataaatagtttgtttaataaataaacaaa-aaggagtggtg-taatGTGAA
632516.3.peg.2373  acgaaagattaaaaaatcaaagaaggaatttaaaatttgcagcgaaata-tataaatagtttgtttaataaataaacaaa-aaggagtggtg-taatGTGAA
1121259.3.peg.826  acgaaaaattaaaaaatcaaagaaggaatttaaaatttgcagcgaaata-tataaatagtttgtttaataaataaacaaa-aaggagtggtg-taatGTGAA
632335.8.peg.638   ataaaaggttaaaaaattaaagaaggaatttaaatttgcagcgaaata-tataaatagtttgtttaataaataaacaaa-aaggagtggtg-taatGTGAA
1387557.3.peg.876  ataaaagatagaaaaaatggagaaggaatttaaaatttgtagcgaatta-tattaatagtttgtttattaaataaactaataaggagtgaggatgctGTGAA
1222016.3.peg.2454 ctaatttaataaa-aaatcaagaaggaaaattaaaattttggcgaagtaatataattagtttatttagtaaacaaactaaaaaggagtgat--tggtGTGAA
1214564.3.peg.112  ctaatttaataaataagtcaaaatggacaataaaaattttggcgatataatataattagtttatttaataaacaaacaa-aaaggagtgat--tggtGTGAA
1387569.3.peg.2083 acaattcaataaa-aagtcaagaaggaaaataaaaaatttggcgaaatattataattagtttatttaataaacaaactaaaaagaagtgat--tggtGTGAA
351627.8.peg.797   ctaatttaataaa-aaatcaagaaggaaaattaaaattttggcgaaataatataattagtttatttagtaaacaaactaaaaaggagtgat--tggtGTGAA
1387555.3.peg.737  caattttgataaa-aagtcgagaaggaaaaataaaaaaattagagaattaatataattagtttatttaataaacaaactaagaaggagtgat--ttttGTGAA
                                        ** **     * * ***      ***         *  **   **  *** * ****** ****   *** **** ** *** ****     *  ******
```

31899.10.peg.1056 **(Athe_0617)** ***xylR-xynA***

31899.10.peg.1056	Caldicellulosiruptor bescii strain DSMZ 6725	632516.3.peg.2376	Caldicellulosiruptor lactoaceticus 6A
632348.3.peg.2118	Caldicellulosiruptor kronotskyensis 2002	608506.3.peg.633	Caldicellulosiruptor obsidiansis OB47
632292.3.peg.2134	Caldicellulosiruptor hydrothermalis 108	632518.3.peg.545	Caldicellulosiruptor owensensis OL
632335.8.peg.641	Caldicellulosiruptor kristjanssonii 177R1B	1387557.3.peg.879	Caldicellulosiruptor sp. Wai35.B1
1121259.3.peg.829	Caldicellulosiruptor acetigenus DSM 7040	1387569.3.peg.2080	Thermoanaerobacter cellulolyticus NA10

```
31899.10.peg.1056  ttgt-tttagcgtca-aaaactgttttcccgcaacctattttgttgtataataatttgcAaagcttaatta-gcaaaggaaaatgacaggATGGGTAACCA
632348.3.peg.2118  ttgt-tttagcgtca-aaaactgttttcctgcaacctattttgttgtataataatttgcaaagcttaatta-gcaaaggaaaatgacaggATGGGTAACCA
632292.3.peg.2134  atgt-tttaacgtca-aaaactgttttcccacagcctattttgttgtataataatttgcaaagcttaatta-gtaaaggaaaatgacaagATGGGCAACCA
608506.3.peg.633   atgtttttgctccttaaaaacattttcccacagctt-tttttgttgtataataatttgcaaagtctaaataagcagaggaaaatgacaggATGGGAAATCA
632518.3.peg.545   atgtgttttgcgcct-aaaaacattttcccacagctt-tttttgttgtataataatttgcaaagtttaaataagcagaggaaaatgacaggATGGGTAATCA
1387569.3.peg.2080 atgt-tttaatg----aagaatatttcatgaacaaaaagttttgagttataataatttccaaacac--attttaaaaaaggacatgaagagATGGGCAATCA
1222016.3.peg.2451 atgt-tttaatg----aagaatatttcatgaacataaagttttgagttataataatttccaaacac--attttaaaaaaggacatgaagaaATGGGCAATCA
632335.8.peg.641   ttgcatttg-tgtga--tagacactttctatc------gttttattgtataataatttgcaaactcaaactatgtaaaggaaaatgacaaaATGGGGAATCA
632516.3.peg.2376  ttgcatttg-tgtga--tagacactttcaatc------tttttattgtataataatttgcaaactcaaactatgtaaaggaaaatgacaaaATGGGGAATCA
1121259.3.peg.829  ttgcattta-tgtga--tagacactttctatc------tttttattgtataataatttgcaaactcaaactatgtaaaggaaaatgacaaaATGGGGAATCA
1387557.3.peg.879  ttgcatttaatgtta--tagacactttctatc------gtttgttgtataataatttgcaaactcaaactgtgtgaaggataatgacaaaATGGGGAACCA
                    **   ***                **                ****     *********** ****      * *        * *    ****     ***** ** **
```

31899.10.peg.2315 (Athe_1803) *msmK* predicted XylR binding site (weak site score=5.03) (potential activator)

31899.10.peg.2315	Caldicellulosiruptor bescii strain DSMZ 6725	608506.3.peg.1732	Caldicellulosiruptor obsidiansis OB47
1387557.3.peg.2083	Caldicellulosiruptor sp. Wai35.B1	1387555.3.peg.1098	Caldicellulosiruptor sp. Rt8.B8
632348.3.peg.968	Caldicellulosiruptor kronotskyensis 2002	632518.3.peg.1644	Caldicellulosiruptor owensensis OL
632516.3.peg.1011	Caldicellulosiruptor lactoaceticus 6A	1222016.3.peg.1977	Caldicellulosiruptor changbaiensis strain CBS-Z
632335.8.peg.973	Caldicellulosiruptor kristjanssonii 177R1B	351627.8.peg.2531	Caldicellulosiruptor saccharolyticus DSM 8903
1121259.3.peg.667	Caldicellulosiruptor acetigenus DSM 7040	1214564.3.peg.774	Caldicellulosiruptor sp. F32
632292.3.peg.1015	Caldicellulosiruptor hydrothermalis 108	1387569.3.peg.1615	Thermoanaerobacter cellulolyticus NA10

```
31899.10.peg.2315  gctgataaggaaaggctccttatcagcctttgtttttggtaagatagtttagacatgttatctaaaaggtttttgaaaagtttgtttaaaaaatcaatag
632348.3.peg.968   gctgataaggaaaggctccttatcagcctttgtttttggtaagatagtttagacatgttatctaaaaggtttttgaaaagtttgtttaaaaaatcaatag
608506.3.peg.1732  gctgataaggtacaactccttatcagcctttgtttttggtaagatagtttagacatgttatctaaaaggtttttgaaaagtttgtttaaaaaatcaatag
632518.3.peg.1644  gctgataaggtacaactccttatcagcctttgtttttggtaagatagtttagacatgttatctaaaaggtttctgaaaagtttgtttaaaaaatcaatag
632516.3.peg.1011  gctgataaggtacaactccttatcagcctttgtttttggtaagatagtttagacatgttatctaaaaggtttttgaaaagtttgtttaaaaaatcaatag
632335.8.peg.973   gctgataaggtacaactccttatcagcctttgtttttggtaagatagtttagacatgttatctaaaaggtttttgaaaagtttgtttaaaaaatcaatag
1121259.3.peg.667  gctgataaggtacaactccttatcagcctttgtttttggtaagatagtttagacatgttatctaaaaggtttttgaaaagtttgtttaaaaaatcaatag
632292.3.peg.1015  gctgataaggtacaactccttatcagcctttgtttttggtaagatagtttagacatgttatctaaaaggtttttgaaaagtttgtttaaaaaatcaatag
1387557.3.peg.2083 gctgataaggtacaactccttatcagcctttgtttttggtaagatagtttagacatgttatctaaaaggtttttgaaaagtttgtttaaaaaatcaatag
1387555.3.peg.1098 gctgataaggtacaactccttatcagcctttgtttttggtaagatagtttagacatgttacctaaaaggtttttaaaaagtttgtttaaaaaatcaatag
1387569.3.peg.1615 gttgataaggtacaactccttatcagcctttgtttttggtaagatagtttagacatgttacctaaaaggtttttaaaaagtttgtttaaaaaatcaatag
1222016.3.peg.1977 gctgataaggtacaactccttatcagcctttgtttttggtaagatagtttagacatgttatctaaaaggtttttgaaaagtttgtttaaaaaatcaatag
351627.8.peg.2531  gctgataaggtacaactccttatcagcctttgtttttggtaagatagtttagacatgttatctaaaaggtttttgaaaagtttgtttaaaaaatcaatag
1214564.3.peg.774  gctgataaggtacaactccttatcagcctttgtttttggtaagatagtttagacatgttatctaaaaggtttttgaaaagtttgtttaaaaaatcaatag
                   * ******** *   ********************************************* *********** * ************************

31899.10.peg.2315  tttttaatcgcaaataattgtatagtctatatTagaagaagatttattacaagttt-atatacaa-tactgtgtaggaggtaaaagaaaaGTGGCAAGTG
632348.3.peg.968   tttttaatcgcaaataattgtatagtctatattagaagaagatttattacaagttt-atatacaa-tactgtgtaggaggtaaaagaaaaGTGGCAAGTG
608506.3.peg.1732  tttttaatcgcaaataattgtatagtctatattagaagaagatttttgcaagttt-atatacaa-tactgtgtaggaggtaaaagaaaaGTGGCAAGTG
632518.3.peg.1644  tttttaatcgcaaataattgtatagtctatattagaagaagatttttacaagttt-atatacaa-tactgtgtaggaggtaaaagaaaaGTGGCAAGTG
632516.3.peg.1011  tttttaatcgcaaataattgtatagtctatattagaagaagatattttgcaagttt-atatacaa-tactgtgtaggaggtaaaagcttaATGGCAAGTG
632335.8.peg.973   tttttaatcgcaaataattgtatagtctatattagaagaagatattttgcaagttt-atatacaa-tactgtgtaggaggtaaaagcttaATGGCAAGTG
1121259.3.peg.667  tttttaatcgcaaataattgtatagtctatattagaaaagatttttgcaagttt-atatacaa-tactgtgtaggaggtaaaagcttaATGGCAAGTG
632292.3.peg.1015  tttttaatcgcaaataattgtatagtctatattagaagaagatattttgcaagttt-atatacaa-tactgtgtaggaggtaaaagcttaATGGCAAGTG
1387557.3.peg.2083 tttttagtcgcgaataattgtatagtctatattagaagaaaat-ttttgcgaatta-atatacaa-tactgtgtaggaggtaaaagaaaaGTGGCAAGTG
1387555.3.peg.1098 tttttggtcgcaaataattgtatagtctatattagaagaaaat-ttttgcgaattttatatacaa-tactatgtaggaggtaaaagagaaGTGGCAAGTG
1387569.3.peg.1615 tttttggccgcaaataattgtatagtctatagtagaagaaaat-ttttgcgaatta-atatacaaatactgtgtaggaggtaaaagaaaaGTGGCAAGTG
1222016.3.peg.1977 tttttagtcgcaaataattgtatagtctatattagaagaaaat-ttttgcgaatta-atatacaa-tactatgtaggaggtaaaagtttaATGGCAAGTG
351627.8.peg.2531  tttttagtcgcaaataattgtatagtctatattagaagaaaat-ttttgcgaatta-atatacaa-tactatgtaggaggtaaaagtttaATGGCAAGTG
1214564.3.peg.774  tttttagtcgcaaataattgtatagtctatattagaagaaaat-ttttgcgaatta-atatacaaatactgtgtaggaggtaaaagaaaaGTGGCAAGTG
                   *****    *** ****************** ***** ** ** * ** * * **  ******** **** ***************    * *********
```

31899.10.peg.999 (Athe_0566) *xynR-xylB* XynR-binding site (repressor)

31899.10.peg.999	Caldicellulosiruptor bescii strain DSMZ 6725	632516.3.peg.2242	Caldicellulosiruptor lactoaceticus 6A
632348.3.peg.2182	Caldicellulosiruptor kronotskyensis 2002	632335.8.peg.2194	Caldicellulosiruptor kristjanssonii 177R1B
632292.3.peg.2180	Caldicellulosiruptor hydrothermalis 108	1387557.3.peg.818	Caldicellulosiruptor sp. Wai35.B1
632518.3.peg.480	Caldicellulosiruptor owensensis OL	351627.8.peg.906	Caldicellulosiruptor saccharolyticus DSM 8903
1222016.3.peg.2326	Caldicellulosiruptor changbaiensis strain CBS-Z	1387569.3.peg.750	Thermoanaerobacter cellulolyticus NA10
608506.3.peg.579	Caldicellulosiruptor obsidiansis OB47	1214564.3.peg.1549	Caldicellulosiruptor sp. F32
1121259.3.peg.773	Caldicellulosiruptor acetigenus DSM 7040	1387555.3.peg.2372	Caldicellulosiruptor sp. Rt8.B8

```
31899.10.peg.999   ttttaaaa-ttttgttgataaaccaactaccaatgtgataaagtatataacAaagaataatcgaaatcgcttacaattc
1387557.3.peg.818  ttttaaaaattttgttgataaaccaactaccaatgtgataaagtatataacaaagagaaatcgaaatcggttacaatat
1387569.3.peg.750  ttttaaaaattttgttgataaaccaactaccaatgtgataaagtatataacaaagagaaatcgaaatcggttacaatat
351627.8.peg.906   ttttaaaaattttgttgataaaccaactaccaatgtgataaagtatataacaaagagaaatcgaaatcggttacaatat
1214564.3.peg.1549 ttttaaaaattttgttgataaaccaactaccaatgtgataaagtatataacaaagagaaatcgaaatcggttacaatat
1121259.3.peg.773  tattgaaattttgttgaaaaccaaacaacaatatgataaagtatataacaaagaacaatcgaaatcgcttacaactt
632516.3.peg.2242  tattgaaattttgttgaaaaccaaacaacaatatgataaagtatataacaaagaacaatcgaaatcgcttacaactt
632335.8.peg.2194  cattaaaattttgttgaaaaccaaacaacaatatgataaagtatataacaaagaagaatcgaaatcgcttacaactt
632348.3.peg.2182  tttaaaaa-ttttgttgataaatcaactaccaatgtgataaagtatataacaaagaataatcgaaatcgcttacaactt
632292.3.peg.2180  tattaat--ttttgttgaaaaccaactaccaatgtgataaagtatatatcaaagaataatcgaaatcgcttacaacac
1222016.3.peg.2326 tattaat--ttttgttgaaaactaactaccaatatgataaagtatatatcaaagaataatcgaaatcgcttacaactc
632518.3.peg.480   tactaaaattttgttgaaaatccaaacaacaatgtgataaagtatataacaaagaataatcgaaatcgcttacaactt
608506.3.peg.579   tactaaaattttgttgaaaaccaaacaacaatgtgataaagtatataacaaagaataatcgaaatcgcttacaactt
1387555.3.peg.2372 gttaaatattttta ttgaataaggttgataaaatgtgatatagt-tataaaaaggaagcatagaaatcgcttacagctc
                        *   **** ****  *         *** ***** *** ****  ** **   ** ******* *****
```

```
31899.10.peg.999   ctg-------tatttgataaaattaaaaag-ga-aaatgaaaggggcagagcatTT-GCCGTCGATTGAGGATGTT
1387557.3.peg.818  ctga------tgtttataaagatgagaaaacaaagagaattaggaggtgaaatttTT-GCCGTCGATTGAAGATGTT
1387569.3.peg.750  ctga------tgtttataaagatgagaaaacaaagagaattaggaggtgaaatttTT-GCCGTCAATTGAAGATGTT
351627.8.peg.906   ctga------tgtttataaagatgagaaaacaaagagaattaggaggtgaaatttTT-GCCATCAATTGAAGATGTT
1214564.3.peg.1549 ctga------tgtttataaagatgagaaaacaaagagaattaggggggtgaaatttTT-GCCATCAATTGAAGATGTT
1121259.3.peg.773  ctat------gatttataaa-ctaaaaag--tgtaagagaaagggGTGAAATTTTT-GCCGTCGATAGAAGATGTT
632516.3.peg.2242  ctat------gatttataaa-ctaaaaag--tgtaagagaaaggggtgaaatttTT-GCCGTCGATAGAAGATGTT
632335.8.peg.2194  ctat------gatttataaa-ctaaaaag--tgtaagagaaagggGTGAAATTTTT-GCCGTCGATAGAAGATGTT
632348.3.peg.2182  cta-------tatttgagaaatttaaaaaa-gctaagtgaaaggaggtagagcatTT-GCCATCAATTGAAGATGTT
632292.3.peg.2180  ctg-------tatttgataaaattaaaaaa-gtaaaatgaaaggggcagagtatTT-GCCGTCAATTGAGGATGTT
1222016.3.peg.2326 atg-------tatttgataaaactaaaaaaagcaaaatgaaaggaggcagagtatTT-GCCGTCAATTGAGGATGTT
632518.3.peg.480   ctagctcatatattttataaaattgttaag--caaaatgaaaggggcagtgtattTTGCCGTCAATTGAAGATGTT
608506.3.peg.579   cta-------tattttataaaattgtgaag--caaaatgaaaggggtagtgtattTTGCCGTCAATTGAAGATGTT
1387555.3.peg.2372 -----------aattgataaagagaataaactaaagaaggagggtaataaaaTT-GCCATCAATTGAAGATGTT
                                ** **       **              *           * *** ** ** ** ******
```

31899.10.peg.2390 (Athe_2354) ***xyl3A*** **XynR-binding site (repressor)**

31899.10.peg.2930	Caldicellulosiruptor bescii strain DSMZ 6725	632518.3.peg.2229	Caldicellulosiruptor owensensis OL
632292.3.peg.189	Caldicellulosiruptor hydrothermalis 108	608506.3.peg.2281	Caldicellulosiruptor obsidiansis OB47

```
31899.10.peg.2930  caaatgatgttgaaaatcaaatgatgttataattaatacAaatagaataatattcatgcttttta-ttgttttaaattgcttgaaaccgctttctaacctc
632292.3.peg.189   caaatggtgtgaaaaaccaaatggtgttataatcataataa--aagatgatattcacactattagttgttttatctatcttgaaaccgttttctaccccc
632518.3.peg.2229  ttgatgaaagtgcatagttgatgaatttaaattgacaatgg-tataattaagttgaaac-ataattttgtttatgaaatttgaaaccgttttctaccccc
608506.3.peg.2281  ttgatgaaagtgcatagttgatgaatttaaattgacaatga-tataatttaattgagac-ataattttattcatgaacattgaaaccgttttctaccccc
                      ***       * *    ***   *** * * *  *     *  **    ** *  *  * * **  ** *      ********* ****** ** *

31899.10.peg.2930  --agcacaaagtttttttgaaaatcatatttggaggtgcaagtaaaaGTGTCAATTGAAAAAAGGGTAAACCAGCTTTTGCAGCAGATGA
632292.3.peg.189   --agcacaaagtttttttgaaaatcatatttggaggtgcaagtaaaaGTGTCAATTGAAAAAAAAGTAAACGAGCTTTTGAAGCAAATGA
632518.3.peg.2229  --agcacaaagtttttt--aaaattatttcaggaggtgta-tttcaaATGTCAATTGAAAAAAAGGTAAATGAGCTTTTACAGAAGATGA
608506.3.peg.2281  ccggcacaaagtttttt--aaatttcaagttggaggtgtaatttcaaGTGTCAATTGAAAAAAAGGTAAATGAGCTTTTACAGAAAATGA
                      **************  *** *       ******* *  *  ** ***************  *****  *******  ** * ****
```

351627.8.peg.677	Caldicellulosiruptor saccharolyticus DSM 8903	1387555.3.peg.2541	Caldicellulosiruptor sp. Rt8.B8
632516.3.peg.931	Caldicellulosiruptor lactoaceticus 6A	632335.8.peg.2598	Caldicellulosiruptor kristjanssonii 177R1B
1121259.3.peg.1395	Caldicellulosiruptor acetigenus DSM 7040	1222016.3.peg.2608	Caldicellulosiruptor changbaiensis strain CBS-Z
632348.3.peg.192	Caldicellulosiruptor kronotskyensis 2002	1214564.3.peg.898	Caldicellulosiruptor sp. F32
1387557.3.peg.2782	Caldicellulosiruptor sp. Wai35.B1	1387569.3.peg.600	Thermoanaerobacter cellulolyticus NA10

```
351627.8.peg.677   tacatttaatttttaattt-atttgaaaccgttttctaaattcaatttt-gtttgattttatt-tggtatctacccccagcacattt
1214564.3.peg.898  tacatttaatttttaattt-atttgaaaccgttttctaaattcaatttt-gtttgattttatt-tggtatctacccccagcacattt
1222016.3.peg.2608 tacatttaatttttaattt-atttgaaaccgttttctaaattcaattttgtttgaatttatt-tggtacctacccccagcacattt
632335.8.peg.2598  tacatttaatttataatttaatttgaaaccgttttctaaggtttaatttc-atttgattttgtt-tcgtgcctacccccagcacattt
1387569.3.peg.600  tgggtattattttactcattattgaaaccgttttctaatttgatttcactttgattttgctgtatttcctacccctagcacattt
632516.3.peg.931   tgcctttccttttaaaatt-agttgaaaacgctttctgtt-------ttatttaaaccctttt----tt---acccccagcacaatt
632348.3.peg.192   tgcctttccttttaaaatt-agttgaaaacgctttctgtt-------ttatttaaaccctttt----tt---acccccagcacaatt
1121259.3.peg.1395 tgccttttcttttgaaagt-aattgaaaacgctttctatt-------ttaattacaagcctttt----tttttgcccccagcacaatt
1387557.3.peg.2782 tgaaattattttagagca-atttgaaaccgatttctgaatt-ggtttcaaaagtttatttcatttttttctacccccagcacaaaa
1387555.3.peg.2541 ttaaattattttagccca-acttgaaaccgatttctaaact-ggtttcaaaagat-gtttcatttttt-ctacccccggcacaaaa
                   *     *  ***          ****** ** *****          *                   *     ****  *****
351627.8.peg.677   ttttaaaccttttaaaatccaaaaaaattggaggtgttttaaaaaGTGTCAGTTGAAAAGAAAGTGAATGATCTTTTGCAGA
1214564.3.peg.898  ttttaaaccttttaaaatccaaaaaaattggaggtgttttaaaaaGTGTCAATTGAAAAGAAAGTGAATGATCTTTTGCAGA
1222016.3.peg.2608 ttt-aaacctttttaaaatccaaaaaaatcggaggtgttttaaaaaGTGTCAATTGAAAAGAAAGTGAATGATCTTTTGCAGA
632335.8.peg.2598  t---aaatcttttaaagtctaaaaaatcggaggtgtttttaaaaGTGTCAATTGAAAAGAGAGTAAATGATCTTTTGCAGA
1387569.3.peg.600  t---aaatttttttaaagtctaaaaa-tcggaggtgttttaaaaaGTGTCAATTGAAAAGAAAGTGAATGATCTTTTGCAGA
632516.3.peg.931   ttt-aaa-gatttaga------aaaattggaggtgtgtgtagagGTGTCAATTGAAAAGAAGGTAAATGAACTTTTACAGA
632348.3.peg.192   ttt-aaa-gatttaga------aaaattggaggtgtgtgtagagGTGTCAATTGAAAAGAAGGTACAAGAACTTTTACAGA
1121259.3.peg.1395 ttt-aaaagatttaga------aaaattggaggtgtat--agagGTGTCAATTGAAAAAAGGGTAAACGAGCTTTTACAGA
1387557.3.peg.2782 ----a-taatattgcagcttaaaattt-ggaggtgtttttaa--GTGTCTATTGAGAAAAAGGTAAATGATCTTTTGCAAA
1387555.3.peg.2541 ----aataatattacagcctgtaattttggaggtgtttttag--GTGTCTATTGAAAAAAGGGTAAACAAGCTTCTCCAGA
                       *      **  *      **  * ******** *  *    *****  **** ** *  **  *  * *** * ** *
```

31899.10.peg.328 (Athe_2722) *xynMNC* XynR-binding site (repressor)

31899.10.peg.328 Caldicellulosiruptor bescii strain DSMZ 6725
632348.3.peg.2706 Caldicellulosiruptor kronotskyensis 2002

```
31899.10.peg.328  agacaacaaaaatatttttt-tggttttatttatttcgtgagatgatataatctttttAgaaactatgttgtatattttttta
632348.3.peg.2706 aggtaattaaaacagtgagaatatttttattttattata-aaaatgatataatttttttgtta--tatttgatttgtttaataa
                  **  **  **** * *     *  ************     * ********** *****   *  *** *  * * ***  * *

31899.10.peg.328  ttt----tcatgaaatcgctttcctatctaaaatattaaaagttaaggtgattgagtATGTCGTTAAGAATGCCTTATTT
632348.3.peg.2706 gtttaataattgaaatcgttttcctattccaaatatttaaagccagggtgattaagtATGTCAATAAAGCTTCCCTATTT
                   *** **    ******* ********   ******* ****  * ******* ********  ***   * ** *****
```

1121259.3.peg.2340	Caldicellulosiruptor acetigenus DSM 7040	632335.8.peg.2087	Caldicellulosiruptor kristjanssonii 177R1B
632516.3.peg.587	Caldicellulosiruptor lactoaceticus 6A	632292.3.peg.1994	Caldicellulosiruptor hydrothermalis 108
608506.3.peg.787	Caldicellulosiruptor obsidiansis OB47	632518.3.peg.693	Caldicellulosiruptor owensensis OL

```
632516.3.peg.587   caaaaagcaagctataattttctgtgctcaga-gatttattttttgatagctatcatttcgatattatgctaaaattaaaattg
632335.8.peg.2087  caaaaagcaagctataattttctgtgctcaga-gatttattttttgatagctatcatttcgatattatgctaaaattaaaattg
1121259.3.peg.2340 caaaaagcaagctataattttctgtgctcaga-gatttattttttgatagctatcatttcgatattatgctaaaattaaaattg
632292.3.peg.1994  caaaaaataagttataaattttttggcttaaa-gattt--tttacgatagctattatttcgatattatgctaaaattatagttg
608506.3.peg.787   ttaatgataagaaacaattttacttattaaaaagatcttacttgcgatag-tactttatcattaattatcgcaagttattga-a
632518.3.peg.693   ttgatgataagagacaattttacctgttaaaaagatcttacttgcgatag-tactttatcattacttatcgcaagttattga-a
                      *    ***  * ** ***      * * * *** *   **  ***** **   * **  ** *   *  ** ***

632516.3.peg.587   cggtaatccta-atttaaatttaaaaataattttttgtgtaatattgaaaacgatttctgcaatggtaaagaggtggttaccaATGACATTAAGGCT
632335.8.peg.2087  cggtaatccta-atttaaatttaaaaataattttttgtgtaatattgaaaacgatttctgcaatggtaaagaggtggttaccaATGACATTAAGGCT
1121259.3.peg.2340 cggtaatccta-atttaaatttaaaaataattttttgtgtgatattgaaaacgatttctgcaatggtaaagaggtggttaccaATGACATTAAGGCT
632292.3.peg.1994  tattaattcta-attcaaa-------ataattttttgtacaat-ttgaaaacgatttctgcatta-taaagaggtggttgccaATGACATTAACACT
608506.3.peg.787   cctcactcttgtatataagcttttcaataaaccttcg-atgtttttaaaa-aatacat---ataccaaagaggggattgctaATGGCACTTAGATT
632518.3.peg.693   ccttgtt-ttgtatataatcttttcaataaaccttcg-atgtttttaaaa-gatacat---atgcgaaagaggggattgctaATGGCACTTAGATT
                         *  *  **  **        ****   ** *     * ** ****  **   *    *       ******* * ***** ** ****  *
```

31899.10.peg.571 (Athe_0174) *xloEFG* XynR-binding site (repressor); AraR-binding site (potential activator)

31899.10.peg.571	Caldicellulosiruptor bescii strain DSMZ 6725	351627.8.peg.2610	Caldicellulosiruptor saccharolyticus DSM 8903
632348.3.peg.2522	Caldicellulosiruptor kronotskyensis 2002	632518.3.peg.126	Caldicellulosiruptor owensensis OL
1121259.3.peg.533	Caldicellulosiruptor acetigenus DSM 7040		

```
31899.10.peg.571  aatagtatcaaattt-------tgaaataagtaattataatattagat--------cgtagga-----tc
632348.3.peg.2522 aatagtatcaaatat-------tgaaataagtaattataatattaaat--------cttagga-----tc
1121259.3.peg.533 aatttttattcatttgttattactcgaaaaaacaacaaagttttaaat-----gggattaaaa----ttc
632518.3.peg.126  cattttgatgtatta--tattgtataatcgaaaaatctgttattaata-----accattagca----ttt
351627.8.peg.2610 ggtagaaattaatgtttacacaataatttcaataatatacttttgaaatgtgaactgtttgaaaacctat
                    *        **                    *      * **              *   *

31899.10.peg.571  aacttgttcacatacaagttttttaataaaatacacacaaaaaaatattgactagtaaacgcttacgata-tattatataaacAaaagaaagttg--atttttaat
632348.3.peg.2522 tacttgttcacatacaagttttt-aataaaatacacacaaaaaaatattgactagtaaacgcttacgata-tattatataaacaaaagaaagttg--atttttgat
1121259.3.peg.533 atgttgtatacgtacaacttttttaatataatgctactaaaaactattgactaatgaacgtttaagataatataatatagacgaaacataactt--accttttgg
632518.3.peg.126  aagttgtatatatacaacttttttaatgcaaagttac-acaaattattgacaaa-aaacgtttacagttatataatgaaatcacaataaatttaagatttttttgt
351627.8.peg.2610 aagttgtatacgtacaagttttttaatattaaattaaca-aaagtattgactttttaaaacacttaggtgtattatatagacaaaagaaattaaatagttttt--
                     ****  *  ***** ***** ***   *       * *** *******     **            *** **  *  *  ** * *      *  ***

31899.10.peg.571  --atattcaattttaaaaaaggttacaaaaccattta--------------------tataaagagtttataggtt-------------------aaactgc
632348.3.peg.2522 --atattcaattttgaaaaaggttacaaaaaaatttg--------------------tataaagagtttataggtt-------------------aaactgc
1121259.3.peg.533 -aatattagaatttgaaaaggctctcaacagtaattagtttagtaaaaattacgcttttaaaattgaccaaatcct--aaatgaatat---agtctaagtaa
632518.3.peg.126  taacaaaagaaattgaaaaggctctcaaacgaaagtatttatct-----tcatgtcttcaaaaatgcataaaacattgaattgtacattttggcataaaaac
351627.8.peg.2610 --ataattcaatttgaaaaggatttcaagagta------------------------taaaaaataataaaaattt-------------------tagttaa
                    * *    *  ** **** * *  ***    *                        *  ***      * *   *                    *

31899.10.peg.571  ctaatttcttaacaaatatagtttgggca---------aaattacgtataaatgt----------atcgaaggagatg-atagatTTGTCTGGGTTTTTAACA
632348.3.peg.2522 ctaatttcttaacaaatatagtttgggca---------aaattacggataaatgt----------atcgaaggagatg-atagatTTGTCTGGTTTTTTAACA
1121259.3.peg.533 gaaatgtata-aataacaaatcttctcaagctaattgtagtttaatgaaggaaaatgca--gcaaacttattaaaataaaaggagttgataaaaaTTGAATAG
632518.3.peg.126  aaagcctatccaacagcaaaatgtacata--taact-tattttgtagatgcaaaatgcgagggtatctaaacgaaaaataaggggttgattaaa-TTGAAAAG
351627.8.peg.2610 aacg----------aacaaaacatc-------------aatttgcaaaaacagaa-----------ctgaatacaaga--aggagttgataata-TTGAATAA
                                *  *  *   *              *  **    *   *                 *     *      *            **
```

31899.10.peg.2600 (Athe_2054) *axuCBA* Predicted AxuRS-binding site (potential repressor)

31899.10.peg.2600	Caldicellulosiruptor bescii strain DSMZ 6725	632335.8.peg.571	Caldicellulosiruptor kristjanssonii 177R1B
632348.3.peg.646	Caldicellulosiruptor kronotskyensis 2002	1121259.3.peg.2300	Caldicellulosiruptor acetigenus DSM 7040
1222016.3.peg.867	Caldicellulosiruptor changbaiensis strain CBS-Z	632516.3.peg.1050	Caldicellulosiruptor lactoaceticus 6A
351627.8.peg.1508	Caldicellulosiruptor saccharolyticus DSM 8903	608506.3.peg.1950	Caldicellulosiruptor obsidiansis OB47

```
1121259.3.peg.2300 tttatttttttgtgggtaagacaaaa-caaaactgtcgggtataacatgtcaaaatattggggtatgagtttttaatt
632516.3.peg.1050  tttatttttttgtgggtaagacaaaa-caaaactgtcgggtataacatgtcaaaatattggggtataagtttttaatt
632335.8.peg.571   tttatttttttgtgggtaagacaaaa-caaaactgtcgggtataacatgtcaaaatattggggtatgagtttttaatt
608506.3.peg.1950  tttcttcctttatctttaaattaatgtctaaactacagggtacaagatgtcaaaaaaatagggtatcattattttgtt
31899.10.peg.2600  gctttttatttttt-aaaaaacatggacaaaagtatagggtataacatgtcaaaatattagggtataaattttaTaat
632348.3.peg.646   gctttttattttttttaaaaaacatggacaaaagtatagggtataacatgtcaaaatattagggtataaattttataat
1222016.3.peg.867  tttttttgtttttt----taatgt---ctaaactgtagggtataagatgtcaaaaaaatagggtattgtgattttatt
351627.8.peg.1508  tttttttgtttttt----aaatgt---ctaaactgtagggtataagatgtcaaaaaaatagggtattgtaattttatt
                     * **  *** *              * *** *   ***** ** ********* * * ******     **    *
```

```
1121259.3.peg.2300 tttgattttagaatt-ataat-tgcaagcaagattcaaaaaaataaacagaaaaccttggaggtttataaATGAAT
632516.3.peg.1050  tttgattttagaatt-ataat-tgcaagcaagattcaaaaaaataaacagaaaaccttggaggtttataaATGAAT
632335.8.peg.571   tttgattttagaatt-ataat-tgcaagcaagattcaaaaaaataaacagaaaaccttggaggtttatatATGAAT
608506.3.peg.1950  tttcctttaaaattcattat-tg-atacagcagt--gagttttgatcaaaa---tttggagggcaaaaaATGAAC
31899.10.peg.2600  tttgactttaaaattatca----ttgaaaatcaagct-caaaaaaaaatac----cgtggaggt-ttataATGAAT
632348.3.peg.646   tttgattttaaaattatca----ttgaaaatcaagct-caaaaaaaaatac----cgtggaggt-ttataATGAAT
1222016.3.peg.867  tttcttttaaaatcatttatgtctgaacagtgggttatagagtaaagt--------tggaggtcttaaaATGAAC
351627.8.peg.1508  tttcttttaatattgtttatgtctgaacaataggcaacaaggtgaaatatat-ttttggaggctttaaaATGAAT
                   ***   ****  **               *               *           ******       *****
```

31899.10.peg.2602 (Athe_2056) *axuSR*

31899.10.peg.2602	Caldicellulosiruptor bescii strain DSMZ 6725	1222016.3.peg.865	Caldicellulosiruptor changbaiensis strain CBS-Z
632348.3.peg.644	Caldicellulosiruptor kronotskyensis 2002	1214564.3.peg.930	Caldicellulosiruptor sp. F32
632335.8.peg.569	Caldicellulosiruptor kristjanssonii 177R1B	351627.8.peg.1506	Caldicellulosiruptor saccharolyticus DSM 8903
1121259.3.peg.2302	Caldicellulosiruptor acetigenus DSM 7040	608506.3.peg.1955	Caldicellulosiruptor obsidiansis OB47
632516.3.peg.1048	Caldicellulosiruptor lactoaceticus 6A		

```
31899.10.peg.2602  TAAtttttt--attgcttatgtaaaaatatttaagttaaaataaaagTatagttaactcc-catgtggtgaaaaa-ATGAAAATATGGCTGACAAAC
632348.3.peg.644   TAAttttct--attgcttatgtaaaaatatttaagttaaaataaaagtatagttaactcc-catgtggtgaaaaa-ATGAAAATATGGCTGACAAAC
632335.8.peg.569   TGAttttttccattgctttgaaaaaattttaagctaaaatgaaaacatgaccagctttatgtgtggtgaaaggtATGAAAAGCCTTCTTACAAAT
632516.3.peg.1048  TGAttttttccattgctttgaaaaaattttaagctaaaatgaaaacatgaccagctttatgtgtggtgaaaggtATGAAAAGCCTTCTTACAAAT
1121259.3.peg.2302 TGAttttttccattgctttgaaaaaattttaagctaaaatgaaaacatgaccagctttatgtgtggtgaaaggtATGAAAAGCCTTCTTACAAAT
1222016.3.peg.865  TGAgtgttgaaaaaacagagt---------------tataattagaattaaagaaatctaatgggtga-ga-aaa-ATGAAACTTTTTTTTACAAAT
1214564.3.peg.930  TGAgtgttgaaaaaacagagt---------------tataattagaattaaagaaatctaatgggtga-ga-aaa-ATGAAACTTTTTTTTACAAAT
351627.8.peg.1506  TGAgtattgaaaac-cagagt---------------taaaattggaattaaagaaatcaaatgggtga-ga-aaagATGAAACTCTTTTTTGCAAAT
608506.3.peg.1955  TGAatattgacaaaata-------------------tataattaaaattaaagaaatcaaataggtga-gataaaaATGAGATTCTTTTTTGTAAAT
                   * * * *    *                        ** ***   *        *         ***  ** *   ****         *   ***
```

31899.10.peg.2603 (Athe_2057) *xylS* Predicted XylQ-binding site (potential activator)

31899.10.peg.2603	Caldicellulosiruptor bescii strain DSMZ 6725
632348.3.peg.643	Caldicellulosiruptor kronotskyensis 2002
632516.3.peg.1047	Caldicellulosiruptor lactoaceticus 6A
632335.8.peg.568	Caldicellulosiruptor kristjanssonii 177R1B
1121259.3.peg.2303	Caldicellulosiruptor acetigenus DSM 7040
1387557.3.peg.2452	Caldicellulosiruptor sp. Wai35.B1
1387569.3.peg.1838	Thermoanaerobacter cellulolyticus NA10
1214564.3.peg.929	Caldicellulosiruptor sp. F32
351627.8.peg.1505	Caldicellulosiruptor saccharolyticus DSM 8903
1222016.3.peg.864	Caldicellulosiruptor changbaiensis strain CBS-Z
1387555.3.peg.2157	Caldicellulosiruptor sp. Rt8.B8
608506.3.peg.1956	Caldicellulosiruptor obsidiansis OB47

```
31899.10.peg.2603  tttgta-ttgattatagcaaagtttttatactttttgcaaaaacgtatgagaagtaaggaaggaggttttatataataaa-tttG
632348.3.peg.643   tttata-ttgattatagcaaagtttttatactttttgcaaaaacgtatgagaagtaaggaaggaggttttatataataaa-tttg
632516.3.peg.1047  atggtacttgtttagagcaaggtatttatactttttgcaaaagagtatgagaagtaagaagagaggtcttgtataataagactag
632335.8.peg.568   atggtacttgtttagagcaaggtatttatactttttgcaaaagagtatgagaagtaagaaaagaagtcttgtataataagactag
1121259.3.peg.2303 atggtacttgtttagagcaaggtatttatactttttgcaaaagagtatgagaagtaagaaaagaggtcttttataataagactag
1214564.3.peg.929  tttata-ttgattagagcaaagtatttatactttttgcaaaaaagtatgagaagtaaaaaaaggggttttgtataataaaacttg
1222016.3.peg.864  tttata-ttgattagagcaaagtatttatactttttgcaaaaaagtatgagaagtaaaaaaagggattttgtataataaaacttg
1387569.3.peg.1838 tctata-ttgattagagcaaggtatttatactttttgcaatagagtatgagaagtgagaaaagaggttttatataataaaacttg
351627.8.peg.1505  tctata-ttgattagagcaaggtatttatactttttgcaatagagtatgagaagtaaaaaaagaggttttatataataaaacttg
1387557.3.peg.2452 gatataattta-----gcaatgtatttatatttttttgcaatatggttggagaaataattt--------ttgttttgtggc-----
1387555.3.peg.2157 gatacaattta-----gcaatgtatttatatttttttgcaatatggttaaagaaataattt--------t-gtttta-agt-----
608506.3.peg.1956  ctttcaattgctcaaagcaaagtatttatactttttgcaaaagagtatgagaagtaatataaaaagcattgtataataacacttg
                        * **       **** ** ****** ********** *  **   **** * *           *  * *

31899.10.peg.2603  agtgttttagacctctttaaaatttctacgaataaacaattggttaaaaacaagta-ggggg--aattaacaaaATGAAATT
632348.3.peg.643   agtgttttagacctcttataaatttctacgaatagacaattggttaaaaacaagtaaggggg--aattaacaaaATGAAATT
632516.3.peg.1047  attatttccaaacttctc---------ttaaacaatgatccattttcactctaaataagggggaattacttgaaATGAAATT
632335.8.peg.568   attatttccaaacttctc---------ttaaacaatgatccattttcactctaaataagggggaattacttgaaATGAAATT
1121259.3.peg.2303 attatttccaaacttctc---------tttaacaatgatccattttcactctaaataagggggaattacttgaaATGAAATT
1214564.3.peg.929  attgttttggacctcttt--------tataaataaataatcgtttcaaaccaagtaagggaggaaatttacaaaATGAAATT
1222016.3.peg.864  attgttttggacctcttt--------tataaataaataatcgtttcaaaccaagtaagggaggaaatttacaaaATGAAATT
1387569.3.peg.1838 attgtttctaaatttttc---------ataagcaataactgtttttaaactaagaaaagggggaaattaacaaaATGAAATT
351627.8.peg.1505  attgtttctaaacttttc---------ataaacaataactgtttttaaactaagaaaagggggaagttaacaaaATGAAATT
1387557.3.peg.2452 -ttatttaatata--------------gtaggtacctaatattttt---ctggaggggaaaa--------caaaATGAAATT
1387555.3.peg.2157 -ttattttttata--------------gtagatacctgatattttt---ctggaggg-agaa--------caggATGAAATT
608506.3.peg.1956  attgttcttcatctcttt---------acaaagcaataattttttttattctaaaggagaggg--attgttaaaATGAAATT
                     * **    *                                **                             ********
```

31899.10.peg.2604 (Athe_2058) *xylQ* Predicted XylQ-binding site (potential activator)

31899.10.peg.2604	Caldicellulosiruptor bescii strain DSMZ 6725	1121259.3.peg.2304	Caldicellulosiruptor acetigenus DSM 7040
632348.3.peg.642	Caldicellulosiruptor kronotskyensis 2002	632335.8.peg.567	Caldicellulosiruptor kristjanssonii 177R1B
1387569.3.peg.1839	Thermoanaerobacter cellulolyticus NA10	632516.3.peg.1046	Caldicellulosiruptor lactoaceticus 6A
1222016.3.peg.863	Caldicellulosiruptor changbaiensis strain CBS-Z	632518.3.peg.1849	Caldicellulosiruptor owensensis OL
1214564.3.peg.928	Caldicellulosiruptor sp. F32	608506.3.peg.1957	Caldicellulosiruptor obsidiansis OB47
351627.8.peg.1504	Caldicellulosiruptor saccharolyticus DSM 8903		

```
31899.10.peg.2604  attattaatagataaaaa-gcttagataaaaagctgccgcaagttttggat-tgcggcagcttttattttgttgtggttgtggtaaaattta
632348.3.peg.642   gaaaaagtaaaatattat-taatagataaaaagctgccgcaagttttagat-tgcggcagcttttattttgttgtggttatggtaaaattta
608506.3.peg.1957  gaaaaaataaaatattat-cagtggatgaaaagctgccgcaagttttagat-tgcggcagcttttattttgttgtggttgtggtaaaattta
632335.8.peg.567   ataaataagaatatcgag-agatgaa-aatgagctgccgcaattttttgaaaaagcggcagcttttattttgttgtagttgtgttaaaatcta
632516.3.peg.1046  ataaataagaatatcgag-agatgaa-aatgagctgccgcaattttttgaaaaagcggcagcttttattttgttgtagttgtgttaaaatcta
1121259.3.peg.2304 ataaataagaatatcgag-agatgaa-aatgagctgccgcaattttttgaaaaagcggcagcttttattttgttgtagttgtgttaaaatcta
632518.3.peg.1849  aaaataaagtattttcat-atatagataaaaagctgctgcaatcta-aaatatgcggcagcttttattttttta-ggttatggtaaaattta
1222016.3.peg.863  gaaaaaataaaatattat-caatggatgaaatgctgccgcaagttttagat-tgcggcagcttttattttgttgtgcttgtggtaaaattta
351627.8.peg.1504  gaaaagataaaatattattcaataggtgaaatgctgccgcaaattttggtc-tgcggcagcttttattttgttgtgcttgtggtaaaattta
1387569.3.peg.1839 gaaaagataaaatattattcaataggtgaaatgctgccgcaaattttggtc-tgcggcagcttttattttgttgagcttgtggtaaaattta
1214564.3.peg.928  gaaaagataaaatattattcaataggtgaaaagctgccgcaaattttggtc-tgcggcagcttttattttgttgagcttgtggtaaaattta
                      *            *     *         ***** ****           ***************** ***   ** ** ****** **

31899.10.peg.2604  tttGatattaccctattgttttt-attgaaaacatacaat-aaagtgaggaaatcacaATGGATGAAAGGTTGAGGCTCAAA
632348.3.peg.642   tttgatattaccctattgttttt-attgaaaacatacaat-aaagtgaggaaatcacaATGGATGAAAGGTTGAGGCTCAAA
608506.3.peg.1957  tttgatactaatgtattgttttt-attgaaaacatacaat-aaagtgaggaaatcacaATGGATGAAAGACTGAGGCTAAAA
632335.8.peg.567   attgagactcatatattgctttt-gtttaaaacatacaat-aaagtggggaaatcacaATGGATGAAAGACTAAAGCTCAAA
632516.3.peg.1046  attgagactcatatattgctttt-gtttaaaacatacaat-aaagtggggaaatcacaATGGATGAAAGACTAAAGCTCAAA
1121259.3.peg.2304 attgagactcatatattgctttt-gtttaaaacatacaat-aaagtggggaaatcacaATGGATGAAAGACTAAAGCTCAAA
632518.3.peg.1849  -tttataattgtgtattatttttttgttaagaacatacaattaaagtggtgaaattgcaATGGATGAAAGGCTGAGACTCAAA
1222016.3.peg.863  tttaagattaatatattgctttt-attgaaaacatacaaa-aaagtggggaaattgtaATGGATGAAAGGTTGAGACTCAAA
351627.8.peg.1504  tttgagataaatacattgcattt-gttgaaagtatacaaa-aaagtggggaaattgcaATGGATGAAAGGTTGAGGCTCAAA
1387569.3.peg.1839 ttagagataaatacattgcattt-gttgaaaacatacaaa-aaagtggggaaattgcaATGGATGAGAGGTTGAGACTCAAA
1214564.3.peg.928  ttagagataaatacattgcattt-gttgaaaacatacaaa-aaagtggggaaattgcaATGGATGAGAGGTTGAGACTCAAA
                    *  * *       ***   ***  ** * *  ******  ******  *****   ********* **  * *  ** ***
```

31899.10.peg.2974 **(Athe_2392)** ***uxaRKHGF*** **Predicted UxaR-binding site** **(repressor)**

31899.10.peg.2974	Caldicellulosiruptor bescii strain DSMZ 6725	632518.3.peg.2278	Caldicellulosiruptor owensensis OL
632348.3.peg.147	Caldicellulosiruptor kronotskyensis 2002	1387557.3.peg.2824	Caldicellulosiruptor sp. Wai35.B1
632292.3.peg.135	Caldicellulosiruptor hydrothermalis 108	608506.3.peg.2321	Caldicellulosiruptor obsidiansis OB47
632335.8.peg.2151	Caldicellulosiruptor kristjanssonii 177R1B	351627.8.peg.403	Caldicellulosiruptor saccharolyticus DSM 8903
632516.3.peg.1531	Caldicellulosiruptor lactoaceticus 6A	1387569.3.peg.576	Thermoanaerobacter cellulolyticus NA10
1121259.3.peg.2130	Caldicellulosiruptor acetigenus DSM 7040		

```
31899.10.peg.2974  --ttgaccaatcttgtgtaatcattttgaatttcatattgcaaatcctcaaaaaatcgagtagaatatta
632335.8.peg.2151  --ttgaccaatcttgtgtaatcattttgaatttcatattgcaattcctcaaaaaatcgagtagaatatta
632516.3.peg.1531  --ttgaccaatcttgtgtaatcattttgaatttcatattgcaattcttcaaaaaatcgagtagaatatta
632348.3.peg.147   --ttgaccaatcttgtgtaattattttgaattttatattgcaattcctcaaaaaattgagtagaatatta
632292.3.peg.135   --ttgaccaatcttgtgtaatcattttgaatttcatattgcaattcctcaaaaaattgagtagaatatta
1121259.3.peg.2130 --ttgaccaatcttgtgtaatcattttgaattttgtattgcaattcctcaaaaaatcgagtagaatatta
632518.3.peg.2278  --ttgaccaatcttgtgtaattatttgaatttcatattgcaaatcctcaaaaaattgagtagaatatta
608506.3.peg.2321  --ttgaccaatcttgtgtaatcattttgaatttcatattgcaaatcctcaaaaaatcgagtagaatatta
1387557.3.peg.2824 --ttgactaatcttgtgtaattcctttgaattttatattgcaattcctcaaaaaatcgagtagaatatta
351627.8.peg.403   tcttgaccaatcttgtgtaattcttttgaatttcatattgcaattcctcaaaaaattgagtagaatatat
1387569.3.peg.576  tcttgaccaatcttgtgta-tttttttgaatttcatattgcaattcctcaaaaaattgagtagaataata
                     ***** *********** *   *********  ******** ** ********* **********
```

```
31899.10.peg.2974  aTaagctgacacacaaatccacatataa-gaaataggtgatgaagagaaATGCTTTCGGCAACG
632335.8.peg.2151  gtaagctgacacacaaatccacatataa-gaatagggtgatga-ggtttATGTTATCAGCAACG
632516.3.peg.1531  gtaagctgacacacaaatccacatataa-gaatagggtgatga-ggtttATGTTATCAGCAACA
632348.3.peg.147   ataagctgacacacaaatccacatgtaa-gaaataggtgatgaagagagATGCTTTCGGCAACG
632292.3.peg.135   ataagctgacacacaaatccacatgtaa-gaaataggtgatgaagagagATGCTTTCGGCAACG
1121259.3.peg.2130 gtaagctgacacacaaatccacatataa-gaa-taggtgatgaagagttATGTTATCAGCAACA
632518.3.peg.2278  ttaagttgacacacaaatccacatataa-aaa---ggtgatgt-gaaagATGCTTTCAGCAACG
608506.3.peg.2321  ataagctgacacacaaatccacatataaagagatgggtgatgaaaagagATGCTTTCAGCAACA
1387557.3.peg.2824 ataagcttacacacaaatccacatgtaa--gaataggtgatgaagagagATGCTTTCGGCAACG
351627.8.peg.403   ataagttgacacacaaatccacatagaa--gaataggtgATGA-AAGGTATGCTCTCTGCAACA
1387569.3.peg.576  gcaagttgacacacaaatccacatagaa--gaataggtgatga-aaggtATGCTCTCTGCAACC
                     *** * ****************  **        ****          *** * ** *****
```

31899.10.peg.1027 (Athe_0594) *celD* Predicted AviRS-binding site (potential activator)

31899.10.peg.1027	Caldicellulosiruptor bescii strain DSMZ 6725	632335.8.peg.2173	Caldicellulosiruptor kristjanssonii 177R1B
632348.3.peg.2151	Caldicellulosiruptor kronotskyensis 2002	632516.3.peg.1550	Caldicellulosiruptor lactoaceticus 6A
1387557.3.peg.849	Caldicellulosiruptor sp. Wai35.B1	632518.3.peg.511	Caldicellulosiruptor owensensis OL
632292.3.peg.2161	Caldicellulosiruptor hydrothermalis 108	608506.3.peg.606	Caldicellulosiruptor obsidiansis OB47
1121259.3.peg.804	Caldicellulosiruptor acetigenus DSM 7040		

```
31899.10.peg.1027  ttttgcaaaatttttaccttatcttagaacaagatgctacaaaaagcaggtcaaaaatttcaaacagatatgaaatttttttaatt
632348.3.peg.2151  ttttgcaaaatttttaccttatcttagagcaagatgctacaaaaagcaggtcaaaaatttcaaacagatatgaaatttttttaatt
632292.3.peg.2161  ttttgcaaaatttttaccttgtcttagagcaagatgctacaaatatcaggtcaaaaatttaaaacagatatgaaatttttttaatt
632335.8.peg.2173  ttttgcaaaatttttaccttgtcttagagcaagatgctacaaatatcaggtcaaaaatttcaaacagatatgaaatttttttaatt
632516.3.peg.1550  ttttgcaaaatttttaccttgtcttagagcaagatgctacaaatatcaggtcaaaaatttcaaacagatatgaaatttttttaatt
1121259.3.peg.804  ttttgcaaaatttttaccttgtattagagcaagatgctacaaatatcaggtcaaaaatttaaaacagatatgaaatttttttaatt
1387557.3.peg.849  ttttgcaaaatttttaccttgtcttagagcaaaatgctacaaatatcagttcaaaaattcaaaacatatatgtaatttttttaatt
632518.3.peg.511   ttttgaaaaatttttaacttttcataaagcaaaatgctataaatatcaggtcaaaaatttaaaacagatatgaaatttttttaatt
608506.3.peg.606   ttttgcaaaatttttaccatgtcttagagcaagatgttacaaatatcagatcaaaaatttaaaacagatatgaaagttttttagtt
                   ***** ********** * * *  ** * *** *** ** *** * *** *********  ***** ***** ** ******* **

31899.10.peg.1027  taaaactttctg-agcatggaatatattaaacTtat-------aacgagatgataacgattacata-ggagggatttgcattATGAGGAAAATT
632348.3.peg.2151  taaaactttctg-agcatggaatatattaaacttat-------aacaagatgataacgattacata-ggagggatttgcattATGAGGAAAATT
632292.3.peg.2161  taaaacttcctg-agcatggaatatattaattttat-------aacaaaatgataacgattacata-ggagggattcacattATGAGAGAAATT
632335.8.peg.2173  taaattctccaa-agaatgaattatattaatttcaa-------aagaaaatgataacgattacata-ggaggtattcacattATGAAAAAGATT
632516.3.peg.1550  taaattctccaa-agaatgaattatattaatttcaa-------aagaaaatgataacgattacata-ggaggtattcacattATGAAAAAGATT
1121259.3.peg.804  taaaacttccta-agcatggattatattaatcttat-------aacaaaatgataacgattacatg-ggagggattcacattATGAGAGGAATT
1387557.3.peg.849  taaattttccat-ggtgtggattatattaattttcg-------aacaaaatgataacgattacata-ggagggatttgtattATGCGAAAGATT
632518.3.peg.511   ttaaacttcata-tgtataatgtatattaatctcatg------aattaaatgataacgattacacaaggagggactcacattATGAAGAAAATT
608506.3.peg.606   cactatttcctatagtatggattatattatttatacactatataacaaaatagtaatgattaaagg-ggaggaaatacaattATGAAAAGAACT
                          *      *  *    *******              **  * **  *** ***** *   ***** * *   ******      * *
```

31899.10.peg.1029 (Athe_0595) *aviABC* Predicted AviRS-binding site (potential activator)

31899.10.peg.1029	Caldicellulosiruptor bescii strain DSMZ 6725	632335.8.peg.2171	Caldicellulosiruptor kristjanssonii 177R1B
632348.3.peg.2149	Caldicellulosiruptor kronotskyensis 2002	632516.3.peg.1548	Caldicellulosiruptor lactoaceticus 6A
632292.3.peg.2159	Caldicellulosiruptor hydrothermalis 108	632518.3.peg.512	Caldicellulosiruptor owensensis OL
1387557.3.peg.850	Caldicellulosiruptor sp. Wai35.B1	1387569.3.peg.2107	Thermoanaerobacter cellulolyticus NA10
608506.3.peg.607	Caldicellulosiruptor obsidiansis OB47	1222016.3.peg.2475	Caldicellulosiruptor changbaiensis strain CBS-Z
1121259.3.peg.806	Caldicellulosiruptor acetigenus DSM 7040	351627.8.peg.783	Caldicellulosiruptor saccharolyticus DSM 8903

```
31899.10.peg.1029  taaatgctggcagctttt--tt-atattattttgacccttcaatatgaca
632348.3.peg.2149  -aaatgctggcagctttt--tttatattattttgacactcttgtatgaca
1387557.3.peg.850  ----tgctggcagctatt--tttatattattttgaccctttaatatgaca
632518.3.peg.512   -----actggcagctatt--tttatattattttgacccttcaatatgaca
608506.3.peg.607   -----gctggcagctatt--tttatattattttgacccttcaatatgaca
632292.3.peg.2159  ----tgctggcagctatt--tttatattattttgacactccaatatgaca
632335.8.peg.2171  -aaatgtcggcagctatt--tttatattattttgaccattcaatatgaca
632516.3.peg.1548  -aaatgtcggcagctatt--tttatattattttgaccattcaatatgaca
1121259.3.peg.806  -aaatgccggcagctatt--tttatattattttgaccattcaatatgaca
1387569.3.peg.2107 -tgccttctacatttttaatttatactttttgactgcttt-taaatca
351627.8.peg.783   -tgccttctacatttttaatttatactttttgactgcttt-taaatca
1222016.3.peg.2475 -tgccttctacatttttaatttatactttttgactgcttt-taaatca
                             **  * **  ** *** * *******       **   **

31899.10.peg.1029  aaatattgcacattttaatatcaacatttcacaaaaaactgcaattttttcaattcattttgaaaaatataggttgtaaact
632348.3.peg.2149  aaatattgcacattttaatatcaacatttcacaaaaaactgcaattttttcaattcattttgaaaaatataggttgtaaact
1387557.3.peg.850  aaatattgcacattttaatatcaacaattcacaaaaagactgcaattttgtcaattcatttttagaaaaataagctgtaaact
632518.3.peg.512   aaatattgcacattttaatatcaacaattcacaaaaagactgcaattttgtcaattcattttgagaaaaataggatgtaaact
608506.3.peg.607   aaatattgcacatcataatatcaataattcacaaaaagactgcaattttgtcaattcattttgagaaaaacaggatgtaaact
632292.3.peg.2159  aaatattgcacattttaatatcaacaattcacaaaaagactgcaattttgtcaattcattttgagaaaaataggatgtaaact
632335.8.peg.2171  aaatattgcacattttaatatcaacaattcacaaaaagactgcaattttgtcaattcattttgaagaaaataggatgtaaact
632516.3.peg.1548  aaatattgcacattttaatatcaacaattcacaaaaagactgcaattttgtcaattcattttgaagaaaataggatgtaaact
1121259.3.peg.806  aaatattgcacattttaatatcaacaattcacaaaaaactgcaattttatcaattcattttgaaaaaaataggatgtaaact
1387569.3.peg.2107 aaataatgcaaatttacatatcaataattaacaaaaagaatgcaattttatcaattcatttttataagtaagattgtaaact
351627.8.peg.783   aaataatgcaaatttacatatcaataattaacaaaaagaatgcaattttatcaattcatttttataagtaagattgtaaact
1222016.3.peg.2475 aaataatgcaaatttatatatcaataattaacaaaaagaatgcaattttatcaattcatttttataagtaggattgtaaact
                   ***** **** **    ******* * ** ******* * ********* ************   **       ********

31899.10.peg.1029  ataattGaaaagttcaaagaa----caattcaaaagtttcaataaacaa-cttgagaaaggagacggcatcagaaATGGCAAA
632348.3.peg.2149  atagttgaaaagttcaaagaa----caattcaaaagtttcaataaacaa-cttgagaaaggagacggcatcagaaATGGCAAA
1387557.3.peg.850  ataatcgacaag-tcaagaaaaaactaagtcaaaagtttcaataaaaaa-cttgagaaaggagacggcatcaaaaATGGCAAA
632518.3.peg.512   ataatcgaaaacctcaaaaaaaaactaagtcaaaagtttcaataaaaaa-cttgagaaaggagacggcatcaaaaATGGCAAA
608506.3.peg.607   ataatcgaaaacctcaagaaaaa-ccaagtcaaaagtttcaataaaaaaactcgagaaaggagacggcatcaaaaATGGCAAA
632292.3.peg.2159  ataatcgaaaacctcaagaaaaa-ctaagtcaaaagtttcaataaaaaa-gttgagaaaggagacggcatcaaaaATGGCAAA
632335.8.peg.2171  ataatcgaaaagttcaacaag----caaatcgaaagtttcaataaacaa-cttgagaaaggagacggcatcaaaaATGGCAAA
632516.3.peg.1548  ataatcgaaaagttcaacaag----caaatcgaaagtttcaataaacaa-cttgagaaaggagacggcatcaaaaATGGCAAA
1121259.3.peg.806  ataatcgaaaagttcaacaag----taaatcgaaagtttcaataaacaa-cttgagaaaggagacggcatcaaaaATGGCAAA
1387569.3.peg.2107 atagttgaaaa--tcaaactcagcacaagg-gaaatttccaaagatttaacat-agaaaggga--acatgcaagATGAGAAG
351627.8.peg.783   atagttgaaaa--tcaaactcagcacaagg-gatatttccaaagatttaacat-agaaaggga--acatgcaagATGAGAAG
1222016.3.peg.2475 atagttgaaaa--tcaaactcagcgcaagg-gaaatttccaaagacttaacat-agaaaggga--acatgcaagATGAGAAG
                   *** * ** **  ****         **    * * ** ***  *   *     ******* **   ***   * ***  **
```

31899.10.peg.1032 (Athe_0599) *aviD-aviSR*

31899.10.peg.1032	Caldicellulosiruptor bescii strain DSMZ 6725	632516.3.peg.1545	Caldicellulosiruptor lactoaceticus 6A
632348.3.peg.2146	Caldicellulosiruptor kronotskyensis 2002	608506.3.peg.610	Caldicellulosiruptor obsidiansis OB47
632292.3.peg.2156	Caldicellulosiruptor hydrothermalis 108	632518.3.peg.515	Caldicellulosiruptor owensensis OL
1121259.3.peg.809	Caldicellulosiruptor acetigenus DSM 7040	1222016.3.peg.2472	Caldicellulosiruptor changbaiensis strain CBS-Z
632335.8.peg.2168	Caldicellulosiruptor kristjanssonii 177R1B	351627.8.peg.787	Caldicellulosiruptor saccharolyticus DSM 8903

```
31899.10.peg.1032  tcccgtttgcttctaatatt-aagaaaaaaatggattgtaattttatattatt-tgagctaaaataaaaaTaaaagtgt
632335.8.peg.2168  tcccgtttattgctaatattagaaaagaaatagattgtatttactgggcgat-tagggtaaaataaaag-aaaaacgt
632516.3.peg.1545  tcccgtttattgctaatattagaaaagaaatagattgtatttactgggcgat-tagggtaaaataaaag-aaaaacgt
632348.3.peg.2146  tcccgtttgcttctaatatt-cagaaaaaaatggattgtaattttatattatt-tgagctaaaatagaagtaaaaatgt
632292.3.peg.2156  tcccgtttgttgctaatatttgaaaagaaatagattgtgattttgtatcaat-tgagctaaaataaaagtaaaagtgc
1121259.3.peg.809  tcccgtttgttgctaatatttgaaaagaaatagattgtgattttatctcaat-taagataaaataaaagtaaaaacgt
608506.3.peg.610   tcccgtttgttgctaatatttgaaaagaaatagattgtgattttatctcaat-taagataaaataaaagtgaaaatat
632518.3.peg.515   tcccgtttgttactaatat---gtgaaaaatgtcttgtgagttaatctcgat-taagataaaataaagataaaaattt
1222016.3.peg.2472 tcccgtttgttactaatattagtcaattaattaaattgattggggcatgaaagtaaggtaaaatataattaaaaatgt
351627.8.peg.787   tcccgtttgttactaatattagtcaattaattaaattgattggagtatgaaagtaaggtaaaatataattaaaaatgt
                   ********  * *******      *  ***    *                 *  * ******* *    ***

31899.10.peg.1032  g--ttatattt-aaaatctttagggttgtgattttttATGAATGACTCATTCAATAAAAGGCG
632335.8.peg.2168  a--tcatgctgatgcagatatagggttgtgattttt-ATGAAAAATGCTTTTGATAAAAGACA
632516.3.peg.1545  a--tcatgctgatgcagatatagggttgtgattttt-ATGAAAAATGCTTTTGATAAAAGACA
632348.3.peg.2146  g--ttatattt-aaattctttagggttgtgattttttATGAATGATTCATTCAATAAAAGGCG
632292.3.peg.2156  a--tcatattt-aaagtttttagggttgtgattttt-ATGAAAGATTCATTTAATAAAAGACA
1121259.3.peg.809  a--ttatacttcaaaaaattcagggttgtgattttt-ATGAAAGATTCATTTAATAAGAGGCA
608506.3.peg.610   a--ttatactt-aaagaattcagggttgtgattttt-ATGAAAGATTCTTTTAATAAAAGACA
632518.3.peg.515   a--ttatgctt-taaggatacagggttgtgattttt-ATGAAAGATACATTCAATAGAAAACA
1222016.3.peg.2472 aagttattcaagcaagagtgtaaggttgggattttaATG---------TTTAGTAAAAAGAA
351627.8.peg.787   aagttattcaagcaagagtgtaaggttgggattttaATG---------TTTAGTAAAAAGAA
                      * **           *  * ***** ******* ***         **   **  *
```

31899.10.peg.2574 (Athe_2028) *lplD*

31899.10.peg.2574	Caldicellulosiruptor bescii strain DSMZ 6725
632348.3.peg.751	Caldicellulosiruptor kronotskyensis 2002
632292.3.peg.853	Caldicellulosiruptor hydrothermalis 108
608506.3.peg.1923	Caldicellulosiruptor obsidiansis OB47
1121259.3.peg.2279	Caldicellulosiruptor acetigenus DSM 7040
351627.8.peg.2971	Caldicellulosiruptor saccharolyticus DSM 8903
632335.8.peg.592	Caldicellulosiruptor kristjanssonii 177R1B
632518.3.peg.1799	Caldicellulosiruptor owensensis OL
632516.3.peg.1071	Caldicellulosiruptor lactoaceticus 6A
1222016.3.peg.900	Caldicellulosiruptor changbaiensis strain CBS-Z

```
31899.10.peg.2574   agaattgtca-atatctttttaggatgcacttttgaaaagtcaaagaaaaaaccagatataatagga--ataaatagaagttaaaga
632348.3.peg.751    agaattgtca-atatctttttaggatgcacttttgaaaagtcaaagaaaaaaccagatataatagga--ataaataaaagttaaaga
632335.8.peg.592    agaattgtca-atatctttttaggatacacttttgaaaagttaaagaaaaaacaagatatactgggt--ataaattaaatttaaaga
632516.3.peg.1071   agaattgtca-atatctttttaggatacacttttgaaaagttaa--aaaaaacaatatatactgggt--ataaattaaatttaaaga
1121259.3.peg.2279  agaattgtca-atatctttttaggatacacttttgaaaagttaaagaaaaaacaagatatactgggt--ataaattaaatttaaaga
632292.3.peg.853    agaattgtca-acatctttttaggatacacttttgaaaagttaaagaaaaaaacagaaatactggag--ataaataaaagttaaaga
608506.3.peg.1923   agaattgtca-atatctttttaggatacacttttgaaaagtcaaagaaaaaaccagatatgctgggt--ataaatgaaagttaaaga
1222016.3.peg.900   tactttgtca-accccatcttgaattgtatcattagaattacttaaaactctcaagtagcattatgttaacaaaatcaattttttac
351627.8.peg.2971   agaattgtca-atatctttttaggatgcacttttgaaaagccaaagaaaaaaccagatataatagga--ataaataaaagttaaaga
632518.3.peg.1799   gtcttcagcatatgcctttttaggatagacttttgaaaagtc----aaaaaaccagatatactgggc--ataaatgaaagttaaaga
                        *   ** *   * * **    *  *   **  **        **      *       *      * ***   ** **

31899.10.peg.2574   taataatggtttggttttaagtgagatttgtgatataatttattTgaaattcacaattattataggagggagtaataTTGAATACCAC
632348.3.peg.751    taataatggtttggttttaagtgagatttgtgatataatttatttgaaattcacaattattataggagggagtaataTTGAATACCAC
632335.8.peg.592    taataatggtttggtattgagtaagatttgtgatataatttatttgaaattcacaattattataggagtgagtaATGTTGAATACCAC
632516.3.peg.1071   taataatggtttggtattgagtaagatttgtgatataatttatttgaaattcacaattattataggagtgagtaATGTTGAATACCAC
1121259.3.peg.2279  taataatggtttggttttgagtaagatttgtgatataatttatttgaaattcacaattattataggagtgagtaATGTTGAATACCAC
632292.3.peg.853    taataatagtttggttttaagtgagatttgtgatataatttatttgaaatttacaattattataggagggagtaATGTTGAATACCAC
608506.3.peg.1923   taataatggtttggttttgagtgagatttgtgatataatttatttgaaattcacaattattataggagggagtaATGTTGAATACCAC
1222016.3.peg.900   ttaatattgtttggatttatcctattttgtgatataatgtatttgaaattttcaaatattataggagggagttATGTTGAATACCAC
351627.8.peg.2971   taataatggtttggttttaagtgagatttgtgatataatttatttgaaattcacaattattataggagggagtaataTTGAATACCAC
632518.3.peg.1799   taataatggtttggttttgagtgagatttgtgatataatttatttgaaattcacaattattataggagggagtaATGTTGAATGCCAC
                    * *  ** ******  ** *      ************* ***********  *** *********** **** ** ****** ****
```

31899.10.peg.2578 (Athe_2033) *pecR2* Predicted novel palindromic TF binding site (motif 2) (potential activator)

31899.10.peg.2579	Caldicellulosiruptor bescii strain DSMZ 6725	632292.3.peg.785	Caldicellulosiruptor hydrothermalis 108
632348.3.peg.667	Caldicellulosiruptor kronotskyensis 2002	fig|1387557.3.peg.2428	Caldicellulosiruptor sp. Wai35.B1
608506.3.peg.1929	Caldicellulosiruptor obsidiansis OB47		

```
632292.3.peg.785   CTTGAAGAATAA-ggctatttacctattgttttgaagacacagtataaaaaggctgcggggatataaataaccctgcag
1387557.3.peg.2428 CTTGAAGAATAGaggcattttacatattattttgaa-atatgatataaaaaggctgcaggcatgtaa-caggcttgcag
608506.3.peg.1929  TTAGAGGAGTAAaa--ttttaataaataaatttaaa----------------gccacaggga-atgatgtcctctgtgg
632348.3.peg.667   CTTGAAGAATAAggctttttttacccattgttaggatgacacaggatgaaa-ggctgcgggaataaagataatcctgcag
31899.10.peg.2579  CTTGAAGAATAAggctttttttactcattgttaggatgacacaggatgaaa-ggctgcgggaataaagacaatcctgcag
                    * ** ** **       ** *   **   *   *                 **  * ** *            **  *
```

```
632292.3.peg.785   -ccttttattacatataataccagtaaacacaacagtaaaaagtatttatttaatttatagcctaaagagaaataaaatagtcatgt
1387557.3.peg.2428 -ccttttattacatataataccagcaaacacgacagtaaaaagtatttatttaatttataggttgaagtgaaataaaatagtcatgt
608506.3.peg.1929  cttttttgttacaagaattaccaataaacataacagtaaaaagtattttttaatttgtaccctcgagagaaataaaatagtcatat
632348.3.peg.667   -ccttttattacatttaataccagtagacacaacagtaaaaagtatttatttaatttatagcctaaagtgaaacaaaatagtcatgt
31899.10.peg.2579  -ccttttattacatttaataccagtagacacaacagtaaaaagtatttatttaatttatagcctaaagagaaacaaaatagtcatgt
                      **** *****   * *****  * ***  **************** ******** **   *  ** **** *********** *
```

```
632292.3.peg.785   ataacaaaacaaaaaatgaatttagaaaaatatttattgagattgact-taaattatatcttgaattaatatattaactatga
1387557.3.peg.2428 ttaacaaaactaaaaataaattttgacaaatatttattgagattgact-taaattatatcttgaattaatatattaactgtga
608506.3.peg.1929  taaacaaaaataaagttaatctttaaataatatttattgaggttgact-tatatattgccatgatttaatatattatttgtaa
632348.3.peg.667   ttaacaaaacgaaaaataaatatataaaaatatttattgagattgact-taaattgtatcttgaattaatatataaactATGA
31899.10.peg.2579  ttaacaaaacaaaaaataaatatataaaaatatttattgaactgaaaagtattctataaccccattgatagtgttagg-acgc
                     *******  ***  * *   *  *  ************  *  *   **     *  *   * * *   * * *
```

31899.10.peg.2578 (Athe_2032) *pecXYZ-pecX2* Predicted PecR2-binding site (potential activator)

31899.10.peg.2578	Caldicellulosiruptor bescii strain DSMZ 6725	608506.3.peg.1928	Caldicellulosiruptor obsidiansis OB47
632348.3.peg.668	Caldicellulosiruptor kronotskyensis 2002	632518.3.peg.1822	Caldicellulosiruptor owensensis OL
632292.3.peg.786	Caldicellulosiruptor hydrothermalis 108	1387557.3.peg.2423	Caldicellulosiruptor sp. Wai35.B1

```
31899.10.peg.2578  tgtttttctataaaactgtaccttgattttaaaaagtgaatgcaccttaaactaagaaattgttgccctttctaaatagttgaa
608506.3.peg.1928  tgtttttctataaaaatgtacattgattttaaaaagtgaatgtgtaccagcctaagaaattgttgctctttctaaatagttgaa
632518.3.peg.1822  tgtttttctataaaaatgtaccttgattttaaaaagtgaatgtgtaccagcctaagaaattgttgctctttctaaatagttgaa
632348.3.peg.668   tgtttttctataaaattgtaccttgattttaaaaagtaaatgtatcctgacctaagaaattgtttccctttctaaatagttgaa
632292.3.peg.786   tgtttttctataaaagtgtaccttgattttaaaaagtgaatgtgcgttagcctaagaaattgttgccctttctaaatagttgaa
1387557.3.peg.2423 tgtttttctataaaagtgtaccttgattttaaaaagtgaatgtgcattagcctaataaattgttaccctttctaaatagttgaa
                   *************** ***** *************** ****          **** ******** * *****************
```

```
31899.10.peg.2578  agttgataaaacatagctcttactgtaacttaggaatcaaaaaaaataaacaaaaaaagaggaggctaagtttgATGTTTGGTAAAAA
608506.3.peg.1928  agttgataaaatatgcaccttgctatagatttgaaactagaaaaaattaacggagaataaggaggctaaattcaATGTTCGGCAAAAA
632518.3.peg.1822  agttgataaaatatgctccttgctatagatttgaaactagaaaaaattaacgaagaataaggaggctaaattcaATGTTCGGCAAAAA
632348.3.peg.668   ggttgataaaacatggctcttgcagtaacctaaaaatcagaaaaaataaacgagaaaaaaggaggctaaattcgATGTTCGACAAAAA
632292.3.peg.786   agttgataaaacatacgtcttgctgtaacttaaaaatcagaaaaaataaacgaaaaaagaggaggctaaattcgATGTTCGACAAAAA
1387557.3.peg.2423 ggttgataaaacataggacttactgtaccttaaaaatcagaaaaaataaaccaaaaaagaggagaccaaatgcaATGTTGGACAAAAA
                    ********** **    *** *  **    *   **  * ******* ***     **  ***** * ** *   ***** *  *****
```

31899.10.peg.2066 (Athe_1569) *pelB*

31899.10.peg.2066	Caldicellulosiruptor bescii strain DSMZ 6725	351627.8.peg.767	Caldicellulosiruptor saccharolyticus DSM 8903
521460.8.peg.1643	Anaerocellum thermophilum DSM 6725	608506.3.peg.1075	Caldicellulosiruptor obsidiansis OB47
1222016.3.peg.2490	Caldicellulosiruptor changbaiensis strain CBS-Z	632335.8.peg.2496	Caldicellulosiruptor kristjanssonii 177R1B
1214564.3.peg.2044	Caldicellulosiruptor sp. F32	632516.3.peg.2071	Caldicellulosiruptor lactoaceticus 6A
632292.3.peg.1248	Caldicellulosiruptor hydrothermalis 108	632348.3.peg.370	Caldicellulosiruptor kronotskyensis 2002

```
31899.10.peg.2066  tcaaaatctaacattttctcaaaattattgagaggattttagtgatttttaggttattttaaatgtAtaaaacttaaagtaataggaggtaaactcGTGAGAATA
521460.8.peg.1643  tcaaaatctaacattttctcaaaattattgagaggattttagtgatttttaggttattttaaatgtataaaacttaaagtaataggaggtaaactcGTGAGAATA
1222016.3.peg.2490 tcaaaatctaacattttctcaaaattattgagaggattttagtgatttttaggttattttaaatgttatgaacctaaagtaataggaggtaaactcTTGAGAATA
608506.3.peg.1075  tcaaaatctaacattttctcaaaattattgagaggattttagtgatttttaggttattttaaatgt-atgaacctaaagtaataggaggtaaactcTTGAGAATA
632292.3.peg.1248  tcaaaatctaacattttctcaaaattattgagaggattttagtgatttttaggttattttaaatgt-atgaacctaaagtaataggaggtaaactcTTGAGAATA
1214564.3.peg.2044 tcaaaatttaacattttctcaaaattattgagaggattttagtgatttttaggttattttaaatgt-atgaacccaaagtaataggaggtacactcTTGAGAATA
351627.8.peg.767   tcaaaatctaacattttctcaaaattattgagaggattttagtgatttttaggttattttaaatgt-atgaacccaaagtaataggaggtacactcTTGAGAATA
632335.8.peg.2496  gcaaaaattgacaacataattaaaattttgagaagaccatattgagaagttgttatatggtgaagat-ataaaaagtttttgctggaggctttagaaATGATAATA
632516.3.peg.2071  gcaaaaattgacaacataattaaaattttgagaagaccatattgagaagttgttatatggtgaagat-ataaaaagtttttgctggaggctttagaaATGATAATA
632348.3.peg.370   gcaaaaacttacaacataattaaaattttgagaagaccaaattaaaaagttgttatatggtaaagat-ataagagctttatggcggagggattagg-ATGATAATA
                    *****  * ***    *     *** * ****** **     * * *     **     ***  * **  *     *         *    *  *     *     *** ****
```

31899.10.peg.2380 (Athe_1867) *celA1-B1-A2-B2-C* new tandem repeat motif for unknown TF (motif 1) (potential activator)
Predicted novel palindromic TF binding site (motif 2) (potential activator)

31899.10.peg.2380	Caldicellulosiruptor bescii strain DSMZ 6725
632348.3.peg.904	Caldicellulosiruptor kronotskyensis 2002
608506.3.peg.1794	Caldicellulosiruptor obsidiansis OB47
1387557.3.peg.2143	Caldicellulosiruptor sp. Wai35.B1
1222016.3.peg.2030	Caldicellulosiruptor changbaiensis strain CBS-Z
1387569.3.peg.1675	Thermoanaerobacter cellulolyticus NA10
351627.8.peg.1211	Caldicellulosiruptor saccharolyticus DSM 8903

```
31899.10.peg.2380  caataaaaa-aaatatcagaaatatgagtatatctaagtaatcgaaaatatttaaactgtactgcattttagacaattaaacaaacaattagacaaaaaaa
632348.3.peg.904   caataaaaa-aaatatcagaaatatgagtatatctaagtaatcaaaaatatttaagctgtactgcattttagacaattaaacaaacaattagacaaaaaaa
608506.3.peg.1794  taataaaaa-gagcataaagaataaaagtgtgtataaggaatgaaaaatagaaatagcgtattgcgtttttgacaattaaataaacaattagacaaaaaaa
1387557.3.peg.2143 caataaaaagaaatatcagaaatataagcgcatctaaggaacagaaagtatataggtgtactgcattttttgacaattaaacaaacaattagacaaaaaaa
1387569.3.peg.1675 taataaaaa-gagtataagaaataaaagtgtgtatatggaatgaaaaatagaaatagcgtattgcgtttttgacaattaaacaaacaattagacaaaaaaa
1222016.3.peg.2030 taataaaaa-gaatatgagaaataaaagtacgtataaggaa-ggaaaatagaaatagcgtagtgtgtttttgacaattaaacaaacaattagacaaaaaaa
351627.8.peg.1211  taataaaaa-gaatatgagaaataaaagtacatataaggaa-ggaaaatagaaatagcgtagtgtgtttttgacaattaaacaaacaattagacaaaaaaa
                    ********  *  ** *  ****  **    * ** * **   *** **   *    *** **  **** ********** *******************
```

```
31899.10.peg.2380  atcaattgtcatctatccattttagttatataattaaaatCgaaa-------taaactgaatga-ggaggttagag-atttATGAAGCG
632348.3.peg.904   atcaattgtcatctatccattttagttatacaattaaaatcgaaa-------taaattgaatga-ggaggttagag-attaATGAAGCG
608506.3.peg.1794  -tcaattggcatgcaagtatttttggtatataat-aaaatcaaaaattatagtaaactgaataa-ggaggttagaa-tttcATGCAGCG
1387557.3.peg.2143 atcaattggcttatacgcattttagttatataattaaaactgaaa-------taaattgaatga-gggggttagag-atttATGAAGCG
1387569.3.peg.1675 atcaattggcatgcacatattttgggtgtataat-aaaatcgaaaattacaataaactgaataaaggaggttagag-gtttATGAAGCG
1222016.3.peg.2030 atcaattggcatgcacgtattttgggtatataattgaaatcgaaaattataataaactgaatga-gggggttagagattttATGCAGCG
351627.8.peg.1211  atcaattggcatgcacgtattttgggtatataattgaaatcgaaaattataataaactgaatga-gggggttagagattttATGCAGCG
                    ******* * *  *   ***** * * ** ***  ***   ***       **** ***** * ** *******   ** *** ****
```

31899.10.peg.2370 (Athe_1860) *celF* Predicted novel palindromic TF binding site (motif 2) (potential activator)

31899.10.peg.2370	Caldicellulosiruptor bescii strain DSMZ 6725	632348.3.peg.915	Caldicellulosiruptor kronotskyensis 2002
608506.3.peg.1783	Caldicellulosiruptor obsidiansis OB47	1387557.3.peg.2134	Caldicellulosiruptor sp. Wai35.B1

```
31899.10.peg.2370  tgcaacaattgttaatataaaaaagagcaatattgtaataattatttgcaaaataatgataGaattaatttgcctta
608506.3.peg.1783  tgcaacaattgttaatataaaaaagagcaatattgtaataattatttgcaaaataatgatagaattaatttgcctta
632348.3.peg.915   tgcaacaattgtcaatacaaaaaa--gcaatattataatatttatttgcaaaatagtgatagaattaatttgtctta
1387557.3.peg.2134 tccaacaattgttagtgcattaag-agcaatattgttataattttttgcaagataattatagattcagcgcatttcg
                   * ********** * *  *  **   ******** * *** ** ******* *** * ***** * *       *

31899.10.peg.2370  aaaatataaaatgatacatggtaggaagcgtgtataaaatttaatacaatacgaaagacagaagtgaggtgatagaaa
608506.3.peg.1783  aaaatataaaatgatacatgataggaagcgtgtataaaatttaatacaatacgaaagacagaagtgaggtgatagaaa
632348.3.peg.915   aaaatataaaatgatatatagtagggagcatgtataaaatttaatacaatacgaaagacaggagtgaggtgatagaaa
1387557.3.peg.2134 aaagtataaaatgttgc-taaaaagaataatgtatcaaatctaaaacaacac-agtgacagatgtggggtgataaaag
                   *** ********* *   *   * * *   ***** **** *** **** ** *  *****  *** ******* **

31899.10.peg.2370  ctaaa-gataaagtataattttcaattacccaactcagactattaaatccataattgaa--aagagggggttaagATGCG
608506.3.peg.1783  ctcaa-gataaagtataattttcaattacctaactcagactattaaatccataactgaa--aagagggggttaagATGCG
632348.3.peg.915   ctaaa-gataaagtataattttcaattacccaactaagactattaaatccataattgaa--aagagggggttaagATGCG
1387557.3.peg.2134 gaaggtaatgttgtacatgtaacatgtaagcttcaaatactttcctatttaaaaaaagagaaagagggggttaaaATGCG
                          **   *** *  *  **  **     *  * *** *   **  * **    *  ************* *****
```

31899.10.peg.2369 (Athe_1856) *pecR* Predicted PecR-binding site (potential activator)

31899.10.peg.2369	Caldicellulosiruptor bescii strain DSMZ 6725	608506.3.peg.1782	Caldicellulosiruptor obsidiansis OB47
632348.3.peg.916	Caldicellulosiruptor kronotskyensis 2002	632335.8.peg.2003	Caldicellulosiruptor kristjanssonii 177R1B
1387557.3.peg.2133	Caldicellulosiruptor sp. Wai35.B1		

```
31899.10.peg.2369  atttttaaagatgcttaaaattttcctgtccaaaataaaattttcttgctttaactgtgacaagttgtgttataaa-ctttatTgtgaa
632348.3.peg.916   atttttaaagatgcttaaaattttcctgtccaaaataaaattttcttgctttaactgtgacaagttgtgttataaa-ctttattgtgaa
1387557.3.peg.2133 atttttaaaggtgtttaaaattttcctgtccaaaataaaattttcttgctttaagtgtgacaagttgcgtgataaa-ctttattgtgaa
608506.3.peg.1782  ag----aaacaagcttaaaattttcctagctgaaataaaactttcttgtttactctgtgacgatttacttgataaagttttattat-aa
632335.8.peg.2003  ag----aaaca-gcttaaaattttcctacctgaaataaaactttcttgtttactttgtcaagatttacttggtaaagttttattat-aa
                   *     ***   * *************  *  ******** ******* **    *** *  * **   *  ****  ****** * **

31899.10.peg.2369  atagctttagcagataaaattaaaat--tcatata-------------aaatc-------caac--cattttt------agtattagg
632348.3.peg.916   atagctttagcagataaaattaaaat--tcatata-------------aaatc-------caac--aattttt------agtattagg
1387557.3.peg.2133 gtggctatagcagataaaattaaaat--tcatata-------------aaatc-------cgac--aattttt------agcatgagg
608506.3.peg.1782  gtggcggaagcaaataaaattgatatgtttatttattacaatatttagaaatcatgtaaacaacacagttttt----agtatatggtg
632335.8.peg.2003  -------aagcaaataaaaattatatgattatttattacgat-ttaaaaaatcatgatgacaacgtagttttttattattatatgatg
                           **** ****** * * **  * ** **             *****       * **    *****         **   *

31899.10.peg.2369  ggttagggggtttatgtaggaaaATGGGAAGAACCAT
632348.3.peg.916   ggttagggggtttatgtaggaaaATGGGAAGAATTAT
1387557.3.peg.2133 ggttagggggtttgtgtagaaagATGAGAAGAATTAT
608506.3.peg.1782  ggttagggggtttacgtggaagaATGAAATGCTTTCT
632335.8.peg.2003  gggtagggggt--acgtggaggaATGAAAAGGTTTTT
                   ** ********    ** *    ***  * *     *
```

31899.10.peg.2368 (Athe_1855) *pecABC* Predicted PecR-binding site (potential activator)

31899.10.peg.2368	Caldicellulosiruptor bescii strain DSMZ 6725	608506.3.peg.1781	Caldicellulosiruptor obsidiansis OB47
632348.3.peg.917	Caldicellulosiruptor kronotskyensis 2002	632335.8.peg.2002	Caldicellulosiruptor kristjanssonii 177R1B

```
31899.10.peg.2368 TAGagaaccatatgtatttaaacataccttaataaaatcctatcacattaaaatttctttattcaaaataaacaaattagtaatataaaa
632348.3.peg.917  TAGagaaccatatgtatttaaacataccttaataaaatcctatcacattaaaatttctttattcaaaataaacaaattagtaatataaaa
608506.3.peg.1781 TAGatgcctaaatatattgaaacctgccttaatataatccaaagagattaaaatttcttgtttaaagtaaacaaaccagttaaataaaa
632335.8.peg.2002 TAGagaccta--tatattaaaacctatcttaatatagtccatgtagattaaaatttcttgtttaatgtaaacaaaccaatttaataaaa
                  ****   * *  * **** **** *  ******* * ***    * *************** ** ** ********  * *   ******

31899.10.peg.2368 ---ttgtttaaccatattataaaactaaagaagtgtcccttgaaaaaaaaa--tcaaattaattattattaataaTaacagtggagtagagg
632348.3.peg.917  ---ttgtttaaccatattataaaactaaagaagtgtcccttgaaaaaaagaattcaaattaattattattaataataacagtggagtagaag
608506.3.peg.1781 -tattatttagc--cgttattaaactaaaaaagtgtcccttgaaataaagaaatcaaattcata--tatttatactcacaagggagtggtgg
632335.8.peg.2002 attcaattttgc--tgttattaaactaaaaaagtgtctcttgaaataagaaaatcaaattcata--tatttatagttgcaagggagtggagg
                        ***  *    **** ******** ******* ******* **  *  ******* **   **** *** *  **  ***** *  *
31899.10.peg.2368 ag--taaaagagaatatttct----actccatactgtggattcaaaagaataagaacacta--agggagggatgaatttgttagcacattttttgtaagc
632348.3.peg.917  ag--taaaagagaatatttat----actccatactgtggattcaaaagaataagaacacca--agggagggatgaatttgttagcacattttttgtaagt
608506.3.peg.1781 gaatcgaacgaaaagatttcttctcactaaaaaata--atatcaaaaataacaatatggca--agggagggataaaattgttgacacattttttataaaa
632335.8.peg.2002 aaatcgaacaaaaagatttcttctcacttaaagatactatatcaaaaatagcaatacggtaaaaggaggggataaaattgttgcaacattttttataaaa
                        **  * ** **** *    ***  *   *      ******  *  *  *    *  ***  ***** ** *****  ********* ***
31899.10.peg.2368 aacagttaaagaactatttt-caacaaattt--ttgatgcaga-ccgtaaataattat-aac--aaaaaagaggaggtttgggtgATGAGTAACAGGAAG
632348.3.peg.917  aacagttaaagaactatttt-caacaaattt--ttgatgcaga-ccgtaaataattat-aac--aaaaaagaggaggtttgggtgATGAGTAACAGGAAG
608506.3.peg.1781 atcaattaaaggactttttttcaacaactttaattggtgcaaagccacaaattatttt-aacaaaaaaagagggaggtttgagtgATGAGTAACAAGAAG
632335.8.peg.2002 atcagttaaaggactttttcccaacaacttta-ttggtgtaaagctacaaattattttttaactaaaaaagagggaggtttgggtgATGAGTAACAAGAAG
                  * ** ****** *** ***  ****** ***  *** ** * * *   **** *** * ***  *****   ********* ************* ****
```

31899.10.peg.2368 (Athe_0096) *nagA*

31899.10.peg.481	Caldicellulosiruptor bescii strain DSMZ 6725
1222016.3.peg.2876	Caldicellulosiruptor changbaiensis strain CBS-Z
608506.3.peg.93	Caldicellulosiruptor obsidiansis OB47
632335.8.peg.2215	Caldicellulosiruptor kristjanssonii 177R1B
632516.3.peg.2333	Caldicellulosiruptor lactoaceticus 6A

```
31899.10.peg.481   ataaatgaaataataagaatat-atcacctataacga--aataat--cattctcagtactaaaaatactt----tgaca-tat
1222016.3.peg.2876 ataaatgaaataataagaatat-atcacctataacga--aataat--cattctcagtactaaaaatactt----tgaca-tat
608506.3.peg.93    ataaatgaaataataagaatat-atcacctataacga--aataat--cattctcagtactaaaaatactt----tgaca-tat
632335.8.peg.2215  ctagaaagaataataatcttgtcatggagaaatacgagcaataataacaatatcaagaacaaaattatttgttttgatagcga
632516.3.peg.2333  ctagaaagaataataatcttgtcatggagaaatacgagcaataataacaatatcaagaacaaaattatttgttttgatagcga
                    ** *   ********   * * **     *  ****  ******  ** * ***  *  **** ** **    *** *

31899.10.peg.481   gatttaaaaaaatcgaattaagtttgtaaaaaagaaaataaaacaatattttga--ggtattttcaatcATGAGAAAATACAT
1222016.3.peg.2876 gatttaaaaaa-tcgaattaagtttgtaaaaaagaaaacaaaacaatattttga--ggtgttttcaatcATGAGAAAATACAT
608506.3.peg.93    gatttaaaaaaatcgaattaagtttgtaaaaaagaaaacaaaacaatattttga--ggtattttcaatcATGAGAAAATACAT
632335.8.peg.2215  aatttaaagaaaacgtgagatattt----tgagaaaatcaaaggtatattgagaaaggtggttttaaaaATGAGAAAAAAGTT
632516.3.peg.2333  aatttaaagaaaacgtgagatattt----tgagaaaatcaaaggtatattgagaaaggtggttttaaaaATGAGAAAAAAGTT
                    ******* **  **       ***      *  ***  ***   *****  **  ***  ***  ** ********* *  *
```

31899.10.peg.1153 (Athe_0699) srlD<>*sorE*

31899.10.peg.1153	Caldicellulosiruptor bescii strain DSMZ 6725	1387557.3.peg.967	Caldicellulosiruptor sp. Wai35.B1
632292.3.peg.2061	Caldicellulosiruptor hydrothermalis 108	1121259.3.peg.911	Caldicellulosiruptor acetigenus DSM 7040
632348.3.peg.2034	Caldicellulosiruptor kronotskyensis 2002	351627.8.peg.987	Caldicellulosiruptor saccharolyticus DSM 8903
632518.3.peg.617	Caldicellulosiruptor owensensis OL	1222016.3.peg.2255	Caldicellulosiruptor changbaiensis strain CBS-Z
608506.3.peg.718	Caldicellulosiruptor obsidiansis OB47		

```
31899.10.peg.1153  acccccttttgatattgttttaacatgttgtctttttgctctatctaaaattaattatacaatatgt
632518.3.peg.617   ccccctttttttgatattgttttaacatgttgtctttttgctttatctacaattaattatacaatatat
608506.3.peg.718   -aacccccttttgatattgttttaacatgttgtctttttgctttgtctacaattaattatacaatatat
632348.3.peg.2034  ctacccccttttgatattgttttaacatgttgtctttttgctctatctaaaattaattatacaatatgt
1387557.3.peg.967  aaacccccttttgatattgttttaacatgttgtctttttgctctatctaaaatcaattatacaatatgt
1121259.3.peg.911  aaacccccttttgatattgctttaacatgttgtctttttgttctatctaaaatcaattatacaatatgt
632292.3.peg.2061  gtacccccttttgatattgttttaacatgttgtctttttgctctatctaaaataaattatacaatacat
351627.8.peg.987   ttacctccttaaacattcttttatatgttgatttttgcacttatctaatactaattatacaacaaat
1222016.3.peg.2255 gtacctcctttgacattgttttaatatgttgatttttaggcttatctaatattaattatacaataaat
                      **   **  * ***  *** * ******  ****     * ****  *  ********** * *

31899.10.peg.1153  gttgtgtttatgtctatatcaatttgatttatttttgagtttattgattttttgactaagatgtattatca
632518.3.peg.617   gttgtgtttatgtctatatcaatttgatttatttttgactttattgattttttggctaagatatattatta
608506.3.peg.718   gttgtgtttatgtctatatcaatttgatttatttttgactttattgattttttggccaagatgtattatca
632348.3.peg.2034  gttgtgtttatgtctatatcaatttgatttatttttgagtttattgatttttggactaagatgtattatca
1387557.3.peg.967  gttgtgtttatgtctatatcaatttgatttgtttttgactttattgattttttggctaagatgtattatca
1121259.3.peg.911  gttgtgtttatgtctatatcaatttgatttatttttgactttattgattttttggctaagatgtattatca
632292.3.peg.2061  gttgtgtttgtgtctatatcaatttgatttatttttgactttattgattttttagccaagatgtattatca
351627.8.peg.987   atttgtttatgtctatattaatttgttttatttttgaatttattgattttttcgatcaaatgtattatca
1222016.3.peg.2255 atttgtttatgtctatattaatttgttttatttttgactttattgattttttcaatcaaatgtattatta
                    ** ***** ********* ****** *** ******* *************     * ** ****** *

31899.10.peg.1153  ttatcttAtgataacattgatgtt-atagttaagatttt-gatttgtaaaggaggatataaa-aATGAA
632518.3.peg.617   ttatcttataataaccctgatgtt-atagataagatttt-gatttataaaggaggatataaa-aATGAA
608506.3.peg.718   ttatcttataatagcattgatatttatagataagatttt-gatttataaaggaggatataaa-aATGAA
632348.3.peg.2034  ttatcttatgacaacattgatgtt-atagttaagatttt-gatttgtaaaggaggatataaa-aATGAA
1387557.3.peg.967  ttatcttataaaaaccctgatgtt-ataaataatatttt-gatttatgaaggaggatataaa-aATGAA
1121259.3.peg.911  ttatcttgtaacaaccctgatgtt-atagataagatttt-gatttgtaaaggaggatataaa-aATGAA
632292.3.peg.2061  ttatcttataacaaccctgatgtt-ataggtaagatttt-gatttgtaaaggaggatataaa-aATGAA
351627.8.peg.987   ttattttataa-gccattaaagta--tgggtaaaatttttattttagaaggaggacctaaacaATGAA
1222016.3.peg.2255 ttgtcttataa-gctattattgtt--tgagtgtaatttttgattttaaagggagggtataaaaaATGAA
                   ** * ** * *      *    *   *   *   *****  ****   * *****  **** ******
```

31899.10.peg.2294 (Athe_1783) deoZ-mtaP-deoUVWR-cda-deoACBD Predicted DeoR-binding site (repressor)

31899.10.peg.2294	Caldicellulosiruptor bescii strain DSMZ 6725	1387557.3.peg.2062	Caldicellulosiruptor sp. Wai35.B1
632348.3.peg.989	Caldicellulosiruptor kronotskyensis 2002	1222016.3.peg.1957	Caldicellulosiruptor changbaiensis strain CBS-Z
632292.3.peg.1037	Caldicellulosiruptor hydrothermalis 108	1387569.3.peg.1588	Thermoanaerobacter cellulolyticus NA10
1121259.3.peg.647	Caldicellulosiruptor acetigenus DSM 7040	351627.8.peg.2511	Caldicellulosiruptor saccharolyticus DSM 8903
632335.8.peg.993	Caldicellulosiruptor kristjanssonii 177R1B	632518.3.peg.1621	Caldicellulosiruptor owensensis OL

```
31899.10.peg.2294  aggcagccttttataattataattcacaaatttataaattttcatattgacttttttgaataaagatcataaaatagtaatGtcagatgtcagatgt
1222016.3.peg.1957 aagcagccttttata-ttatagttcacaaatttacaaattttcatattgacttttttgaacaaagattataaaatagtaatgtcagatgtcagatgt
351627.8.peg.2511  aagcagctttttata-ttatagttcacaaatttacaaattttcatattgacttttttgaacaaagattataaaatagtaatgtcagatgtcagatgt
1387569.3.peg.1588 aagcagccttttata-ttatagttcacaaatttacaaattttcatattgacttttttgaacaaaaattataaaataatagtgtcagatgtcagatgt
632348.3.peg.989   aggcagccttttataattataattcacaaatttataaattttcatattgacttttttgaataaagatcataaaatagtaatgtcagatgtcagatgt
1121259.3.peg.647  aggcagccttttataattataattcacaaatttataaattttcatattgacttttttgaacaaaggttataaaataataacgtcagatgtcagatgt
632335.8.peg.993   aggcagccttttataattataattcacaaatttataaattttcatattgacttttttgaacaaaggttataaaatagtaatgtcagatgtcagatgt
632292.3.peg.1037  aggcagccttttataattataattcacaaatttataaattttcatattgacttttttgaacaaaggttataaaataataacgtcagatgtcagatgt
1387557.3.peg.2062 aggcagtcttttataattataattcacaaatttataaattttcatattgacttttttgaacaaaaattataaaatagtaatgtcagatgtcagatgt
632518.3.peg.1621  aggcagccttttataattataattcacaaatttataaattttcatattgacttttttgaacaaagattataaaatagtaatgtcagatgtcagatat
608506.3.peg.1712  aggcagccttttataattataattcacaattttataaattttcatattgacttttttgaacaaagattataaaatagtaatgtcagatgtcagatat
                   * ****  ******* ***** ******* **** ************************* ***  * ******** **  ************** *

31899.10.peg.2294  cagatgttagatgtctgacatctaaaaaatacaaaaggaggtttgctttttATGAAGAG
1222016.3.peg.1957 -------tagatgtctgacatctaaaaaataaaaagg-aggtttgctttttATGAAGAA
351627.8.peg.2511  -------tagatgtctgacatctaaaaaataaaaagggaggtttgctttttATGAAGAA
1387569.3.peg.1588 -------tagatgtctgacatctaaaaaataaaaaatgaggtttgcattttATGAAGAA
632348.3.peg.989   -------tagatgtctgacatctaaaaaatacaaaaggaggtttgctttttATGAAGAG
1121259.3.peg.647  -------tagatgtctgacatctaaaaaataaaaaaggaggtttgctttttATGAAGAA
632335.8.peg.993   -------tagatgtctgacatctaaaaaataaaaaaggaggtttgctttttATGAAGAA
632292.3.peg.1037  -------tagatgtctgacatctaaaaaataaaaaaggaggtctggtttttATGAAGAA
1387557.3.peg.2062 --------------ctgacatctaaaaaataaaagaggaggtttgctttttATGAAGAA
632518.3.peg.1621  -------tagatgtctgacatctaaaaattaaaaaag-aggtttg-tttttATGAAGAA
608506.3.peg.1712  -------tagatgtctgacatctaaaaattaaaaagg-aggtttg-tttttATGAAGAA
                                 ************** ** **    **** **  ***********
```

31899.10.peg.127 (Athe_2580) rhaD2-ramA

31899.10.peg.127	Caldicellulosiruptor bescii strain DSMZ 6725		
632292.3.peg.291	Caldicellulosiruptor hydrothermalis 108	632518.3.peg.2313	Caldicellulosiruptor owensensis OL
632335.8.peg.147	Caldicellulosiruptor kristjanssonii 177R1B	351627.8.peg.494	Caldicellulosiruptor saccharolyticus DSM 8903
1121259.3.peg.1295	Caldicellulosiruptor acetigenus DSM 7040	1222016.3.peg.446	Caldicellulosiruptor changbaiensis strain CBS-Z
632348.3.peg.300	Caldicellulosiruptor kronotskyensis 2002	608506.3.peg.2362	Caldicellulosiruptor obsidiansis OB47
632516.3.peg.553	Caldicellulosiruptor lactoaceticus 6A	1387557.3.peg.19	Caldicellulosiruptor sp. Wai35.B1

```
31899.10.peg.127   gttttcaaaatgtcaaatttgggtattttcaaatcaaccaaataattgtaaaataaaata
632335.8.peg.147   catttaaagaggtcaaatttgggtattttcaaatcaatcaaataattgtaaaatgaaata
1121259.3.peg.1295 catttaaagaggtcaaatttgggtattttcaaatcaatcaaataattgtaaaatgaaata
632516.3.peg.553   catttaaagaggtcaaatttgggtattttcaaatcaatcaaataattgtaaaatgaaata
632292.3.peg.291   cttttcaagaggtcaaatttgggtattttcaaatcaatcaaataattgtaaaatgaaata
632518.3.peg.2313  gtttttaagaggtcaaatttgggtattttcaaatcaatcaaataattgtagaataaaata
632348.3.peg.300   gttttcaaaatgtcaaatttgggtattttcaaatcaaccaaataattgtaaaataaaata
351627.8.peg.494   gtttttaaaatgtcaaatttggatattttcaaatcaatcaaataattgtaaaataaaata
1387557.3.peg.19   gttttcaaaatgtcaaatttgggtattttcaaatcaatcaaataattgtaaaataaaata
1222016.3.peg.446  cttttcaaaaggtcaaatttgggtattttcaaatcaatcaaataattgtaaaatgaaata
608506.3.peg.2362  cttttcaaaaggtcaaatttgggtattttcaaatcaatcaaataattgtaaaataaaata
                     *** ** * *********** ************** ************ *** *****

31899.10.peg.127   Gaacaaaaaa-tcaaaacaatacaatattacgacaatacactttaaaagaaaggtggaaaaa--ATGCAGGATAT
632335.8.peg.147   gaacaaaaaaatcaaaacaacacaatatcatgacaatacattttaaa-gaaaggtggaaaaaa-ATGCAGAATAT
1121259.3.peg.1295 gaacaaaaaaatcaaaacaacacaatatcatgacaatacattttaaa-gaaaggtggaaaaaa-ATGCAGAATAT
632516.3.peg.553   gaacaaaaaaatcaaaacaacacaatatcatgacaatacattttaaa-gaaaggtggaaaaa--ATGCAGAATAT
632292.3.peg.291   gaacaaaaaa-tcaaaacaacacaatatcatgacaatacattttaaa-gaaaggtggaaaaaaaATGCAGGATAT
632518.3.peg.2313  gaacaaaaaaatcaaaacaaaacaatatcatgacaatgcattttaaa-gaaaggtggaaaaaa-ATGCAGGATAT
632348.3.peg.300   gaacaaaaaa-tcaaaacaatacaatattacgacaatacactttaaaagaaaggtggaaaaa--ATGCAGGATAT
351627.8.peg.494   taacaaaaaa-tcaaaacaccacaatattacgataatacattttaaaagaaaggtggaaaaaa-ATGCAGGATAT
1387557.3.peg.19   taacaaaaaa-tcaaaacaacacaatatcatgacaatatactttaaa-gaaaggtggaaaaaa-ATGCAGGATAT
1222016.3.peg.446  gaacaaaaaa-tcaaaacaacacaatatcatgacaatacattttaaa-gaaaggtggaaaaaa-ATGCAGGATAT
608506.3.peg.2362  gaacaaaaaa-tcaacacaacacaatatggtgacaatacattttaaa-gaaaggtggagaaa--ATGCAAGATAT
                    ********* **** ***  *******   ** ****   ****** ********** **   *****  ****
```

31899.10.peg.2644 (Athe_2094) *ramB*

632518.3.peg.1894	Caldicellulosiruptor owensensis OL
31899.10.peg.2644	Caldicellulosiruptor bescii strain DSMZ 6725
632348.3.peg.571	Caldicellulosiruptor kronotskyensis 2002
1222016.3.peg.826	Caldicellulosiruptor changbaiensis strain CBS-Z
1387557.3.peg.2507	Caldicellulosiruptor sp. Wai35.B1
608506.3.peg.2003	Caldicellulosiruptor obsidiansis OB47

```
31899.10.peg.2644  cctccttcta-tagtgataaaat-agaaggaggttttatttttgtgtggtttt-----atttaatatttattgatatttgaaatgtttt
632348.3.peg.571   cctccttcta-tagtgataaaattagaaggaggttttatttttgtgtggtttt-----atttaatatttattgatatttgaaatgtttt
632518.3.peg.1894  cctccttctaacagtgataaagt-agaaggaggttttatttttgtgtggtttt-----atttaatatttattgatatttgaaatgtttt
1222016.3.peg.826  cctccttcta-tggtaacaaaat-agaaggaggttttattttt-tgtggtttt-----atttaatatttattgatttttggaatgtttt
1387557.3.peg.2507 cctccttcta-tagaggcaaagt-agaaggaggttttattttttttgtggtttt-----atttaatatttattgatatttgaaattttttt
608506.3.peg.2003  cccccttctactttt--tactgt-agaagggggttttatttttgtgtattattttgttgtttaatatttgttgatatttataatgattt
                   ** *******        *   * ****** ************ ***  * **      ********** ***** ***  ***  ***

31899.10.peg.2644  tgtataaaattttattaaataaagccaaaatcaatctaataaa-tgtgaaaggggaaacatacaATGAA
632348.3.peg.571   tgtgtaaaattttattaaataaagccaaaatcaatctaataaa-tgtgaaaggggaagcatacaATGAA
632518.3.peg.1894  tgtgtaaaattttattaaataaagctaaaatcaatctaacaaa-tgtgaaaggggaaatatccaATGAA
1222016.3.peg.826  tgtgtaaaattttattaaataaggcaaaaatcaatctaataaaatgtgaaaggagaaacatacaATGAA
1387557.3.peg.2507 tgtgtaaaattttattaaacaaagcaaaaatcaatctaataaa-tgtgaaaggagaaacatacaATGAA
608506.3.peg.2003  tgtgtagaatctttattaaatagaaataataaaaatcacataaa-aatgaaaggagaaataaaaaTTGAA
                   *** ** *** ********* *     ** *  ****  * ***   ******* ***  *   * ****
```

31899.10.peg.2643 (Athe_2093) *rglFGH-rglX*

31899.10.peg.2643	Caldicellulosiruptor bescii strain DSMZ 6725	1222016.3.peg.827	Caldicellulosiruptor changbaiensis strain CBS-Z
632518.3.peg.1892	Caldicellulosiruptor owensensis OL	608506.3.peg.2002	Caldicellulosiruptor obsidiansis OB47
632348.3.peg.572	Caldicellulosiruptor kronotskyensis 2002		

```
31899.10.peg.2643  gcc--tttggttttgtaagtctttccttgtagcaatatttagctcactttcaaagtaaaaaaatctttgtagctgttc
1222016.3.peg.827  gccc-tttggttttgtaagtatttctttttagcaatatttagctcattttaaaaataaaaaactctttttagttattc
608506.3.peg.2002  gctaattttgtttttaaatcttttctttgagcaatatttaactcactttaaaattaaaaag-tccttatagctattc
632348.3.peg.572   gcc--tttggttttgtaagtctttccttgtagcaatatttagctcactttaaaagttaaaaaatctttgtagctgttc
632518.3.peg.1892  gcc--tttggttttgtaagtctttccttgtagcaatatttagctcactttaaaaataaaaaaatctttgtagctgttc
                   **   *** ***** *** * ***  **  *********** **** *** *** * ****  ** ** *** * ***
31899.10.peg.2643 aaaaataaaataaaaaaaataggtattgacacaacaaaaacaaagtattaaaataaaattAaaccaaaaacaaaattcaaatctca
1222016.3.peg.827 aaaaagaaaataaaaaaaataggtattgacacaacaaaaacaaagtattaaaataaaattaaaccaaaaacaaaattcaaatctca
608506.3.peg.2002 aaaaataaaataaaaaaaataggtattgacacaacaaaaacaaagtattaaaataaaattaaaccaaaaacaaaattcaaatctca
632348.3.peg.572  aaaaataaaataaaaaaaataggtattgacacaacaaaaacaaagtattaaaataaaattaaaccaaaaacaaaattcaaatctcc
632518.3.peg.1892 aaaaataaaataaaaaaaataggtattgacacaacaaaaacaaagtattaaaataaaattaaaccaaaaacaaaattcaaatctca
                  ***** *******************************************************************************

31899.10.peg.2643 atattaataccgaacaaaaataatggaaatctgatgaaaacaatattatcaagggaccaaaaaa-gaagggagagaatgatttaATGACA
1222016.3.peg.827 atattaataccgaacaaaaataatggaaatctgatgaaaacaatattctcaagggatcaaaaaa-gaagggagagaatgatttaATGACA
608506.3.peg.2002 atattaataccgaataaaaataatggaaatctgatgaaaacaatattatcaagggaccaaaaaaagaagggagagaatgttttaATGACA
632348.3.peg.572  atattaataccgaataaaaataatggaaatctgatgaaaacaatattatcaatggaccaaaaaa-gaagggagagaatgatttaATGACA
632518.3.peg.1892 atattaatacctaacaaaaataatggaaatctgatgaaaacaatattatcaagggaccaaaaaa-gaaaggagagaatgttttaATGACA
                  *********** ** ************************************* *** ******* ************** **********
```

31899.10.peg.469 (Athe_0085-86-87) Putative TFBS of 2-comp. system Csac_2550-2549 (absent in C.bescii)

31899.10.peg.469	Caldicellulosiruptor bescii strain DSMZ 6725	1387557.3.peg.334	Caldicellulosiruptor sp. Wai35.B1
632348.3.peg.71	Caldicellulosiruptor kronotskyensis 2002	1214564.3.peg.1337	Caldicellulosiruptor sp. F32
1121259.3.peg.2213	Caldicellulosiruptor acetigenus DSM 7040	1222016.3.peg.2897	Caldicellulosiruptor changbaiensis strain CBS-Z
608506.3.peg.74	Caldicellulosiruptor obsidiansis OB47	1387555.3.peg.423	Caldicellulosiruptor sp. Rt8.B8
632292.3.peg.60	Caldicellulosiruptor hydrothermalis 108	351627.8.peg.2753	Caldicellulosiruptor saccharolyticus DSM 8903

```
1899.10.peg.469   taaagtctctgcctttttgtttttgctcaaaaatcttaaatttagataccacttcttaaaaaacccctcttttttatagactttgccc
214564.3.peg.1337 tatagtcccctgcttatt-tttttgcg-aaaaaccttaaatttagataccacttctaaaaaaagccatcttttttatagactttgccc
222016.3.peg.2897 tatagtcccctgcttatt-tttttgcg-aaaaaccttaaatttagataccacttcttaaaaaagccatcttttttatagactttgccc
32348.3.peg.71    taaagtctctgcctttttatttttgatcaaaaatcttaaatttagataccacttcttaaaaaacccctcttttttatagactttgccc
121259.3.peg.2213 tagagcctctgcctttttgttttt-cttaaaaatcttaaatttagataccacttcttaaaaaagccctcttttttatagactttgccc
08506.3.peg.74    tagagcctctgcctttttgttttttctcaaaaatcttaaatttagataccacttcttaaaaaagccctcttttttatagactttgccc
32292.3.peg.60    t-gagcctctgcctttttgtttttgctcaaaaatcttaaatttagataccacttcttaaaaaagccctcttttttatagactttgccc
387557.3.peg.334  ttaagcctctgcctttttatttttgctcaaaaaccttaaatttagataccacttctaaaaaaagccatcttttttatagactttgccc
387555.3.peg.423  aataatcccctgcttatttttttt--caaaacaccttaaatttagataccacttcttaaaaaacccatcttttttatagactttgccc
51627.8.peg.2753  tgttttttattacatataatttaagatttgagaccttaaatttagataccacttctaaaaaaacaggtcttttttatatgacttatcc
                              * * *  ***        * * ********************** ******    ***********    **  **

1899.10.peg.469   cattttataataaaatCagaaaaacaaattgtagcttatgtaatataaaaaatataacctc-tatagaaggaaggcgatgtatcATGGAA
214564.3.peg.1337 cattttataataaaatcagaaaaacaaattgtagcttatgtaatataaaaagtataacttc-tacagaaggaaggcgatatatcATGGAA
222016.3.peg.2897 cattttataataaaatcagaaagataaattgtagcttatgtaatataaaaagtataacctc-tacagaaggaaggcgatatctcATGGAA
32348.3.peg.71    cattttataataaaatcagaaaaacaaattgtagtttatgtaatataaaaaatataacctt-tatagaaggaaggcgatatatcATGGAA
121259.3.peg.2213 cattttataataaaatcagaaaaataaattgtagcttatgtaatataaaaaatataacctc-tacagaaggaaggcgatgtattATGGAA
08506.3.peg.74    cattttataataaaatcagaaaaataaattgtagcttatgtaatataaaaaatataacctc-tatagaaggaaggcgatgtatcATGGAA
32292.3.peg.60    cattttataataaaatcagaaaaataaattgtagcttatgtaatataaaaaatattacctt-tgcagaaggaaggcgatatgtcATGGAA
387557.3.peg.334  cattttataataaaatcagaaaaataaattgtagcttatgtaatattaaaaatataacctc-tacagaaggaaggcgatatctcATGGAA
387555.3.peg.423  cattttataataaaaacagaaaaagaaattgtagcttatgtaatat-aaaaatataacctgacaaagaaggaaggcgattgtttATGGAA
51627.8.peg.2753  cactttataataaaatcagaaaaacaaattagtaattttgcaatacatattttataaaact-----aaaggaaggcggtaagtaATGGAT
                  ** ************ ****** * *****     ** ** ****   *   *** *          **********      *   * *
```

31899.10.peg.2806 (Athe_2242) *hypBA1*

31899.10.peg.469	Caldicellulosiruptor bescii strain DSMZ 6725	1387557.3.peg.334	Caldicellulosiruptor sp. Wai35.B1
632348.3.peg.71	Caldicellulosiruptor kronotskyensis 2002	1214564.3.peg.1337	Caldicellulosiruptor sp. F32
1121259.3.peg.2213	Caldicellulosiruptor acetigenus DSM 7040	1222016.3.peg.2897	Caldicellulosiruptor changbaiensis strain CBS-Z
608506.3.peg.74	Caldicellulosiruptor obsidiansis OB47	1387555.3.peg.423	Caldicellulosiruptor sp. Rt8.B8
632292.3.peg.60	Caldicellulosiruptor hydrothermalis 108	351627.8.peg.2753	Caldicellulosiruptor saccharolyticus DSM 8903

```
31899.10.peg.2806  attattcatcttgaaaagtgaataaaaatattagaaaataaaattAgtttagaaaaacaaaaaatattttaggtaggtgaaaaa--gATGAGTGATAAA
521460.8.peg.2361  attattcatcttgaaaagtgaataaaaatattagaaaataaaattagtttagaaaaacaaaaaatattttaggtaggtgaaaaa--gATGAGTGATAAA
632348.3.peg.432   atttttcatcttgaaaagtgaataaaaatattagaaaataaaattagtttagaaaaacaaaaaatattttaggtaggtgaaaaa--gATGAGTGATAAA
632292.3.peg.580   atttttcatcttgaaaaatgaataaaaatattagaaaataaaattagtttagaaaaataaaaaatattttaggcaggtgaaaaa--tATGAAAGATAAA
1121259.3.peg.1238 atttttcatcttgaaaaatgaataaaaatattagaaaataaaattagtttataaaaacaaaaattattttaggttggtgaaaaa--tATGAATGATAAA
632518.3.peg.2046  acttaagacattgaaaattggattaaaatattagaaaat-----tagttt-taaaaataaaaaatattttaggcaggtgaaaaaaagATGAATGATAAA
1214564.3.peg.368  ttttttcaacttgaaaaagtaagaataatgtcggaaaataaagacgaaggataaaaattaaatac---ttaggt-ggtgaaaaaa-tATGACTGAAAAT
351627.8.peg.823   ttttt-caacttgaaaaagtaagaataatgtcagaaaataaagacaaagaataaaa-ttagatac---ttaggt-ggtgaaaaaa-tATGACTGAAAAT
1387557.3.peg.2643 ttttt-caacttgaaaaagtaagagtaatgtcagaaaataaagataaagaataaaa-ttaaatga---ttaggt-ggtgaaaaaa-tATGACTGAAAAT
1222016.3.peg.2417 ttttt-caacttgaaaaagtaagagtaatgtcagaaaataaagataaagaataaaa-ttaaatga---ttaggt-ggtggaaaaa-tATGACTGAAAAT
                     *    *  *******    *    *** *  *****              ****   * *      *****  **** ****   ****  ** **
```

31899.10.peg.1130 (Athe_0679) *manP2*

31899.10.peg.1130	Caldicellulosiruptor bescii strain DSMZ 6725
632348.3.peg.2055	Caldicellulosiruptor kronotskyensis 2002
632335.8.peg.1641	Caldicellulosiruptor kristjanssonii 177R1B
632292.3.peg.2081	Caldicellulosiruptor hydrothermalis 108
632516.3.peg.2164	Caldicellulosiruptor lactoaceticus 6A
608506.3.peg.693	Caldicellulosiruptor obsidiansis OB47
1121259.3.peg.890	Caldicellulosiruptor acetigenus DSM 7040
632518.3.peg.598	Caldicellulosiruptor owensensis OL
351627.8.peg.966	Caldicellulosiruptor saccharolyticus DSM 8903
1214564.3.peg.1100	Caldicellulosiruptor sp. F32
1222016.3.peg.2274	Caldicellulosiruptor changbaiensis strain CBS-Z
1387555.3.peg.2334	Caldicellulosiruptor sp. Rt8.B8
1387557.3.peg.946	Caldicellulosiruptor sp. Wai35.B1
1387569.3.peg.791	Thermoanaerobacter cellulolyticus NA10

```
31899.10.peg.1130  tattgacaggtttc-aaagatattttacataatttttttgtaattattcaataccaaatgtggtatatttcaatgtatagaca
632292.3.peg.2081  tcttgagagattttaaaaatattta-ataatttttttgtaattattcaacagcaaatgtggtatatttcaatgtatagaca
632516.3.peg.2164  tcttgacagattttaaaaatattta-ataatttttttgtaattattcaacagcaaatgtggtatatttcaatgtatagaca
632335.8.peg.1641  tcttgacagattttaaaaatattta-ataatttttttgtaattattcaacagcaaatgtggtatatttcaatgtatagata
1121259.3.peg.890  tcttgacagattttaaaaatattta-ataatttttttgtaattattcaacagcaaatgtggtatatttcaatgtatagata
351627.8.peg.966   ccttgacagtttttaaaaatattta-ataatttttttgtaatgattcaacagaaaatgtggtatatttcattgtatagata
1214564.3.peg.1100 ccttgacagtttttaaaaatattta-ataatttttttgtaatgattcaacagaaaatgtggtatatttcattgtatagata
1222016.3.peg.2274 gcttgacagtttttaaaaatattta-ataatttttttgtaatgattcaacagaaaatgtggtatatttcattgtatagata
1387555.3.peg.2334 ccttgacagtttttaaaaatattta-ataattttctgtaatgattcaacagaaaatgtggtatatttcaatgtatagaga
1387557.3.peg.946  ccttgacagtttttaaaaatattta-ataatttttttgtaatgattcaacagaaaatgtggtatatttcattgtatagata
1387569.3.peg.791  tcttgacagttttaaaaaatattta-ataatttttttgtaatgattcaacagaaaatgtggtatatttcaatgtatagacg
632348.3.peg.2055  tattgacaggtttc-aaagatattttacataatttttttgtaattattcaataccaaatgtggtatatttcaatgtatagaca
608506.3.peg.693   ccttgacagatttccaaagatattttacataatttttttgtaattattcaatatcaaatgtggtatatttcaatgtataaata
632518.3.peg.598   ccttgacagatttccaaagatattttacataatttttttgtaattattcaatatcaaatgtggtatatttcaatgtatagata
                     **** ** ***  *** ******** ********* ****** ****** *  **************** ****** *

31899.10.peg.1130  attt-atttgtgggggattaaaacaATGAAAAAGGTCTTTGCAAAAAAGGACATATTTACACGCTA
632292.3.peg.2081  attt-atttgtgggggattaaaacaATGAAAAAGGTCTTTGCAAAAAAGGACATATTTACCCGCTA
632516.3.peg.2164  attt-atttgtgggggattaaaataATGAAAAAGGTCTTTGCAAAAAAGGATATATTCACACGCTA
632335.8.peg.1641  attt-atttgtgggggattttattA-TGAAAAAGGTCTTTGCAAAAAAGGATATATTCACACGCTA
1121259.3.peg.890  attt-atttgtgggggattttattA-TGAAAAAGGTCTTTGCAAAAAAGGATATATTCACACGCTA
351627.8.peg.966   aatc--attgtgggggattttac--ATGAAGAAGGTCTTTGCAAAAAAGGATATATTTACGCGCTA
1214564.3.peg.1100 aatc--attgtgggggattttac--ATGAAGAAGGTCTTTGCAAAAAAGGATATATTTACGCGCTA
1222016.3.peg.2274 aatc--attgtgggggattttac--ATGAAGAAGGTCTTTGCAAAAAAGGATATATTCACGCGCTA
1387555.3.peg.2334 aata-gattgtgggggatttgac--ATGAAGAAAGTCTTTGCAAAAAAGGATATATTTACACGTTA
1387557.3.peg.946  aatc--atttgggggattttac--ATGAAGAAGGTCTTTGCAAAAAAGGATATATTTACGCGCTA
1387569.3.peg.791  aata-aattgtgggggattttac--ATGAAGAAGGTCTTTGCAAAAAAAGATATATTCACTCGCTA
632348.3.peg.2055  attt-atttgtgggggattaaaacaATGAAAAAGGTCTTTGCAAAAAAGGATATATTCACGCGCTA
608506.3.peg.693   attttatttgtgggggattttaatA-TGAAAAAGGTCTTTGCAAAAAAGGACATATTTACGCGCTA
632518.3.peg.598   attt-atttgtgaggggattttaatA-TGAAAAAGGTCTTTGCAAAAAAGGATATATTCACACGCTA
                   * *    ** ** *******  *    **** ** ************** ** ***** ** ** **
```

31899.10.peg.2625 (Athe_2076) *Athe_2076-78-79*

31899.10.peg.2625 Caldicellulosiruptor bescii strain DSMZ 6725
1222016.3.peg.843 Caldicellulosiruptor changbaiensis strain CBS-Z
608506.3.peg.1987 Caldicellulosiruptor obsidiansis OB47
632348.3.peg.594 Caldicellulosiruptor kronotskyensis 2002
632518.3.peg.1882 Caldicellulosiruptor owensensis OL
1387557.3.peg.2482 Caldicellulosiruptor sp. Wai35.B1

```
31899.10.peg.2625  taggttttgatttgtttttattgattctttttattttgttgtgtataattaaaa---taaataact-----tt
1222016.3.peg.843  taggttttgatttgtttttgttgattctttttattttgttgtgtataattaaaa---taaataact-----tt
1387557.3.peg.2482 tagattttgatttgtttttgttgattctttttattttgtttagtataattaaag---taaataatt-----ct
608506.3.peg.1987  ttgttttgtttaattctatgttgattgtatttaacttgtggagtataattaagaatataattaatcaaaaaac
632518.3.peg.1882  ttgttttgtttaattctatgttgattgtatttaacttgtggagtataattaagaatataatcaatcaaaaaac
632348.3.peg.594   ttgattctttcaattttgtgttgattgtatttgagttatatagtataattagggatgtaaatataaaaaaacc
                   * * **        * * * ****** * ***   ** *   *********      ***  *

31899.10.peg.2625 aaagtaaatacaa-------c-taaaacaattt----------ggagtgaaaaaaA-----TGAACATCAACTTTAA
1222016.3.peg.843 aaaataaatacaa-------c-taaaacaattt----------ggagtgaaagaaA-----TGGACATCAATTTCAA
1387557.3.peg.2482aaaataaatacca-------cataaaataattt----------ggagtgaaatgaA-----TGACCATTAATTTTAA
608506.3.peg.1987 aaaataaataaaa-------tttaatgcaaatttctctacttgggagtgaaagaaA-----TGAATATTAACTTCAA
632518.3.peg.1882 aaaataaataaaa-------tttaatgcaaatttctctacttaggagtgaaagaaA-----TGAATATTAACTTCAA
632348.3.peg.594  aaaacaaatttgaacagttttttattactattagctaaatataggagtgaggaaaacagaATGGACATTAATTTCAA
                  ***  ****   *         **     * *           *******    *      **   ** ** ** **
```

Genes from the reconstructed CggR and Rex regulons (Central Carbohydrate Metabolism)

31899.10.peg.1901 (Athe_1407) cggR-gap-(pgk-tpi)-pgm-eno **Predicted CggR-binding site (inverted repeat) (repressor)**

31899.10.peg.1901	Caldicellulosiruptor bescii strain DSMZ 6725
608506.3.peg.1243	Caldicellulosiruptor obsidiansis OB47
632518.3.peg.1271	Caldicellulosiruptor owensensis OL
632292.3.peg.1411	Caldicellulosiruptor hydrothermalis 108
632348.3.peg.1378	Caldicellulosiruptor kronotskyensis 2002
632335.8.peg.1516	Caldicellulosiruptor kristjanssonii 177R1B
632516.3.peg.126	Caldicellulosiruptor lactoaceticus 6A
1121259.3.peg.236	Caldicellulosiruptor acetigenus DSM 7040
1387557.3.peg.1656	Caldicellulosiruptor sp. Wai35.B1
351627.8.peg.2134	Caldicellulosiruptor saccharolyticus DSM 8903

```
31899.10.peg.1901  atgccttcctctgctttttttgtatgtatacacttgaatggagataaaaaaatggtataat-at
608506.3.peg.1243  atgtctccttctgctttttttgtatgtatacacttgaatggaaatagaaaaatggtataat-at
632518.3.peg.1271  atgtctccttctgctttttttgtatgtatacacttgaatggaaatagaaaaatggtataat-at
632292.3.peg.1411  atgtcttcctctgctttttttgtatgtatacacttgaatggaaataaaaaaatggtataat-at
632348.3.peg.1378  -tgccttcctctgctttttttgtatgtatacacttgaatggagataaaaaaatggtataat-at
632335.8.peg.1516  -tgccttcctctgctttttttgtatgtatacacttgaatggaaataaaaaaatggtataat-at
632516.3.peg.126   -tgccttcctctgctttttttgtatgtatacacttgaatggaaataaaaaaatggtataat-at
1121259.3.peg.236  -tgccttcctctgctttttttgtatgtatacacttgaatggaaataaaaaaatggtataat-at
1387557.3.peg.1656 -tgccttcctctgctttttttgtatgtatacacttgaatggaaataaaaaaatggtataat-at
351627.8.peg.2134  ttgccttcctctgctttttttgcatgtatacgcttgaataaagctttaaaagtgatataattaa
                    ** ** * ************* ******** *******  *  *  **** ** ****** *

31899.10.peg.1901  aatt-Gggactaaaaagaaaggtatatggataaaattgtccc-gctggagaatgagagagt-ATGGAAAGTT
608506.3.peg.1243  tatttgggactaaaaatcatgatatatggataaatttgtccc-gctggagaatgagagagttATGGAAAGTT
632518.3.peg.1271  tatttgggactaaaaatcatgatatatggataaatttgtccc-gctggagaatgagagagttATGGAAAGTT
632292.3.peg.1411  tatt-gggaccaaaaagcaagatatgtggataaatttgtccc-gctggagaatgagagagttATGGAAAGTT
632348.3.peg.1378  aatt-gggactaaaaagaaagttatatggataaaattgtccc-gctggagaatgagagagt-ATGGAAAGTT
632335.8.peg.1516  aatt-gggactaaaaagaaagagatgtggataaaattgtccc-gctggagaatgagagagt-ATGGAAAGTT
632516.3.peg.126   aatt-gggactaaaaagaaagagatgtggataaaattgtccc-gctggagaatgagagagt-ATGGAAAGTT
1121259.3.peg.236  aatt-gggactaaaaagaaagttatatggataaaattgtccc-gctggagaatgagagagt-ATGGAAAGTT
1387557.3.peg.1656 tatt-gggactaaaaagcaagagatatggataaaattgtccc-gctggagaatgagagagttATGGAAAGTT
351627.8.peg.2134  tagt-gggacaaaactatatgggggatggataaaactgtccctgatggagtaatgatgag--ATGGAAGATT
                    * * ***** ***    * *     ********  ****** * ***** *     ***  ******  **
```

31899.10.peg.1783 **(Athe_1295)** ***hydDECBA*** **(BifH)** **Rex-binding site** **(score=5.32, EMSA+) (repressor)**

31899.10.peg.1783	Caldicellulosiruptor bescii strain DSMZ 6725	632292.3.peg.1518	Caldicellulosiruptor hydrothermalis 108
632348.3.peg.1487	Caldicellulosiruptor kronotskyensis 2002	608506.3.peg.1367	Caldicellulosiruptor obsidiansis OB47
632335.8.peg.1402	Caldicellulosiruptor kristjanssonii 177R1B	1222016.3.peg.1510	Caldicellulosiruptor changbaiensis strain CBS-Z
1121259.3.peg.134	Caldicellulosiruptor acetigenus DSM 7040	351627.8.peg.2037	Caldicellulosiruptor saccharolyticus DSM 8903
632516.3.peg.214	Caldicellulosiruptor lactoaceticus 6A	1214564.3.peg.2349	Caldicellulosiruptor sp. F32
1387557.3.peg.1536	Caldicellulosiruptor sp. Wai35.B1	1387555.3.peg.1622	Caldicellulosiruptor sp. Rt8.B8
632518.3.peg.1167	Caldicellulosiruptor owensensis OL		

```
31899.10.peg.1783  agatgcccatcatctatgggagataaatgaaagggaatttttattgaaagtgatatactgtatacaatatttttcaattaaattctccaaaattta
1222016.3.peg.1510 tgatgcacatcatctatgggatattaacgaaaaggagtttttattgaaagtgatatactgtatacaatatttttcaagtaaattctccaaaattta
351627.8.peg.2037  tgatgcacatcatctatgggatattaacgaaaaggagtttttattgaaagtgatatactgtatacaatatttttcaagtaaattctccaaaattta
1214564.3.peg.2349 tgatgcacatcatctgtgggatattaacgaaaaggaatttttattgaaagtgatatactgtatacaatatttttcaagtaaattctccaaaattta
632348.3.peg.1487  agatgcccatcatctgtgggagataaatgaaagggaatttttattgaaagtgatatactgtatacaatatttttcaattaaattctccaaaattta
1121259.3.peg.134  agatgctcatcatttgtgggagataaacgaaagagaatttttattgaaagtgatatactgtatacaatatttttcgattaaattctccaaaattta
632516.3.peg.214   agatgctcatcatttgtgggagataaacgaaagagaatttttattgaaagtgatatactgtatacaatatttttcgattaaattctccaaaattta
632335.8.peg.1402  agatgctcatcatttgtgggagataaacgaaagagaatttttattgaaagtgatatactgtatacaatatttttcgattaaattctccaaaattta
1387557.3.peg.1536 agatgctcatcgtctgtgggaaataaacgaaagagagtttttattgaaagtgatatactgtatacaatatttttcgattaaattcttcaaaattta
632518.3.peg.1167  agatgctcatcatctgtgggagataaacgaaagagagtttttattgaaagtgatatactgtatacaatatttttcgattaaattctccaaaattta
608506.3.peg.1367  agatgcccatcatctggggggagataaacgaaagagagtttttattgaaagtgatatactgtatacaatatttttcgattaaattctccaaaattta
632292.3.peg.1518  agatgctcatcatctatgggaaataaacgaaagagaatttttattgaaagtgatatactgtatacaatatttttcgattaaattcttcaaaattta
1387555.3.peg.1622 agatgcacatcatttgtgggaaattaacgaaagagagtttttattgaaagtgatatactgtatacaatattcttcaaataatttttcgaaatttc
                    ***** **** * *  **** ** ** ****  ** *********************************** *** * *** ** * * ******
```

```
31899.10.peg.1783  tacttcatttataacccgttgtatgctacaatattaacAgtgg-ttttaactccatatgttaaatttctaacaatagaaggggatgcagatttATGTCT
1222016.3.peg.1510 tacttcatttataccctattatatgttacaatagagaaagtag-gtgtaattgaatatgttaaatttctaacaatagaaggggatgcgatttaATGTCT
351627.8.peg.2037  tacttcatttataccctattatatgttacaatagtgaaagtag-gtgtaattgaatatgttaaatttctaacaatagaaggggatgcgatttaATGTCT
1214564.3.peg.2349 tacttcatttataccctattatatgttacaatagtgaaagtag-gtgtaattgaatatgttaaatttctaacaatagaaggggatgcgatttaATGTCT
632348.3.peg.1487  tacttcatttataacccattgtatgttacaatattaacagtag-ctttaactccatatgttaaatttctaacaatagaaggggatgcagatttATGTCT
1121259.3.peg.134  tacttcatttataacccgttgtatgttacaatattaacagtag-ttttaactcagtatgttaaatttctaacaatagaaggggatgcagatttATGTCT
632516.3.peg.214   tacttcatttataacccgttgtatgttacaatattaacagtag-ttttaactcagtatgttaaatttctaacaatagaaggggatgcagatttATGTCT
632335.8.peg.1402  tacttcatttataacccgttgtatgttacaatattaacagtag-ttttaactcagtatgttaaatttctaacaatagaaggggatgtagatttATGTCT
1387557.3.peg.1536 tacttcatttataaccctttgtatgttacaatattaacagtag-ttttaactcagtatgttaaatttctaacaatagaaggggatgcagatttATGTCT
632518.3.peg.1167  tacttcatttataaccctttgtatgttacaatattaacagtag-ttttaactcagtatgttaaatttctaacaatagaaggggatgcagacttATGTCT
608506.3.peg.1367  tacttcatttataatcctttgtatgttacaatattaacagtag-ttttaactcagtatgttaaatttctaacaatagaaggggatgtagatttATGTCT
632292.3.peg.1518  tacttcatttataacccgttgtatgttacaatattaacagtag-ttttaactcagtatgttaaatttctaacaatagaaggggatgcagattcATGTCT
1387555.3.peg.1622 tacttcaattatcctccactgtgtgttacaatattgaaagtaaagtataattgaatatgttaaacttctaacaatagaaggggatgcaaattaATGTCT
                   ******* ****   *   * * ** *******   * ***    * *** *   ********* ********************     * ******
```

31899.10.peg.564 (Athe_0168) *copG-hydG* Rex-binding site (score=5.4, EMSA+) (repressor)

31899.10.peg.564 Caldicellulosiruptor bescii strain DSMZ 6725
1121259.3.peg.539 Caldicellulosiruptor acetigenus DSM 7040
632335.8.peg.185 Caldicellulosiruptor kristjanssonii 177R1B
632516.3.peg.1228 Caldicellulosiruptor lactoaceticus 6A
632348.3.peg.2528 Caldicellulosiruptor kronotskyensis 2002
608506.3.peg.179 Caldicellulosiruptor obsidiansis OB47
1387557.3.peg.441 Caldicellulosiruptor sp. Wai35.B1
632292.3.peg.2575 Caldicellulosiruptor hydrothermalis 108
1222016.3.peg.2755 Caldicellulosiruptor changbaiensis strain CBS-Z
1214564.3.peg.1816 Caldicellulosiruptor sp. F32
351627.8.peg.2616 Caldicellulosiruptor saccharolyticus DSM 8903
632518.3.peg.119 Caldicellulosiruptor owensensis OL
1387555.3.peg.498 Caldicellulosiruptor sp. Rt8.B8

```
31899.10.peg.564   aagaaaaaattctcaatagtacact-tttaaatagtacacttttaatattctaaagaatatgttataatattaAcaaaca
1214564.3.peg.1816 aaagaaaaaat---------------tctcaatagtacacttttaatattctaaagagtatgttataatattaacaaata
351627.8.peg.2616  aaagaaaaaat---------------cctcaatagtgcacttttaatattctaaaaagtatgttataatattaacaaata
1387557.3.peg.441  aaagaaaaaat---------------tctaaatagtcgacttctaatatttcaaaaagtatgttataatattaacaaaca
632292.3.peg.2575  tagaaaagtat---------------ttctattagaatactttcaaaatctcaaaaagtatgttataatattaacaaaca
1222016.3.peg.2755 tagaaaagtat---------------ttctattagaatactttcaaaaactcaaaaagtatgttataatattaacaaaca
632518.3.peg.119   tagaaaagtat---------------ttctattaggatactttcaaaatctcaaaaagtatgttataatattaacaaact
632348.3.peg.2528  aataaaagcat---------------ttctattagaatactttcaaaatctcaaaaactatgttataatattaacaaaca
1387555.3.peg.498  atgaagaaaaa---------------atttttcaggtagctttaaaaagtttcgaaagtatgttataatattaacaaaca
632335.8.peg.185   aataaagatatttttgcttaaggggattaaaatagtg---ttttaatttactaaaaactatgttataatattaacaaaca
632516.3.peg.1228  aataaagatatttttgcttaaggggattaaaatagtg---ttttaatttactaaaaactatgttataatattaacaaaca
1121259.3.peg.539  aataaagatatttttgctgaaggagattaaaatagtg---ttttaatttactaaaaactatgttataatattaacaaaca
608506.3.peg.179   aa-aaagataattttacttgacgaa--cgaaatagta---tttcaatttactaaaaaatatgttataatattaacaaact
                       *                            **     **  **        * * ********************

31899.10.peg.564   aaatatttgatcctgacttagagggcatcatggcctgataaggaggggaatgggat---tgatgtcaacaataagacataaatccacctc
1214564.3.peg.1816 aaatatttgctcctgacttagagggcaaaatggcctgataaggaggggga-tgtaaa---agatg--aagagaaagatat----ccacctc
351627.8.peg.2616  aaatatttgctcctgacttagagggcaaaatggcctgataaggaggggga-tgtgaa---agatg--aaaagaaagatat----ccacctc
1387557.3.peg.441  aaatatttgatcctgacttagagggcaaaatggcctgataaggaggggga-tgt-aa---agatg--aagaagaagatat----ccacctc
632292.3.peg.2575  aaatatttgctcctgacttagagggcaaaatggcctgataaggaggggaagtggattggggaaagcgagaaaaaggcttgcatccacctc
1222016.3.peg.2755 aaacatttgctcctgacttagagggcatcgtggcctgataaggaggggatgtg--------aaaatgaagagaaagat---atccacctc
632518.3.peg.119   aaacatttgctcctgacttagagggcaaaatggcctgataaggaggggat-tggattgaaagcagaaaaaataaggcttgtatccacctc
632348.3.peg.2528  aaatatttgatcctgacttagagggtatcatggcctgataaggaggggaatgggat----gatgtcaataataagacataaatccacctc
1387555.3.peg.498  gaatatttgatcctgacttagagggcattgcagcctgatagggagggggttattggtt-tgatgctaaaaaggaaggctatgtccacctc
632335.8.peg.185   aaacatttgctcctgacttagagggcaaagtagcctgataaggaggggaattggattgaagtcagcaagaataaggcttgtatccacctc
632516.3.peg.1228  aaacatttgctcctgacttagagggcaaagtagcctgataaggaggggaattggattgaagtcagcaagaataaggcttgtatccacctc
1121259.3.peg.539  aaacatttgctcctgacttagagggcaaagtagcctgataaggaggggaattggattgaagtcagcaagaataaggcttgtatccacctc
608506.3.peg.179   aaacatttgctcctgacttagagggcaaaatggcctgataaggaggggga-ttggattggaaactgcaaaaataaggtttgtatccacctc
                    ** ***** *************** *     ******** *******                   *     *

31899.10.peg.564   ttttggctgaaaaggt--------------taaaaagtcaggagaggtgg---ttttattttacctgaaaa-agggaatggtgaagcaaaTTGGAAAG
1214564.3.peg.1816 ttttggctaaagataaggctttggacac-gtaaaaggtcaggagaggtggattttttatttttacattaaa-ggagaatggtgctttt--ATGGAAAG
351627.8.peg.2616  ttttggctaaagatgaggctttggacac-gtaaaaggtcaggagaggtggattttttatttttacattaaa-ggagaatggtgctttt--ATGGAAAG
1387557.3.peg.441  ttttggctaaggatgatgctttggatac-gtaaaaggtcaggagaggtggattttttatttttacattaaa-agggaatgatgctttt--ATGGAAAG
632292.3.peg.2575  ttttggctaaaaagg-----ttaa-------aaaaagtcaggagaggtgg---atttattttatctaaaaa-agggaacggtgaagtggaTTGGAAAG
1222016.3.peg.2755 ttttggctaaagcgaagcttttgatgaaagcaaaatgtcaggagaggtgg---ttttattttttctaaaaagagggaacggtgaagtggaTTGGAAAG
632518.3.peg.119   ttttgtttaaaaagg-----ttaaa------ataaagtcaggagaggtgg---atttatttttttagaaaaaggaga----tgcatttttATGGAAAG
632348.3.peg.2528  ttttggctgaaaagg-----ttaa---------aaagtcaggagaggtgg---ttttattttacctaaaaa-agggaatggtgaagtgaaTTGGAAAG
1387555.3.peg.498  ttttgattgaa----------------------aaggtcaggagaggtgg---atttatttt--ttgaaaa---ggaacggtgaagtaaaTTGGAAAG
632335.8.peg.185   ttttggctaaaaagg-----ttaa--------aaaagtcaggagaggtgg---atttatttttttagaaaa--ggggaggcagttt----ATGGAAAG
632516.3.peg.1228  ttttggctaaaaagg-----ttaa--------aaaagtcaggagaggtgg---atttatttttttagaaaa--ggggaggcagttt----ATGGAAAG
1121259.3.peg.539  ttttggctaaaaagg-----ttaa--------aaaagtcaggagaggtgg---atttatttttttagaaaa--gggaatggtgttttt--ATGGAAAG
608506.3.peg.179   ttttgactaaataag-----gtta--------agaagtcaggagaggtgg---atttattttttta-aaga--gggaatggtgttttt--ATGGAAAG
                                                                                                             ********
```

31899.10.peg.1563 (Athe_1082) *echABCDEF-hypABF* (MBH) Rex-binding site (score=5.07, EMSA+) (activator)

31899.10.peg.1563	Caldicellulosiruptor bescii strain DSMZ 6725	608506.3.peg.1551	Caldicellulosiruptor obsidiansis OB47
632348.3.peg.1701	Caldicellulosiruptor kronotskyensis 2002	1121259.3.peg.1086	Caldicellulosiruptor acetigenus DSM 7040
632292.3.peg.1747	Caldicellulosiruptor hydrothermalis 108	632516.3.peg.1466	Caldicellulosiruptor lactoaceticus 6A
632335.8.peg.1191	Caldicellulosiruptor kristjanssonii 177R1B	351627.8.peg.1692	Caldicellulosiruptor saccharolyticus DSM 8903
632518.3.peg.971	Caldicellulosiruptor owensensis OL	1222016.3.peg.1877	Caldicellulosiruptor changbaiensis strain CBS-Z

```
31899.10.peg.1563  aaatgttgaacaaaaaatagtaagaatttcacctgtattctggggtttgttaacaaattaactttttgcatactgtatacaagaca
632335.8.peg.1191  aaatgttgaacaaaaaataataagaatttcacctgtattctggagtttgttaacaaattaactttttgcatacagtatacaagaca
632516.3.peg.1466  aaatgttgaacaaaaaatagtaagaatttcacctgtattctggagtttgttaacaaattaactttttgcatacagtatacaagaca
1121259.3.peg.1086 aaatgttgaacaaaaaatagtaagaatttcacctgtattctggagtttgttaacaaattaactttttgcatactgtatacaagaca
632348.3.peg.1701  aaatgttgaacaaaaaatagtaagaatttcacctgtatgctggggtttgttaacaaattaactttttgcatactgtatacaagaca
632518.3.peg.971   aaatattgaacaaaaaatagcagctatttttcaagtattctggagtttgttaacaaattaactttttgcatactgtatacaagaca
608506.3.peg.1551  aaatattgaacaaaaaatagcagctatttttcaagtattctggagtttgttaacaaattaactttttgcatactgtatacaagaca
632292.3.peg.1747  aaatgttgaacaaaaaatagcagctatttttcaagtattctggagtttgttaacaaattaactttttgcatactgtatacaagaca
351627.8.peg.1692  aaatgttgaacaaaaaagagatagaagctttcttctattctgaactttgttaacaaattaactttttgcatactgtatacaagacc
1222016.3.peg.1877 aaatgttgaacaaaaaagagatagaagctttcttctattctgaactttgttaacaaattaactttttgcatactgtatacaagacc
                   **** ************ *      *  *  *   *** ***   ************************** ***********

31899.10.peg.1563  aagattatatgttataatctttaatAgcaatagtttatcatcataaatagaaaggggcaagaaagaaacagATGGCGAG
632335.8.peg.1191  aagattatatgctataatctttaatagcaatagtttatcatcacaaatggaaaggggcaagaaagaaacagATGGCGAG
632516.3.peg.1466  aagattatatgctataatctttaatagcaatagtttatcatcacaaatagaaaggggcaagaaagaaacagATGGCGAG
1121259.3.peg.1086 aagattatatgctataatctttaatagcaatagtttatcatcacaaatagaaaggggcaagaaagaaacagATGGCGAG
632348.3.peg.1701  aagattatatgttataatctttaatagcaatagtttatcatcataaatagaaaggggcaagaaagaaacagATGGCGAG
632518.3.peg.971   aagattatatgttataatctttaatagcaatagtttatcatcacaaatagaaaggggcaaggaagaaactgATGGTGAA
608506.3.peg.1551  aagatcatatgttataatctttaatagcaatagtttatcatcacaaatagaaaggggcaaggaagaaactgATGGTGAA
632292.3.peg.1747  aagattatatgttataatctttaatagcaatagtttatcatcacaaatagaaaggggcaaggaagaaactgATGGTGAA
351627.8.peg.1692  acgagaatgtgttataatctt-aatagcaatagtttatcatcacaaagagaaaggggcaatattgga-catATGGAGTA
1222016.3.peg.1877 acgagaatgtgttataatctt-aatagcaatagtttatcatcacaaagagaaaggggcaatattgga-catATGGAGTA
                   * **  ** ** ********* ******************** ***  ***********    * * *  **** *
```

31899.10.peg.1096 (Athe_0654) *rex-thrC* Rex-binding site (score=4.84, EMSA+) (repressor)

31899.10.peg.1096	Caldicellulosiruptor bescii strain DSMZ 6725
632335.8.peg.684	Caldicellulosiruptor kristjanssonii 177R1B
632348.3.peg.2079	Caldicellulosiruptor kronotskyensis 2002
1387557.3.peg.919	Caldicellulosiruptor sp. Wai35.B1
1121259.3.peg.874	Caldicellulosiruptor acetigenus DSM 7040
632292.3.peg.2096	Caldicellulosiruptor hydrothermalis 108
1222016.3.peg.689	Caldicellulosiruptor changbaiensis strain CBS-Z
608506.3.peg.673	Caldicellulosiruptor obsidiansis OB47
632516.3.peg.320	Caldicellulosiruptor lactoaceticus 6A
351627.8.peg.1362	Caldicellulosiruptor saccharolyticus DSM 8903
1214564.3.peg.1415	Caldicellulosiruptor sp. F32
1387555.3.peg.817	Caldicellulosiruptor sp. Rt8.B8
632518.3.peg.583	Caldicellulosiruptor owensensis OLZ

```
31899.10.peg.1096  aagcaaaactttcaaagttttaaaagata-----gtaatca-aaagcttagcgagtataataaacgatatgacggaaaaagAagataac
1222016.3.peg.689  aagcgaaactttcaaaatttctaaaagaca-----gcaacca-gaaactaagcgagtacaacaaacgatttgacggcaaaagaagataa-
351627.8.peg.1362  aagcaaaactttcaaaatttctaaaagaca-----gcaacca-gaaactaagcgagtacaacaaacgatttgacggcaaaagaggataa-
632348.3.peg.2079  aagcaaaactttcaaagttttaaaagata-----gtaatca-aaagcttagcgagtataataaacgatatgacggaaaaagaagataac
632335.8.peg.684   aggcgaaactttcaaagttttaaaagata-----gcaatca-aaagatagtagagtacaacaagagatttgacgggaaaagaagataac
632516.3.peg.320   aggcgaaactttcaaagttttaaaagata-----gcaatca-aaagatagtagagtacaacaagagatttgacgggaaaagaagataac
1121259.3.peg.874  aagcaaaactttcaaagttttaaaagata-----gcaatca-aaagatagtagagtacaacaagagatttgacgggaaaagaagataac
1387557.3.peg.919  aagcaaaactttcaaagttttaaaagata-----gcaatca-aaagatagtagagtacaacaaacgatttgacgggaaaagaagataac
632292.3.peg.2096  aagcaaaactttcaaagttttaaaagaca-----gcaatca-aaagcttagtgaatacaacaaacgatttgacgggaaaagaagataac
608506.3.peg.673   aagcaaaactttcaaagttttaaaagaca-----gcaatca-aaagcttagtgagtacaacaaacgatttgacgggaaaagaagataac
632518.3.peg.583   aagcaaaactttcaaagttttaaaagaca-----gcaatca-aaagctcagtgagtacaacaaacgatttgacgggaaaagaagataac
1214564.3.peg.1415 ggttaaaaaaggcagtatagcgcaaggcaaagacagcaaccagagacttaagtgagtacagtagacgatttgatggcagaagaaattaaa
1387555.3.peg.817  aaataaataaaacagttttattgaaggca---------------gatcaaagtgac--------------------------------at
                        **     **   *     ** *                  *       **                                 *

31899.10.peg.1096  agcctgttttaaaattgaaacaggcttttaatttagaaaaagtatgtcaaaaaaatatcaaaagaagataagggggagaaaagTTGTTC
1222016.3.peg.689  agcctgttttaaaatcaaaacaggcttttaattta-agaaggtatgtcaaaaaaatatcaaa-gaggatgaggaggataaaaaTTGTTT
351627.8.peg.1362  agcctgttttaaaatcaaaacaggcttttaattta-agaaggtatgtcaaaaaaatatcaaa-gaggatgaggaggataaaaaTTGTTT
632348.3.peg.2079  agcctgttttaaaattgaaacaggcttttaatttagaaaaagtatgtcaaaaaaatatcaaaagaagataagggggagaaaagTTGTTC
632335.8.peg.684   agcctgttttgagtttgaaacaggcttttaatttagaagaagtatgtcaaaaaaatatcaaaaaaagataagggggagaaaagTTGTTC
632516.3.peg.320   agcctgttttgagtttgaaacaggcttttaatttagaagaagtatgtcaaaaaaatatcaaaaaaagataagggggagaaaagTTGTTC
1121259.3.peg.874  agcctgttttgagtttgaaacaggcttttaatttagaagaagtatgtcaaaaaaatatcaaaagaaaataagggggagaaaagTTGTTC
1387557.3.peg.919  agcctgttttaaaattgaaacaggcttttaatttagaaaaagtatgtcaaaaaaatatcaaaagaagataagggggagaaaaaTTGTTC
632292.3.peg.2096  agcctgttttaaaattgaaacaggcttttaatttagaagaagtatgtcaaaaaaatatcaaaagaagataagggggaggaaaaTTGTTC
608506.3.peg.673   agcctgttttaaaattgaaacaggcttttaatttagaagaagtatgtcaaaaaaatatcaaaagaagataagggggaggaaaaTTGTTC
632518.3.peg.583   agcctgttttaaaattgaaacaggcttttaatttagaagaagtatgtcaaaaaaatatcaaaataagataagggggagaaaaaTTGTTC
1214564.3.peg.1415 agcctgttttgaatttaaaacaggcttttaattta-agaaggtatgtcaaaaaaatatcaaa-gaggatgaggaggataaaaaTTGTTT
1387555.3.peg.817  agcctgttttaaaataaaaacaggcttttaatttg-agtgagtatgtcaaaaaaatatcaaa-taagatggggaggataaagaTTGTTT
                   ********** *  *  *****************  *    *********************  * ***  ** ***  **  ******
```

31899.10.peg.1279 (Athe_0820) *gorSL-moaD-moeB-tupABC-moeAA-moaAC* (GOR) Rex-binding site (score=5.16, EMSA+)(repressor)

31899.10.peg.1279 Caldicellulosiruptor bescii strain DSMZ 6725
632518.3.peg.729 Caldicellulosiruptor owensensis OL
632292.3.peg.1958 Caldicellulosiruptor hydrothermalis 108
1387557.3.peg.1091 Caldicellulosiruptor sp. Wai35.B1
351627.8.peg.1096 Caldicellulosiruptor saccharolyticus DSM 8903
1214564.3.peg.2121 Caldicellulosiruptor sp. F32
1387555.3.peg.949 Caldicellulosiruptor sp. Rt8.B8
1121259.3.peg.1855 Caldicellulosiruptor acetigenus DSM 7040
1222016.3.peg.2155 Caldicellulosiruptor changbaiensis strain CBS-Z
632348.3.peg.1931 Caldicellulosiruptor kronotskyensis 2002
608506.3.peg.831 Caldicellulosiruptor obsidiansis OB47
632335.8.peg.2049 Caldicellulosiruptor kristjanssonii 177R1B
632516.3.peg.1869 Caldicellulosiruptor lactoaceticus 6A

```
31899.10.peg.1279  aaatt-gcaagtag-cctcttgtaaactttcattttaggtgatataataagattGttaaattttaaacgaaatttaattttgagtgattaaaggttt
351627.8.peg.1096  aaatt-gcaagaagacatattgtaaactttcatatcaagtggtataataagattgttaaaaaataaacaaa--tacaagctgaactgtttagtttgga
1214564.3.peg.2121 aaatt-gcaagaagacatattgtaaactttcatatcaagtggtataataagattgttaaaaaataaacaaa--tacaagctgaactgtttagtttgga
1222016.3.peg.2155 aaatt-gcaagaagacatattgtaaactttcatatcaagtggtataataagattgttaaaaaataaacaaa--tacaagctgaactgtttagtttaga
1387557.3.peg.1091 aaatttgcaagtag-cctcttgtaaactttcatgttggatgatataataagattgttaaattttaaacgaaatttggattttaggtgattaaatgtct
1121259.3.peg.1855 aaatt-gcaagtag-cctattgtaaactttcattttggatgatataataagattgttaaattttaaacgaaatttggactttgggtgattaaaggtct
632518.3.peg.729   aaatttgcaagtag-cctcttgtaaactttcattttgaatgatataataagattgttaaattttaaacgaaatttggatttggggcgattaaggagct
632292.3.peg.1958  aaatt-gcaagtag-cctcttgtaaactttcattttaggtgatataataagattgttaaattttaaacgaaatttggattttgggtgattaaaggtct
632335.8.peg.2049  aaatt-gcaagtag-cctattgtaaactttcattttggatgatataataagattgttaaattttaaacgaaatttggactttgggtgattaaaggtct
632516.3.peg.1869  aaatt-gcaagtag-cacattgtaaactttcattttgaatgatataataagattgttaaattttaaacaaaatttg-actttgggtgattaaaggtct
608506.3.peg.831   aaatt-gcaagtag-cctcttgtaaactttcattttggatgatataataagattgttaaattttaaacgaaatttggattttgggtgattaaggaact
632348.3.peg.1931  aaatt-gcaagtag-cctcttgtaaactttcattttaggtgatataataaaattgttaaattttaaacgaaatttgattttgggtgattaaaggttt
1387555.3.peg.949  aaatt-gcaagaaaacatattgtaaagtttcatttcaggtggtataataagattgttaaaaaataaacaaaa--aaatatttgattgttctggggctt
                   ***** ***** *  *   ******* ****** *    ** ******** *********   ***** **         *     * *

31899.10.peg.1279  aatattttgggtattaattaaaatagtatttttgg-agg--ataa-taatata-agaaagttttaaatatgat-tttacaggtggtgatttaa-TTGCCA
351627.8.peg.1096  gtccatgtgggtataaatagctatatactgcctatttgagaataaacagtgtgataattaatcaattcattttattaataggtggtgatttaa-TTGCCA
1214564.3.peg.2121 gtccatgtgggtataaatagctatatactgcctatttgagaataaacagtgtgataattaatcaattcattttattaataggtggtgatttaa-TTGCCA
1222016.3.peg.2155 gtccgtgtgggtataaatagttatatactgcctatttgggagtaaatattgttataattaatcaattcg-----ttaataggtggtgatttaa-TTGCTA
1387557.3.peg.1091 actattttgggtattaattaatatagcacttttgtcagg--acaa-taattttggaaagttgaaaatatgat-tttacaggtggtgatttaa-TTGCCA
1121259.3.peg.1855 actattttgggtattaattaaaatagtatttttag-agg--atta-taatttg-ggaagttttaaaatatgat-tttacaggtggtgatttaa-TTGCCA
632518.3.peg.729   gatattttgggtattaattaaattagtattttttg-agg--ataa-taattttggaaagttttaaaatatgat-tttacaggtggtgatttaaaTTGCCC
632292.3.peg.1958  aatattttgggtattaattaaaatagtatttttgg-agg--ataa-taatttt-ggaaagttttaaatatgat-tttacaggtggtgatttaa-TTGCCA
632335.8.peg.2049  actattttgggtattaattaaaatagtatttttgtcaga--ataa-taattttggaa-ggttgaaaatatgat-tttacaggtggtgatttaa-TTGCCA
632516.3.peg.1869  attatattgggtaataattaaaatagtgtttttatcaaa--ataa-tagttttggaa-gttttaaaatatgat-tttacaggtggtgatttaa-TTGCCA
608506.3.peg.831   gatattttgggtattaattaaattagtatttttattagaggataa-taattttagaaagttttaaaatatgat-tttacaggtggtgatttaa-TTGCCA
632348.3.peg.1931  aatattttgggtattaattaaaatagtatttttggagga---taa-taatattggaaagttttaaa-tatgat-tttacaggtggtgatttaa-TTGCCA
1387555.3.peg.949  ----ttatgggtataaatacttatatattgctcaatttaggaaaa-tactattaagatagagtcaaatatga-----ataggtggtgatttaaaTTGCCA
                          ******  ***     **                   *  * * *    *       *            * ************** **** *
```

31899.10.peg.2681 (Athe_2126) *serC-serA-Athe_2124-23-22* Rex-binding site (score=4.96, EMSA+) (potential repressor)

31899.10.peg.2681	Caldicellulosiruptor bescii strain DSMZ 6725	1387557.3.peg.2537	Caldicellulosiruptor sp. Wai35.B1
632292.3.peg.684	Caldicellulosiruptor hydrothermalis 108	632516.3.peg.2512	Caldicellulosiruptor lactoaceticus 6A
632348.3.peg.540	Caldicellulosiruptor kronotskyensis 2002	1222016.3.peg.786	Caldicellulosiruptor changbaiensis strain CBS-Z
632518.3.peg.1934	Caldicellulosiruptor owensensis OL	1387555.3.peg.925	Caldicellulosiruptor sp. Rt8.B8
632335.8.peg.514	Caldicellulosiruptor kristjanssonii 177R1B	1214564.3.peg.133	Caldicellulosiruptor sp. F32
608506.3.peg.2038	Caldicellulosiruptor obsidiansis OB47	351627.8.peg.1450	Caldicellulosiruptor saccharolyticus DSM 8903
1121259.3.peg.1945	Caldicellulosiruptor acetigenus DSM 7040		

```
31899.10.peg.2681  ttgtttata--ttaccaattatttgttgcaagg-ggttgttataaataaacaaaaacatcctgctttttttatgtttaaaatgtataataataa
632348.3.peg.540   ttgtttata--ttaccaattatttgttgcaagg-ggttgttataaataaacaaaaacattctgcttttatatgtttaaaatgtataataataa
632292.3.peg.684   ttgtttata--ttaccaattatttgttgcaagg-agttgttttaaataaacaaaaacattctacttttatatgtttaaaatgtataataataa
1121259.3.peg.1945 ttgtttata--ttacaagtt-tttgccatgaga-agttgttttaaatagacaaaaatattctatttttatgtgttcaaaatgtataataataa
632516.3.peg.2512  ttgtttata--ttacaagtt-tttgccatgaga-agttgttttaaatagacaaaaatattctatttttatgtgttcaaaatgtataataataa
632335.8.peg.514   ttgtttata--ttacaagtt-tttgccatgaga-agttgttttaaatagacaaaaatattctatttttatgtgttcaaaatgtataataataa
1387557.3.peg.2537 ttgtttata--atacaagtt-tt-gctgtgaag-agttgttttaaatagacaaagatattttattttcatgtgtataaaatgtataataataa
608506.3.peg.2038  ttgtttata--ttaccaatt-tttgttgcaagg-agttgttttaaatagacaaagacattctatttttatatgtttaaaatgtataataataa
632518.3.peg.1934  ctgtttata--ttaccatgtattaacaaca--g-aattgttttaattatacaaaaaacgttgtctcttattgtgtacaaaatgtataataataa
1222016.3.peg.786  ttgtttcttcatgttttgtttgtcacagcagtgcaattgttttagattgacaaaaaaatatgctttggttgtgttgaaaatgtataataataa
1214564.3.peg.133  ttgtttcttcatgttttgtttgtcacagcagtgcaattgttttagattgacaaaaaaatatgctttggttgtgttgaaaatgtataataataa
351627.8.peg.1450  t--ttattttgtatcttatttgtcacagcagagcaattgttttatattgacaaaagaatgtattttggctgtgtagaaaatgtataataataa
1387555.3.peg.925  ttgtttatt--tgtttttgttgtt-cagcaggataattgttgtttattgacaaaaagttctggcctgatagtgtttaaaatgtataataataa
                      **  *           *  *             ***** *   *  *****     *      *     ***  *****************

31899.10.peg.2681  gattgtcaaattattgacattgtcacacgcattagatataatcaactTaaaacaaatt--gagtttgtttgaaaggggtagaagaaagaatATGAGA
632348.3.peg.540   gattgtcaaattattgacattgtcagacgcattagatataatcaacttaaaacaaatt--gagtttgtttgaaaggggtagagttta-aatATGAGA
632292.3.peg.684   gattgtcaaattattgacattgtctgacgcattagatataatcaacttaaaacaaatt--gagtttgtttgaaaggggtagagttta-aatATGAGA
1121259.3.peg.1945 gattgtcaaattattgacattgtctgatacattggatataatcaacttaaaacacatt--gagtttgcttgaaaggggtagagttta-aatATGAGA
632516.3.peg.2512  gattgtcaaattattgacattgtctgatacattggatataatcaacttaaaacacatt--gagtttgcttgaaaggggtagagttta-aatATGAGA
632335.8.peg.514   gattgtcaaattattgacattgtccgatacatttgatataatcaacttaaaatacatt--gagttt-cttgaaaggggtagagttta-aatATGAGA
1387557.3.peg.2537 gattgtcaaattattgacattgtctgatacattagatataatcaacttaaaacacatt--gagattgcttgaaaggggtagagttta-gatATGAGA
608506.3.peg.2038  gattgtcaaattattgacattctctgatacattagatataatcaacttaaaataaatt--gagtttgcttgaaaggggtagagttta-agtATGAGA
632518.3.peg.1934  gattgtcaaattattgacattgtatcgaacattagatataatcaacttaaaataaatga-gagaaaagctgaaaggggtagtagtta-gatATGAGA
1222016.3.peg.786  gattgtcaaattattgacattgctaagtagtttagatataatcaaacttaaataatcattcagttttgttgaaaggggtagaaaattaaccATGAGA
1214564.3.peg.133  gattgtcaaattattgacattgctaagtagtttagatataatcaaacttaaataatcattcagttttgttgaaaggggtagaaaattaaccATGAGA
351627.8.peg.1450  gattgtcaaattattgacattgctaagtagtttagatataatttaaacataaataatcattcagttttattgaaaggggtagaaaattaaccATGAGA
1387555.3.peg.925  gattgtcaaattattgacattgccgggtagattggatataatcaaacttaaataattattcagttttgttgaaaggggtagaatggtaaatATGAGA
                   *********************          ** ******** **    ***  *      **      ************          ******
```

31899.10.peg.2269 (Athe_1758) *tusA-dsrE-oxd-X* Rex-binding site (score=5.26) (potential repressor)

31899.10.peg.2269	Caldicellulosiruptor bescii strain DSMZ 6725	1387557.3.peg.2039	Caldicellulosiruptor sp. Wai35.B1
632292.3.peg.1063	Caldicellulosiruptor hydrothermalis 108	1387555.3.peg.1136	Caldicellulosiruptor sp. Rt8.B8
632516.3.peg.1600	Caldicellulosiruptor lactoaceticus 6A	608506.3.peg.904	Caldicellulosiruptor obsidiansis OB47
632335.8.peg.1912	Caldicellulosiruptor kristjanssonii 177R1B	1222016.3.peg.1009	Caldicellulosiruptor changbaiensis strain CBS-Z
1121259.3.peg.620	Caldicellulosiruptor acetigenus DSM 7040	351627.8.peg.2488	Caldicellulosiruptor saccharolyticus DSM 8903
632348.3.peg.1015	Caldicellulosiruptor kronotskyensis 2002	1214564.3.peg.417	Caldicellulosiruptor sp. F32
632518.3.peg.1595	Caldicellulosiruptor owensensis OL		

```
31899.10.peg.2269  ttttgaatccttaaaagttttgtgtttaa---gaatgttattatt--taacaatctaattttaaaaaaatttttttgca
632348.3.peg.1015  ttttgaatccttaaaagttttgtgtttaa---gaatgttattatt--taacaatctaattttaaaaaaatttttttgca
632518.3.peg.1595  tgttgaatgcttaaaagcttttgtgttcaa---aaatgttattatt--taacaatctgatttaaaaaaa-tttttttgca
608506.3.peg.904   tgttaaatgcttaaaagcttttgtgttcaa---aaatgttattatt--taacaatctgattttaaaaaa-tttttttgca
632292.3.peg.1063  tgttgaatgcttaaaagcttttgtgttcaa---aaatgttattatt--taacaatctgattttaaaaaa-tttttttgca
632335.8.peg.1912  ttttgaatgcttaaaagcttttgtgttcaa---aaatgttattatt--taacaatctgatttttaaaaa-ttttttttaca
1121259.3.peg.620  ttttgaatgcttaaaagcttttgtgttcaa---aaatgttattatt--taacaatctgatttttaaaaa-ttttttttaca
632516.3.peg.1600  ttttgaatgcttaaaagcttttgtgttcaa---aaatgttattatt--taacaatctgatttttaaaaa-ttttttttaca
1387557.3.peg.2039 ttttgaatgcttaaaagcttttgtgtttta---aaatgttattatt--taacaatctaatttcaaaa----tttttttaaa
351627.8.peg.2488  ttaaactcatttgagacattgtattttgtgtaaaagtgttattatt--taacaaagtgttttgtaaagaattttattgtg
1214564.3.peg.417  ttaaactcatttgagacattgtattttgtgtaaaagtgttattatt--taacaaagtgttttgtaaagaattttattgtg
1222016.3.peg.1009 ttaaactcatttgagacattgtattttgtgtaaaagtgttattatt--taacaaagtgttttgtaaagaattttattgtg
1387555.3.peg.1136 ttaaagataattagtatttttcttttttt--aaattagattgagcagttataatttgttcaatagcgttattattttaa
                   *         **   *  ** * * **       * *   ***      * * **  *  *    *      **  **

31899.10.peg.2269  gaagtttgttaaataaatctcatgcaaatggggtaacaatataa-aaggtaaaaataaaaatttatggaggtgtgtttaaaATGGCAGA
632348.3.peg.1015  gaagtttgttaaataaatctcatgaaaatggggtaacaatataa-aaggtaaaaataaaaatttatggaggtgtatttaaaATGGCAGA
632518.3.peg.1595  gaagtttgttaaataaatctcatgcaaatagggtaacaatataa-aagg-aaaaataaaaatttatggaggtgtgatcaagATGGCAGA
608506.3.peg.904   gaagtttgttaaataaatctcatgcaaatggggtaacaatataa-aaggtaaaaataaaaatttatggaggtgcgatcaagATGGCAGA
632292.3.peg.1063  gaagtttgttaaataaatctcatgcaaatggggtaacaatataa-aaggtaaaaataaaaatttatggaggtgcgatcaagATGGCAGA
632335.8.peg.1912  gaagtttgttaaataaatctcatgcaaatagggtaacaatataa-aaggtaaaaacaaaaatttatggaggtgcgatcaagATGGCAGA
1121259.3.peg.620  gaagtttgttaaataaatctcatgcaaatagggtaacaatataa-aaggtaaaaacaaaaatttatggaggtgcgatcaagATGGCAGA
632516.3.peg.1600  gaagtttgttaaataaatctcatgcaaatagggtaacaatataa-aaggtaaaaacaaaaatttatggaggtgcgatcaagATGGCAGA
1387557.3.peg.2039 gagctttttaaataaatctcatgcagatatagtaacaatataa-aaag--aagataaaagtttgtggaggtgtagtcaagATGGCAGA
351627.8.peg.2488  tgcttttgttaaataaatcctattgccaaggggtaacaatataa-aaagttaaaaataaagtttttggaggtgtg-tgattGTGGCAGA
1214564.3.peg.417  tgcttttgttaaataaatcctattgccaaggggtaacaatataa-aaagttaaaaataaagtttttggaggtgtg-tgattGTGGCAGA
1222016.3.peg.1009 tgcttttgttaaataaatcctattgccaaggggtaacaatataa-aaagttaaaaataaagtttttggaggtgcg-tgattGTGGCAGA
1387555.3.peg.1136 caaagtattta--taaactttttaaagat-gggtaatagtataacaaaattgaaaataaaatctctggaggtgt--taaaaTTGGCAGA
                        *  ***  ****     *    *    **** * ***** **     * *  *** * * ********   * *   *******
```

31899.10.peg.1987 (Athe_1494) *pta-ackA* Rex-binding site (score=4.93) (repressor)

31899.10.peg.1987	Caldicellulosiruptor bescii strain DSMZ 6725	1214564.3.peg.534	Caldicellulosiruptor sp. F32
632292.3.peg.1326	Caldicellulosiruptor hydrothermalis 108	1387557.3.peg.1745	Caldicellulosiruptor sp. Wai35.B1
632348.3.peg.1292	Caldicellulosiruptor kronotskyensis 2002	351627.8.peg.2219	Caldicellulosiruptor saccharolyticus DSM 8903
1121259.3.peg.324	Caldicellulosiruptor acetigenus DSM 7040	632518.3.peg.1360	Caldicellulosiruptor owensensis OL
1222016.3.peg.1289	Caldicellulosiruptor changbaiensis strain CBS-Z	608506.3.peg.1157	Caldicellulosiruptor obsidiansis OB47
632516.3.peg.35	Caldicellulosiruptor lactoaceticus 6A	1387555.3.peg.1341	Caldicellulosiruptor sp. Rt8.B8
632335.8.peg.1603	Caldicellulosiruptor kristjanssonii 177R1B		

```
31899.10.peg.1987  taattcttgtatacaagatacaatttgtttataatatat-aaGgtgc-aatatgtcaaataataaacgaaaggagtattaaaaa-ATGGCAT
1222016.3.peg.1289 tacttcttgtatacaagatacaatttggtttataatatat-agggtgtaataatgttaaataataaacgaaaggggctttt-aaagATGGCAT
351627.8.peg.2219  tacttcttgtatacaagatacaatttggtttataatatat-aaggtgtgataatgttaaataataaacgaaaggggctttt-aaagATGGCAT
1214564.3.peg.534  tacttcttgtatacaagatacaatttagtttataatatat-aaggtgtga-aatgttaaataataaacgaaaggggcttttttaaaaATGGCAT
632348.3.peg.1292  taattcttgtatacaagatacaatttgtttataatatat-aaggtgc-aatatgtcaaataataaacgaaaggagtattaaaaa-ATGGCAT
632292.3.peg.1326  taattcttgtatacaagatacaatttgtttataatatat-aaggtgc-aatatgtcaaataataaacgaaaggagtatcaaaaa-ATGGCAT
632518.3.peg.1360  taattcttgtatacaagatacaatttgtttataatatattaaggtac-aatatgacaaataataaacgaaaggagtatcaaaaa-ATGGCTT
608506.3.peg.1157  taattcttgtatacaagatacaatttgtttataatatat-aaggtactaatatgacagataataaacgaaaggagcatcaaaaaaATGGCTT
1121259.3.peg.324  taattcttgtatacaagatacaacttgtttataatatat-aaggtgc-gatatgtcaaataataaacgaaaggagtatcaaaa--ATGGCTT
632335.8.peg.1603  taattcttgtatacaagatacaacttgtttataatatat-aaggtgc-aatatgtcaaataataaacgaaaggagtatcaaaaa-ATGGCTT
632516.3.peg.35    taattcttgtatacaagatacaatttgtttataatatat-aaggtgc-gatatgtcaaataataaacgaaaggagtatcaaaaa-ATGGCTT
1387557.3.peg.1745 taattcttgtatacaagatacaatttgtttataatatat-aaggtgc-aatatgacaaataataaacgaaaggagtatcaaaaa-ATGGCTT
1387555.3.peg.1341 taattcttgtatacaagatacaatttagtttataatatat-aaggtgcaaaaatgttaaacaataaacgaaaggggctttcagag-ATGTCAT
                   ** ******************** **  ************ * ***                                        *** * *
```

31899.10.peg.1337 (Athe_0874) *porGDAB* Rex-binding site (score=5.27, EMSA+) (potential repressor)

31899.10.peg.1337	Caldicellulosiruptor bescii strain DSMZ 6725
632518.3.peg.779	Caldicellulosiruptor owensensis OL
1387557.3.peg.1151	Caldicellulosiruptor sp. Wai35.B1
632348.3.peg.1877	Caldicellulosiruptor kronotskyensis 2002
632516.3.peg.344	Caldicellulosiruptor lactoaceticus 6A
632292.3.peg.1904	Caldicellulosiruptor hydrothermalis 108
608506.3.peg.881	Caldicellulosiruptor obsidiansis OB47
1121259.3.peg.1808	Caldicellulosiruptor acetigenus DSM 7040
632335.8.peg.718	Caldicellulosiruptor kristjanssonii 177R1B
351627.8.peg.1619	Caldicellulosiruptor saccharolyticus DSM 8903
1222016.3.peg.951	Caldicellulosiruptor changbaiensis strain CBS-Z
1387555.3.peg.2012	Caldicellulosiruptor sp. Rt8.B8
1214564.3.peg.1454	Caldicellulosiruptor sp. F32

```
31899.10.peg.1337  gcaatccct-aatccaaatatatgcaattatggtgtattcaaaaatatgttgattttttaacattcaaaatatataataaaaagGta--a
351627.8.peg.1619  tcaagccatataac-aaataattacaattatggtgtattcaaaaatatgttgattttttaacattcaaaatatataataaaaaggta--g
1214564.3.peg.1454 tcaagccatataac-aaataattacaattatggtgtattcaaaaatatgttgattttttaacattcaaaatatataataaaaaggta--g
1222016.3.peg.951  tcaagccatataac-aaataattacagttatggtgtattcaaaaatatgttgattttttaacatttaaaatatataataaaagggta--g
1121259.3.peg.1808 gcaatccct-aatccaaatatatacaattatggtgtattcaaaaatatgttgattttttaacattcaaaatatataataaaaaggta--a
632335.8.peg.718   gcaatccct-aatccatatatatacaattatggtgtattcaaaaatatgttgattttttaacattcaaaatatataataaaaaggta--a
632348.3.peg.1877  gcaatccct-aatccatatatatacaattatggtgtattcaaaaatatgttgattttttaacattcaaaatatataataaaaaggta--a
632516.3.peg.344   gcaatccct-aatccaaatatatacaattatggtgtattcaaaaatatgttgattttttaacattcaaaatatataataaaaaggta--a
632518.3.peg.779   gcaatccct-aatccaaatatatgcaattatggtgtattcaaaaatatgttgattttttaacattcaaaatatataataaaaaggta--a
608506.3.peg.881   gcaatccccaaaccaaatatatacaattatggtgtattcaaaaatatgttgattttttaacattcaaaatatataataaaaaggta--a
632292.3.peg.1904  gcaatccct-gatccaaatatatgcaattatggtgtattcaaaaatatgttgattttttaacattcaaaatatataataaaaaggta--g
1387557.3.peg.1151 tcaagccctttaac-aaataattacaattatggtgtattcaaaaatatgttgattttttaacattcaaaatatataataaaaaggta--a
1387555.3.peg.2012 -caattttaatattcaatttttaagaagtgaatgtgttaagaaaagctttg--ttatcaaggtcagtttccagtaatcaattggtacta
                    ***       *   *  *   *          *** ** * ***    ***  ** * **  *          **** **  ****
```

```
31899.10.peg.1337  gtagcctta--tagtgataagtttagctttttcaaataaagttttcaaatgtttt---tgagggaggtttgaacATGGGC
351627.8.peg.1619  t--gttaaa--tattgattctctttaatagttcaaataaaagactgtaacgtttttgaaaaaggaggtacaaacATGGGC
1214564.3.peg.1454 t--gttaa---tattgattctctttagtagttcaaataaaagactgtaacgtttttaaaaaaggaggtacgaacATGGGC
1222016.3.peg.951  t--attaaa--tattgattctctt-gatagttcaaataaaagactgtaacgtttt-aaaaaaggaggtacgaacATGGGC
1121259.3.peg.1808 gtagcctta--tagtgatgagtttatctttttcaaataaagttttcaaatgtttttt-tgagggaggtttgaacATGGGC
632335.8.peg.718   gtagcctta--tagtgatgagtttatctttttcaaataaagttttcaaatgtttttt-tgagggaggtttgaacATGGGC
632348.3.peg.1877  gtagcctta--tagtgatgagtttatctttttcaaataaagttttcaaatgtttttt-tgagggaggtttgaacATGGGC
632516.3.peg.344   gtagctttc--tagtgatgagtttatctttttcaaataaagttttcaaatgtttttt-tgagggaggtttgaacATGGGC
632518.3.peg.779   gtaaccttc--tagtgatgagtttatctttttcaaataaagttttcaaatgtttttt-taagggaggtttgaacATGGGT
608506.3.peg.881   gtaaccttt--tattgatgagtttatctttt-caaataaagttttcaaatgtttttt-taagggaggtttgaacATGGGC
632292.3.peg.1904  t--gttaaa--tattgattctctttaatagttcaaataaaagactgtaacgtttttgaaaaaggaggtacgaacATGGGC
1387557.3.peg.1151 gtggcctta--tagtggtgagttttatttttcaaataaagttttcaaatgtttttt-taagggaggtttgaacATGGGC
1387555.3.peg.2012 ataatttaatgtaccaattgagatatgaagttttaagtgatttgataatcatattatacgagggaggtacgaaaATGGGC
                              **    *     *      *   **   *       *   * **     * ******   ** *****
```

31899.10.peg.2899 (Athe_2324) *glmM* Rex-binding site (score=4.89) (repressor)

31899.10.peg.2899	Caldicellulosiruptor bescii strain DSMZ 6725	1214564.3.peg.879	Caldicellulosiruptor sp. F32
632348.3.peg.244	Caldicellulosiruptor kronotskyensis 2002	351627.8.peg.701	Caldicellulosiruptor saccharolyticus DSM 8903
1222016.3.peg.2584	Caldicellulosiruptor changbaiensis strain CBS-Z	1121259.3.peg.1350	Caldicellulosiruptor acetigenus DSM 7040
1387557.3.peg.2755	Caldicellulosiruptor sp. Wai35.B1	632335.8.peg.2547	Caldicellulosiruptor kristjanssonii 177R1B
632292.3.peg.226	Caldicellulosiruptor hydrothermalis 108	608506.3.peg.2249	Caldicellulosiruptor obsidiansis OB47
632518.3.peg.2197	Caldicellulosiruptor owensensis OL	1387555.3.peg.2409	Caldicellulosiruptor sp. Rt8.B8
632516.3.peg.964	Caldicellulosiruptor lactoaceticus 6A	1387555.3.peg.95	Caldicellulosiruptor sp. Rt8.B8

```
31899.10.peg.2899  tacctgaggtaaaa-aattttgttcatgctgcctga--------aaaaatttctctggcagctttttttgtctttaaaa
1214564.3.peg.879  aa-ctactggattaaaattt-atactccacactccagt------aaagttattctttgcc-aaagaaccaact---aaa
351627.8.peg.701   aaactactggattaaaattt-atactccacactccagt------aaagttattctttgcccaaagaaccaact---aaa
632335.8.peg.2547  aaactactggattaaaattt-atactccgcactccagt------aaaattactctctgat-caagaaccaact---aaa
632518.3.peg.2197  tatttaaggtagtttgctttggtgattgctgcccgggg------aag--tttctgtggcagctttcactttttctaaaa
608506.3.peg.2249  tatttcaggtaatttgctttggtgagtgctgcccggtg------aaaatttcccttggcagcttttattttttctaaaa
632348.3.peg.244   tacctgaggtaaaa-aattttgttcatgctgcctga--------aaaaatttctctggcagctttttttgtctttaaaa
1222016.3.peg.2584 tacttgagataaga-agttt-attcttaatgcctgg--------aaagttttctacggcagctttttt-gtctttaaaa
632292.3.peg.226   tacttgagataaga-agttt-attcttgatgcctgg--------aaagttttctacggcagctttttt-gtctttaaaa
1387557.3.peg.2755 tacttgaggtaaga-aattt-attcttgctgctgtg--------gaggttttatacggcagctttttttgtctttaaaa
632516.3.peg.964   tacctgaggtaaga-gtttt-gttcttgctgcctgg--------aaagttttctacggcagctttttttgtctttaaaa
1387555.3.peg.2409 tacttcttacttatcacttgccttaacattagcacaatgt-tgcaaaggaaaagatgtatacatatctaatggtcag--
1387555.3.peg.95   tactttttacttatcacttgccttaacattagcacaatgt-tgcaaaggaaaagatgtatacatatctaatggtcag--
1121259.3.peg.1350 agtttttaa--taacacacactttaacatttatggggcatatgagattgcaaagcttgcagctttttttgtctttaaaa
                       *                 *                       *

31899.10.peg.2899  taaatgttcacattgttattttttcaaacataggtatataatagataaAgtaataaatcactttcatatgaaaggggacgagaATGGGAA
1214564.3.peg.879  taaatgttcacattgttatttttcgaacataggtatataatagataaagtaataaatcactttcatatgaaaggggacgagaATGGGAA
351627.8.peg.701   taaatgttcacattgttatttttcgaacataggtatataatagataaagtaataaatcactttcatatgaaaggggacgagaATGGGAA
632335.8.peg.2547  taaatgttcacattgttattttttcaaacataggtatataatagataaagtaataaatcattcttctgtgaaaggggacgagaATGGGAA
632518.3.peg.2197  taaacgttcacattgtgatttcttaaacataggtatataatagataaagtaacaggtta-tttcttgtgaaaggggacgggaATGGGAA
608506.3.peg.2249  tgaatgttcacattgtgatttcttaaacataggtatataatagataaagtaatgaatcactttcatatgaaaggggacgagaATGGGAA
632348.3.peg.244   taaatgttcacattgttattttttcaaacataggtatataatagataaagtaataaatcactttcatatgaaaggggacgagaATGGGAA
1222016.3.peg.2584 taaatgttcacattgttatttttcgaacataggtatataatagataaagtaataaatcactttcatatgaaaggggacgagaATGGGAA
632292.3.peg.226   taaatgttcacattgttatttttcgaacataggtatataatagataaagtaataaatcactttcatatgaaaggggacgagaATGGGAA
1387557.3.peg.2755 taaatgttcacattgttattttttcaaacataggtatataatagatagagtaataaatcattctattgtgaaaggggacgagaATGGGAA
632516.3.peg.964   taaatgttcacattgttattttttcaaacataggtatataatagataaagtaataaatcacattcttatgaaaggggacgagaATGGGAA
1387555.3.peg.2409 caaatgt--agattgttattttttatgtatggttatataatagataaagtaaaataaaat-ctactgaaaaaggaggagattgATGGGAA
1387555.3.peg.95   caaatgt--agattgttattttttatgtatggttatataatagataaagtaaaataaaat-ctactgaaaaaggaggagattgATGGGAA
1121259.3.peg.1350 taaatgttcacattgttattttttcaaacataggtatataatagataaagtaataaatcattcttctgtgaaaggggacgagaATGGGAA
                     ** **   * ***** **** *     ** * ************* *****      *    *   *    ***** ***      *******
```

Other Central Carbohydrate Metabolism genes without reconstructed TF regulons

31899.10.peg.984 (Athe_0552) ***glk***

31899.10.peg.984	Caldicellulosiruptor bescii strain DSMZ 6725
632348.3.peg.2197	Caldicellulosiruptor kronotskyensis 2002
608506.3.peg.557	Caldicellulosiruptor obsidiansis OB47
632518.3.peg.454	Caldicellulosiruptor owensensis OL
632292.3.peg.2200	Caldicellulosiruptor hydrothermalis 108
1387569.3.peg.727	Thermoanaerobacter cellulolyticus NA10
1121259.3.peg.1667	Caldicellulosiruptor acetigenus DSM 7040
632335.8.peg.2212	Caldicellulosiruptor kristjanssonii 177R1B
1387557.3.peg.798	Caldicellulosiruptor sp. Wai35.B1
1214564.3.peg.1570	Caldicellulosiruptor sp. F32
351627.8.peg.886	Caldicellulosiruptor saccharolyticus DSM 8903
1222016.3.peg.2344	Caldicellulosiruptor changbaiensis strain CBS-Z
1387555.3.peg.2387	Caldicellulosiruptor sp. Rt8.B8

```
31899.10.peg.984   ctaaaaagtctattt-tacttaatctcttttgttgatataataaaa-tCaaaa-tcttaagaattggaaaggagagagaaaGTGTATTACATA
608506.3.peg.557   ctaaaaagtctattt-tacttaatcccttttgttgatataataaaa-tcaaaa-tcttagggattggaaaggagagagaagGTGTATTACATA
632518.3.peg.454   ctaaaaagtctattt-tacttaatccattttgttgatataataaaa-tcaaaa-tcttaagggttgggaaggagagagaagGTGTATTACATA
1214564.3.peg.1570 ctaaaaagtatattt-tacttaatctcttttgttgatataataaaa-tcaaaa-tcttaagaattggaaaggagagagaagGTGTATTACATA
351627.8.peg.886   ctaaaaagtatattt-tacttaatctcttttgttgatataataaaa-tcaaaa-tcttaagaattggaaaggagagagaagGTGTATTACATA
1222016.3.peg.2344 ctaaaaagtatattt-tacttaatctcttttgttgatataataaaa-tcaaaa-tcttaagaattggaaaggagagagaagGTGTATTACATA
632292.3.peg.2200  ctaaaaagtctattt-tacttaatctcttttgttgatataataaaa-tcaaaa-tcttaagaattagaaaggagagagaagGTGTATTACATA
1387569.3.peg.727  ctaaaaagtatattt-tacttaatctcctttgttgatataataaaa-tcaaaa-tcttaagatttagaaaggagagagaagGTGTATTACATA
1387555.3.peg.2387 ------------tgt-tacttag-cttttttcttgatataataaaa-tcaaaaatcataaagattagagaggagagaaaagGTGTATTACATA
632348.3.peg.2197  ctaaaaagtctattt-tacttaatctcttttgttgatataataaaa-tcaaaa-tcttgagaattggaaaggagagagaaaGTGTATTACATA
1121259.3.peg.1667 cacaaaaagctattt-tacttaacctcttttgttgatataataaaa-tcaaaa-tcttaaggattggaaaggagagagaagGTGTATTACATA
1387557.3.peg.798  ataaatga--tattt-tacttag-cttttttgttgatataataaaa-tcaaaaatcttaaagattagagaggagagaaaagATGTATTACATA
632335.8.peg.2212  tttttcaatctatttacaatcaagaatttattgt---tataataaaaatcaaaa----gacaaagttgaaaggagagagaagGTGTATTACATA
                               * *  * * *      * ** ** ********** ******           * *  ******** **  ***********
```

31899.10.peg.1059 (Athe_0619) ***pgi2-Athe_0620-fba2***

31899.10.peg.1059	Caldicellulosiruptor bescii strain DSMZ 6725	1387557.3.peg.882	Caldicellulosiruptor sp. Wai35.B1
1121259.3.peg.832	Caldicellulosiruptor acetigenus DSM 7040	632518.3.peg.547	Caldicellulosiruptor owensensis OL
632335.8.peg.644	Caldicellulosiruptor kristjanssonii 177R1B	1387569.3.peg.2014	Thermoanaerobacter cellulolyticus NA10
632516.3.peg.2379	Caldicellulosiruptor lactoaceticus 6A	1222016.3.peg.648	Caldicellulosiruptor changbaiensis strain CBS-Z
632348.3.peg.2115	Caldicellulosiruptor kronotskyensis 2002	1214564.3.peg.14	Caldicellulosiruptor sp. F32
608506.3.peg.636	Caldicellulosiruptor obsidiansis OB47	1387555.3.peg.783	Caldicellulosiruptor sp. Rt8.B8
632292.3.peg.2131	Caldicellulosiruptor hydrothermalis 108	351627.8.peg.1330	Caldicellulosiruptor saccharolyticus DSM 8903

```
31899.10.peg.1059  acttctttagaagtggtgctttcct---att-ttttgtgtggttataaattcctattttttgtgtggttataaa
1121259.3.peg.832  agttctttagaagtggtgctttcct---att-ttttgtgtggttttgaa-------------------------
632335.8.peg.644   agttctttagaagtggtgctttcct---att-ttttgtgtggttttgaa-------------------------
632516.3.peg.2379  agttctttagaagtggtgctttcct---att-ttttgtgtggttttgaa-------------------------
1387557.3.peg.882  acttctttagaattggtgctttcct---att-ttttgtgtggttttgaa-------------------------
632518.3.peg.547   acttctttagaagtggtgcttttct---att-ttttgtgtggttttgaa-------------------------
632292.3.peg.2131  actttcttggaagtggtgctttcct---att-ttttgtgtggttttgaa-------------------------
632348.3.peg.2115  acttctttagaagtggtgctttcct---att-ttttgtgtggttatgaa-------------------------
608506.3.peg.636   acttctttaga-gtggtgctttccttttatt-ttttatgtggttttgaa-------------------------
1387569.3.peg.2014 atagatgttgatgaggtgtttaagtgagcctattttatatattttaacatgtctaatttg----ggtcttgaaa
1387555.3.peg.783  -------------gagtgttt---tgtggctatggcat--------------------------ggctttgaaa
351627.8.peg.1330  atagatgttgatgaggtgttcaaatgagaacattttatatattgtaacacatttgtttt-----ggcttttaaa
1222016.3.peg.648  atagatgttgatgaggtgttcaaatgagatcattttatatattgtaacacatttgcttt-----ggcttttaaa
1214564.3.peg.14   atagatgttgatgaggtgttcaaatgagatcattttatatattgtaacacatttgtttt-----ggcttttaaa
                                  *** *    *       *    *

31899.10.peg.1059  gttgaaaaactgaaaaattgttatattatgaaatAgaaaatttt-aaagaaaggagtctgggaaa----ATGCTTGATAATTTAG
1121259.3.peg.832  gttgaaaaactgaaaaattgttatattatgaaatagaaaatttt-aaagaaaggagtctgggaaa----ATGCTTGATAATTTAG
632335.8.peg.644   gttgaaaaactgaaaaattgttatattatgaaatagaaaatttt-aaagaaaggagtctgggaaa----ATGCTTGATAATTTAG
632516.3.peg.2379  gttgaaaaactgaaaaattgttatattatgaaatagaaaatttt-aaagaaaggagtctgggaaa----ATGCTTGATAATTTAG
1387557.3.peg.882  gttgaaaaactgaaaaattgttatattatgaaatagaaaatttt-aaagaaaggagcctgggaaa----ATGCTTGATAATTTAG
632518.3.peg.547   gttgaaaaactgaaaaattgttatattatgaaatagaaaattttaaggaaaggagtcagggaaa----ATGCTTGATAATTTAG
632292.3.peg.2131  gttgaaaaactgaaaaattgttatattatgaaatagaaaatttt-atagaaaggagtcagggaaa----ATGCTTGATAATTTAG
632348.3.peg.2115  gttgaaaaactgaaaaattgttatattatgaaatagaaaatttt-aaagaaaggagtctgggaaa----ATGCTTGATAATTTAG
608506.3.peg.636   gttgaaaaactgaaaaattgttatattatgaaatagaaaatttt-atagaaaggagtcagggaaa----ATGCTTGATAATTTAG
1387569.3.peg.2014 gttgaaaaactgaaaaattattatattatgaaatagaaaatttt-taagaaaggagaaataatca-aagATGCTTGATAACTTAG
1387555.3.peg.783  gttgaaaaaatgaaaaattattatattatgaaataggaaatttt-taagaaaggagaaatggggagaagATGCTTGACAATCTTG
351627.8.peg.1330  gttgaaaaactgaaaaattattatattatgaaatagaaaaattt-aaagaaaggagaaatgaaga-gctATGCTTAATAACTTAG
1222016.3.peg.648  gttgaaaaactgaaaaattattatattatgaaatagaaaaattt-aaagaaaggagaaatgaaga-gccATGCTTGATAACTTAG
1214564.3.peg.14   gttgaaaaactgaaaaattattatattatgaaataggaaaattt-aaagaaaggagaaatgaaga-gctATGCTTGATAACTTAG
                   ********** ********* *************** *** ***    ********        *    ****** * **  * *
```

31899.10.peg.2336 (Athe_1824) *ppi-pfk*

31899.10.peg.2336	Caldicellulosiruptor bescii strain DSMZ 6725
632348.3.peg.948	Caldicellulosiruptor kronotskyensis 2002
632292.3.peg.995	Caldicellulosiruptor hydrothermalis 108
608506.3.peg.1753	Caldicellulosiruptor obsidiansis OB47
632335.8.peg.952	Caldicellulosiruptor kristjanssonii 177R1B
1121259.3.peg.688	Caldicellulosiruptor acetigenus DSM 7040
632516.3.peg.987	Caldicellulosiruptor lactoaceticus 6A
632518.3.peg.1664	Caldicellulosiruptor owensensis OL
1387557.3.peg.2103	Caldicellulosiruptor sp. Wai35.B1
1222016.3.peg.1997	Caldicellulosiruptor changbaiensis strain CBS-Z
351627.8.peg.2551	Caldicellulosiruptor saccharolyticus DSM 8903
1387555.3.peg.1078	Caldicellulosiruptor sp. Rt8.B8
1387569.3.peg.1635	Thermoanaerobacter cellulolyticus NA10
1214564.3.peg.754	Caldicellulosiruptor sp. F32

```
31899.10.peg.2336  ttgattcacctgaaaatattgataacttcaacaaagttgagtaa-----tgGcttctatgtttaac
1222016.3.peg.1997 ttgattcacctacagctatagacggctttaaaaaagtagaatgagaa-acggcttcaatttttaac
351627.8.peg.2551  tggattcacctacagacatagatgactttaaaaaagtagaatgagaa-acggcttcaatttttaac
632348.3.peg.948   ttgattcacctgaaaatattgataacttcaacaaagttgagtaa-----tggcttctatgtttaac
1121259.3.peg.688  ttgattcgcctgaaaatattgataactttaacaaagttgagtaa-----tggcttctatgtttaac
632516.3.peg.987   ttgattcgcctgaaaatattgataactttaacaaagttgagtaa-----tggcttctatgtttaac
632335.8.peg.952   ttgattcgcctgaaaatattgataactttaacaaagttgagtaa-----tggcttctatgtttaac
632292.3.peg.995   ttgattcacctgaaaatattgataactttaacaaagttgagtaa-----tggcttctatgtttaac
608506.3.peg.1753  ttgactcgcctgaaaatattgataactttaacaaagttgagtaa-----tggcttctatgtttaac
632518.3.peg.1664  ttgattcgcctgaaaatattgataactttaacaaagttgagtaa-----tggcttctatgtttaac
1387557.3.peg.2103 ttaattcgcctgaaaatattgataactttaacaaagttgagtaa-----cggcttctatgtttaac
1387555.3.peg.1078 tcgactctccggaatccatagatacttttaaaaaggtgcaataaaaa-agggcttctatggctaac
1387569.3.peg.1635 tagattctccaacatctatagacaattttaaaaaaagtggaataagaatatggcttctatggctaac
1214564.3.peg.754  tggattcacctacagatatagatgactttaaaaaagtagaatgacag-acggcttcaatgtttaac
                   *  * ** **   *   ** **    ** ** ** **  * * *      ****** **   ****

31899.10.peg.2336  aatttagataaactaaaaatac-----ggacgggagggattttgaATGCCAAAAGAAGG
1222016.3.peg.1997 -attaaaataaactgaaaatttaacaaaagcgggagggatctgaaATGCCAAAAGAGGG
351627.8.peg.2551  -attaagataaactgaaaatttaacaaaagcgggagggatctaaaATGCCAAAAGAGGG
632348.3.peg.948   aatttagataaactaaaaatac-----ggacgggagggattttgaATGCCAAAAGAAGG
1121259.3.peg.688  -gttaaaataaactaaaaatac-----ggacgggagggattttgaATGCCAAAAGAAGG
632516.3.peg.987   -gttaaaataaactaaaaatac-----ggacgggagggattttgaATGCCAAAAGAAGG
632335.8.peg.952   -gttaaaataaactaaaaatac-----ggacgggagggattttgaATGCCAAAAGAAGG
632292.3.peg.995   tattgaaataaactaaaaatat-----ggacgggagggattttgaATGCCAAAAGAAGG
608506.3.peg.1753  tattgaaataaactaaaaatac-----agacgggagggattttgaATGCCAAAAGAAGG
632518.3.peg.1664  tattgaaataaactaaaaatat-----ggacgggagggatttaaaATGCCAAAAGAAGG
1387557.3.peg.2103 tattgaaataaactaaaaatac-----ggacgggagggattttgaATGCCAAAAGAAGG
1387555.3.peg.1078 -attaaaataaactaaaaattttacttgatcgggagggatttgaaATGCCAAAAGAAGG
1387569.3.peg.1635 -attaaaataaactaaaaatttaactaaagcgggagggattcaaaatcccaaaagaggg
1214564.3.peg.754  -attaagataaactgaaaatttaacaaaagcgggagggatctaaaaggccaaaagaggg
                     ** * ******* *****          **********    **  ***********
```

31899.10.peg.1750 (Athe_1263) ***Athe_1263-64-pfk-pyk-Athe_1267***

31899.10.peg.1750	Caldicellulosiruptor bescii strain DSMZ 6725
632292.3.peg.1550	Caldicellulosiruptor hydrothermalis 108
1387557.3.peg.1504	Caldicellulosiruptor sp. Wai35.B1
632348.3.peg.1520	Caldicellulosiruptor kronotskyensis 2002
632518.3.peg.1135	Caldicellulosiruptor owensensis OL
632516.3.peg.247	Caldicellulosiruptor lactoaceticus 6A
1121259.3.peg.102	Caldicellulosiruptor acetigenus DSM 7040
632335.8.peg.1366	Caldicellulosiruptor kristjanssonii 177R1B
608506.3.peg.1399	Caldicellulosiruptor obsidiansis OB47
1214564.3.peg.2317	Caldicellulosiruptor sp. F32
1222016.3.peg.1542	Caldicellulosiruptor changbaiensis strain CBS-Z
351627.8.peg.2005	Caldicellulosiruptor saccharolyticus DSM 8903
1387555.3.peg.1657	Caldicellulosiruptor sp. Rt8.B8
1387569.3.peg.1035	Thermoanaerobacter cellulolyticus NA10

```
31899.10.peg.1750   AAATGTTTGGATAGAAGATATAGATTCTTGAttttagaccgttttgttataatatttgaaAagaggtgaga-tgaaatATGGAAAATGAAAATGTA
632348.3.peg.1520   AAATGTTTGGATAGAAGATATAGATTCTTGAttttagaccgttttgttataatatttgaaaagaggtgagagtgagacATGGAAAATGAAAATGTA
632292.3.peg.1550   AAATGTTTGGATAGAAGATATAGATTCTTGAttttagaccgttttgttataatatttgaaaagaggtgagattgagacATGGAAAATGAAAATGTA
1387557.3.peg.1504  AAATGTTTGGATAGAAGATATAGATTCTTGAttttaggccgttttgttataatatttgaaaagaggtgagattgagatATGGAAAATGAAAATGTA
632518.3.peg.1135   AAATGTTTGGATAGAAGATATAGATTCTTGAttttagaccgttttgttataatatttgaaaagaggtgaga-tgaaatATGGAAAATGAAAATGTA
608506.3.peg.1399   AAATGTTTGGATAGAAGATATAGATTCTTGAttttagactgttttgttataatatttgaaaagaggtgaga-tgaaatATGGAAAATGAAAATGTA
632516.3.peg.247    AAATGTTTGGATAGAAGATATAGATTCTTGAttttagaccgttttgttataatatttgaaaagaggtgaga-tgaaatATGGAAAATGAAAATGTA
632335.8.peg.1366   AAATGTTTGGATAGAAGATATAGATTCTTGAttttagaccgttttgttataatatttgaaaagaggtgaga-tgaaatATGGAAAATGAAAATGTA
1121259.3.peg.102   AAATGTTTGGATAGAAGATATTGATTCTTGAttttagaccgttttgttataatatttgaaaagaggtgaga-tgaaatATGGAAAATGAAAATGTA
1214564.3.peg.2317  AAATGTGTGGCTTGAAGATTTAGATTCTTGAttttaggtatttttgttataatatttgaaaagaggtgaga---aacaataATGAACGAAAATTTA
1222016.3.peg.1542  AAATGTGTGGCTTGAAGATTTAGATTCTTGAttttaggtatttttgttataatatttgaaaagaggtgaga---aacaataATGAACGAAAATTTA
351627.8.peg.2005   AAATGTGTGGCTTGAAGATTTAGATTCTTGAttttaggcatttttgttataatatttgaaaagaggtgaga---aacaataATGAACGAAAATTTA
1387569.3.peg.1035  AAATGTGTGGCTTGAAGATTTAGATTCTTGAttttaggcttttttgttataatatttgaaaagaggtgaga---aaggataATG---GAAAATGTA
1387555.3.peg.1657  AAATGTATGGCTTGAAGATTTAGATTCTTGAttttaggcatttttgttataatatttgaaaagaggtgagg---aaagataATGAGTGAAAATGCA
                    ****** *** * ****** * ****************   *****************************    *            ******  *
```

31899.10.peg.2606 (Athe_2060) *tkt*

31899.10.peg.2606	Caldicellulosiruptor bescii strain DSMZ 6725	632518.3.peg.1851	Caldicellulosiruptor owensensis OL
632335.8.peg.565	Caldicellulosiruptor kristjanssonii 177R1B	1214564.3.peg.926	Caldicellulosiruptor sp. F32
1121259.3.peg.2306	Caldicellulosiruptor acetigenus DSM 7040	1387555.3.peg.2160	Caldicellulosiruptor sp. Rt8.B8
632516.3.peg.1044	Caldicellulosiruptor lactoaceticus 6A	351627.8.peg.1502	Caldicellulosiruptor saccharolyticus DSM 8903
632348.3.peg.640	Caldicellulosiruptor kronotskyensis 2002	1387569.3.peg.1841	Thermoanaerobacter cellulolyticus NA10
608506.3.peg.1959	Caldicellulosiruptor obsidiansis OB47	1387557.3.peg.2455	Caldicellulosiruptor sp. Wai35.B1
632292.3.peg.754	Caldicellulosiruptor hydrothermalis 108	1222016.3.peg.861	Caldicellulosiruptor changbaiensis strain CBS-Z

```
31899.10.peg.2606  aaggttttctg--tttgagttaaaaatgatcaatatgatataatg-tcaat--Atgatataatataaaggaaaaagatagt
632348.3.peg.640   aaggttttctg--tttgagttaaaaatgatcaatatgatataatggtcaat--atgatataatataaaggaaaaagatagt
1214564.3.peg.926  gatgttcgtgaggttttaggtgaaggtttttctgtttgagttaaaaatgatttaatggtataatataaagaaaaaaaga-agt
351627.8.peg.1502  gatgttcgtgaggttttaggtgaaggtttttctgtttgagtaaaaaatgatttaatggtataatataaagaaaaaaaga-agt
1222016.3.peg.861  gatgttcgtgaggttttaggtgaaggtttttctgtttgagttaaaaatgatttaatggtataatataaagaaaaaaaga-agt
1387569.3.peg.1841 gatgttcgtgaggttttaggtgaaggtttttctgtttaagtaaaaaatgatttaatggtataatgtaaagaaaaaaaga-agt
1387557.3.peg.2455 gatgttcgtgaggttttaggtgaaggtttttctgtttgagtaaaaaatgatttaatggtataatataaagaaaaaaaga-agt
1387555.3.peg.2160 gacgttcgagaggttttgggtgaaggtttttctgtttga-ttaaaaataatttaatggtataatataaagaaaaaaaga-aat
608506.3.peg.1959  gatgttcgtgaggttttaggtgaaggtttttctgtttgagtaaaaaataatttaatggtataatаaaga---aaaaag-aat
632518.3.peg.1851  gatgttcgtgaagttttaggtgaaggtttttctgtttgagcaaaaaatagtttaatggtataatataaa---aaaaga-aat
632335.8.peg.565   -atgttcgtgaggttttaggtgaaggtttttctgtttgagaaaaaaatgatttgatggtataatgtaaagaaaaaaagatact
632516.3.peg.1044  -atgttcgtgaggttttaggtgaaggtttttctgtttgagaaaaaaatgatttgatggtataatgtaaagaaaaaaagatact
1121259.3.peg.2306 -atgttcgtgaggttttaggtgaaggtttttctgtttgagaaaaaaatgatttgatggtataatataaagaaaaaaagatact
632292.3.peg.754   gatgttcgtgaggttttaggtgagggtttttctgtttgagtaaaaaatgatttaatggtataatataaagaaaaaaaga-act
                    * ***        ***  ** *    *  **  *  * *   **   *   *  *** ******  * *   ****   * *

31899.10.peg.2606  ttgaaaaaatcattttaaagggaaagtccaaaccaaatattgtatttttgcaaa-ggaggtttaagaaagacaATGGATA
632348.3.peg.640   ttgaaaaaatcattttaaagggaaagtccaaaccaaatattgtatttttgcaaa-ggaggtttaagaaagacaATGGATA
1214564.3.peg.926  --aa-accatcagttaattggtaaatgagaatccaaa--------tttcgtaag-ggaggttcaaggtacaagATGGACA
351627.8.peg.1502  --aa-accaccagttaattggtaaatgagaatccaaa--------tttcgtaag-ggaggttcaaggtacaagATGGACA
1222016.3.peg.861  --aa-accatcagttaattggtaaatgagaatccaaa--------tttcgtaag-ggaggttcaaggtacaagATGGACA
1387569.3.peg.1841 --aa-accaccagttaattggtaaatgagaatccaaa--------tttcgtaag-ggaggttcaaggtataagATGGACA
1387557.3.peg.2455 --aa-accaccagttaattggtaaatgagaatccaaa--------tttcgtaag-ggaggttcaaggtataagATGGACA
1387555.3.peg.2160 --aa-gctacctgtcagcaggcaaaagacaattcaaa--------tttcataaa-ggaggtcaaatgtataaaATGGACA
608506.3.peg.1959  --aa-tctatcatttaacgggtaaacaggaatcctacaattgtatttttataaaaggaggtttaaggtacaaaATGGACA
632518.3.peg.1851  --ca-gccatgagttaactggtaggcgacaatccaaaaattgtatttt-ataaa-ggaggttagaagtacaaaATGGATA
632335.8.peg.565   --aagaacaccaattaacagacaaaaaccaacccaaagattgta-ttttgcaaa-ggaggtttaaaagctaagATGGACA
632516.3.peg.1044  --aagaacaccaattaacagacaaaaaccaacccaaagattgta-ttttgcaaa-ggaggtttaaaagctaagATGGACA
1121259.3.peg.2306 --aagaacaccaattaacaggcaaaaaccaacccaaaaattgta-ttttgcaaa-ggaggtttaaaagctaagATGGACA
632292.3.peg.754   --aa-accaccagttaactggtaaaacacaatccaaaaattgta-tcttacaaa-ggaggtttaaaagctaagATGGACA
                      *    *    *     *  *      **  * *         * *   **  ******            ***** *
```

31899.10.peg.1982 (Athe_1489) *tal*

31899.10.peg.1982	Caldicellulosiruptor bescii strain DSMZ 6725
632348.3.peg.1297	Caldicellulosiruptor kronotskyensis 2002
1121259.3.peg.319	Caldicellulosiruptor acetigenus DSM 7040
608506.3.peg.1162	Caldicellulosiruptor obsidiansis OB47
632292.3.peg.1331	Caldicellulosiruptor hydrothermalis 108
632518.3.peg.1355	Caldicellulosiruptor owensensis OL
632516.3.peg.44	Caldicellulosiruptor lactoaceticus 6A
632335.8.peg.1598	Caldicellulosiruptor kristjanssonii 177R1B
1214564.3.peg.529	Caldicellulosiruptor sp. F32
1222016.3.peg.1294	Caldicellulosiruptor changbaiensis strain CBS-Z
1387557.3.peg.1736	Caldicellulosiruptor sp. Wai35.B1
1387569.3.peg.1292	Thermoanaerobacter cellulolyticus NA10
1387555.3.peg.1346	Caldicellulosiruptor sp. Rt8.B8
351627.8.peg.2214	Caldicellulosiruptor saccharolyticus DSM 8903

```
31899.10.peg.1982  catgttaagtaggttgtaattatacagcaattgctataaaatattaaTacgaaga---aaattaaaagaaagaggtgtt-gtc-agaaATGAAACTTT
521460.8.peg.1558  catgttaagtaggttgtaattatacagcaattgctataaaatattaatacgaaga---aaattaaaagaaagaggtgtt-gtc-agaaATGAAACTTT
632348.3.peg.1297  catgttaagtaggttgtaattatacagcaattgctataaaatattaatacgaaga---aaattaaaagaaagaggtgtt-gtc-agaaATGAAACTTT
608506.3.peg.1162  catgttaagtgtgttgtaattatacaccagttagtataaaatat-aatacgaaga---aaattggaggaaagaggtgtt-gtc-aaaaATGAAACTTT
632292.3.peg.1331  catgttaagtgtgttgtaattatacaccaattagtataaaatat-aatacgaaga---aaattagaggaaagaggtgtt-gtc-agaaATGAAACTTT
632518.3.peg.1355  tatgttaagtgtgttgtaattatacaccagttagtataaaatat-aatacggaga---aaatcagagaaaagaggtgtt-gtc-aaaaATGAAACTTT
1121259.3.peg.319  catgttcgatgtgttgtaattatacagcaattggtataaaatat-tatacgaaga---aaattaaaagaaagaggtgtt-gta-aaaaATGAAACTTT
632335.8.peg.1598  catgttcgatgtgttgtaattatacagcaattggtataaaatat-tatacgaaga---aaattaaaagaaagaggtgtt-gta-aaaaATGAAACTTT
1214564.3.peg.529  catttttagttattgtaattatacagcaattggtataaaataatagtcgaaaaaa-tcttaacttagagagaggtgttagat-caacATGAAACTTT
1222016.3.peg.1294 catttttagttattgtaattatacagcaattggtataaaataatagtcgaaaaaa-tcttaacttagagagaggtgttagat-caacATGAAACTTT
351627.8.peg.2214  catttttagttattgtaatcatacagcaattggtataaaataatagtcgaaaaaaattttaactttaggagaggtgttagat-caacATGAAACTTT
632516.3.peg.44    c-ttataatgatattgttttatgcaacacttggtataaaataataa-acgaaaa-taaaatctttgaaaagaggtgtt-gta-aaaaATGAAACTTT
1387557.3.peg.1736 c-taataatgatattgttttatgcaacacttggtataaaataataa-acgaaaaatacaatctttgaaaagaggtgttagtt-gaagATGAAACTTT
1387569.3.peg.1292 catttttagttattgtaattatatagcaattggtataaaataatagtcgaaaaaa-------ttttaagagaggtgttagct-caagATGAAACTAT
1387555.3.peg.1346 catttttagttattgtaattatgcagcaactggtataaaataatagtcagaaaaaatcttaa-ttgaaaagaggtgtaaaatacaggATGAAACTAT
                     *  *       ****  * **  * **  *  *********         * *               ********          ******** *
```

31899.10.peg.1071 (Athe_0632) *Athe_0630-31-rpi*

31899.10.peg.1071	Caldicellulosiruptor bescii strain DSMZ 6725	632518.3.peg.559	Caldicellulosiruptor owensensis OL
632348.3.peg.2104	Caldicellulosiruptor kronotskyensis 2002	1387557.3.peg.895	Caldicellulosiruptor sp. Wai35.B1
632292.3.peg.2120	Caldicellulosiruptor hydrothermalis 108	1222016.3.peg.666	Caldicellulosiruptor changbaiensis strain CBS-Z
1121259.3.peg.850	Caldicellulosiruptor acetigenus DSM 7040	1387569.3.peg.2003	Thermoanaerobacter cellulolyticus NA10
632516.3.peg.293	Caldicellulosiruptor lactoaceticus 6A	1387555.3.peg.794	Caldicellulosiruptor sp. Rt8.B8
632335.8.peg.659	Caldicellulosiruptor kristjanssonii 177R1B	351627.8.peg.1341	Caldicellulosiruptor saccharolyticus DSM 8903
608506.3.peg.648	Caldicellulosiruptor obsidiansis OB47	1214564.3.peg.3	Caldicellulosiruptor sp. F32

```
31899.10.peg.1071  aaagaatattcttcttttcagatttcacaagaaagaatataatataaaaG
608506.3.peg.648   aaagaatattcttcttttcagatttcgcaagaaagaatataatataaaag
632518.3.peg.559   aaagaatattcttcttttcagatttcgcaagaaagaatataatataaaag
1121259.3.peg.850  aaagaatattcttcttttcagatttcgcaagaaagaatataatataaaag
632335.8.peg.659   aaagaatattcttcttttcagatttcgcaagaaagaatataatataaaag
632516.3.peg.293   aaagaatattcttcttttcagatttcgcaagaaagaatataatataaaag
632348.3.peg.2104  aaagaatattcttcttttcagatttcacaagaaagaatataatataaaag
632292.3.peg.2120  aaagaatattcttcttttcagatttcgcaagaaagaatataatataaaag
1387557.3.peg.895  aaagaatattcttcttttcatatttcgctacaatagatataatataaaag
351627.8.peg.1341  aaagaatattcttcttttcatatttcgctacaatagatataatataaaag
1222016.3.peg.666  aaagaatattcttcttttcatatttcgctacaatagatataatataaaag
1387569.3.peg.2003 aaagaatattcttcttttcatatttcgctacaatagatataatataaaag
1214564.3.peg.3    aaagaatattcttcttttcatatttcgccacaatagatataatataaaag
1387555.3.peg.794  aa-gaatattcttcttttcatatttcgctacaatagatataatataaaag
                   ** ***************** ***** * * **   **************
```

```
31899.10.peg.1071  aatgggaagataaataaaagctttttgaaaagagagggtgcaagag-TTGAAAGAAGCTTATG
608506.3.peg.648   aatgggaaaatggataagagtttttttaaaagagagggtgcagaaa-TTGAAAGAGACTTATG
632518.3.peg.559   aatgggaaaatggataagagtttttg-aaaagagagggtgcagaaa-TTGAAAGAGACTTATG
1121259.3.peg.850  aatgggaaaataaataaaagctttttgaaaagagagggtgcaagag-TTGAAAGAAGCTTATG
632335.8.peg.659   aatgggaaaataaataaaagctttttgaaaagagagggtgcaagag-TTGAAAGAAGCTTATG
632516.3.peg.293   aatgggaaaatgaataaaagctttttgaaaagagagggtgcaagag-TTGAAAGAAGCTTATG
632348.3.peg.2104  aatgggaaaataaataaaagctttttgaaaagagagggtgcaagag-TTGAAAGAAGCTTATG
632292.3.peg.2120  aatgggaaaataaataaaagctttttgaaaagagagggtgcaagag-TTGAAAGAAGCTTATG
1387557.3.peg.895  aatggg--aatagtgaaaagccttttt-tagagagagggtgcaagaacTTGAAACCAGATCATG
351627.8.peg.1341  aatggg--aatagtgaaaagccttttt-tagagagagggtgcaagaacTTGAAACCAGATCATG
1222016.3.peg.666  aatggg--aatagtgaaaagccttttt-tagagagagggtgcaagaacTTGAAACCAGATCATG
1387569.3.peg.2003 aatggg--aatagtgaaaagccttttt-tagagagagggtgcaagaacTTGAAACCAGATCATG
1214564.3.peg.3    aatgggg-aatagtaaaaagtcttttt-tagagagagggtgcaaggatTTGAAACCAAATCATG
1387555.3.peg.794  aatggg--aatagtgaaaagccttttt-tggagagagggtgcaaggatTTGAAACCAAATCATG
                   *                                               ******     * ***
```

Abbreviations:
C. bescii gene IDs are highlighted in yellow. Gene coding regions are in red. Experimentally determined transcription start sites (TSSs) in *C. bescii* are in red and bold capital letter. Predicted -10 and -35 promoter boxes are underlined. Predicted TF-binding sites are highlighted by color boxes.
