# Supplementary figures and images for: Transcriptional Regulation of Plant Biomass Degradation and Carbohydrate Utilization Genes in the Extreme Thermophile Caldicellulosiruptor bescii
Source: mSystems. 2021 Jun 1;6(3):e01345-20. doi: 10.1128/mSystems.01345-20 (PMC8579813; doi:10.1128/mSystems.01345-20)

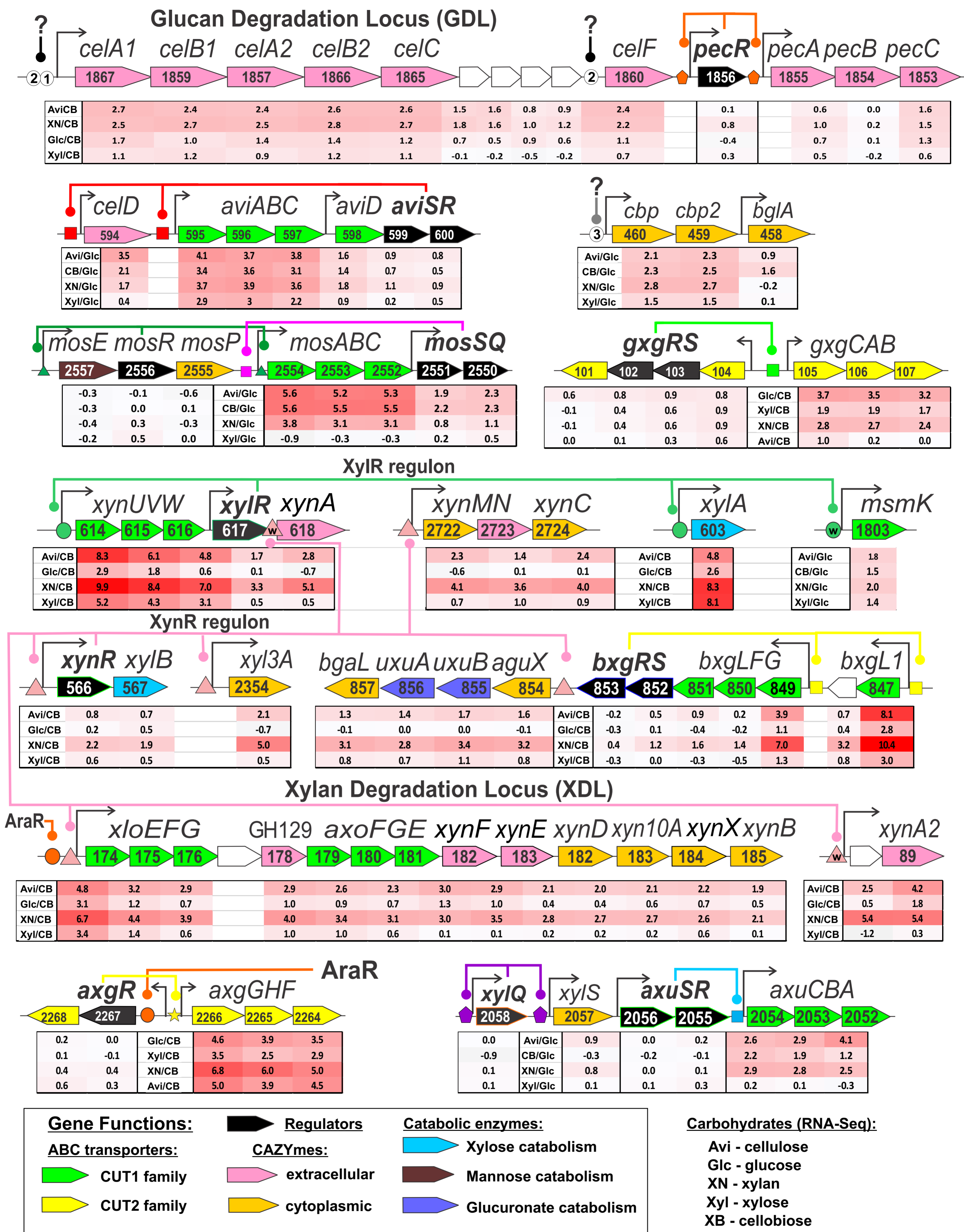

Figure S3.

Supplement: FIG S3 [file msystems.01345-20-sf003.pdf]

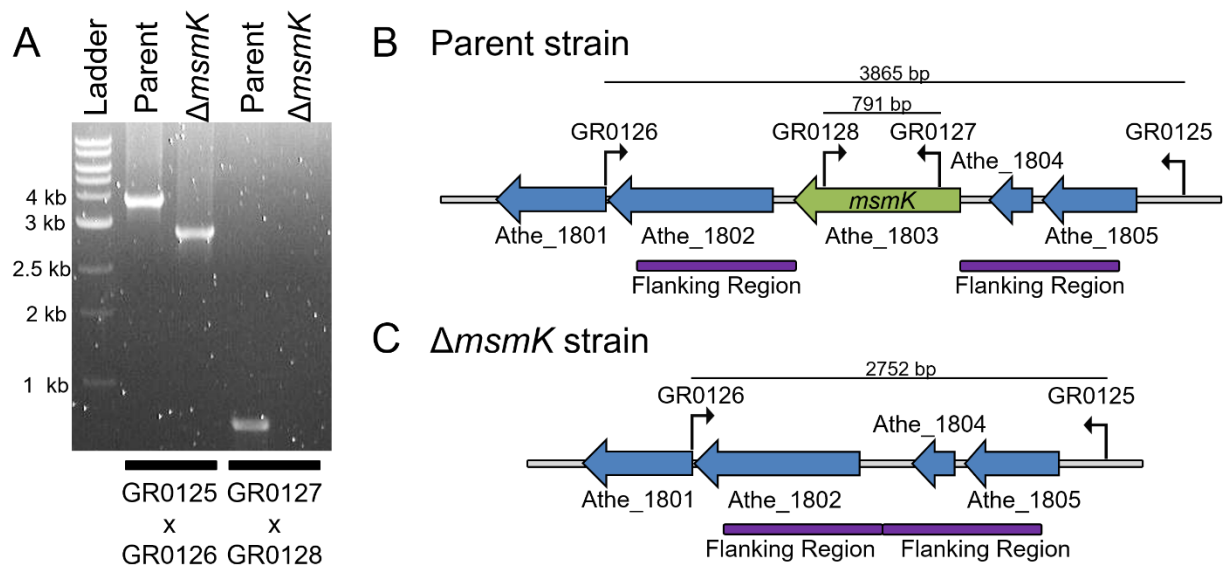

**Figure S2.**

Supplement: FIG S2 [file msystems.01345-20-sf002.pdf]
